# Molecular Docking and Molecular Dynamics Aided Virtual Search of OliveNet™ Directory for Secoiridoids to Combat SARS-CoV-2 Infection and Associated Hyperinflammatory Responses

**DOI:** 10.3389/fmolb.2020.627767

**Published:** 2021-01-07

**Authors:** Neelaveni Thangavel, Mohammad Al Bratty, Hassan Ahmad Al Hazmi, Asim Najmi, Reem Othman Ali Alaqi

**Affiliations:** Department of Pharmaceutical Chemistry, Faculty of Pharmacy, Jazan University, Jazan, Saudi Arabia

**Keywords:** hyperinflammatory, molecular docking, molecular dynamics, olive secoiridoids, SARS-CoV-2 spike (S) protein, main protease, virtual search

## Abstract

Molecular docking and molecular dynamics aided virtual search of OliveNet™ directory identified potential secoiridoids that combat SARS-CoV-2 entry, replication, and associated hyperinflammatory responses. OliveNet™ is an active directory of phytochemicals obtained from different parts of the olive tree, *Olea europaea* (Oleaceae). Olive oil, olive fruits containing phenolics, known for their health benefits, are indispensable in the Mediterranean and Arabian diets. Secoiridoids is the largest group of olive phenols and is exclusive to the olive fruits. Functional food like olive fruits could help prevent and alleviate viral disease at an affordable cost. A systematized virtual search of 932 conformers of 78 secoiridoids utilizing Autodock Vina, followed by precision docking using Idock and Smina indicated that Nüzhenide oleoside (NZO), Oleuropein dimer (OED), and Dihydro oleuropein (DHO) blocked the SARS-CoV-2 spike (S) protein-ACE-2 interface; Demethyloleuropein (DMO), Neo-nüzhenide (NNZ), and Nüzhenide (NZE) blocked the SARS-CoV-2 main protease (M^pro^). Molecular dynamics (MD) simulation of the NZO-S-protein-ACE-2 complex by Desmond revealed stability during 50 ns. RMSD of the NZO-S-protein-ACE-2 complex converged at 2.1 Å after 20 ns. During MD, the interaction fractions confirmed multiple interactions of NZO with Lys417, a crucial residue for inhibition of S protein. MD of DMO-M^pro^ complex proved its stability as the RMSD converged at 1.6 Å. Analysis of interactions during MD confirmed the interaction of Cys145 of M^pro^ with DMO and, thus, its inhibition. The docking predicted IC_50_ of NZO and DMO was 11.58 and 6.44 μM, respectively. Molecular docking and dynamics of inhibition of the S protein and M^pro^ by NZO and DMO correlated well. Docking of the six-hit secoiridoids to IL1R, IL6R, and TNFR1, the receptors of inflammatory cytokines IL1β, IL6, and TNFα, revealed the anti-inflammatory potential except for DHO. Due to intricate structures, the secoiridoids violated Lipinski's rule of five. However, the drug scores of secoiridoids supported their use as drugs. The ADMET predictions implied that the secoiridoids are non-toxic and pose low oral absorption. Secoiridoids need further optimization and are a suitable lead for the discovery of anti-SARS-CoV-2 therapeutics. For the moment, olive secoiridoids presents an accessible mode of prevention and therapy of SARS-CoV-2 infection.

## Introduction

The current pandemic of coronavirus disease-2019 (COVID-19) caused by severe acute respiratory syndrome coronavirus-2 (SARS-CoV-2) has worsened persons' quality of life and socioeconomic status globally. Rigorous research for the prevention and therapy of SARS-CoV-2 infection using vaccines and small molecules are underway. Natural products are inspiring sources of drugs, including antivirals. It is imperative to explore functional foods as therapeutic combat against SARS-CoV-2, as they are accessible and affordable. Focuses on nutritional supplements as a mode of prevention and therapy for SARS-CoV-2 infection have increased (Zabetakis et al., 2020). Olive fruits (olives) and oil are indispensable in the Mediterranean (Martinez-Gonzalez and Martin-Calvo, 2016) and Arabian diet (Al-Ruqaie et al., 2016) and are known for their health benefits. The northern provinces of Saudi Arabia have suitable climatic conditions and cultivate olive tree, *Olea europaea* (Oleaceae) for olive oil and olives (Hemida et al., 2014; Fraihat et al., 2017). Olive oil obtained from olives has earned nutritional and therapeutic value. Especially olive oil polyphenols demonstrated antioxidant, antihyperglycemic, anticancer, antilipidemic, antiviral, anti-inflammatory, cardioprotective, and immunomodulation properties (Rigacci and Stefani, 2016; Gorzynik-Debicka et al., 2018). The phytochemicals of olive leaves have also exhibited beneficial effects on ailments like diabetes, cancer, obesity, bacterial, and viral infections (Medina Pradas et al., 2019; Acar-Tek and Agagündüz, 2020). The green, black forms of olives consumed as table olives are high in plant polyphenols implicated in several diseases (Rahmani et al., 2014; Fernández-Poyatos et al., 2019; Conte et al., 2020). Olives contain secoiridoids, the largest group of polyphenols, in their glycoside and aglycone forms. Olives are also composed of other polyphenols-like flavonoids, triterpenes, and lignans (Hashmi et al., 2015). Olives are the primary source of secoiridoids, whereas oil contains the metabolites of secoiridoids (Sivakumar et al., 2018). Secoiridoids are useful in cancer therapy, heart diseases, neurodegeneration, immunoinflammatory, diabetes, obesity, and aging-related ailments (Celano et al., 2019; Castejón et al., 2020). Few studies are available on the antiviral property of secoiridoids against AIDS and influenza viruses (Omar, 2010; Vilaplana-Pérez et al., 2014). Oleuropein, a secoiridoid, possesses potent antiviral activity against parainfluenza type 3 virus, respiratory syncytial virus, hepatitis, and herpes mononucleosis (Omar, 2010). Oleuropein targeted the surface glycoprotein HIV-1 gp41 and HIV integrase, which blocked HIV entry and replication (Lee-Huang et al., 2007). A recent molecular docking study identified the flavonoids Cyanidin-3-rutinoside and Paeonidin-3-rutinoside from *Olea europaea* as inhibitors of SARS-CoV-2 main protease (Shawky et al., 2020).

The spike protein (S protein), a surface structural protein of SARS-CoV-2, binds with a high affinity to human angiotensin-converting enzyme 2 (ACE-2), which facilitates the entry of the virus into the human host (Wrapp et al., 2020). Therefore, inhibition of spike protein and its interaction with ACE-2 can block virus entry into the host. The main protease (M^pro^) of SARS-CoV-2, also known as 3CLpro, is responsible for the cleavage of polyproteins 1a and 1b, which is crucial for virus replication (Prajapat et al., 2020). The inhibition of virus replication is possible through the inhibition of M^pro^. The S protein and M^pro^ are thus attractive targets for designing and discovering drugs to combat SARS-CoV-2 infection. On the host's invasion by SARS-CoV-2, there is a hyperinflammatory response by the immune system leading to acute respiratory distress and multiple organ failure. Elevated levels of pro-inflammatory cytokines, interleukin-1 (IL1β), interleukin-6 (IL6), and tumor necrosis factor-α (TNFα) in moderate to severe COVID-19 cases contribute to the hyperinflammatory response (Maiti et al., 2020; Tang et al., 2020). It is of paramount importance to block the actions of inflammatory cytokines to improve the patient's well-being and reduce the fatality rate of SARS-COV-2 infected patients. The receptors IL1R (Maiti et al., 2020), IL6R (Chen et al., 2017), and TNFR1 (Maiti et al., 2020) of cytokines are useful targets to evaluate the anti-inflammatory potential of secoiridoids.

One of the rapid and efficient methods to identify potential small molecule therapeutics is utilizing virtual search methods like molecular docking (da Silva Rocha et al., 2019). OliveNet™ is an active directory of phytochemicals reported from olive leaves, olives, and olive oil (Bonvino et al., 2018). The directory includes 222 phenolic compounds, further subdivided into 13 subgroups, among which the 79 secoiridoids are the principal constituents of olive fruits (Owen et al., 2003; Silva et al., 2006; Obied et al., 2007; Alagna et al., 2012; Ghanbari et al., 2012; Kanakis et al., 2013). Secoiridoids biosynthesis in olive trees involves their parent compounds tyrosol and hydroxytyrosol (Ali et al., 2019). Tyrosol and hydroxytyrosol also possess commendable therapeutic properties (Vilaplana-Pérez et al., 2014; Hashmi et al., 2015). It was intriguing that most secoiridoids were exclusive to the olive fruits, and its consumption could combat SARS-CoV-2 infection and associated hyperinflammatory responses that exemplify the viral disease. Hence, we aimed to explore OliveNet™ by a virtual search for olives' secoiridoids capable of combating S protein and M^pro^ of SARS-CoV-2. We also predicted the anti-inflammatory property of secoiridoids to combat the SARS-CoV-2 associated hyperinflammatory response by molecular docking to cytokine receptors IL1R, IL6R, and TNFR1. Furthermore, molecular dynamics studies of the hit secoiridoid-S protein and hit secoiridoid-M^pro^ complexes aided the stability prediction during trajectories for 50 ns.

## Materials and Methods

### Virtual Search of OliveNet™ Directory

We accessed OliveNet™ at https://mccordresearch.com.au/. Autodock Vina was the software used to perform the virtual search of OliveNet™ secoiridoids (Trott and Olson, 2010). The virtual search utilized a target-based docking to the structural and non-structural proteins of the virus (Maia et al., 2020). In this study, the drug targets were mainly the SARS-CoV-2 S protein, a structural protein, and M^pro^, a non-structural protein. The virtual search process comprised the steps described herein.

#### Sources of SARS-CoV-2 Spike Protein, Main Protease, and Secoiridoids

We obtained the three-dimensional X-ray crystallographic structures of SARS-CoV-2 S protein and M^pro^ from the protein data bank using PDB IDs, 6LZG, and 6LU7, respectively. The targeted viral proteins were in their pdb file formats. The secoiridoids reported in OliveNet™ possess molecular weights between 184 and 2,692 g/mol. We filtered and used those secoiridoids with molecular weight <1,100 g/mol. Based on the molecular weight factor, we excluded oleuropein trimer, oleuropein tetramer, and oleuropein pentamer. Instead, we included the parent compounds tyrosol hydroxytyrosol that was within the molecular weight range. Finally, 78 secoiridoids contributed to the virtual search. OliveNet™, PubChem, and ZINC were the databases used to retrieve the structures of the chosen secoiridoids. We applied the canonical smiles of secoiridoids to generate the three-dimensional structures.

#### Preparation of SARS-CoV-2 Spike Protein, Main Protease, and Secoiridoids

Chimera 1.13.2 was the program used to prepare the target proteins (Pettersen et al., 2004). The 6LZG is the receptor-binding domain of the SARS-CoV-2 S protein complexed with the human ACE-2 receptor. The S protein chain B and the ACE-2 chain A were retained, and the ligand atoms were removed. The aim was to dock secoiridoids to the interface of S protein and ACE-2. In the case of 6LU7, it is the SARS-CoV-2 M^pro^ monomer complexed with a peptide inhibitor. The monomer chain A was retained, and the non-standard residues, including the inhibitor, was deleted. Afterward, the prepared protein structures underwent energy minimization to overcome unfavorable backbone and sidechain interactions through the steepest descent of 100 steps under the Amber ff99SB force field. Stabilization of the protein structures involved merging non-polar hydrogens, adding polar hydrogens, and assigning Kollmann charges.

At last, using the Autodock tools program, we assigned partial charges and atom type and saved the stabilized proteins in pdbqt format (Morris et al., 2009). We created and optimized the three-dimensional structures of the secoiridoids and the reference drugs Chloroquine (Wahedi et al., 2020) for S protein and Lopinavir (Kumar et al., 2020) for M^pro^ using Schrödinger's LigPrep facility (Schrödinger Release, 2020). The OPLS3e force field, when applied to the 3D structures of the ligands, generated 932 conformers of secoiridoids, which served as the input ligands for subsequent docking studies. The resultant minimum energy conformations of the secoiridoids were saved in mol2 format.

#### Molecular Docking of Secoiridoids to SARS-CoV-2 Spike Protein and Main Protease

Virtual search by docking secoiridoids to S protein-ACE-2 and M^pro^ utilized the Autodock Vina program. All the 932 conformers were docked to the binding sites identified and presented inside the grid. Repeated precision docking of secoiridoids to the targets using the software Idock (Li et al., 2012) and Smina (Koes et al., 2013) avoided the false-positive identification. We generated the S protein grid, surrounding Asn33, His34, Glu37, Asp38, Lys353, Ala387, Gln388, Pro389, Phe390, Arg393, Lys403, Tyr453, Tyr495, Gly496, Phe497, Ser494, and Tyr505, the critical interface residues of S protein-ACE-2 (Liu et al., 2020a; Shang et al., 2020). For M^pro^, we centered the grid on Cys145 (Gurung et al., 2020; Liu et al., 2020b; Zhang et al., 2020). The parameters for docking were: maximum binding modes and energy enabled; exhaustiveness of search of 50. Precise redocking of the three top-ranked secoiridoids under the same docking setup in Idock and Smina gadgets confirmed the results. Modeling the 3D structures of the resultant complexes of SARS-CoV-2 targets and secoiridoids in Biovia Discovery Studio Visualizer v16.1.0.15350 provided details of the 2D, 3D conformations and the number, nature of intermolecular interactions (BIOVIA, 2015). The top-ranked (hit) secoiridoid for S protein and M^pro^ chosen based on the binding energy and number of binding interactions entered into the subsequent IC_50_ prediction studies.

#### Prediction of Inhibition Constant

The binding affinity of the hit secoiridoids, in terms of the inhibition constant (IC_50_) against S protein and M^pro^, was predicted using Autodock tools (Morris et al., 2009). The search protocol for the best docking conformer consisted of a population of 150 individuals and a maximum of 25,000,000 energy evaluations in each run with other docking parameters at default. Overall, 100 conformations of each compound were generated with IC_50_ values. Finally, we carried out molecular dynamics studies to assess the stability of the complexes of NZO and DMO bound to respective targets.

#### Molecular Dynamics of Apo and Bound Forms of SARS-CoV-2 Spike Protein and Main Protease

Molecular dynamics (MD) simulation helps understand the dynamic motions of the atoms of protein targets and target-ligand complexes (Hospital et al., 2015). MD also unveils the conformational stability of target proteins and ligands before and after the interaction (De Vivo et al., 2016). MD of the apo (unbound) and the hit secoiridoids-bound forms of SARS-COV-2 S protein and M^pro^ were studied using the Desmond program (DE Shaw Research, 2020). After creating the topology, the apoproteins and the protein-ligand complexes were placed in the OPLS3e force field to study the number and stability of interactions. The complex was then immersed into a TIP3P water model at 300°K, maintaining 10 Å from the center of the box. The complex underwent energy minimization up to 5,000 steepest descent steps. Then, added sodium and chloride ions to mimic the *in-vivo* environment. Long-range electrostatic interactions were calculated using the Particle-mesh Ewald (PME) method. The constant temperature and pressure were maintained using a Nose-Hoover thermostat and the Martina-Tobias-Klein method. The motion equations were integrated using the multistep RESPA integrator with an inner time step of 2.0 fs for bonded and non-bonded interactions within the short-range cutoff. Periodic boundary conditions were applied. After equilibration, the target proteins and their complexes with the best secoiridoids were subjected to the production run for 50 ns in the N (total atoms in the system), P (system pressure), and T (system temperature) ensemble. The root mean square deviation (RMSD) analysis, root mean square fluctuation (RMSF), full contacts, and the interaction fractions maintained throughout the MD simulation indicate the protein's stability and ligand's stability in bound form. Origin (2016) software was used to plot the comparative RMSF of the protein and ligand.

### Molecular Docking of Top-Ranked Secoiridoids to Inflammatory Protein Receptors

The three top-ranked secoiridoids obtained from docking to S protein and M^pro^ went through additional docking to cytokine receptors to assess the ability to inhibit the binding, thereby pro-inflammatory cytokine actions. Autodock Vina, Idock, and Smina tools were used for docking. The PDB IDs 1ITB, 1N26, and 1NCF correspond to the 3D structures of IL1R, IL6R, and TNFR1 receptors. We applied the previously prepared energy minimized structures of the secoiridoids for docking. The 3D structures of the cytokine receptors were stabilized using the Chimera tool. Methotrexate served as the standard drug for comparing the secoiridoids' inhibitory effect on IL1R. Methotrexate can directly inhibit the binding of IL1β to IL1R, resulting in inhibition of IL1β mediated cellular responses (Brody et al., 1993). A small-molecule inhibitor of IL6R, Chemiome CID5329098 (Chen et al., 2017), was used as the reference molecule for IL6R inhibition. Physcion-8-glucoside is an inhibitor of TNFR1 (Saddala and Huang, 2019), used as the reference molecule for TNFR1 inhibition. We applied the blind docking method that involves docking ligands to the whole surface of human cytokine receptors.

### Virtual Physicochemical, Pharmacokinetics, and Drug Score Screening

Secoiridoids have novel chemical structures that need an investigation of molecular, pharmacokinetic, and toxicity properties. Examining the secoiridoids' molecular properties like molecular weight, the number of hydrogen bond donor/acceptor groups, and the topological polar surface area, for Lipinski's violation provides insight into the oral bioavailability. The SwissADME server was used to predict the molecular properties and the bioavailability score (Daina et al., 2017). The admetSAR 2.0 online tool helped predict the distribution, toxicity, and LD_50_ (Yang et al., 2019). The OSIRIS property explorer (Osiris, 2020) was used to predict the drug score, which is a combination of drug-likeness score, lipophilicity, hydrophilicity, molecular weight, and the risk of toxicity of secoiridoids. The drug score helps to verify the overall quality of the secoiridoids to be potential drugs.

## Results

### Virtual Search

The virtual search of the OliveNet™ directory using Autodock Vina, Idock, and Smina was successful. The grids generated around the binding site of the 3D crystalline structures of the S protein-ACE-2 receptor interface (6LZG) and the catalytic Cys145 of M^pro^ (6LU7) provided reliable results. The virtual search using Autodock Vina generated 932 energy minimized conformers of the screened secoiridoids, which were then docked to the SARS-CoV-2 targets and graded based on the binding energy. All the docked conformers of secoiridoids had the potential to bind to the SARS-CoV-2 S protein's active site and its interface with ACE-2 with binding energies in the range of −8.9 to −4.1 kcal/mol. Similarly, the olive secoiridoids have shown binding affinities between −8.9 and −4.3 kcal/mol toward the binding site of SARS-CoV-2 M^pro^. Nüzhenide oleoside (NZO), Oleuropein dimer (OED), and Dihydro oleuropein (DHO) secured the top three ranks, respectively, for binding to SARS-COV-2 S protein-ACE-2 receptor complex. Demethyloleuropein (DMO), Neo-nüzhenide (NNZ), and Nüzhenide (NZE) were the three top-ranked secoiridoids exhibiting efficient binding to SARS-COV-2 M^pro^. Hence, the top-ranked secoiridoids passed through the next step of precise docking using the Autodock Vina, Idock, and Smina tools.

### Molecular Docking of Secoiridoids to SARS-CoV-2 Spike Protein and Main Protease

**Figure 1:**
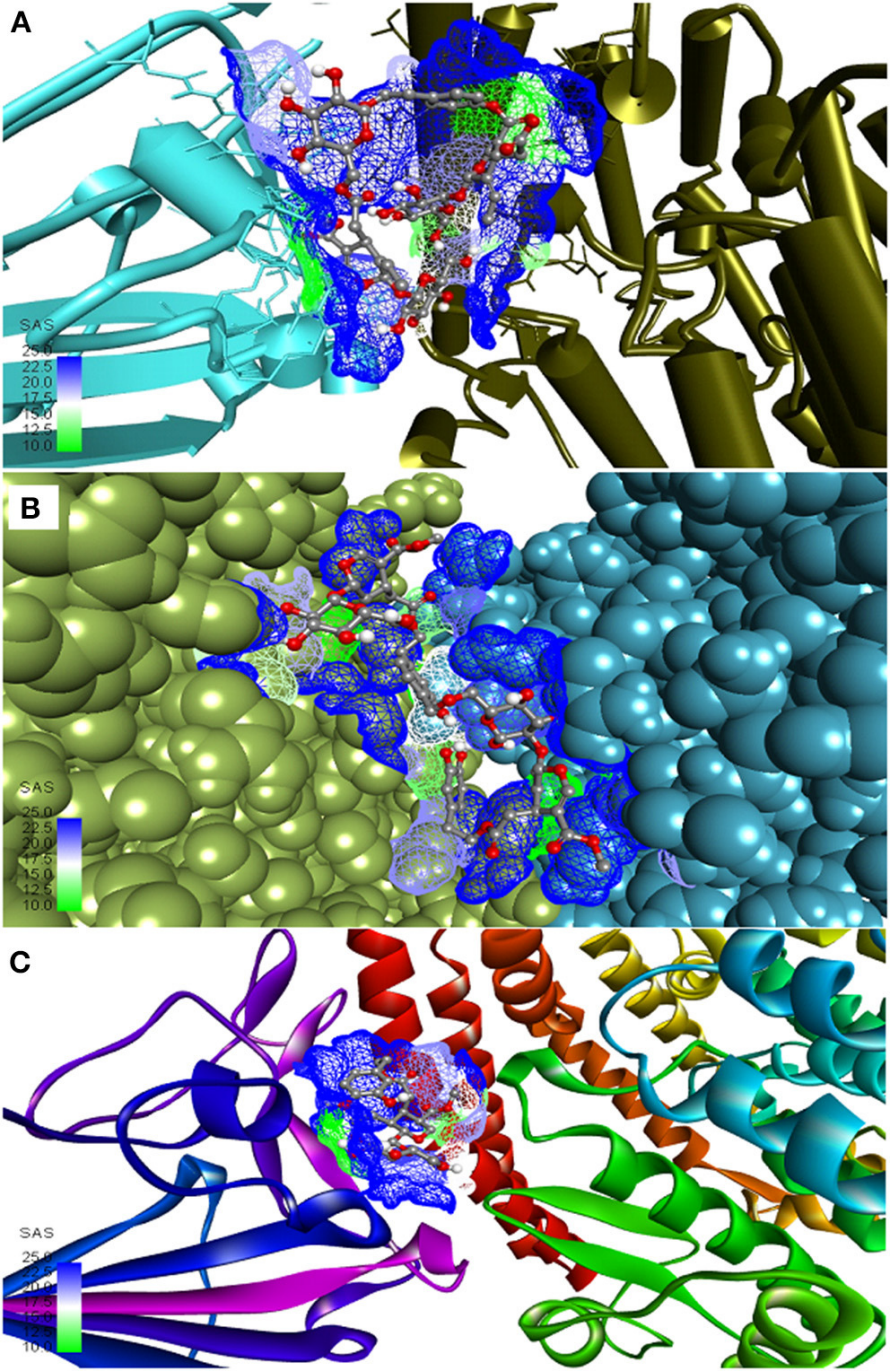
Most active secoiridoids docked to the SARS-CoV-2 spike protein-ACE-2 interface. **(A)** Nüzhenide oleoside **(B)** Oleuropein dimer **(C)** Dihydro oleuropein. Mesh around the binding site represents it's solvent accessible surface (SAS). All models show the spike protein in blue and ACE-2 in green. Panel **(A)** represents the SARS-CoV-2 S protein-ACE-2 in their secondary structure forms, **(B)** represents targets in the CPK model, and ribbons in C represent targets.

**Figure 2:**
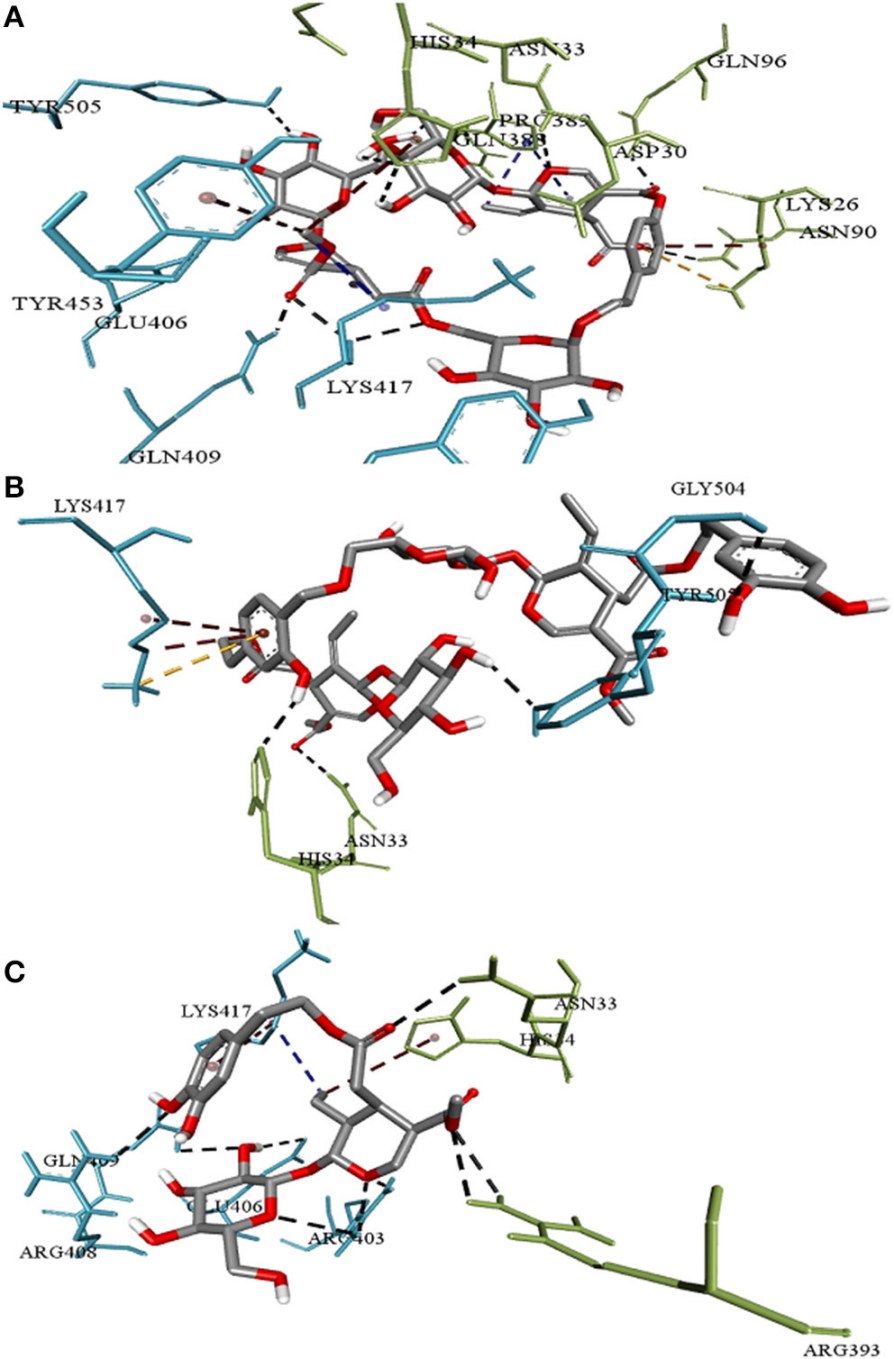
Intermolecular interactions of **(A)** Nüzhenide oleoside **(B)** Oleuropein dimer **(C)** Dihydro oleuropein with SARS-CoV-2 spike protein-ACE-2 interface residues. Interaction with Lys417 of the spike protein is considered crucial for inhibition. Nature of bonds: black—hydrogen bonds; yellow—attractive charge; brown—π-alkyl; blue—alkyl-alkyl interactions.

The three top-ranked secoiridoids on precise docking in Autodock Vina, Idock, and Smina displayed better binding efficiency at the S protein-ACE-2 interface and M^pro^ binding sites than the reference drugs shown in Table 1. The secoiridoid-SARS-CoV-2 target complexes obtained from precise docking in Autodock Vina, when probed in Discovery Studio Visualizer, unraveled the *in-silico* binding inhibitory mechanisms. Figures 1A–C show the 3D best binding poses of NZO, OED, and DHO docked to the S protein-ACE-2 target interface. The intermolecular bonds of NZO, OED, and DHO with the S protein-ACE-2 interface are shown in Figures 2A–C. NZO interacted with the S protein-ACE-2 interface through 10 hydrogen bonds and 1 attractive charge interaction (Figure 2A). Four hydrogen bonds existed with S protein, and six hydrogen bonds were with the ACE-2. Lys417 of the S protein was involved in the formation of two hydrogen bonds with NZO. The attractive charge interaction was between the cationic Lys26 of the ACE-2. Six hydrophobic interactions of π-alkyl and alkyl-alkyl type also existed in the NZO-S protein-ACE-2 complex. Hydrogen bonds were the main forces stabilizing the bound form of NZO as they were shorter than the hydrophobic bonds. OED and DHO interacted through four and nine hydrogen bonds, respectively. OED also established an electrostatic charge interaction with cationic residue Lys417 of the S protein (Figure 2B). Two hydrophobic interactions between Lys417 of S protein and DHO is also significant (Figure 2C). Chloroquine interacted with S protein through a hydrogen bond with Glu406, a π-alkyl interaction with Lys417, besides a hydrogen bond, a π-cation interaction with His34, and an attractive charge interaction with Glu37 of ACE-2.

**Table 1:** Binding energies during precise docking of the three top-ranked olive secoiridoids to SARS-CoV-2 targets.

**SARS-CoV-2 target/PDB ID**	**Secoiridoid**	**Autodock Vina**	**Smina**	**Idock**
		**Binding energy (kcal/mol)**		
S protein-ACE-2/6LZG	Nüzhenide oleoside	−8.90	−9.20	−7.60
	Oleuropein dimer	−8.70	−8.70	−7.04
	Dihydro oleeuropein	−8.70	−7.90	−6.94
	Chloroquine (reference drug)	−5.70	−5.90	−5.69
M^pro^/6LU7	Demethyloleuropein	−8.90	−10.20	−8.85
	Neo–nüzhenide	−8.70	−9.70	−8.46
	Nüzhenide	−8.60	−9.20	−8.11
	Lopinavir (reference drug)	−7.80	−7.30	−7.91

**Figure 3:**
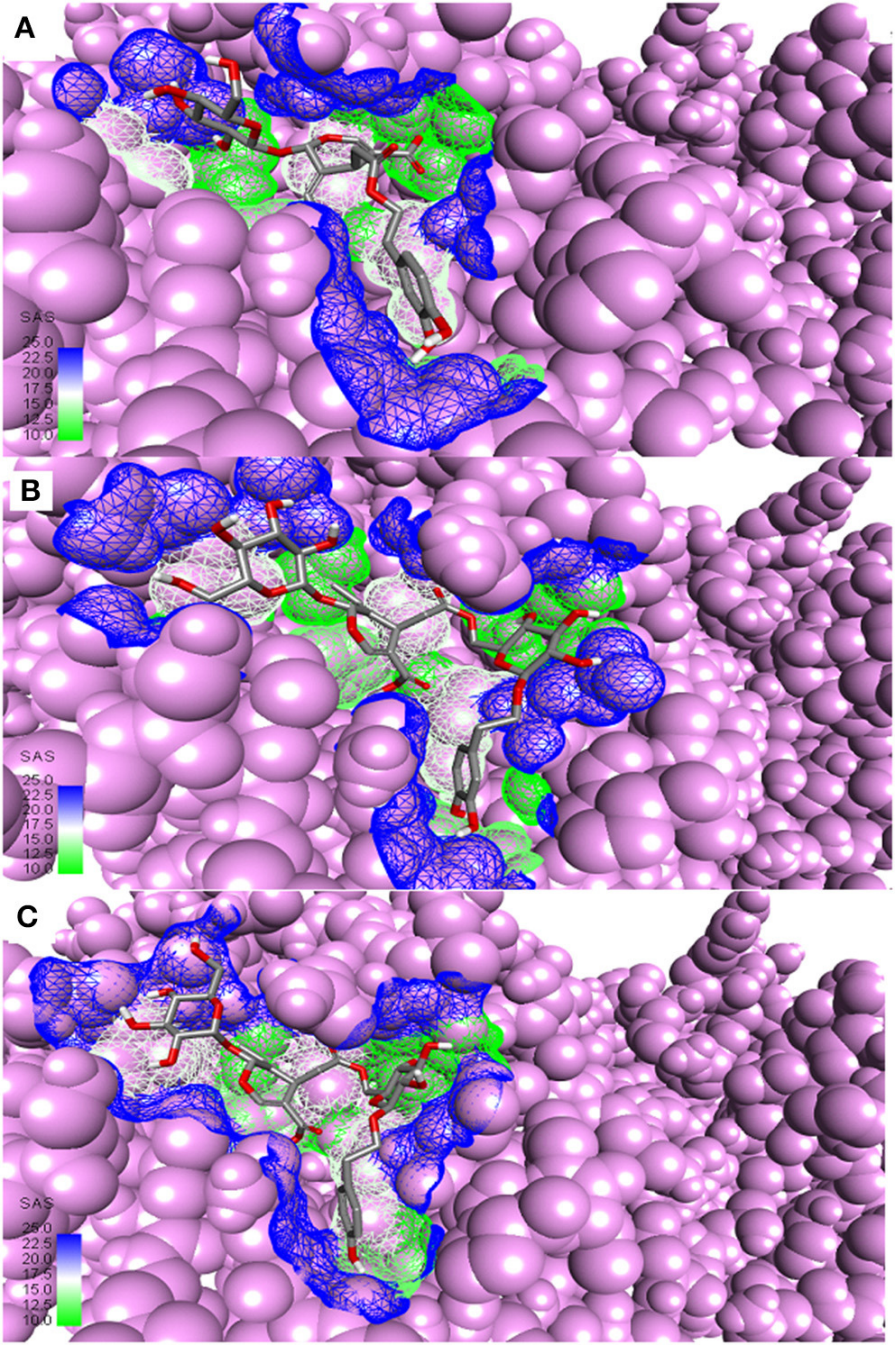
Most active secoiridoids docked to SARS-CoV-2 main protease. **(A)** Demethyloleuropein **(B)** Neo-nüzhenide **(C)** Nüzhenide. The viral protease is in the CPK model. Mesh around the binding site represents it's solvent accessible surface (SAS).

**Figure 4:**
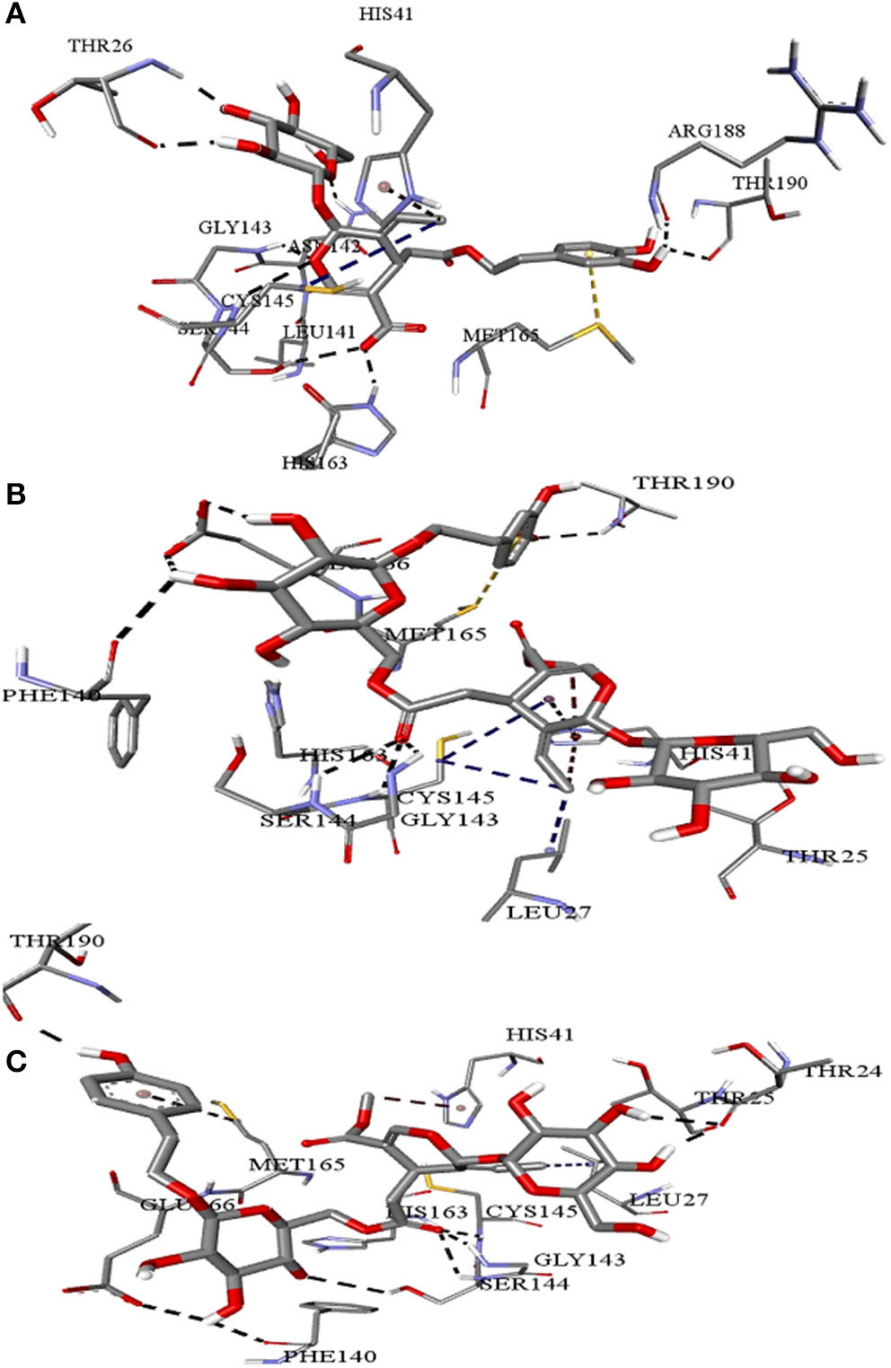
Intermolecular interactions of **(A)** Demethyloleuropein **(B)** Neo-nüzhenide **(C)** Nüzhenide with SARS-CoV-2 main protease. Interaction with Cys145 of the M^pro^ is considered crucial for inhibition. Nature of interactions: black—hydrogen bonds; yellow—π-sulfur; brown—π-alkyl; blue—alkyl-alkyl interactions.

Table 1 shows the results of precise docking of the top-ranked secoiridoids to M^pro^. The binding energies of olive compounds were less than the reference drug Lopinavir. Figure 3 shows the active conformations of the DMO (Figure 3A), NNZ (Figure 3B), and NZE (Figure 3C) bound to the M^pro^. DMO established nine hydrogen bonds with M^pro^ (Figure 4A). Besides other significant interactions, one hydrogen bond with catalytic Cys145 and one hydrophobic π-alkyl interaction with catalytic His41 predicted for DMO indicated its inhibitory property. NNZ established seven hydrogen bonds with M^pro^, of which one bond was with the catalytic Cys145 (Figure 4B). NNZ also interacted with the catalytic duo Cys145-His41 through two hydrophobic alkyl-alkyl and three π-alkyl interactions, respectively. The predicted hydrogen bonds were stable than the hydrophobic interactions due to their shorter bond lengths. NZE also made nine hydrogen bonds with the M^pro^. The active Cys145 residue was engaged in hydrogen bonding with NZE (Figure 4C), while His41 made one π-alkyl interaction with NZE. Lopinavir formed six hydrogen bonds with M^pro^, including one with Cys145. It also interacted through π-anion, π-alkyl, π-sulfur, and π-π stacked forces with Glu166, Met49, Met165, and His41, respectively.

### Prediction of Inhibition Constant

AutoDock predicted IC_50_ against the screened drug targets helped confirm the inhibitory property of the top-ranked secoiridoids NZO and DMO. The IC_50_ of NZO against SARS-CoV-2 S protein was 11.58 μM, and the IC_50_ of DMO against M^pro^ was 6.44 μM.

### Molecular Dynamics of Apo and Bound Forms of SARS-CoV-2 Spike Protein and Main Protease

**Figure 5:**
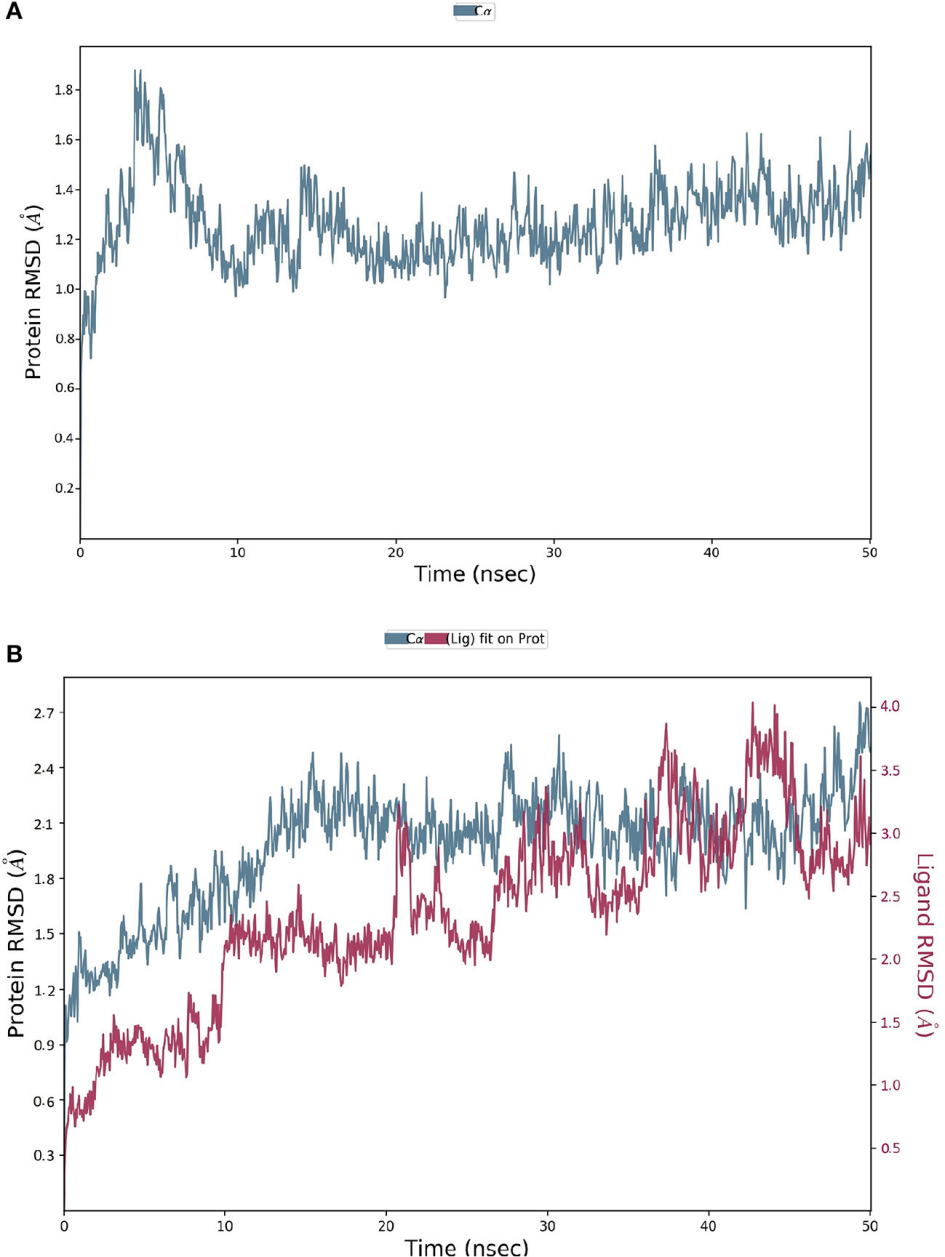
Results of MD of NZO-SARS-CoV-2 S-protein-ACE-2 complex. **(A)** RMSD of the apo form of SARS-CoV-2 S-protein-ACE-2. **(B)** RMSD of the NZO-SARS-CoV-2 S-protein-ACE-2 complex. The RMSDs were stable after 20 ns and converged at 2.1 Å.

**Figure 6:**
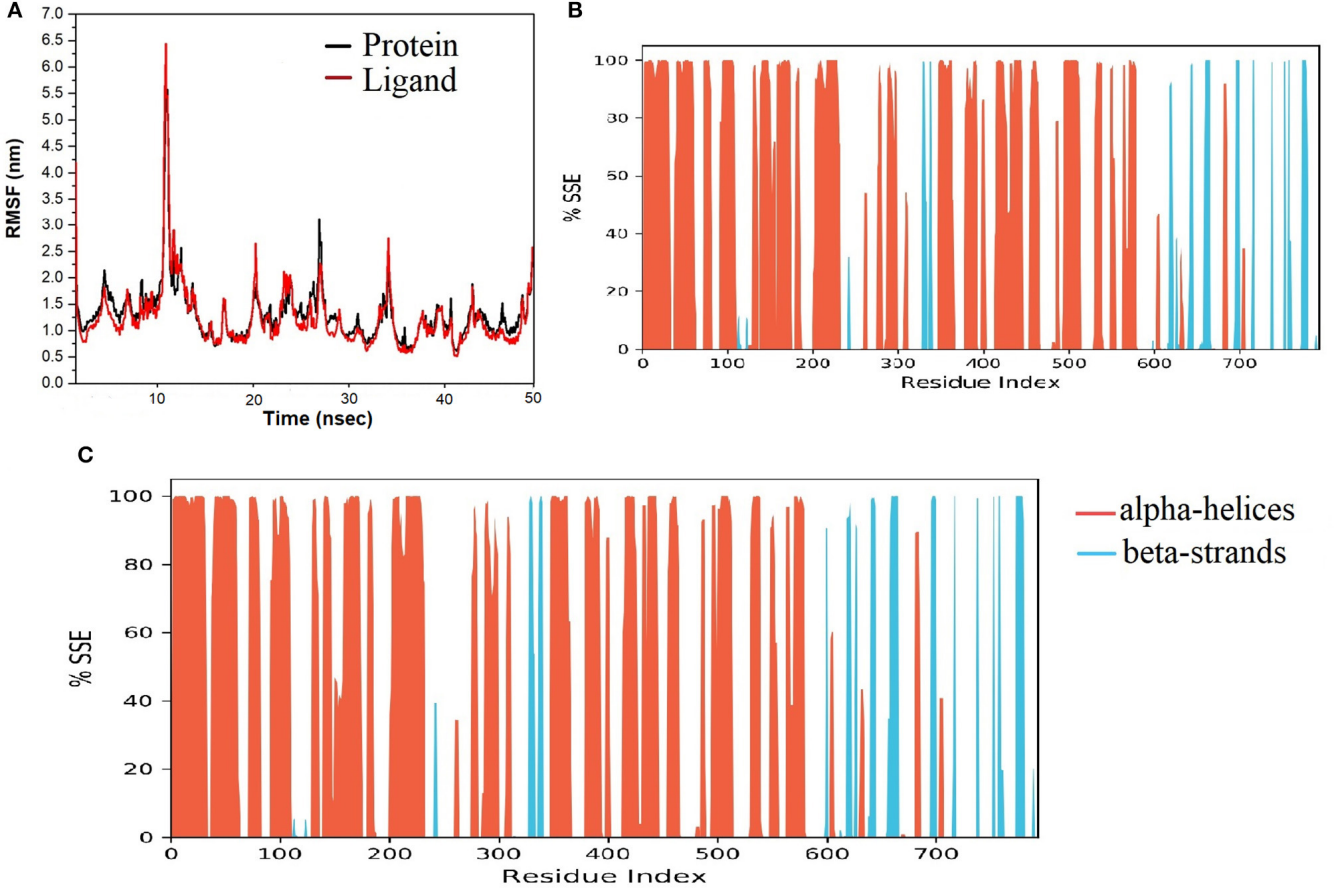
Results of MD of NZO-SARS-CoV-2 S-protein-ACE-2 complex. **(A)** RMSF of the NZO-SARS-CoV-2 S-protein-ACE-2 complex. RMSF of protein and ligand were < 2 Å for most of the simulation time. **(B)** Secondary structure elements (SSE) of the apo form of SARS-CoV-2 S-protein-ACE-2. **(C)** SSE of the NZO-SARS-CoV-2 S-protein-ACE-2 complex. No significant change in the percentage of SSE between the apo and bound proteins indicates conformational stability.

The spike-ACE-2 receptor complex's apoprotein, when subjected to MD simulation studies, had a zero-net charge, 91,253 total atoms, 67.355 mM of Na^+^ ions, 50.690 mM of Cl^−^ ions surrounded by 26,184 water molecules. The final simulation box for the S-protein-ACE-2-NZO complex consisted of 26,110 water molecules, 68.243 mM of Na^+^ ions, and 50.834 mM of Cl^−^ ions, possessing a zero-net charge, and 91,167 total atoms maintained at 300°K. For an MD run of 50 ns, RMSD and RMSF were predicted for the apo and bound forms. A ligand's interaction can ward off unfolding and stabilize the protein (Mazal et al., 2018). Hence, we analyzed the protein's secondary structures before and after docking to understand the conformational changes due to ligand binding. Figure 5 shows the results of RMSD analysis of the spike protein-ACE-2 interface before and after docking NZO. The spike protein-ACE-2 apo form attained an equilibrium after 10 ns (Figure 5A). A stable conformation was achieved by the target protein at RMSD 1.2 Å, an acceptable value for protein structures. RMSD of the NZO complex with S-protein was stable after 20 ns and got fixed and converged at 2.1 Å that disclosed the stability of the complex (Figure 5B). The plot of RMSF for apo spike protein-ACE-2 indicated that the N-terminal and C-terminal residues oscillated above 3.0 Å RMSF. The other secondary structures remained consistent throughout the trajectories, and the RMSF of the protein-ligand complex (Figure 6A) predicted was below 2.5 Å, indicating conformational stability during the simulation. Given that the S-protein-ACE-2 receptor interface targeted involved both proteins' residues, the total number of atoms in the simulated system was high, justifying high RMSF of the terminals. Moreover, it is usual for a protein's N and C-terminals to fluctuate more than the other stable secondary structures like α-helices and β-strands (Kato et al., 2017). Analysis of RMSF of the NZO-S-protein complex (Figure 6A) indicated fewer fluctuations (Figure 6A) shows the secoiridoid had a proper fit into the protein's binding site. The secondary structure of the apo S-protein-ACE-2 (Figure 6B) had 40.50% α-helices and 6.22% of β-strands, while the NZO-docked S protein-ACE-2 (Figure 6C) had 40.23% α-helices and 7.11% β-strands. The negligible increase in the % of β-strands indicated the minimum unfolding of the α-helices during MD of the complex.

**Figure 7:**
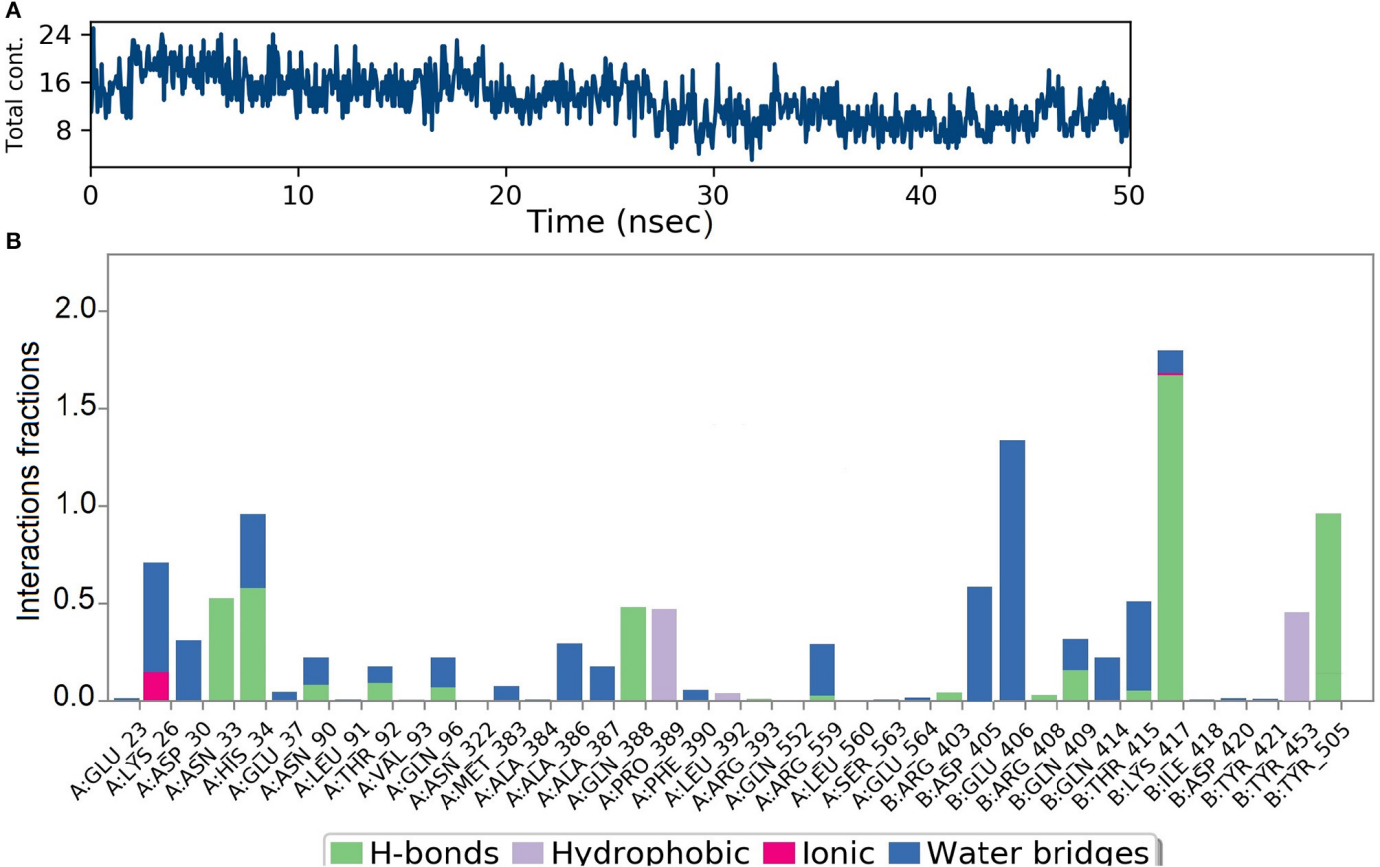
Results of MD of NZO-SARS-CoV-2 S-protein-ACE-2 complex. **(A)** Timeline of the total contacts between NZO and SARS-CoV-2 S-protein-ACE-2 interface. **(B)** Stacked bar chart represents the normalized interactions throughout the trajectory. *Y*-axis represents the % of simulation time the specific interaction was maintained. Asn33 of ACE-2 receptor, chain A maintained hydrogen bond contact for 50% of the simulation time. Lys417 of the S protein, chain B, value above 1 indicates that it maintained multiple hydrogen bond contacts with the ligand throughout the simulation time.

Analysis of intermolecular interactions of the NZO-S-protein-ACE-2 complex during the MD simulation, shown in Figure 7A, indicated that analyzed trajectories exhibited a minimum of 8 and a maximum of 24 contacts confirming the molecular docking results. Additionally, the plot of interaction fractions against the binding site residues (Figure 7B) confirmed that Lys417 of the spike protein furnished multiple contacts (interaction fraction greater than 1.5). Results of molecular docking were consistent with the MD results, as the plot indicated that the binding site residues of the spike protein Gln409, Lys417, and Tyr505 were involved in bonding for 23, 100, and 95.5% of the simulation time, respectively. The hydrophobic interactions of NZO with Pro389 of ACE-2 receptor and Tyr453 of the S protein were also prominent. These interactions contributed to the stability of the complex for 50% of the simulation time.

**Figure 8:**
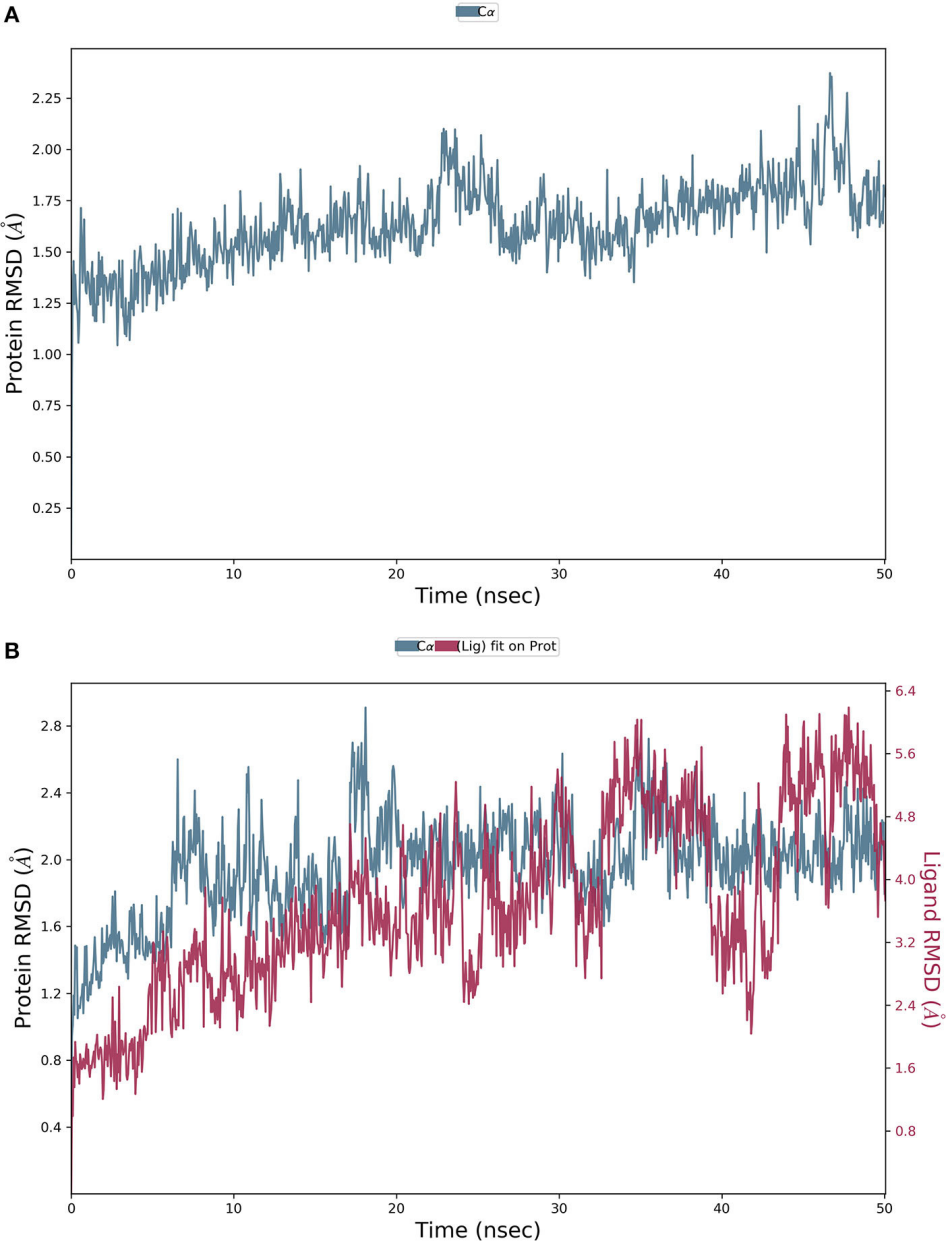
Results of MD of DMO-SARS-CoV-2 M^pro^ complex. **(A)** RMSD of the apo form of M^pro^. **(B)** RMSD of the DMO-M^pro^ complex. RMSDs were stable, <2.4 Å, and converged throughout the run time, indicating the complex's stability.

**Figure 9:**
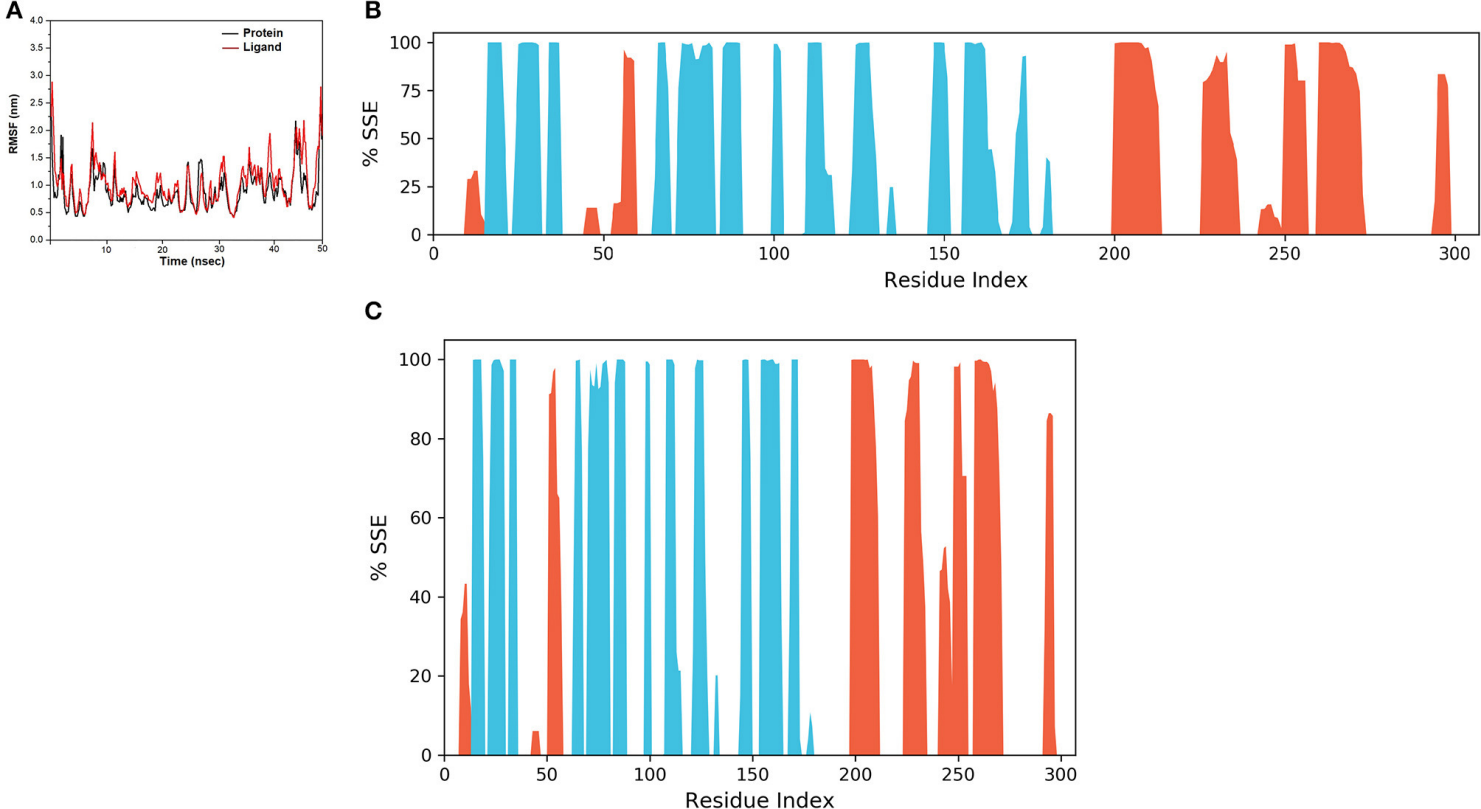
Results of MD of DMO-SARS-CoV-2 M^pro^ complex. **(A)** RMSF of the DMO-M^pro^ complex. RMSF of protein and ligand were <2 Å throughout the simulation time. **(B)** SSE of the apo form of M^pro^. **(C)** SSE of the DMO-M^pro^ complex. M^pro^ was conformationally stable as there was no considerable change in % SSE.

The final system of the apo SARS-CoV-2 M^pro^ was composed of 35,926 total atoms, zero net charges, 50.742 mM of Na^+^, and 50.743 mM of Cl^−^ surrounded by 10,391 molecules of water at a temperature of 300°K. The DMO-M^pro^ complex for MD runs had 35,902 total atoms, zero net charges, 59.636 mM of Na^+^, 50.866 of mM Cl^−^, and 10,366 water molecules. The RMSD of apo M^pro^ in Figure 8A indicated that the system stabilized at the start of the simulation itself. The RMSD remained unchanged and stable at 1.5 Å throughout the 50 ns, except for a slight increase at 25 ns. The RMSD of the docked DMO-M^pro^ equilibrated at 5 ns and remained stable and converged for the rest of the simulation time (Figure 8B). The convergence of the RMSD at 1.6 Å of DMO and M^pro^ in the complex occurred at 12 ns. The convergence of RMSD values indicated the DMO and M^pro^ maintained their contacts throughout the MD. The RMSF of the docked DMO-M^pro^ (Figure 9A) was commendable as it was <1.5 Å. The RMSF of the ligand <2 Å indicated its proper fit into the protein's binding site throughout the MD run. The analysis of secondary structures of the apo (Figure 9B) and docked forms of M^pro^ (Figure 9C) revealed that it achieved a stable conformation without much unfolding after interaction with the secoiridoid. The apo M^pro^ was made up of 16.57% of α-helices and 23.04% of β-strands; M^pro^ docked to DMO was composed of 18.09% of α-helices and 23.70% of β-strands indicating the conformational stability of the protein.

**Figure 10:**
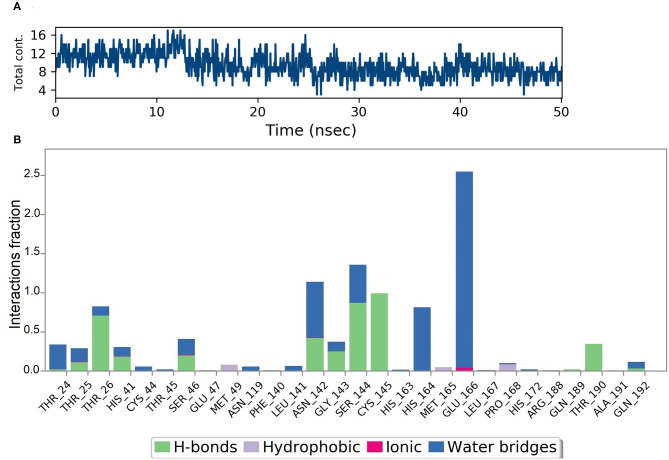
Results of MD of DMO-SARS-CoV-2 M^pro^ complex. **(A)** Timeline of the total contacts between DMO and M^pro^. **(B)** Interactions fractions during simulation time. Cys145 of M^pro^, a crucial residue for inhibition of M^pro^, maintained hydrogen bonds with DMO for 100% of simulation time.

Figure 10A shows that a minimum of nine hydrogen bonds was maintained between DMO and M^pro^ from 12 to 50 ns of MD simulation time. Probing the interactions fractions during the MD trajectories confirmed that DMO could inhibit Cys145 of the M^pro^ for 100% of MD run time (Figure 10B). Besides, Thr26, His41, Asn142, Gly143, Ser144, and Thr190 also contributed considerably toward the interactions of M^pro^ with DMO. Hydrophobic interactions did not significantly contribute to the stability of the complex of DMO with M^pro^. The interactions revealed by MD were in correlation with intermolecular interactions predicted by molecular docking of DMO to M^pro^.

### Molecular Docking of Top-Ranked Secoiridoids to Inflammatory Protein Receptors

**Figure 11:**
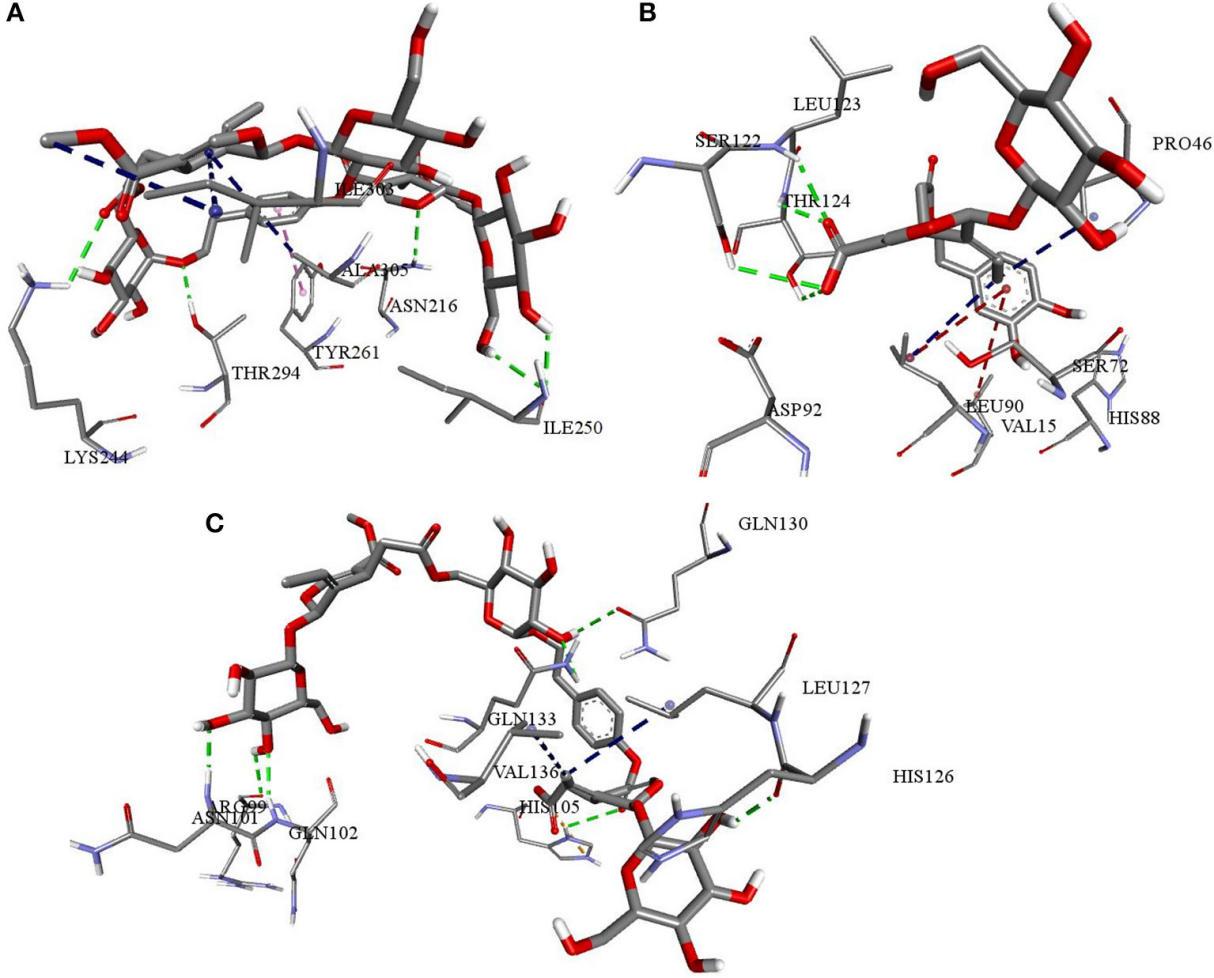
Interactions of NZO and DMO with cytokine receptors **(A)** NZO interactions with IL1R **(B)** DMO interactions with IL6R **(C)** NZO interactions with TNFR1. Green color bonds are the hydrogen bonds, Red color bonds are the π-alkyl, and Blue color bonds are the alkyl-alkyl interactions.

We used Autodock Vina, Idock, and Smina for molecular docking to avoid false-positive identification of potential cytokine receptor inhibitors. The docking tools evaluated the binding energies and scored the secoiridoids. The six secoiridoids, when docked to the inflammatory cytokine receptors, exhibited good binding affinity except for DHO. IL1R interactions with the reference drug Methotrexate was similar to the interactions of secoiridoids. The binding site for the inhibitors of IL1R shown in Figure 11A comprised of Asn216, Leu237, Asp239, Ala241, Lys244, Ile250, Glu252, Glu259, Tyr261, Thr277, Thr294, Ile303, Ala305, and Tyr307 shows a high binding affinity with Methotrexate and NZO due to less binding energy of −7.8 kcal/mol. NZO and DMO, the identified SARS-CoV-2 inhibitors, can inhibit IL1R because of stronger hydrogen bond interactions with Ile250 and Tyr261 compared to Methotrexate.

Docking calculations predicted Chemiome CID5329098 to be less active than the olive secoiridoids, though both compounds occupied the same binding site. The binding site of IL6R was made up of Glu34, Lys45, Pro46, Ala47, Arg54, Agr65, Ser72, Asn74, Leu90, Ser122, Leu123, and Thr124. NNZ made vital hydrogen bond contacts with Ala47 and Ser72 of IL6R characteristic for its inhibition. DMO, as in Figure 11B, established hydrogen bond contacts with Ser122, Leu123, Thr124, and hydrophobic contacts with Pro46. The interactions of Chemiome CID5329098 and DMO precisely correlated with each other. NZO ranked fourth due to high binding energy and interacted with residues different from Chemiome CID5329098 and DMO but occupied the same binding site.

NZO was more efficient than Physcion-8-glucoside, a default TNFR1 inhibitor, and other secoiridoids in binding to TNFR1. All the secoiridoids filled a binding cavity outlined by residues Arg77, Arg99, Asn101, Gln102, His105, His126, Leu127, Gln130, Lys132, Gln133, and Val136. NZO, as shown in Figure 11C, interacted with TNFR1 through seven hydrogen bonds and one electrostatic attractive charge interaction. Physcion-8-glucoside formed four hydrogen bonds, one each with Ser74, Arg77, Arg104, and Lys132. The reference inhibitor and the secoiridoids occupied the same binding site. Physcion-8-glucoside and the secoiridoids similarly interacted with Lys132.

### Virtual Physicochemical, Pharmacokinetics, and Drug Score Screening

Olive secoiridoids are natural products possessing new, intricate structures. Their physicochemical and ADMETox properties influence the biological activity; hence we proceeded to predict them. Table 2 presents the results of virtual physicochemical, pharmacokinetics, and drug score screening of the six-hit secoiridoids. The predicted human intestinal absorption of secoiridoids was lesser than Lopinavir. All the secoiridoids violated Lipinski's rule of five due to their molecular weight higher than 500, the number of hydrogen bond acceptors like oxygen atoms greater than ten, and the number of hydrogen bond donors like -OH greater than five. Secoiridoids possess a topological polar surface area >150 Å^2^, which might pose problems in oral absorption, but the predicted bioavailability was favorable. Secoiridoids are non-irritant, non-mutagenic, non-tumorigenic, and are safe on the reproductive system as predicted by SwissADME. The admetSAR predicted all six secoiridoids to achieve subcellular distribution to the mitochondria. Secoiridoids were non-inhibitors of P-glycoprotein, CYP450 1A2, CYP2C19, CYP450 2C9, CYP450 2D6, and CYP450 3A4 enzymes. The non-inhibition of these hepatic metabolizing enzymes by secoiridoids indicates that they will not interfere with the drug metabolism and are safe to co-administer (Showande et al., 2019). The admetSAR tool predicted that secoiridoids are substrates for P-glycoprotein and CYP450 3A4. If a molecule is a substrate for P-glycoprotein, it may face the problems of low bioavailability and drug resistance; therefore, secoiridoids may suffer from this disadvantage (Srivalli and Lakshmi, 2012). Predicted blood-brain barrier penetration of secoiridoids was less than the standard drug Lopinavir. Predicted acute oral toxicity (LD_50_) of secoiridoids was higher than 2000 mg/kg (on the conversion of predicted values in Table 2 in mol/kg to mg/kg), indicating that they are safe in humans. The overall drug score was in favor of the secoiridoids ranging from 0.220 to 0.359. It is a combination of cLogP (lipophilicity), log S (hydrophilicity), molecular weight, drug-likeness, mutagenicity, tumorigenicity, irritant, and reproductive effects. Osiris predicted drug score for Lopinavir was 0.168.

**Table 2:** Results of virtual physicochemical, pharmacokinetics, and drug score screening.

**Seco-iridoids**	**MW**	**LogP_**O/W**_**	**HBA**	**HBD**	**TPSA (Å^2^)**	**n/viol**	**BA**	**Abs**	**BBB**	**LD_**50**__**MOL/KG**_**	**Drug score**
Nüzhenide oleoside	1058.98	3.05	27	12	412.57	3	0.7571	0.4694	0.2742	3.693	0.240
Oleuropein dimer	1077.04	5.01	25	10	372.11	3	0.7143	0.4159	0.6847	3.488	0.220
Dihydro-oleuropein	544.55	2.99	12	13	201.67	3	0.8857	0.5871	0.2398	3.258	0.340
Demethyl-oleuropein	526.49	0.65	13	7	212.67	3	0.8143	0.3865	0.3135	3.534	0.359
Neo-nüzhenide	702.65	2.66	18	9	280.80	3	0.7714	0.3731	0.3406	3.365	0.270
Nüzhenide	686.65	2.78	17	8	260.59	3	0.7571	0.3869	0.3007	2.982	0.270
Lopinavir	628.81	3.44	5	4	120.00	1	0.6857	0.9624	0.9104	2.994	0.168

*MW, molecular weight in g/mol; HBA, number of hydrogen bond acceptor groups; HBD, number of hydrogen bond donor groups; TPSA, topological polar surface area; n/viol, number of Lipinski's violation; BA, human oral bio-availability; Abs, human intestinal absorption; BBB, bloo brain barrier permeation; LD_50_, acute toxicity in rats*.

## Discussion

The Mediterranean and Arabian diet consist of consuming olive fruits (olives) regularly. OliveNet™ is the exclusive, unique database of phytochemicals of the olive tree, *Olea europaea*. The largest group of polyphenols called secoiridoids reported in OliveNet™ were mainly from olives (MacCord Research OLIVEAMINE®, 2017). The other groups of phenols like Catechol, Gallic acid, flavonoids like Hesperidin, Luteolin, Quercetin, Rutin reported in the OliveNet™ have undergone rigorous virtual screening for anti-SARS-CoV-2 activity as they are commonly present in most of the plants. The research question was whether the consumption of olives could prevent SARS-CoV-2 entry and replication. Therefore, the study aimed to explore the secoiridoids of olives reported in OliveNet™ directory for their potential to combat SARS-COV-2 entry, replication by inhibition of the interaction of SARS-CoV-2 spike protein with human ACE-2 receptor, and inhibition of SARS-CoV-2 M^pro^. The methodology was to search OliveNet™ by virtual means applying molecular docking using the Autodock Vina technique. The three-dimensional structure minimization using LigPrep yielded 932 conformers of secoiridoids. Virtual search involved molecular docking of all the 932 conformers to S protein and M^pro^ in Autodock Vina. The Autodock Vina technique scores the binding poses based on the binding energy in kcal/mol. The secoiridoids NZO, OED, and DHO topped the score list for the best inhibitory potential against the SARS-CoV-2 S protein-ACE-2 receptor. NNZ, NZE, and DMO were the top scorers for their lowest binding energies to interact and inhibit SARS-CoV-2 M^pro^.

We repeated the molecular docking of the above-mentioned top-ranked conformers of secoiridoids using Autodock Vina, Idock, and Smina techniques for precision. Virtual search predictions were precise because we obtained similar results with all three techniques applied for docking. On probing the 3D structure of the complexes obtained from precise docking, the secoiridoids' inhibitory binding to SARS-CoV-2 targets was explicit. The residues Glu406, Arg408, Gln409, Lys417, Tyr453, Gly504, and Tyr505 of the S protein and Lys26, Asp30, Asn33, His34, Asn90, Gln96, Gln388, Pro389, and Arg393 residues of the ACE-2 receptor marked the binding site for olive secoiridoids. Asp30, Asn33, His34, Pro389, Arg393, Lys417, Tyr453, and Gly504 amino acids are crucial for the binding of the SARS-CoV-2 S protein with human ACE-2 receptor, hence for its entry (Wahedi et al., 2020). The potential of olive secoiridoids to bind to the critical residues at the interface suggests that they can prevent the viruses' S protein binding to ACE-2 and block the entry of SARS-CoV-2 into the host (Khan et al., 2020). Olive secoiridoids exhibited lesser binding energy and higher binding efficiency than Chloroquine. Though the binding site was the same, Chloroquine made lesser hydrogen bonds than secoiridoids.

Exploring the 3D structures of secoiridoids bound to M^pro^ provided insight into the mechanism of binding and inhibition. The binding site of M^pro^ was composed of Thr24, Thr26, Leu27, His41, Met49, Phe140, Leu141, Asn142, Gly143, Ser144, Cys145, His163, Met165, Glu166, Arg188, Gln189, and Thr190. The SARS-CoV-2 M^pro^ is a cysteine protease, and targeting the catalytic Cys145-His41 residues is indispensable for achieving efficient inhibition of virus replication *in-vivo* (Liu et al., 2020b, Zhang et al., 2020). The secoiridoids were able to bind to the active residues Cys145 and His41 of M^pro^, indicating potential virus replication inhibition. Olive secoiridoids and Lopinavir interacted similarly with Cys145 and His41, but Lopinavir's binding energy was more than that of secoiridoids. The IC_50_ values of the most active secoiridoids confirmed their inhibitory potential against SARS-CoV-2 S protein and M^pro^. Therefore, the secoiridoids NZO and DMO were chosen against S protein and M^pro^, respectively, and carried over to molecular dynamics simulation studies.

Molecular dynamics of apoproteins and protein-ligand complexes constitute a significant paradigm in assessing the conformational stability of protein and its complex with any ligand in a simulated biological environment (Hospital et al., 2015). The prediction of RMSD and RMSF values of the protein's trajectories alone and its ligand docked structure throughout the simulation time is a valuable tool to determine the validity of the molecular dynamics protocol, stability of protein structure, and its interactions with the ligand (Kato et al., 2017). The RMSD and RMSF of the apo SARS-CoV-2 S protein-ACE-2 receptor and its NZO docked complex were <3.0 Å. There was no variation in the RMSD and RMSF values between the apo and docked forms of the S protein, indicating that the conformational change induced by the ligand was minimum. Also, protein and ligand RMSD during the simulation were stable and converged, revealing the stability of S-protein-NZO interactions.

Furthermore, the lack of significant change in the percentage composition of the protein's secondary structure elements before and after docking confirmed that the protein existed in a stabilized conformation during the molecular dynamics simulation. When we subjected the SARS-CoV-2 M^pro^ in its apo and DMO docked forms to MD run, it was clear that the systems stabilized well below 2 Å, starting from 0 to 50 ns. The system also exhibited a constant, converged RMSD revealing the stability of M^pro^-DMO interactions. A significant proportion of interaction of DMO with Cys145- His41, and Ser144 suggests a potent inhibition of the function of M^pro^. There was no considerable variation in the secondary structure elements of M^pro^ before and after binding to DMO, which suggested that the ligand has not induced a significant conformational change.

Molecular docking results followed by molecular dynamics simulation studies were consistent and identified Nüzhenide oleoside and Demethyloleuropein as potential inhibitors of the SARS-CoV-2 S protein-ACE-2 interface and M^pro^, respectively. The identified olive secoiridoids formed strong stabilizing hydrogen bond interactions with crucial residues of virus targets, suggesting that the compounds could inhibit SARS-CoV-2 entry into the host and its replication.

Hyperinflammatory responses due to cytokine storm after SARS-CoV-2 infections are fatal. Therefore, it is essential to identify and treat hyperinflammatory responses at an early stage of COVID-19. Current clinical interventions include the use of glucocorticoids, Tocilizumab, Chloroquine, and other inhibitors of IL1, IL6 (Gao et al., 2020). To explore the anti-inflammatory potential of the top-ranked secoiridoids, we performed molecular docking to three receptors of inflammatory proteins IL1β, IL6, and TNFα. Molecular docking utilizing the same three docking tools was reliable in identifying cytokine receptor inhibitors from olives. DHO did not show good binding to any of the inflammatory cytokine receptors. The olive secoiridoids NZO and DMO, which emerged from the study with high inhibitory potential toward the SARS-COV-2 S protein and M^pro^, can inhibit inflammatory actions of IL1β, IL6, and TNFα by blockade of their receptors. The binding sites and binding modes of secoiridoids with cytokine receptors were similar to the reference molecules. The NZO and DMO can bind and significantly inhibit IL1R, IL6R, and TNFR1 *in-silico*. The binding of secoiridoids to cytokine receptors shall inhibit the binding of inflammatory cytokines leading to an anti-inflammatory effect that may be beneficial in COVID-19. The cytokine receptor antagonistic property of NZO, DMO, and other secoiridoids was comparable to Methotrexate, Physcion-8-glucoside, and Chemiome CID5329098, the reference inhibitors. The binding of secoiridoids, a significant number of interactions with IL1R, IL6R, and TNFR1 indicate that they can combat cellular inflammatory responses, which need further investigations to confirm the cytokine-inhibitory potential.

The multiple modes of action of the secoiridoids resulting in inhibition of SARS-CoV-2 entry, replication, and inhibition of associated hyperinflammatory responses present a feasible array of therapeutic molecules. Though the secoiridoids are potential enough to combat the SARS-CoV-2 S protein and M^pro^, their physicochemical properties do not favor their drug-likeness. The overall drug score of the secoiridoids was acceptable with a safe pharmacokinetic profile. Secoiridoids contain chemical structures with more than 10 hydrogen bond donors and acceptor groups, as predicted by the online tools SwissADME, admetSAR, and Osiris. High molecular weight and oxygen atoms greater than 10 might present problems in oral absorption. However, olive fruits and olive oil are well-known functional foods (Alagna et al., 2012; Hashmi et al., 2015; Rigacci and Stefani, 2016). The reported *in-vivo* bioavailability of olive oil phenols, which have molecular weight, the number of hydrogen bond donors/acceptor groups, and topological polar surface area similar to the hit secoiridoids, was <66 mol/100 g (Vissers et al., 2004). Therefore, the oral absorption of secoiridoids from olives needs investigation. Nevertheless, the drug score for all the secoiridoids was higher than the reference drug Lopinavir.

**Figure 12:**
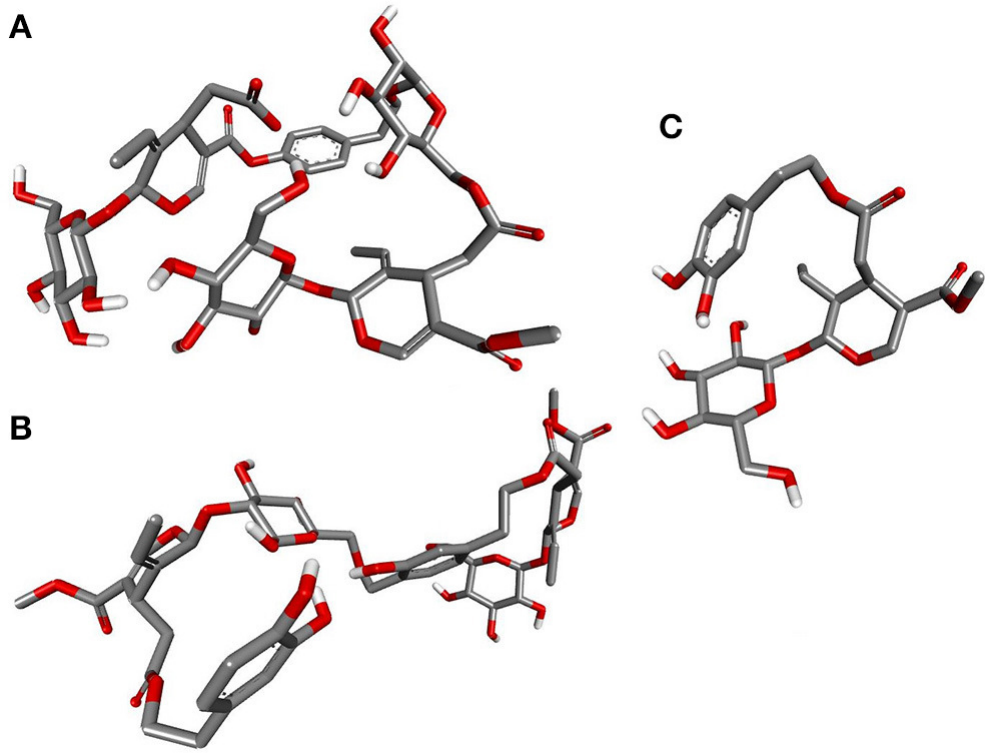
Closeup of 3D conformations of **(A)** Nüzhenide oleoside **(B)** Oleuropein dimer **(C)** Dihydro oleuropein exposing the shape of the active secoiridoids at the SARS-CoV-2-ACE-2 interface. The perfect “U” shape acquired by the ligands is significant for binding.

**Figure 13:**
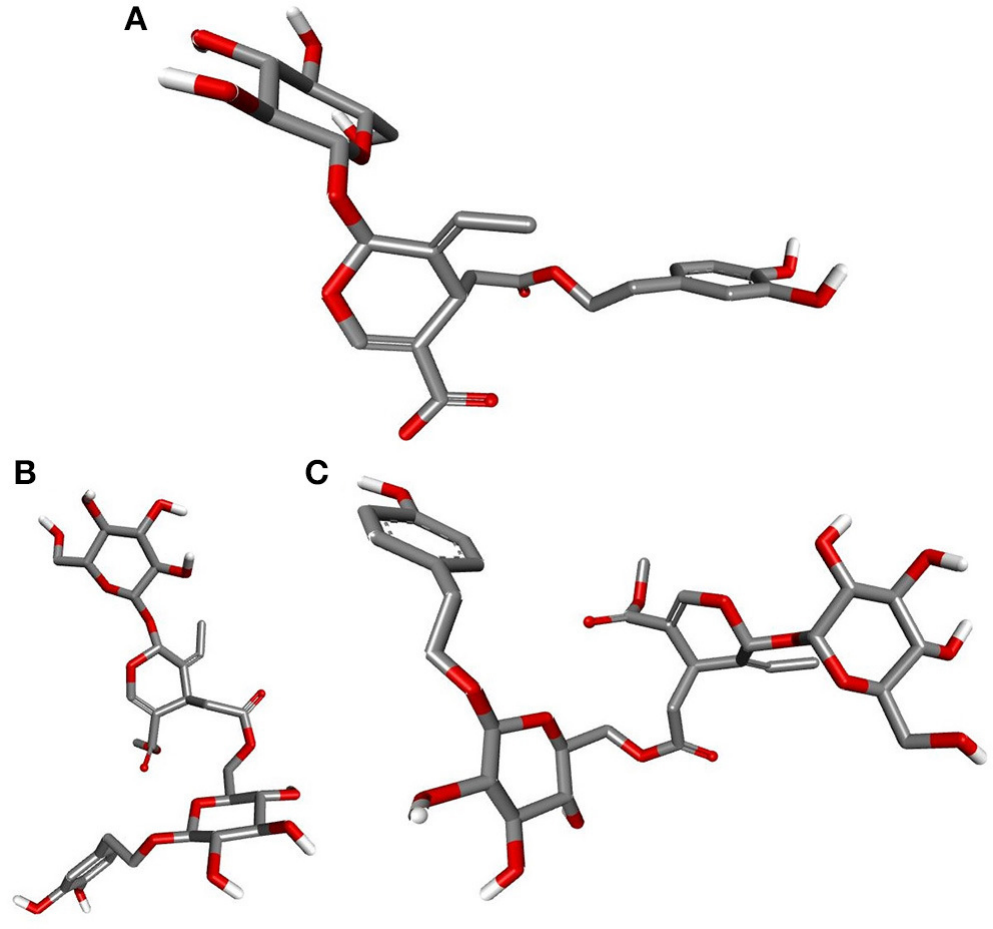
Closeup of 3D conformations of **(A)** Demethyloleuropein **(B)** Neo-nüzhenide **(C)** Nüzhenide showcasing the shape of the active secoiridoids inside the binding cavity of SARS-CoV-2 M^pro^. Secoiridoids assumed an “L” shape favoring the intermolecular interactions.

Deducing the shape and pharmacophores of the identified inhibitors' active conformers inside the binding site during interaction is essential for further lead optimization and design. Figure 12 provides the active conformations of the three top-ranked secoiridoids neutralizing the SARS-CoV-2 S protein: (A) Nüzhenide oleoside (B) Oleuropein dimer (C) Dihydro oleuropein. All the compounds have adopted a “U” shaped geometry in the binding site at the S protein-ACE-2 receptors' interface. Dihydro oleuropein though a smaller molecule compared to Nüzhenide oleoside and Oleuropein dimer, has also assumed a “U” shape. The larger the molecule's size, the more influential the occupancy at the interface as the binding cavity is broad, covering both the SARS-COV-2 S protein and the ACE-2 receptor. Hence, maintaining the same number of rings in Nüzhenide oleoside during future drug design shall provide the appropriate size for efficient binding and help establish necessary hydrophobic contacts. The hydrogen bonds with the crucial residues Lys417 of S protein and Asp30 of ACE-2 receptor involved the ligand's electronegative oxygen atoms in the bridges and the side chains. Lipinski's violations for the lead likeness could be resolved by reducing the hydrogen bond acceptor atoms like oxygen in the rings. Figure 13 represents the active conformations of the three top-ranked secoiridoids (A) Demethyloleuropein (B) Neo-nüzhenide (C) Nüzhenide extracted from the binding sites of M^pro^. All three secoiridoids have accommodated inside the binding site of M^pro^ in similarly extended “L” conformations. It was clear that small molecules like Demethyloleuropein can bind to M^pro^'s active site more effectively than larger secoiridoid like Nüzhenide oleoside. The presence of phenolic hydroxyl groups and the carboxylic acid group in Demethyloleuropein significantly influence hydrogen bond interactions. The topological polar surface area must be less for better druggability qualities of Demethyloleuropein. Hence, lead optimization studies to reduce the number of oxygen atoms in the bridges could benefit, as they do not interact with the virus protease.

In summary, the research identified olive secoiridoids as inhibitors of SARS-CoV-2 entry and replication. The virtual search generated 932 conformers of 78 secoiridoids in the OliveNet™, unraveling Nüzhenide oleoside (NZO) and Demethyloleuropein (DMO) as the most active SARS-CoV-2 spike protein and M^pro^ inhibitors. The potential of olive secoiridoids to combat the hyperinflammatory responses in COVID-19 is an additional benefit. Molecular dynamics of the virus targets bound to the secoiridoids confirmed virus protein stability and secoiridoids interactions. Due to intricate molecular structures, secoiridoids may present problems in oral absorption. Given the longstanding use of olive fruits in Mediterranean and Arabian diets, the bioavailability of secoiridoids can be explicated but needs investigation. Also, the dose of secoiridoids that can combat the virus entry and replication, the amount of secoiridoids in different forms of olives, and recommended daily intake require further research. Besides, *in-vitro* and *in-vivo* studies to substantiate the virtual anti-SARS-CoV-2 activity of olive secoiridoids are imminent. The explored secoiridoids are novel leads for the design, discovery, and development of anti-SARS-CoV-2 therapeutics. For now, the known safety of olives as a functional food and the explored anti-SARS-COV-2 activity of olive secoiridoids afford a plausible intervention of SARS-CoV-2 infection and associated hyperinflammatory responses.

## Data Availability Statement

The datasets generated and analyzed, including videos of MD simulations of this study can be found online at repositories. The names of the repository/repositories6 and accession number(s) can be found in the article/Supplementary Material.

## Author Contributions

NT: Research concept, experimental design, precision docking, molecular dynamics, result analysis, original drafting of manuscript, and final approval. MA: Experimental design, preliminary docking analysis, result analysis, manuscript revision and editing, and final approval. HA: Experimental design, ADMETox screening, result analysis, manuscript revision and editing, and final approval. AN: Experimental design, preliminary docking analysis, ADMETox screening, result analysis, manuscript revision and editing, and final approval. RA: Experimental design, preliminary docking analysis, ADMETox screening, result analysis, original drafting of manuscript, and final approval. All authors contributed to the article and approved the submitted version.

## Conflict of Interest

The authors declare that the research was conducted in the absence of any commercial or financial relationships that could be construed as a potential conflict of interest.

## References

[B1] Acar-Tek N. Agagündüz D. (2020). Olive leaf (*Olea europaea* L. folium): potential effects on Glycemia and Lipidemia. Ann. Nutr. Metab. 76, 63–68. 10.1159/000505508 31901903

[B2] Alagna F. Mariotti R. Panara F. Caporali S. Urbani S. Veneziani G.. (2012). Olive phenolic compounds: metabolic and transcriptional profiling during fruit development. BMC Plant Biol. 12:162. 10.1186/1471-2229-12-162 22963618 PMC3480905

[B3] Ali M. U. Zulqarnain M. S. J. ur Rehman M. A. Rehman M. A. Alsamadany H. Shah Z. H. (2019). Metabolic and biochemical profiling of phenolic compound and their biosynthesis in oil crops. BJSTR 3, 13652–13661. 10.26717/BJSTR.2019.18.003166

[B4] Al-Ruqaie I. Al-Khalifah N. Shanavaskhan A. (2016). Morphological cladistic analysis of eight popular Olive (Olea europaea L.) cultivars grown in Saudi Arabia using Numerical Taxonomic System for personal computer to detect phyletic relationship and their proximate fruit composition. Saudi J. Biol. Sci. 23, 115–121. 10.1016/j.sjbs.2015.05.008 26858547 PMC4705253

[B5] BIOVIA (2015). Dassault Systèmes, Discovery Studio Visualizer, v16.1.0.15350. San Diego: Dassault Systèmes. https://discover.3ds.com/discovery-studio-visualizer-download (accessed June 20, 2016).

[B6] Bonvino N. P. Karagiannis T. C. Boskou D.-C. Zafiris E. Hung A. Ray N. B.. (2018). OliveNet™. Database (Oxford). 10.1093/database/bay016 29688352 PMC5808783

[B7] Brody M. Böhm I. Bauer R. (1993). Mechanism of action of methotrexate: experimental evidence that methotrexate blocks the binding of interleukin 1β to the interleukin 1 receptor on target cells. Eur. J. Clin. Chem. Clin. Biochem. 10:667. 10.1515/cclm.1993.31.10.667 8292668

[B8] Castejón M. L. Montoya T. Alarcón-de-la-Lastra C. Sánchez-Hidalgo M. (2020). Potential protective role exerted by Secoiridoids from *Olea europaea* L. in cancer, cardiovascular, neurodegenerative, aging-related, and immunoinflammatory diseases. Antioxidants 9:149. 10.3390/antiox9020149 32050687 PMC7070598

[B9] Celano M. Maggisano V. Lepore S. M. Russo D. Bulotta S. (2019). Secoiridoids of olive and derivatives as potential coadjuvant drugs in cancer: a critical analysis of experimental studies. Pharmacol. Res. 142, 77–86. 10.1016/j.phrs.2019.01.045 30772463

[B10] Chen Q. Niu X. Li N. (2017). Exploring the natural chemiome to target interleukin-6 receptor (IL-6R) cytokines: an atomic scale investigation for novel rheumatoid arthritis drug discovery. Braz. J. Pharm. Sci. 53:e17256 10.1590/s2175-97902017000317256

[B11] Conte P. Fadda C. Del Caro A. Urgeghe P. P. Piga A. (2020). Table olives: an overview on effects of processing on nutritional and sensory quality. Foods 9:514 10.3390/foods9040514 PMC7231206 32325961

[B12] da Silva Rocha S. F. Olanda C. G. Fokoue H. H. Sant'Anna C. M. (2019). Virtual screening techniques in drug discovery: review and recent applications. Curr. Top. Med. Chem. 19, 1751–1767. 10.2174/1568026619666190816101948 31418662

[B13] Daina A. Michielin O. Zoete V. (2017). SwissADME: a free web tool to evaluate pharmacokinetics, drug-likeness and medicinal chemistry friendliness of small molecules. Sci. Rep. 7:42717. 10.1038/srep.42717 28256516 PMC5335600

[B14] DE Shaw Research (2020). Download Desmond. https://www.deshawresearch.com/downloads/download_desmond.cgi/ (accessed July 10, 2020).

[B15] De Vivo M. Masetti M. Bottegoni G. Cavalli A. (2016). Role of molecular dynamics and related methods in drug discovery. J. Med. Chem. 59, 4035–4061. 10.1021/acs.jmedchem.5b01684 26807648

[B16] Fernández-Poyatos M. Ruiz-Medina A. Llorent-Martínez E. (2019). Phytochemical profile, mineral content, and antioxidant activity of *Olea europaea* L. cv. Cornezuelo table olives. Influence of in vitro simulated gastrointestinal digestion. Food Chem. 297:124933. 10.1016/j.foodchem.2019.05.207 31253274

[B17] Fraihat S. Gilbert-López B. Molina-Díaz A. Sabouni I. (2017). Physicochemical characterization of olive oil from Aljouf area of Saudi Arabia. Int. J. Chemtech. Res. 10, 1004–1010.

[B18] Gao Y. M. Xu G. Wang B. Liu B. C. (2020). Cytokine storm syndrome in coronavirus disease 2019: a narrative review. J. Int. Med. 10.1111/joim.13144 32696489 PMC7404514

[B19] Ghanbari R. Anwar F. Alkharfy K. M. Gilani A.-H. Saari N. (2012). Valuable nutrients and functional bioactives in different parts of olive (*Olea europaea* L.)—a review. Int. J. Mol. Sci. 13, 3291–3340. 10.3390/ijms13033291 22489153 PMC3317714

[B20] Gorzynik-Debicka M. Przychodzen P. Cappello F. Kuban-Jankowska A. Marino Gammazza A. Knap N.. (2018). Potential health benefits of olive oil and plant polyphenols. Int. J. Mol. Sci. 19:686. 10.3390/ijms19030686 29495598 PMC5877547

[B21] Gurung A. B. Ali M. A. Lee J. Farah M. A. Al-Anazi K. M. (2020). Unravelling lead antiviral phytochemicals for the inhibition of SARS-CoV-2 Mpro enzyme through *in silico* approach. Life Sci. 255:117831. 10.1016/j.lfs.2020.117831 32450166 PMC7243810

[B22] Hashmi M. A. Khan A. Hanif M. Farooq U. Perveen S. (2015). Traditional uses, phytochemistry, and pharmacology of *Olea europaea* (olive). *Evid. Based Complement. Altern. Med* 2015:541591 10.1155/2015/541591 PMC4352757 25802541

[B23] Hemida M. H. Ibrahim A. Al-Bahnsawy R. M. Al-Shathly M. R. (2014). Influence of environmental factors on olive oil production and quality in the Northern Region of kingdom of Saudi Arabia. J. Am. Sci. 10, 61–66. 10.7537/marsjas100114.13

[B24] Hospital A. Goñi J. R. Orozco M. Gelp,í J. L. (2015). Molecular dynamics simulations: advances and applications. Adv. Appl. Bioinform. Chem. 8, 37–47. 10.2147/AABC.S70333 26604800 PMC4655909

[B25] Kanakis P. Termentzi A. Michel T. Gikas E. Halabalaki M. Skaltsounis A.-L. (2013). From olive drupes to olive oil. An HPLC-orbitrap-based qualitative and quantitative exploration of olive key metabolites. Planta Med. 79, 1576–1587. 10.1055/s-0033-1350823 24072502

[B26] Kato K. Nakayoshi T. Fukuyoshi S. Kurimoto E. Oda A. (2017). Validation of molecular dynamics simulations for prediction of three-dimensional structures of small proteins. Molecules 22:1716. 10.3390/molecules22101716 29023395 PMC6151455

[B27] Khan M. Khan M. Khan Z. Ahamad T. Ansari W. (2020). Identification of dietary molecules as therapeutic agents to combat COVID-19 using molecular docking studies. Res. Squ. [Preprint] (accessed June 3, 2020). 10.21203/rs.3.rs-19560/v1

[B28] Koes D. R. Baumgartner M. P. Camacho C. J. (2013). Lessons learned in empirical scoring with smina from the CSAR 2011 benchmarking exercise. J. Chem. Inf. Model 53, 1893–1904. 10.1021/ci300604z 23379370 PMC3726561

[B29] Kumar Y. Singh H. Patel C. N. (2020). In silico prediction of potential inhibitors for the Main protease of SARS-CoV-2 using molecular docking and dynamics simulation based drug-repurposing. J. Infect. Public Health 13, 1210–1223. 10.1016/j.jiph.2020.06.016 32561274 PMC7297718

[B30] Lee-Huang S. Huang P. L. Zhang D. Lee J. W. Bao J. Sun Y. (2007). Discovery of small-molecule HIV-1 fusion and integrase inhibitors oleuropein and hydroxytyrosol: Part I. Integrase inhibition. Biochem. Biophys. Res. Commun. 354, 872–878. 10.1016/j.bbrc.2007.01.071 17275783 PMC2790717

[B31] Li H. Leung K.-S. Wong M.-H. (2012). “idock: a multithreaded virtual screening tool for flexible ligand docking,” in IEEE Symposium on Computational Intelligence in Bioinformatics and Computational Biology (CIBCB). (San Diego, CA: IEEE), 77–84. (INSPEC Accession Number: 12803637). 10.1109/CIBCB.2012.621721

[B32] Liu H. Lupala C. Li X. Lei J. Chen H. Qi J.. (2020a). Computational simulations reveal the binding dynamics between human ACE2 and the receptor binding domain of SARS-CoV-2 spike protein. bioRxiv [Preprint]. Available at: 10.1101/2020.03.24.005561 (accessed June 20, 2020).

[B33] Liu S. Zheng Q. Wang Z. (2020b). Potential covalent drugs targeting the main protease of the SARS-CoV-2 coronavirus. Bioinformatics 36, 3295–3298. 10.1093/bioinformatics/btaa224 32239142 PMC7184403

[B34] MacCord Research OLIVEAMINE® (2017). Original OliveNetTM Library: Quick Reference. https://mccordresearch.com.au/resources/original-olivenet-library-quick-reference/ (accessed July 10, 2020).

[B35] Maia E. H. B. Assis L. C. de Oliveira T. A. da Silva A. M. Taranto A. G. (2020). Structure-based virtual screening: from classical to artificial intelligence. Front Chem. 8:343. 10.3389/fchem.2020.00343 32411671 PMC7200080

[B36] Maiti S. Banerjee A. Nazmeen A. Kanwar M. Das S. (2020). Active-site molecular docking of Nigellidine to nucleocapsid/Nsp2/Nsp3/MPro of COVID-19 and to human IL1R and TNFR1/2 may stop viral-growth/cytokine-flood, and the drug source *Nigella sativa* (black cumin) seeds show potent antioxidant role in experimental rats. Research Square [Preprint]. 10.21203/rs.3.rs-26464/v132875925

[B37] Martinez-Gonzalez M. A. Martin-Calvo N. (2016). Mediterranean diet and life expectancy; beyond olive oil, fruits and vegetables. Curr. Opin. Clin. Nutr. Metab. Care 19, 401–407. 10.1097/MCO.0000000000000316 27552476 PMC5902736

[B38] Mazal H. Aviram H. Riven I. Haran G. (2018). Effect of ligand binding on a protein with a complex folding landscape. Phys. Chem. Chem. Phys. 20, 3054–3062. 10.1039/C7CP03327C 28721412

[B39] Medina Pradas E. Romero Barranco C. García García P. Brenes Balbuena M. (2019). Characterization of bioactive compounds in commercial olive leaf extracts, and olive leaves and their infusions. Food Funct. 10, 4716–4724. 10.1039/C9FO00698B 31304950

[B40] Morris G. M. Huey R. Lindstrom W. Sanner M. F. Belew R. K. Goodsell D. S.. (2009). AutoDock4 and AutoDockTools4: automated docking with selective receptor flexibility. J. Comput. Chem. 30, 2785–2791. 10.1002/jcc.21256 19399780 PMC2760638

[B41] Obied H. K. Bedgood D. R. Jr, Prenzler, P. D. Robards K. (2007). Chemical screening of olive biophenol extracts by hyphenated liquid chromatography. Anal. Chim. Acta 603, 176–189. 10.1016/j.aca.2007.09.044 17963838

[B42] Omar S. H. (2010). Oleuropein in olive and its pharmacological effects. Sci. Pharm. 78, 133–154. 10.3797/scipharm.0912-18 21179340 PMC3002804

[B43] Origin (2016). OriginLab Corporation. Northampton, MA: OriginLab Corporation.

[B44] Osiris (2020). Osiris Property Explorer https://www.organic-chemistry.org/prog/peo/ (accessed August 21, 2020).

[B45] Owen R. Haubner R. Mier W. Giacosa A. Hull W. Spiegelhalder B.. (2003). Isolation, structure elucidation and antioxidant potential of the major phenolic and flavonoid compounds in brined olive drupes. Food Chem. Toxicol. 41, 703–717. 10.1016/S0278-6915(03)00011-5 12659724

[B46] Pettersen E. F. Goddard T. D. Huang C. C. Couch G. S. Greenblatt D. M. Meng E. C.. (2004). UCSF Chimera—a visualization system for exploratory research and analysis. J. Comput. Chem. 25, 1605–1612. 10.1002/jcc.20084 15264254

[B47] Prajapat M. Sarma P. Shekhar N. Avti P. Sinha S. Kaur H.. (2020). Drug targets for corona virus: a systematic review. Indian J. Pharmacol. 52, 56–65. 10.4103/ijp.IJP_115_20 32201449 PMC7074424

[B48] Rahmani A. H. Albutti A. S. Aly S. M. (2014). Therapeutics role of olive fruits/oil in the prevention of diseases via modulation of antioxidant, anti-tumour and genetic activity. Int. J. Clin. Exp. Med. 7, 799–808. 24955148 PMC4057827

[B49] Rigacci S. Stefani M. (2016). Nutraceutical properties of olive oil polyphenols. An itinerary from cultured cells through animal models to humans. Int. J. Mol. Sci. 17:843. 10.3390/ijms17060843 27258251 PMC4926377

[B50] Saddala M. S. Huang H. (2019). Identification of novel inhibitors for TNFα, TNFR1 and TNFα-TNFR1 complex using pharmacophore-based approaches. J. Transl. Med. 17:215. 10.1186/s12967-019-1965-5 31266509 PMC6604280

[B51] Schrödinger Release (2020). LigPrep, Schrödinger. https://www.schrodinger.com/ligprep (accessed May 6, 2020).

[B52] Shang J. Ye G. Shi K. Wan Y. Luo C. Aihara H.. (2020). Structural basis of receptor recognition by SARS-CoV-2. Nature 581, 221–224. 10.1038/s41586-020-2179-y 32225175 PMC7328981

[B53] Shawky E. Nada A. A. Ibrahim R. S. (2020). Potential role of medicinal plants and their constituents in the mitigation of SARS-CoV-2: identifying related therapeutic targets using network pharmacology and molecular docking analyses. RSC Adv. 10, 27961–27983. 10.1039/D0RA05126H PMC9055652 35519104

[B54] Showande S. J. Fakeye T. O. Kajula M. Hokkanen J. Tolonen A. (2019). Potential inhibition of major human cytochrome P450 isoenzymes by selected tropical medicinal herbs—implication for herb–drug interactions. Food Sci. Nutr. 7, 44–55. 10.1002/fsn3.789 30680158 PMC6341161

[B55] Silva S. Gomes L. Leitao F. Coelho A. Boas L. V. (2006). Phenolic compounds and antioxidant activity of Olea europaea L. fruits and leaves. Food Sci. Technol. Int. 12, 385–395. 10.1177/1082013206070166

[B56] Sivakumar G. Uccella N. A. Gentile L. (2018). Probing downstream olive biophenol secoiridoids. Int. J. Mol. Sci. 19:2892. 10.3390/ijms19102892 30249049 PMC6212805

[B57] Srivalli K. M. R. Lakshmi P. K. (2012). Overview of P-glycoprotein inhibitors: a rational outlook. Braz. J. Pharm. Sci. 48, 353–367. 10.1590/S1984-82502012000300002

[B58] Tang Y. Liu J. Zhang D. Xu Z. Ji J. Wen C. (2020). Cytokine storm in COVID-19: the current evidence and treatment strategies. Front. Immunol. 11:1708. 10.3389/fimmu.2020.01708 32754163 PMC7365923

[B59] Trott O. Olson A. J. (2010). AutoDock Vina: improving the speed and accuracy of docking with a new scoring function, efficient optimization, and multithreading. J. Comput. Chem. 31, 455–461. 10.1002/jcc.21334 19499576 PMC3041641

[B60] Vilaplana-Pérez C. Auñón D. García-Flores L. A. Gil-Izquierdo A. (2014). Hydroxytyrosol and potential uses in cardiovascular diseases, cancer, and AIDS. Front. Nutr. 1:18. 10.3389/fnut.2014.00018 25988120 PMC4428486

[B61] Vissers M. Zock P. Katan M. (2004). Bioavailability and antioxidant effects of olive oil phenols in humans: a review. Eur. J. Clin. Nutr. 58, 955–965. 10.1038/sj.ejcn.1601917 15164117

[B62] Wahedi H. M. Ahmad S. Abbasi S. W. (2020). Stilbene-based natural compounds as promising drug candidates against COVID-19. J. Biomol. Struct. Dyn. 10.1080/07391102.2020.1762743 32345140

[B63] Wrapp D. Wang N. Corbett K. S. Goldsmith J. A. Hsieh C.-L. Abiona O.. (2020). Cryo-EM structure of the 2019-nCoV spike in the prefusion conformation. Science 367, 1260–1263. 10.1126/science.abb2507 32075877 PMC7164637

[B64] Yang H. Lou C. Sun L. Li J. Cai Y. Wang Z.. (2019). admetSAR 2.0: web-service for prediction and optimization of chemical ADMET properties. Bioinformatics 35, 1067–1069. 10.1093/bioinformatics/bty707 30165565

[B65] Zabetakis I. Lordan R. Norton C. Tsoupras A. (2020). COVID-19: The inflammation link and the role of nutrition in potential mitigation. Nutrients 12:1466. 10.3390/nu12051466 32438620 PMC7284818

[B66] Zhang L. Lin D. Sun X. Curth U. Drosten C. Sauerhering L.. (2020). Crystal structure of SARS-CoV-2 main protease provides a basis for design of improved α-ketoamide inhibitors. Science 368, 409–412. 10.1126/science.abb3405 32198291 PMC7164518

