# Supplementary material for: Molecular Docking and Molecular Dynamics Aided Virtual Search of OliveNet™ Directory for Secoiridoids to Combat SARS-CoV-2 Infection and Associated Hyperinflammatory Responses
Source: Front Mol Biosci. 2021 Jan 7;7:627767. doi: 10.3389/fmolb.2020.627767 (PMC7817976; doi:10.3389/fmolb.2020.627767)
Supplement: Supplementary file 2 [file Table_2.DOCX]

| Binding interactions of the three top-ranked secoiridoids and Chloroquine with SARS-CoV-2 S protein-ACE-2 receptor interface | | | | |
| --- | --- | --- | --- | --- |
| Name | H bonds | | Hydrophobic bonds | |
|  | Target residue | Distance (Å) | Target residue | Distance ( Å) |
| Nuzhenide oleoside | Lys26  Asn33  Asn90  His34  Gln96  Gln388  Gln409  Lys417  Tyr505 | 4.02 (attractive charge)  3.03  2.89  2.86, 2.92  2.13  2.33  2.01  2.61,2.76  2.85 | Lys26  His34  Tyr453  Pro389  Lys417 | 3.96 (ℼ-alkyl)  4.94 (ℼ-alkyl)  4.99 (ℼ-alkyl)  4.11, 4.28 (alkyl-alkyl)  3.93 (alkyl-alkyl) |
| Oleuropein dimer | Asn33  His34  Lys417  Gly504  Tyr505 | 2.86  2.34  4.32  2.49  2.20 | Lys417 | 4.32 (ℼ-cation)  3.78, 4.15 (ℼ-alkyl) |
| Dihydro oleuropein | Asn33  Arg393  Arg403  Glu406  Arg408  Gln409 | 2.81  2.86, 3.00  1.99, 2.62, 3.10  2.84  2.62  2.80 | His34  Lys417 | 4.89 (ℼ-alkyl)  4.02 (alkyl-alkyl)  4.92 (ℼ-alkyl) |
| Chloroquine | His34  Glu37  Glu406 | 2.23  4.84 (attractive charge)  2.45 | His34  Lys417 | 2.59 (ℼ-cation)  4.93 (ℼ-alkyl) |

**Table 4.** Detailed intermolecular interactions of the secoiridoids with S protein-ACE-2 interface
