# Supplementary material for: Molecular Docking and Molecular Dynamics Aided Virtual Search of OliveNet™ Directory for Secoiridoids to Combat SARS-CoV-2 Infection and Associated Hyperinflammatory Responses
Source: Front Mol Biosci. 2021 Jan 7;7:627767. doi: 10.3389/fmolb.2020.627767 (PMC7817976; doi:10.3389/fmolb.2020.627767)
Supplement: Supplementary file 3 [file Table_3.DOCX]

| Binding interactions of the three top-ranked secoiridoids and lopinavir with SARS-CoV-2 Mpro. | | | | |
| --- | --- | --- | --- | --- |
| Name | H bonds | | Hydrophobic bonds | |
|  | Target residue | Distance (Å) | Target residue | Distance (Å) |
| Demethyl oleuropein | Thr26  Asn142  Gly143  Ser144  Cys145  His163  Arg188  Thr190 | 1.93, 2.07  2.61  2.11  2.65  2.82  2.14  2.24  2.45 | His41  Met165 | 4.31 (ℼ-alkyl)  4.98 (ℼ-S interaction) |
| Neo-nuzhenide | Phe140  Gly143  Ser144  Cys145  Thr190  Glu166 | 2.69  2.28  2.45  2.38  2.55  2.13, 2.93 | Leu27  His41  Cys145  Met165 | 3.82 (alkyl-alkyl)  3.67, 4.99, 5.00 (ℼ-alkyl)  4.60, 4.94(alkyl-alkyl)  4.97 (ℼ-S interaction) |
| Nuzhenide | Thr24  Phe140  Gly143  Ser144  Cys145  Glu166  Thr190 | 1.94, 2.55  2.46  2.18  2.43, 2.96  2.34  2.91  1.89 | Leu27  His41  Met165 | 4.46 (alkyl-alkyl)  3.72 (ℼ-alkyl)  4.98 (ℼ-alkyl) |
| Lopinavir | Asn142  Ser144  Cys145  Glu166  Gln189  Thr190 | 3.08  2.20  2.35  2.16  2.07  2.35 | His41  Met49  Met165  Glu166 | 4.93 (ℼ- ℼ stacked)  4.56 (ℼ-alkyl)  4.68 (ℼ-S interaction)  4.64 ( ℼ-anion) |

**Table 5.** Detailed intermolecular interactions of the secoiridoids with SARS-CoV-2 Mpro.
