# Supplementary material for: Molecular Docking and Molecular Dynamics Aided Virtual Search of OliveNet™ Directory for Secoiridoids to Combat SARS-CoV-2 Infection and Associated Hyperinflammatory Responses
Source: Front Mol Biosci. 2021 Jan 7;7:627767. doi: 10.3389/fmolb.2020.627767 (PMC7817976; doi:10.3389/fmolb.2020.627767)
Supplement: Supplementary file 4 [file Table_4.DOCX]

| **Drug Target** | **General name** | **AutodockVina** | **Smina** | **Idock** |
| --- | --- | --- | --- | --- |
| IL1R_1ITB | METHOTREXATE | -7.9 | -7.8 | -7.68 |
|  | Nuzhenideoleoside | -6.6 | -7.8 | -7.83 |
|  | Nuzhenide | -7.4 | -7.4 | -7.66 |
|  | Neo-nuzhenide | -7.5 | -6.9 | -7.36 |
|  | Demethyloleuropein | -6.9 | -7.2 | -7.68 |
|  | Oleuropein dimer | -7.1 | -6.7 | -7.22 |
|  | Dihydrooleuropein | -6.3 | -6.2 | -6.58 |
| IL6-1N26 | CHEMIOMECID5329098 | -6.5 | -7.4 | -7.0 |
|  | Neo-nuzhenide | -6.6 | -7.7 | -7.27 |
|  | Nuzhenide | -6.2 | -7.5 | -6.67 |
|  | Demethyloleuropein | -7.0 | -6.9 | -6.46 |
|  | Nuzhenideoleoside | -6.3 | -6.7 | -6.01 |
|  | Oleuropein dimer | -6.0 | -5.8 | -5.08 |
|  | Dihydrooleuropein | -5.6 | -6.6 | -5.33 |
| TNFR1-1NCF | PHYSCION-8-GLUCOSIDE | -7.9 | -8.7 | -8.0 |
|  | Nuzhenideoleoside | -8.0 | -8.9 | -7.87 |
|  | Oleuropein dimer | -7.1 | -7.2 | -7.18 |
|  | Neo-nuzhenide | -7.0 | -7.6 | -6.71 |
|  | Demethyloleuropein | -7.3 | -7.1 | -6.20 |
|  | Nuzhenide | -6.8 | -7.3 | -6.34 |
|  | Dihydrooleuropein | -5.6 | -6.6 | -5.33 |

**Table S6**: Binding energies and binding interactions of the top-ranked olive secoiridoids with the inflammatory cytokine receptors

| **Binding interactions of top-ranked secoiridoids with inflammatory cytokine receptors** | | | | |
| --- | --- | --- | --- | --- |
| Name | H bonds | | Hydrophobic bonds | |
|  | Target residue | Distance (Å) | Target residue | Distance ( Å) |
| IL1R_1ITB |  |  |  |  |
| METHOTREXATE | Leu237  Ala241  Ile250  Glu259  Thr277 | 1.86  1.89  2.20  2.19,2.49  2.07 | Tyr261 | 3.72, 4.91, 5.01 |
| Nuzhenide oleoside | Asn216  Lys244  Ile250  Thr294 | 2.44  2.67  2.14,2.15  2.15 | Tyr261  Ile303  Ala305 | 4.86  4.64, 4.96  4.65 |
| Nuzhenide | Met219  Asp239  Glu259  Tyr261  Arg287  Pro292 | 2.38  2.17  2.72  2.58  2.38  1.86, 2.29 | Pro214  Tyr261  Pro292 | 4.98  4.70  4.27 |
| Neo-nuzhenide | Asp239  Ile250  Glu259 | 2.22  2.49  2.13 | Tyr242  Tyr261  Ile240 | 3.61  3.96, 4.48  5.01 |
| Demethyloleuropein | Tyr261  Ile250  Ser248 | 2.39  1.87  2.72 | Ile250 | 3.63 |
| Oleuropein dimer | Glu252  Glu259 | 1.84  2.04, 2.35, 2.47 | Ile240  Val249  Tyr261 | 4.96  4.56  4.13 |
| Dihydrooleuropein | Leu237  Ala241  Glu259  Tyr261 | 2.31  2.54  2.05  2.45 | Ile240  Tyr261 | 4.81  4.01, 4.70, 4.98 |
| IL6-1N26 |  |  |  |  |
| CHEMIOMECID5329098 | Ser122  Leu123  Thr124 | 2.61  2.38  2.63 | Pro46  Leu90 | 4.87  4.16, 4.57 |
| Neo-nuzhenide | Glu34  Ala47  Arg54  Agr65  Ser72 | 2.78  1.97  2.72  2.57  2.09, 2.53 | Lys45  Ala47 | 4.05 (ℼ-cation), 4.44  3.75, 5.04 |
| Nuzhenide | His70  Leu90 | 1.81, 2.64  2.72 | Lys45  Leu90  Leu123 | 5.01  4.99  5.03 |
| Demethyloleuropein | Ser122  Leu123  Thr124 | 3.00  2.26  2.11, 3.05 | Pro46  Leu90  Val15 | 4.06  4.40, 4.97  5.01 |
| Nuzhenideoleoside | Trp55  His51  His70  His88  Gln68  Asp71 | 2.90  2.57  2.36  2.04  2.73  2.33 | His70  His88 | 4.14 (ℼ-cation), 4.15  4.67 (ℼ-cation) |
| Oleuropein dimer | Cys28  Ser72  Ala47  Arg44  Thr124 | 2.82  2.20, 2.77, 2.84  2.51  2.50, 2.67  2.46 | Lys45  Pro46 | 3.87  4.78, 4.12 |
| Dihydrooleuropein | Met58  His70  Asp71  Arg65 | 1.80, 2.73  2.09  2.02, 2.41  2.65 | Trp55  Leu63  Leu62 | 3.85, 4.15  4.34  5.04 |
| TNFR1-1NCF |  |  |  |  |
| Physcion-8-glucoside | Ser74  Arg77  Arg104  Lys132 | 2.82  2.70  2.41  2.18 | Arg77  Cys96  Cys96 | 4.91  4.73  4.48 (ℼ-sulfur) |
| Nuzhenideoleoside | Arg99  Asn101  Gln102  His105  His126  Gln130  Gln133 | 2.52  2.03  2.16  2.99, 5.04 (attractive charge)  2.07  2.50  2.90 | Leu127  Val136 | 4.37  4.74 |
| Oleuropein dimer | Tyr103  His105  Gln130  Lys132  Gln133  Asn134  Thr135  Glu147 | 2.32  2.81  2.29  2.11  2.88  2.77  1.90  2.99 | Tyr106  His126  Leu127  Lys132 | 4.19  3.71(ℼ-cation)  4.22  4.66 |
| Neo-nuzhenide | Gln130  Lys132  Thr135 | 3.90  2.99  2.39 | Leu127  Lys132 | 4.99  4.25 |
| Demethyloleuropein | Asn134  His105 | 2.28  5.01 | Lys132  Leu127 | 4.54  4.99 |
| Nuzhenide | Lys132  Asn134  His105 | 2.46  2.42  2.48 | His105  Leu127  Val136 | 4.32  4.31  4.83 |
| Dihydrooleuropein | His105  Asn134  Gln130 | 2.34  1.97  2.62 | His105  Leu127  Lys132 | 4.50  5.05  4.33 |
