# Supplementary material for: Molecular Docking and Molecular Dynamics Aided Virtual Search of OliveNet™ Directory for Secoiridoids to Combat SARS-CoV-2 Infection and Associated Hyperinflammatory Responses
Source: Front Mol Biosci. 2021 Jan 7;7:627767. doi: 10.3389/fmolb.2020.627767 (PMC7817976; doi:10.3389/fmolb.2020.627767)
Supplement: Supplementary file 5 [file Table_5.DOCX]

**Table S7:** Grid parameters for docking of secoiridoids to cytokine receptors.

| **Drug Target** | **Center_X** | **Center_Y** | **Center_Z** | **Size_X** | **Size_Y** | **Size_Z** |
| --- | --- | --- | --- | --- | --- | --- |
| 1ITB-IL1R | 43.430 | 7.030 | 16.655 | 90.900 | 58.730 | 27.450 |
| 1N26-IL6R | 29.160 | 42.00 | 74.620 | 54.350 | 39.320 | 90.690 |
| 1NCF-TNFR1 | 20.100 | 12.80 | 39.707 | 41.170 | 39.830 | 70.900 |
