# Supplementary material for: Molecular Docking and Molecular Dynamics Aided Virtual Search of OliveNet™ Directory for Secoiridoids to Combat SARS-CoV-2 Infection and Associated Hyperinflammatory Responses
Source: Front Mol Biosci. 2021 Jan 7;7:627767. doi: 10.3389/fmolb.2020.627767 (PMC7817976; doi:10.3389/fmolb.2020.627767)
Supplement: Supplementary file 6 [file Table_6.docx]

| **Following is the list of Tables, Figures, and Videos generated and analysed for the published study in Frontiers in Molecular Biosciences.**  **Molecular Docking and Molecular Dynamics Aided Virtual Search of OliveNetTM Directory for Secoiridoids to Combat SARS-CoV-2 Infection and Associated Hyperinflammatory Responses** | | |
| --- | --- | --- |
| Table S1 | List of 78 olive secoiridoids and their 2D structures |  |
| Table S2 | List of 932 conformers of 78 secoiridoids generated from virtual search |  |
| Table S3 | Results of virtual search by Autodock Vina |  |
| Table S4 | Detailed intermolecular interactions of the secoiridoids with S protein-ACE-2 interface |  |
| Table S5 | Detailed intermolecular interactions of the secoiridoids with Mpro |  |
| Table S6 | Molecular docking of secoiridoids to cytokine receptors |  |
| Table S7 | Legends of tables, figures, and videos |  |
| Figure S1 | Grid docking of secoiridoids to Mpro |  |
| Figure S2 | Gid docking of secoiridoids to S protein |  |
| Figure S3 | Chloroquine interactions with S protein-ACE-2 interface |  |
| Figure S4 | Lopinavir binding interactions with Mpro |  |
| Figure S5 | Drug-score calculation by Osiris for Demethyloleuropein (DMO) |  |
| Figure S6 | Drug-score of Nuzhenideoleoside (NZO) |  |
| Figure S7 | Drug-score of Lopinavir |  |
| Figure S8 | Interactions of methotrexate with IL1R |  |
| Figure S9 | Interactions of Chemiome CID 5329098 with IL6R |  |
| Figure S10 | Interactions of Physcion-8-glucoside with TNFR1 |  |
| Figure S11 | RMSF of apo form of S-protein-ACE-2 complex |  |
| Figure S12 | RMSF of NZO when bound to S-protein-ACE-2 interface |  |
| Figure S13 | RMSF of apo form of Mpro |  |
| Figure S14 | RMSF of DMO bound to Mpro |  |
| Video 1 | MD simulation of NZO- S-protein-ACE-2 complex |  |
| Video 2 | MD simulation of DMO-Mpro complex |  |
