# Supplementary material for: Molecular Docking and Molecular Dynamics Aided Virtual Search of OliveNet™ Directory for Secoiridoids to Combat SARS-CoV-2 Infection and Associated Hyperinflammatory Responses
Source: Front Mol Biosci. 2021 Jan 7;7:627767. doi: 10.3389/fmolb.2020.627767 (PMC7817976; doi:10.3389/fmolb.2020.627767)
Supplement: Supplementary file 10 [file Data_Sheet_2.PDF]

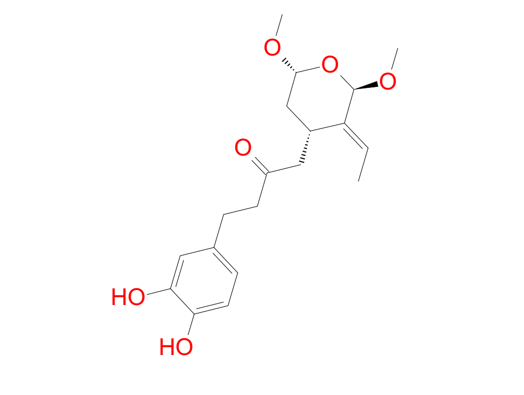

title P_3,4-DHPEA-DETA.c

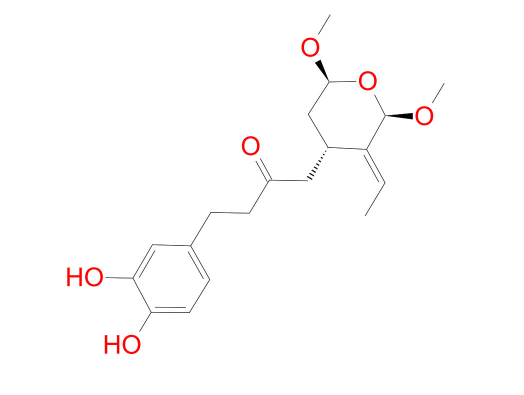

title P_3,4-DHPEA-DETA.c

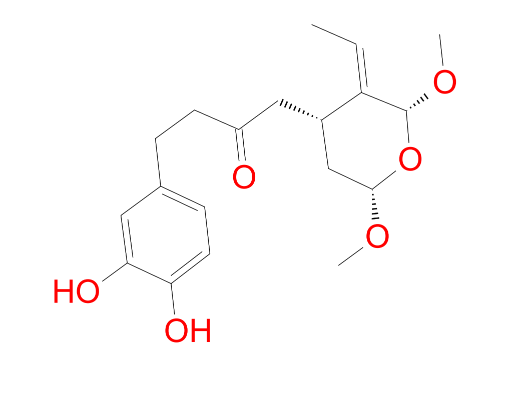

title P_3,4-DHPEA-DETA.c

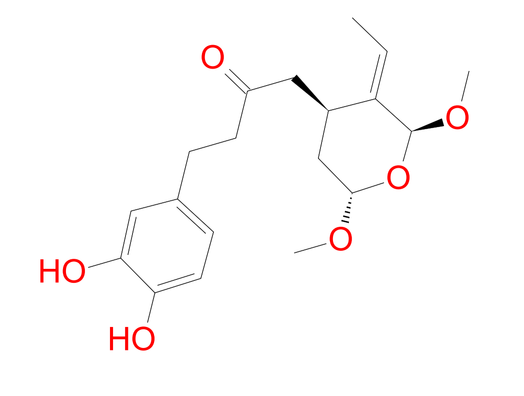

title P_3,4-DHPEA-DETA.c

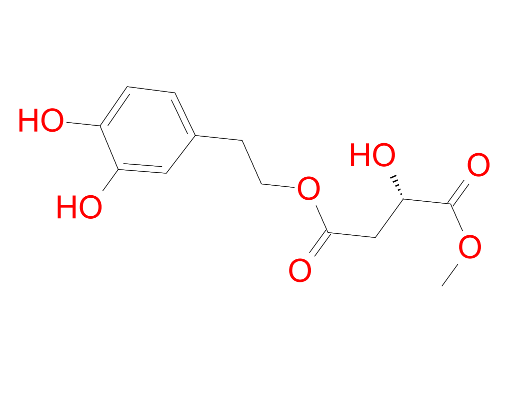

title P6_Methyl malate-hy

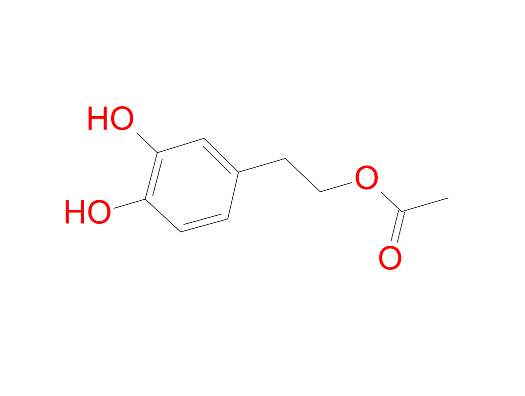

title P7_Hydroxytyrosol a

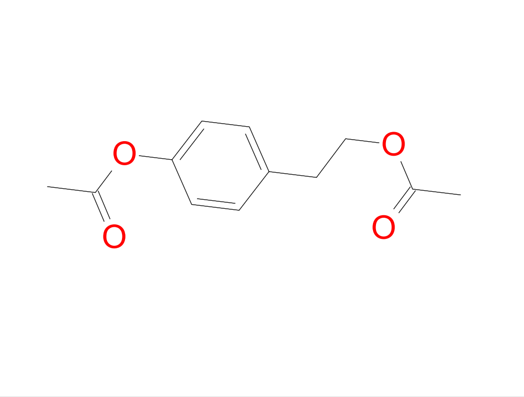

title P8_Tyrosol acetate.c

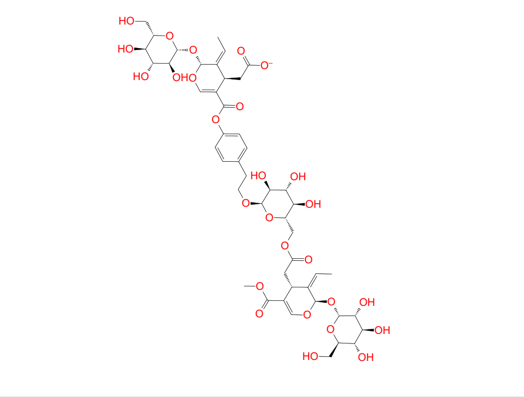

title P117_Nuzhenide ole

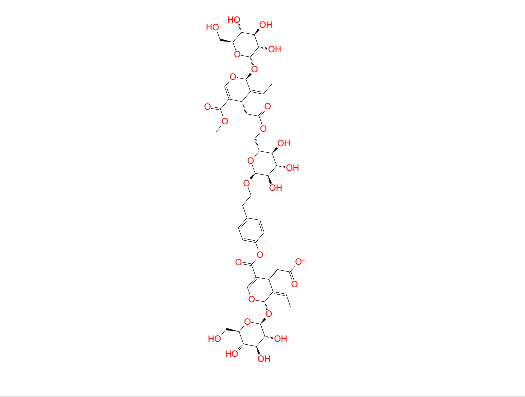

title P117_Nuzhenide ole

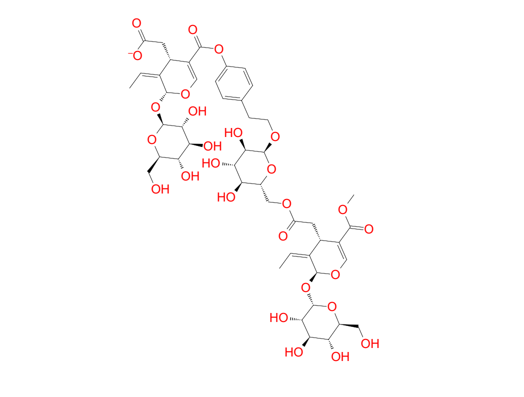

title P117_Nuzhenide ole

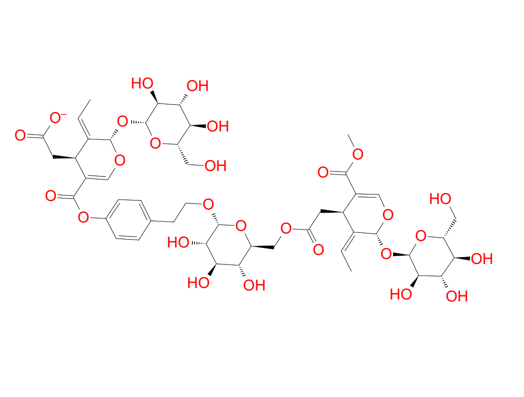

title P117_Nuzhenide ole

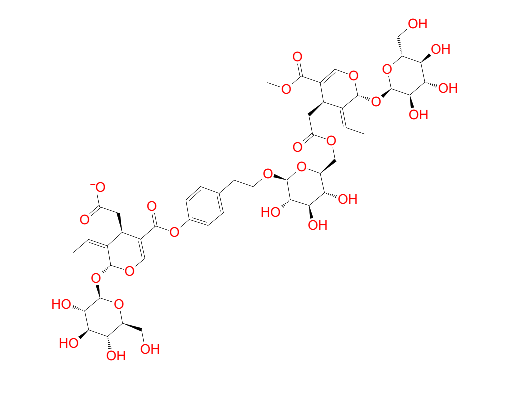

title P117_Nuzhenide ole

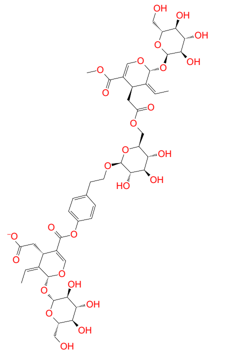

title P117_Nuzhenide ole

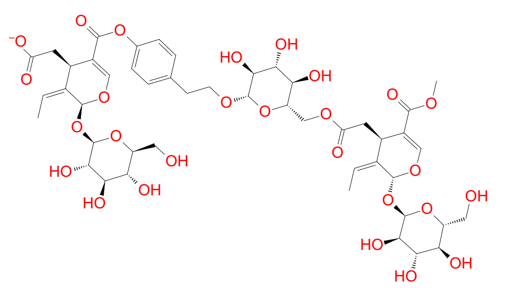

title P117_Nuzhenide ole

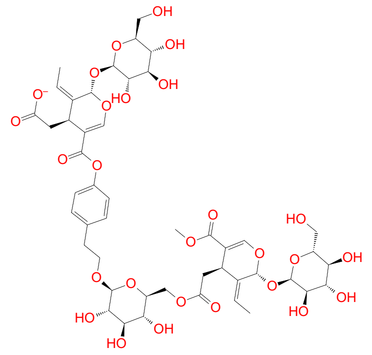

title P117_Nuzhenide ole

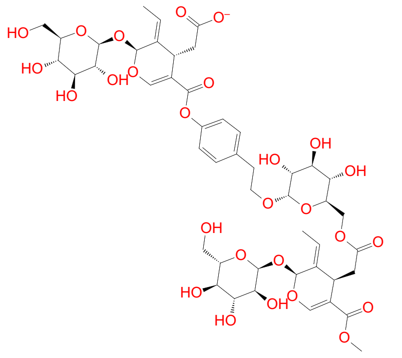

title P117_Nuzhenide ole

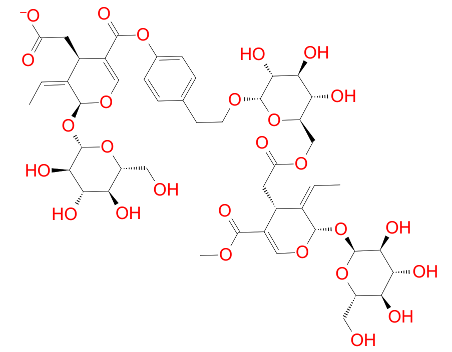

title P117_Nuzhenide ole

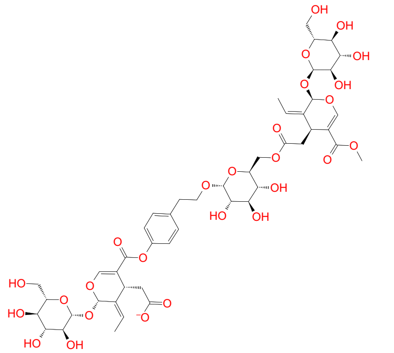

title P117_Nuzhenide ole

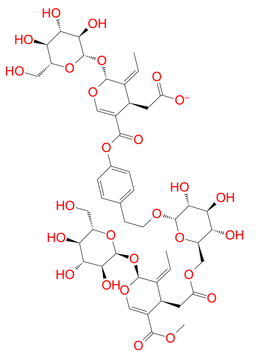

title P117_Nuzhenide ole

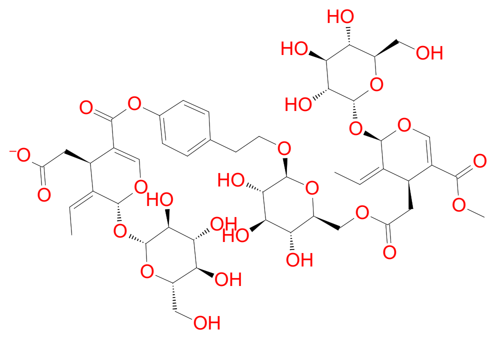

title P117_Nuzhenide ole

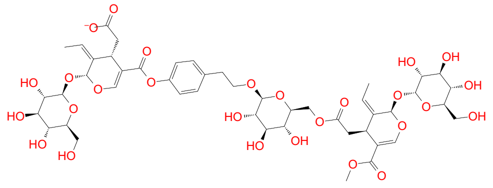

title P117_Nuzhenide ole

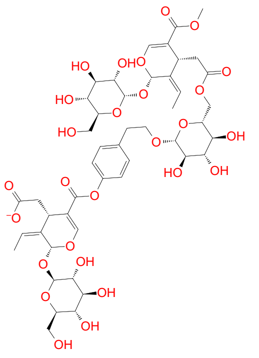

title P117_Nuzhenide ole

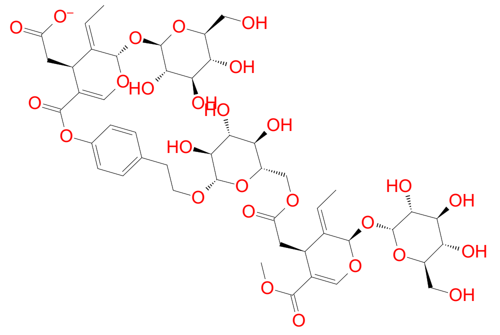

title P117_Nuzhenide ole

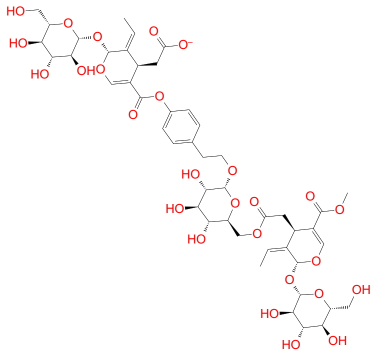

title P117_Nuzhenide ole

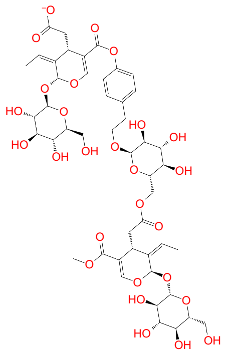

title P117_Nuzhenide ole

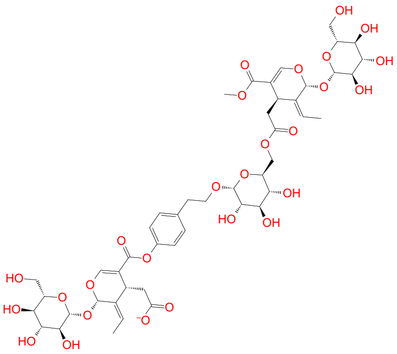

title P117_Nuzhenide ole

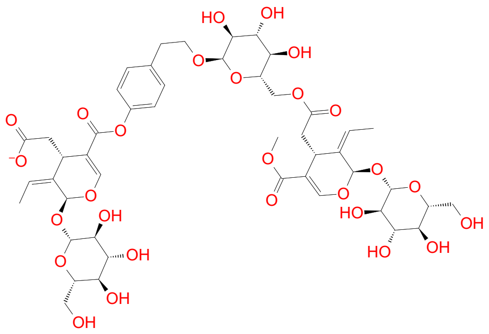

title P117_Nuzhenide ole

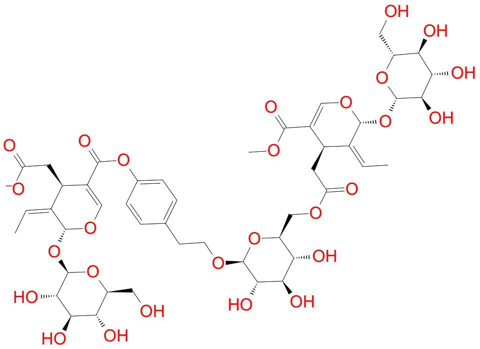

title P117_Nuzhenide ole

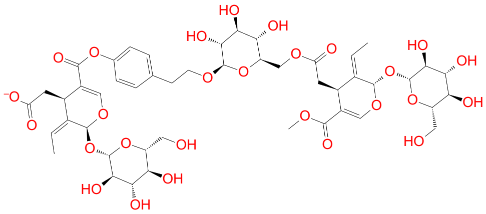

title P117_Nuzhenide ole

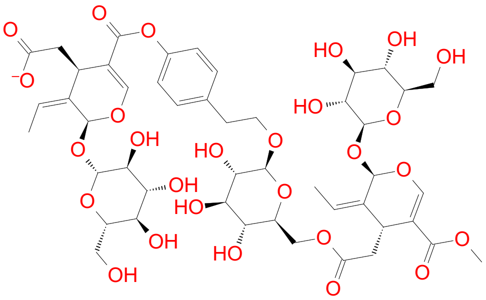

title P117_Nuzhenide ole

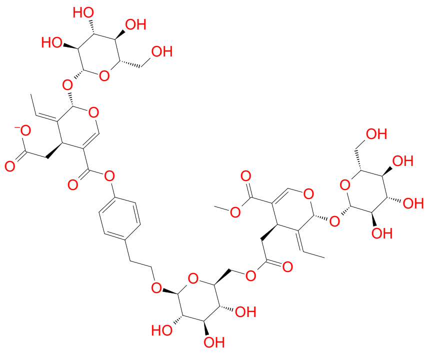

title P117_Nuzhenide ole

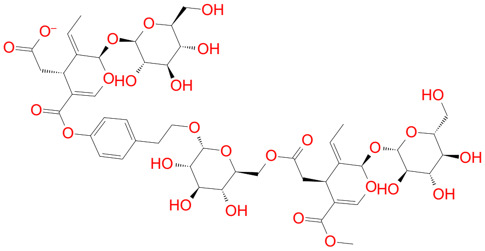

title P117_Nuzhenide ole

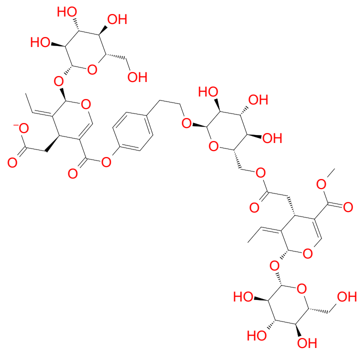

title P117_Nuzhenide ole

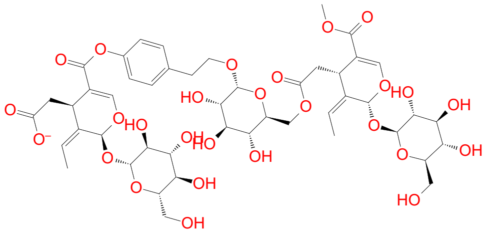

title P117_Nuzhenide ole

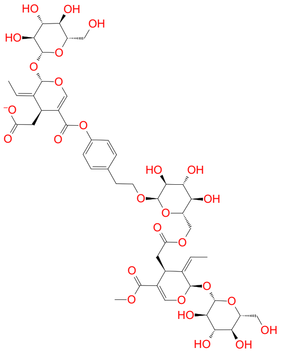

title P117_Nuzhenide ole

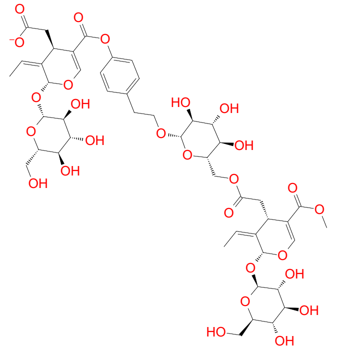

title P117_Nuzhenide ole

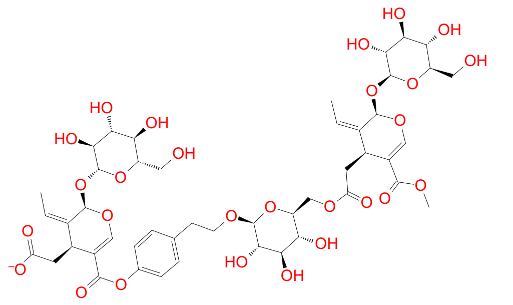

title P117_Nuzhenide ole

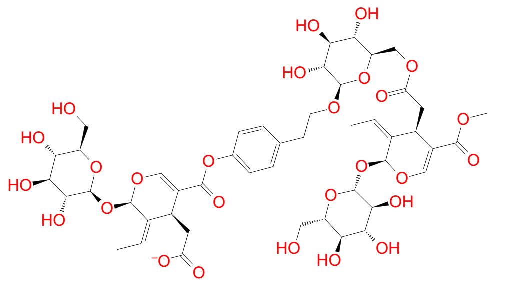

title P117_Nuzhenide ole

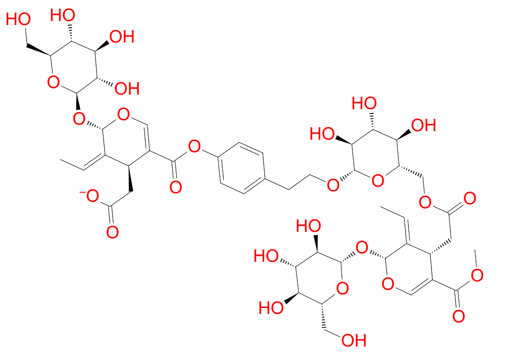

title P117_Nuzhenide ole

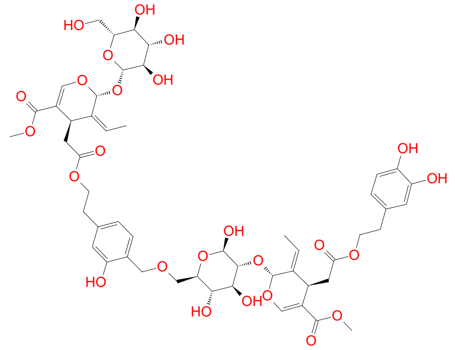

title P121_Oleuropein dim

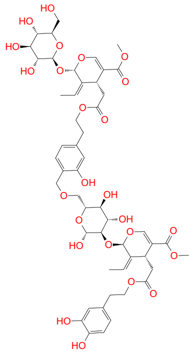

title P121_Oleuropein dim

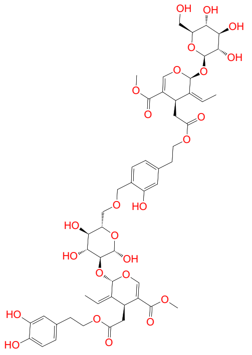

title P121_Oleuropein dim

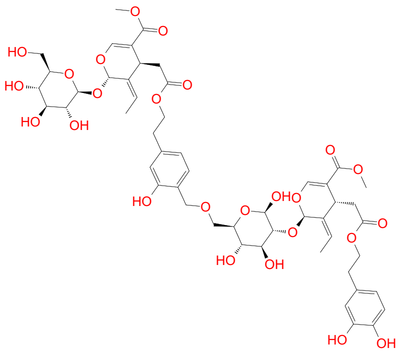

title P121_Oleuropein dim

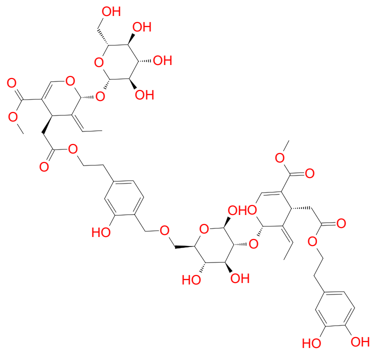

title P121_Oleuropein dim

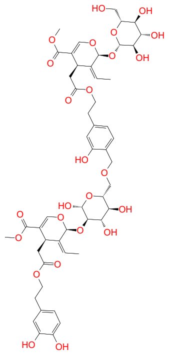

title P121_Oleuropein dim

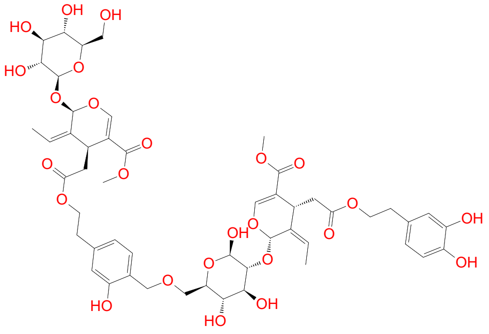

title P121_Oleuropein dim

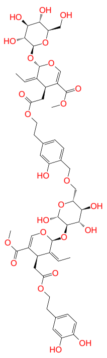

title P121_Oleuropein dim

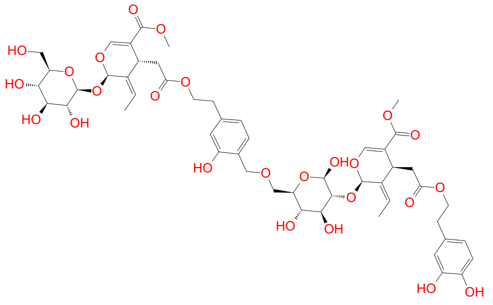

title P121_Oleuropein dim

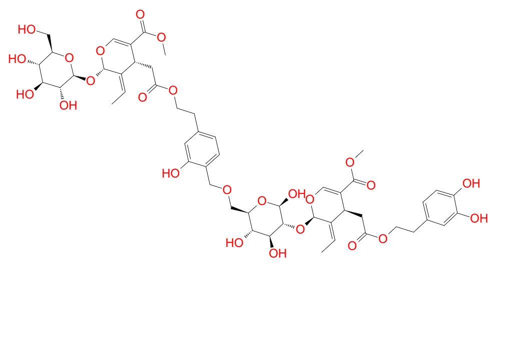

title P121_Oleuropein din

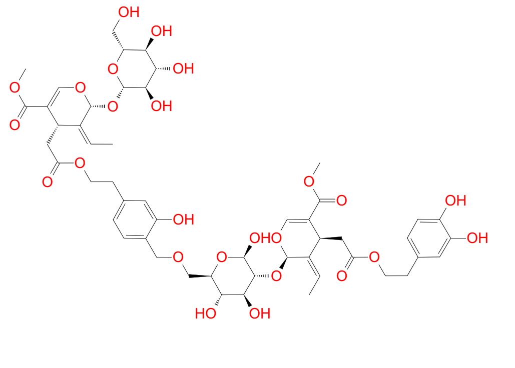

title P121_Oleuropein din

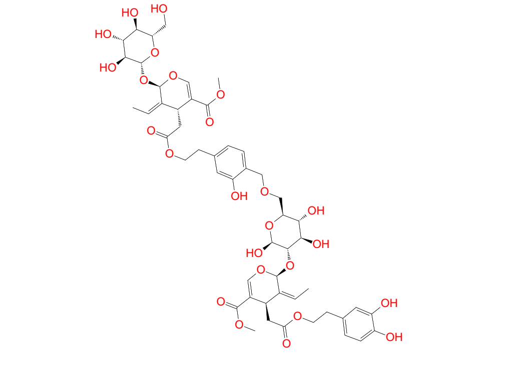

title P121_Oleuropein din

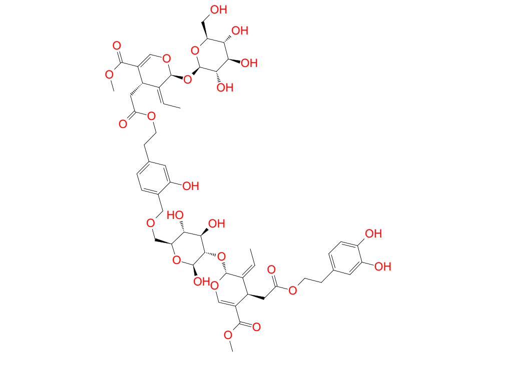

title P121_Oleuropein din

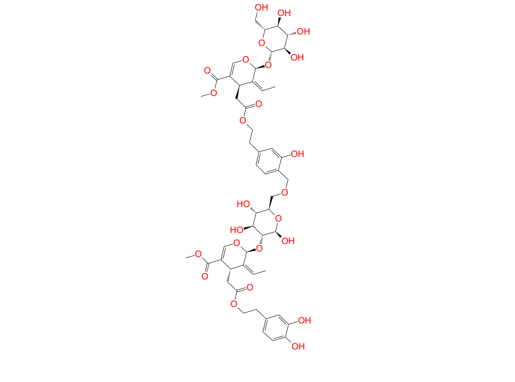

title P121_Oleuropein din

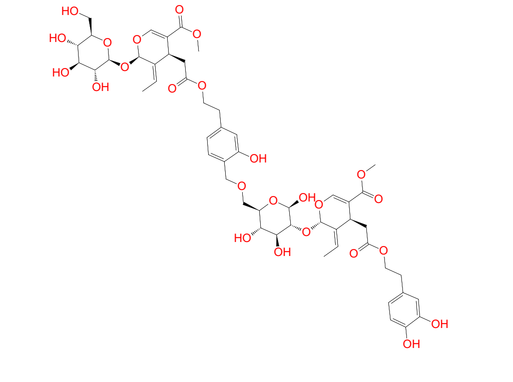

title P121_Oleuropein din

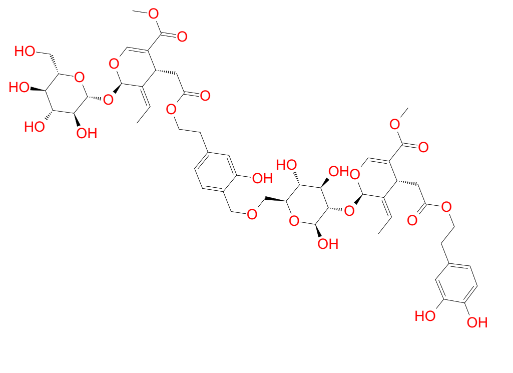

title P121_Oleuropein din

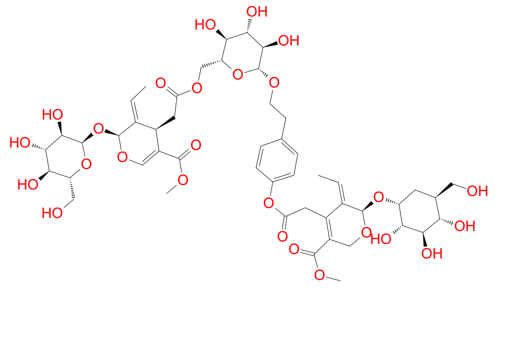

title P122_Nuzhenide 11-

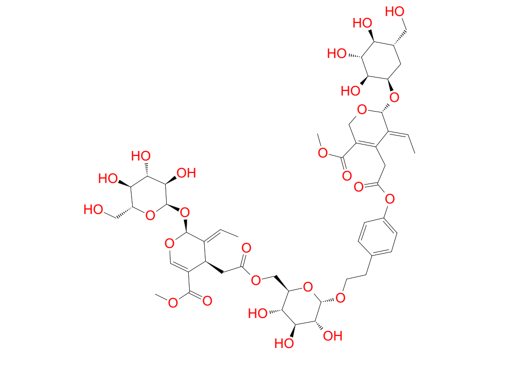

title P122_Nuzhenide 11-

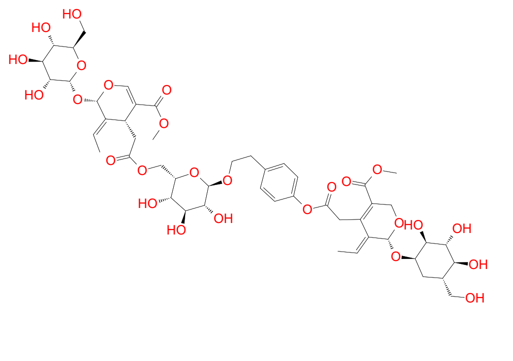

title P122_Nuzhenide 11-

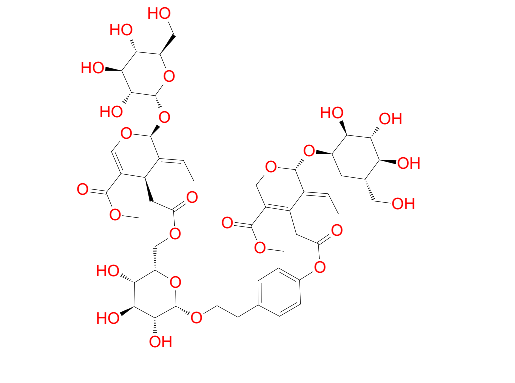

title P122_Nuzhenide 11-

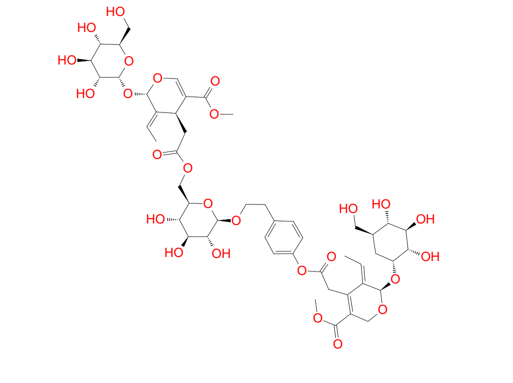

title P122_Nuzhenide 11-

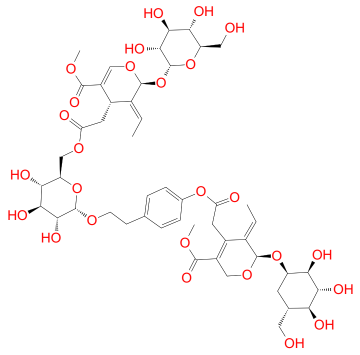

title P122_Nuzhenide 11-

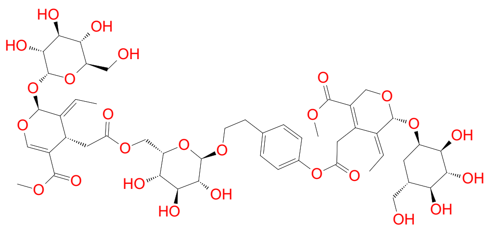

title P122_Nuzhenide 11-

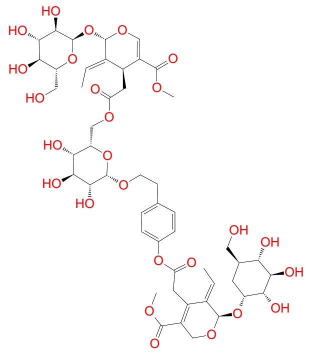

title P122_Nuzhenide 11-

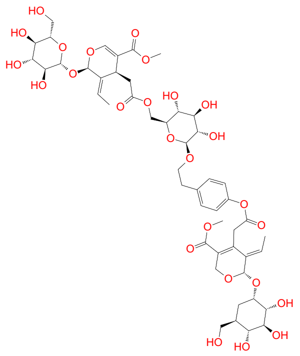

title P122_Nuzhenide 11-

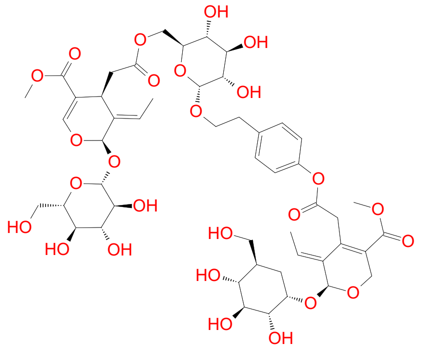

title P122_Nuzhenide 11-

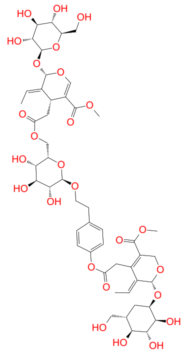

title P122_Nuzhenide 11-

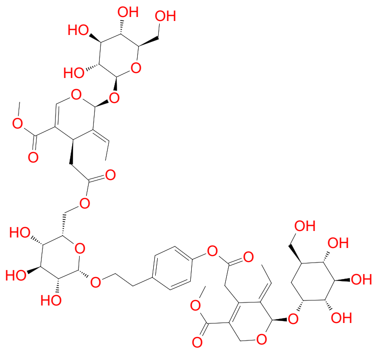

title P122_Nuzhenide 11-

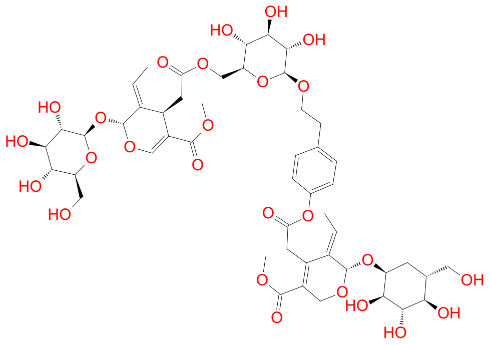

title P122_Nuzhenide 11-

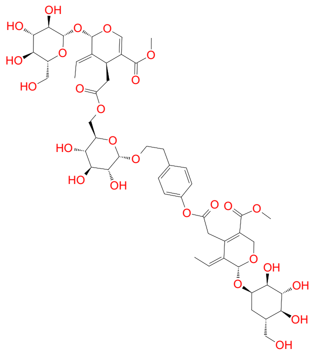

title P122_Nuzhenide 11-

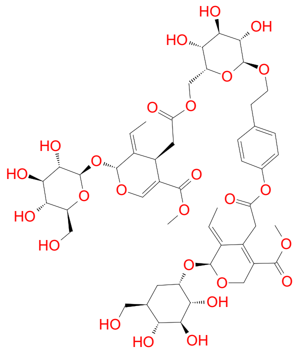

title P122_Nuzhenide 11-

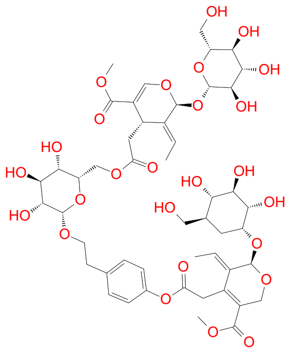

title P122_Nuzhenide 11-

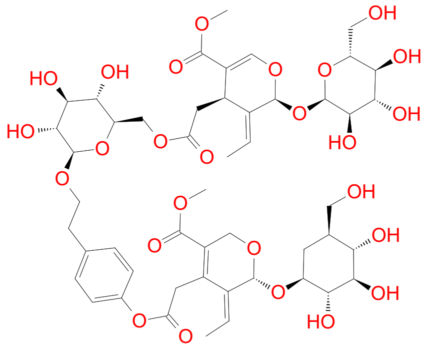

title P122_Nuzhenide 11-

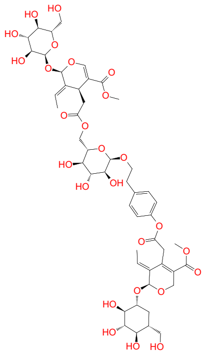

title P122_Nuzhenide 11-

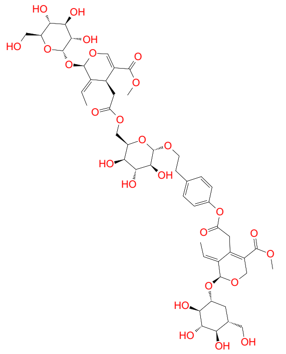

title P122_Nuzhenide 11-

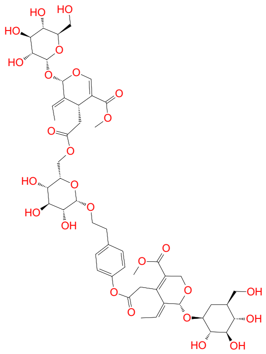

title P122_Nuzhenide 11-

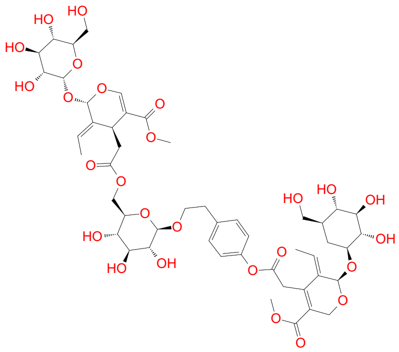

title P122_Nuzhenide 11-

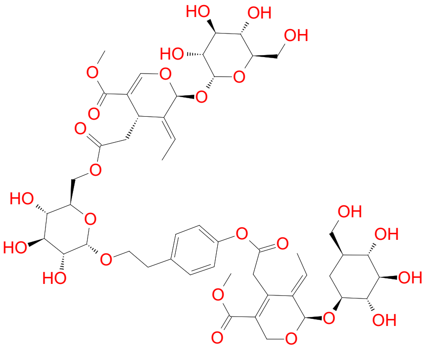

title P122_Nuzhenide 11-

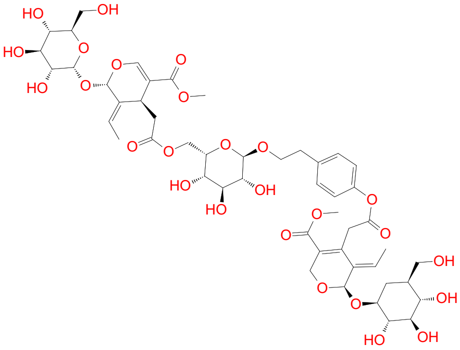

title P122_Nuzhenide 11-

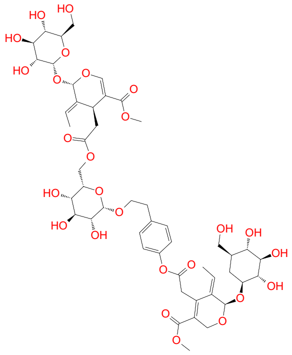

title P122_Nuzhenide 11-

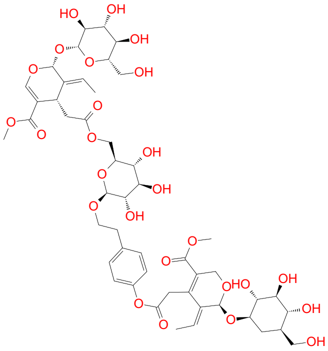

title P122_Nuzhenide 11-

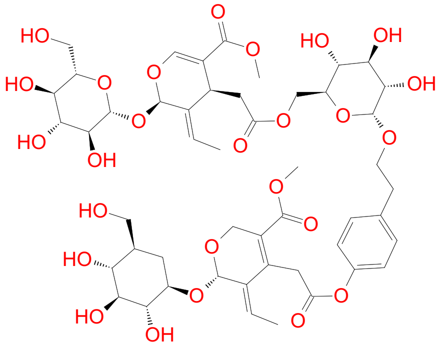

title P122_Nuzhenide 11-

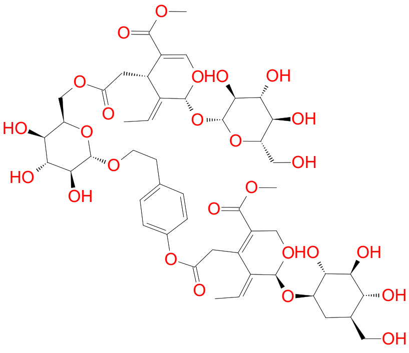

title P122_Nuzhenide 11-

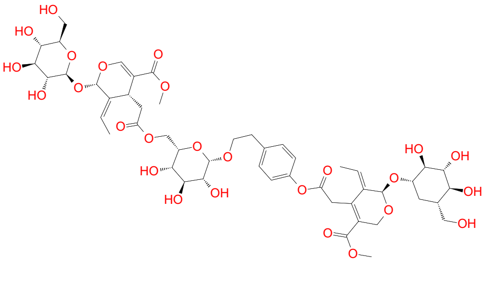

title P122_Nuzhenide 11-

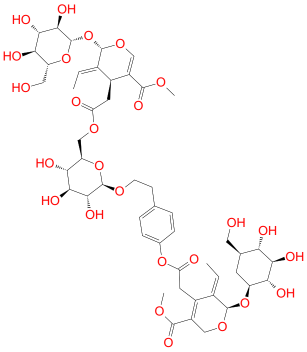

title P122_Nuzhenide 11-

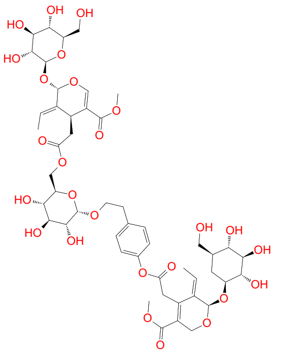

title P122_Nuzhenide 11-

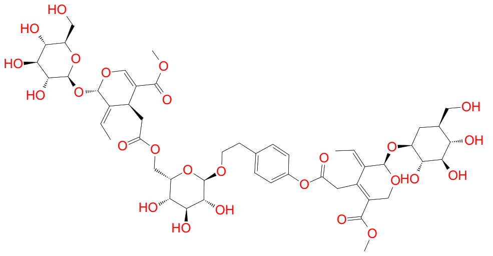

title P122_Nuzhenide 11-

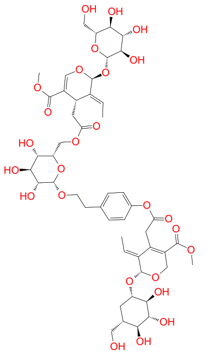

title P122_Nuzhenide 11-

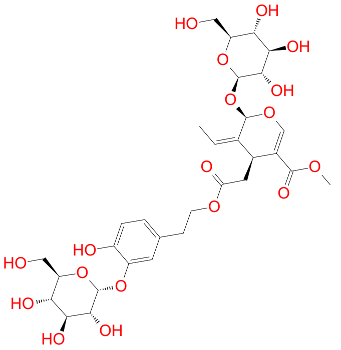

title P123_Oleuropein dig

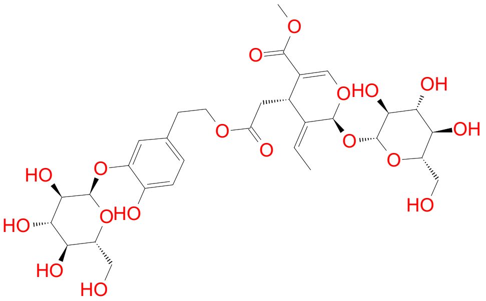

title P123_Oleuropein dig

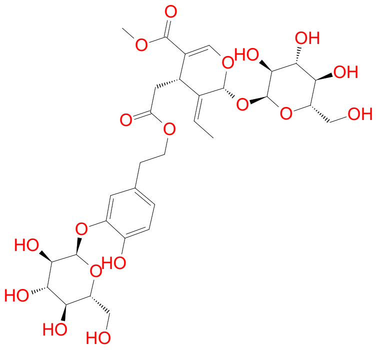

title P123_Oleuropein dig

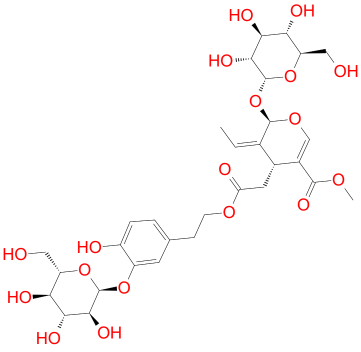

title P123_Oleuropein dig

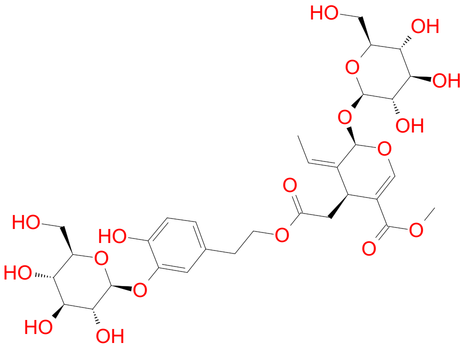

title P123_Oleuropein dig

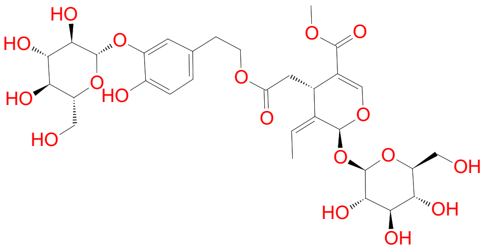

title P123_Oleuropein dig

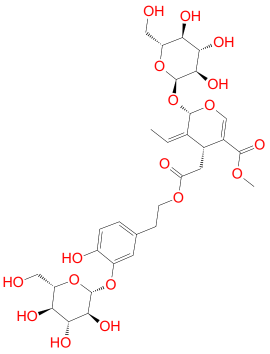

title P123_Oleuropein dig

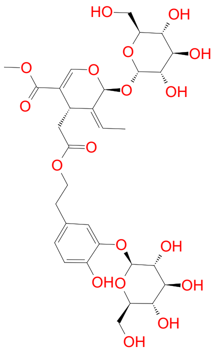

title P123_Oleuropein dig

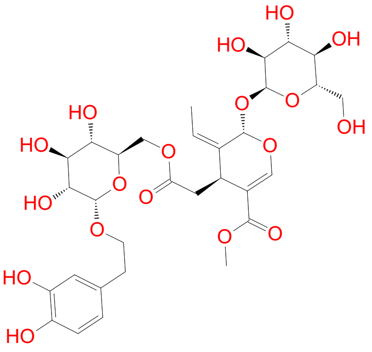

title P124_Neo-nuzhenide

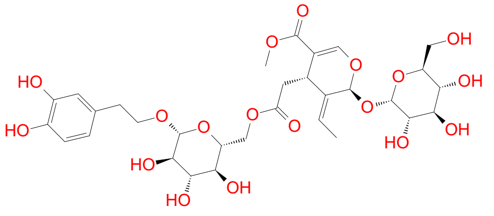

title P124_Neo-nuzhenide

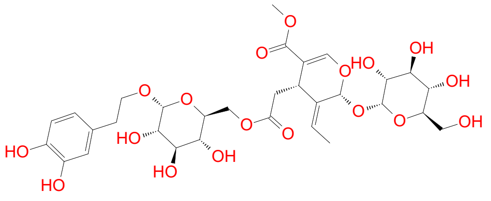

title P124_Neo-nuzhenide

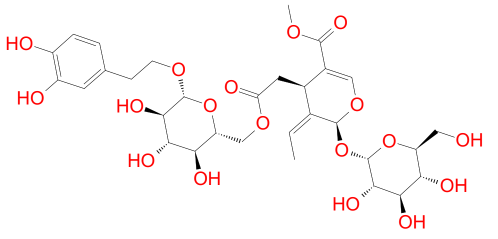

title P124_Neo-nuzhenide

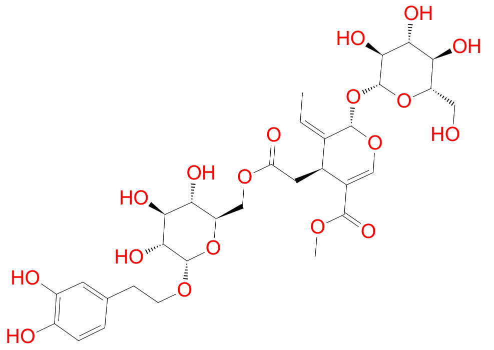

title P124_Neo-nuzhenide

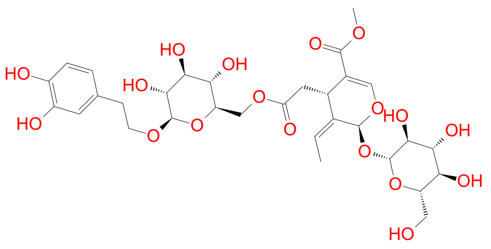

title P124_Neo-nuzhenide

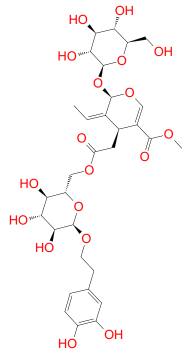

title P124_Neo-nuzhenide

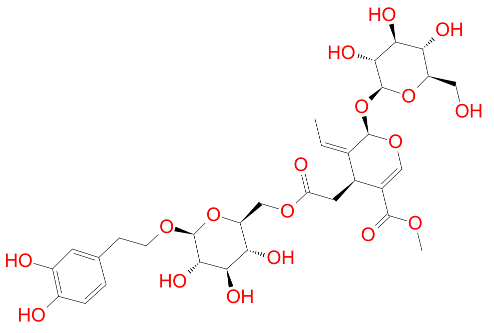

title P124_Neo-nuzhenide

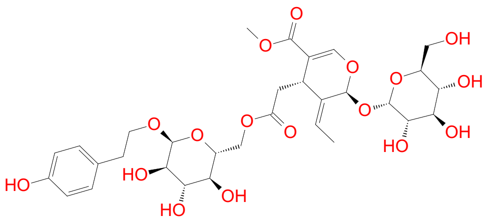

title P125_Nuzhenide.cdx

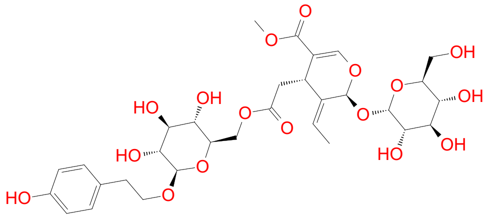

title P125_Nuzhenide.cdx

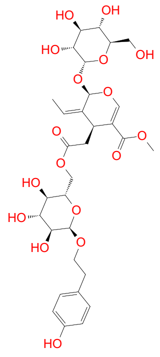

title P125_Nuzhenide.cdx

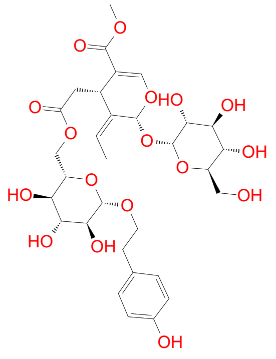

title P125_Nuzhenide.cdx

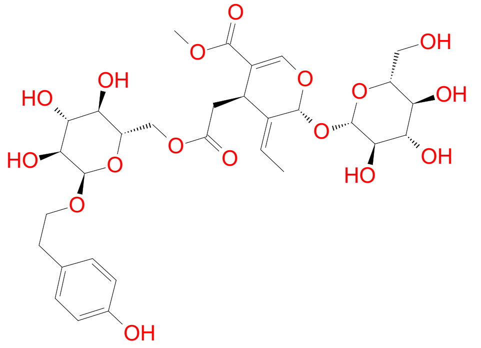

title P125_Nuzhenide.cdx

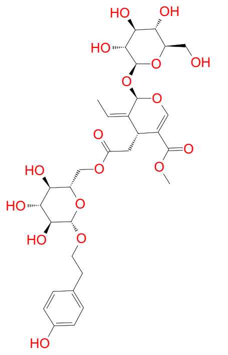

title P125_Nuzhenide.cdx

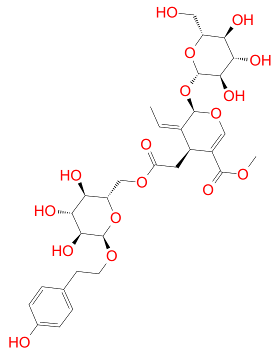

title P125_Nuzhenide.cdx

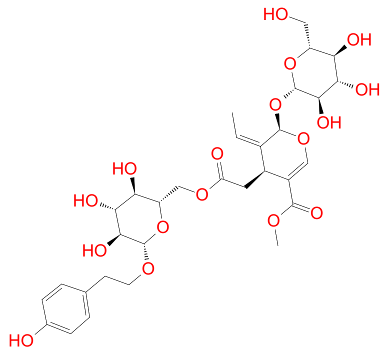

title P125_Nuzhenide.cdx

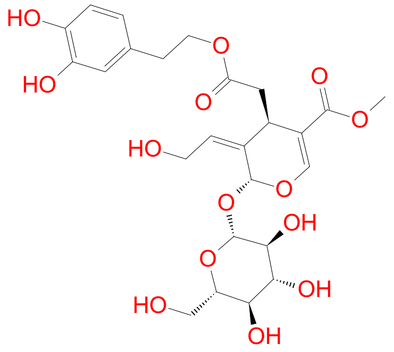

title P126_10-Hydroxyole

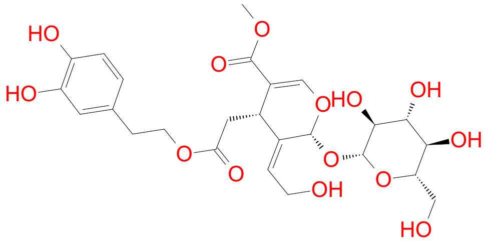

title P126_10-Hydroxyole

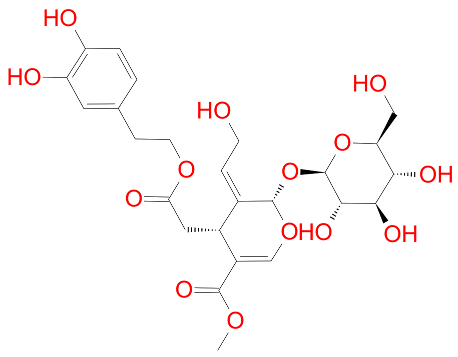

title P126_10-Hydroxyole

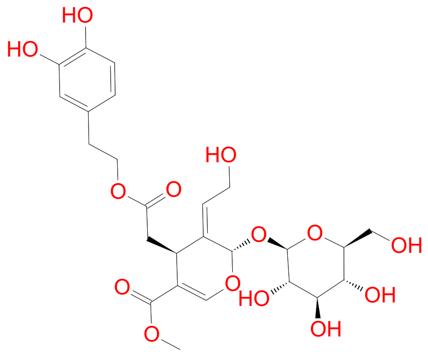

title P126_10-Hydroxyole

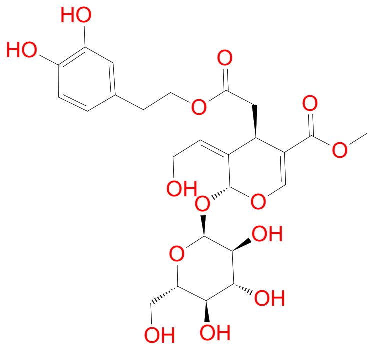

title P126_10-Hydroxyole

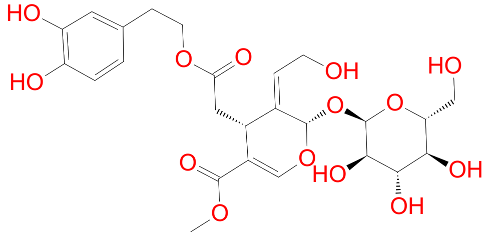

title P126_10-Hydroxyole

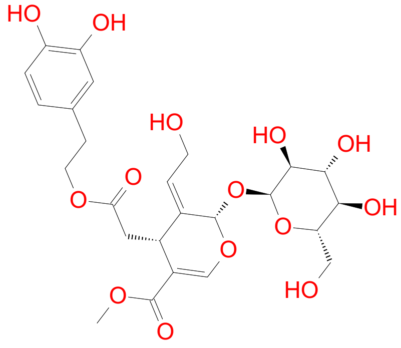

title P126_10-Hydroxyole

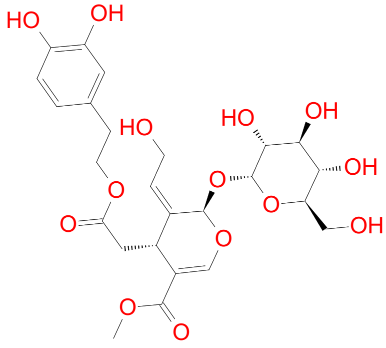

title P126_10-Hydroxyole

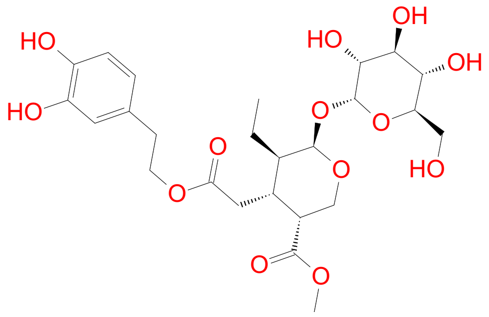

title P127_Dihydrooleuro

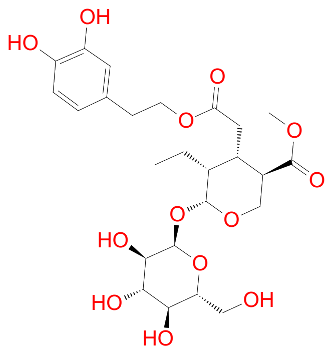

title P127_Dihydrooleurop

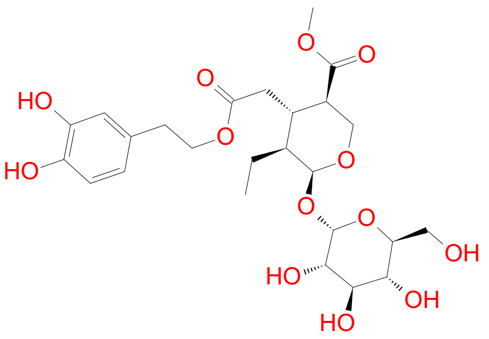

title P127_Dihydrooleurop

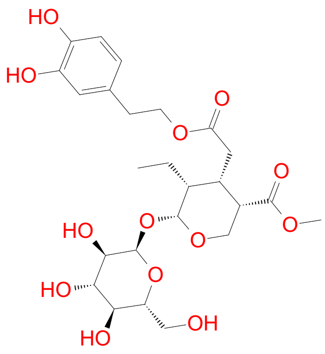

title P127_Dihydrooleurop

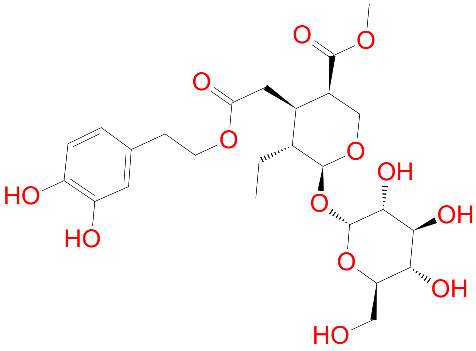

title P127_Dihydrooleurop

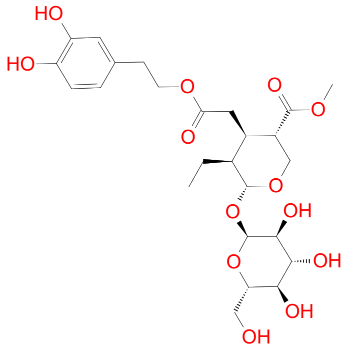

title P127_Dihydrooleurop

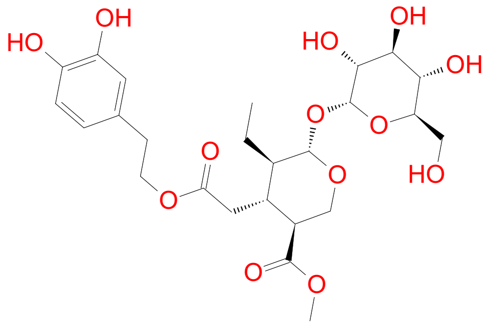

title P127_Dihydrooleurop

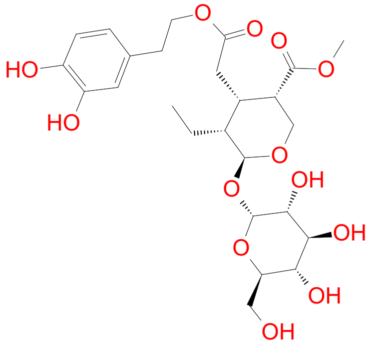

title P127_Dihydrooleurop

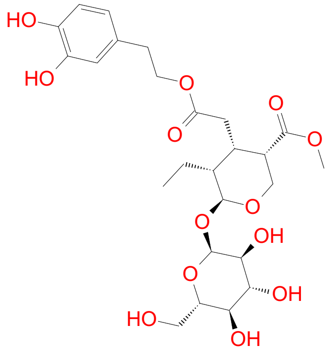

title P127_Dihydrooleurop

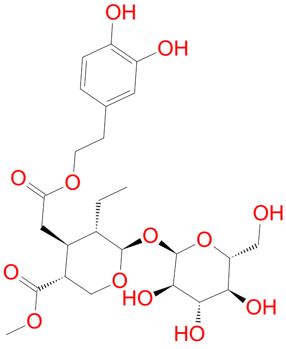

title P127_Dihydrooleurop

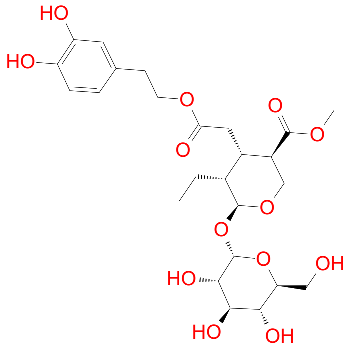

title P127_Dihydrooleurop

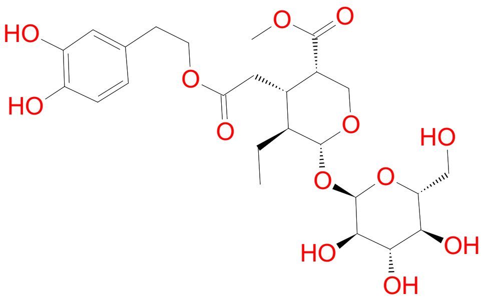

title P127_Dihydrooleurop

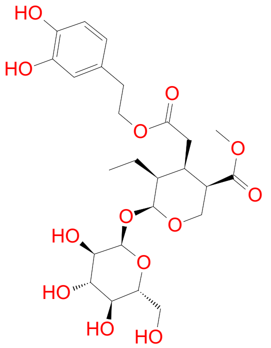

title P127_Dihydrooleurop

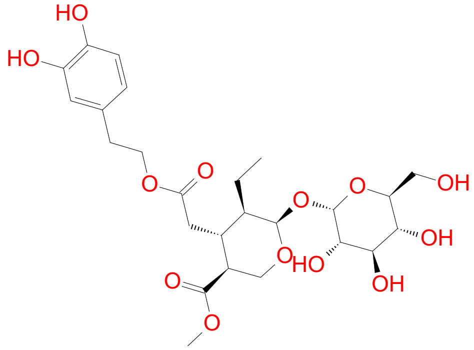

title P127_Dihydrooleurop

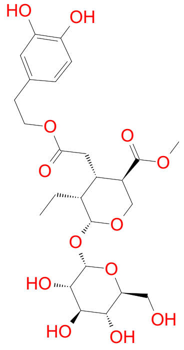

title P127_Dihydrooleurop

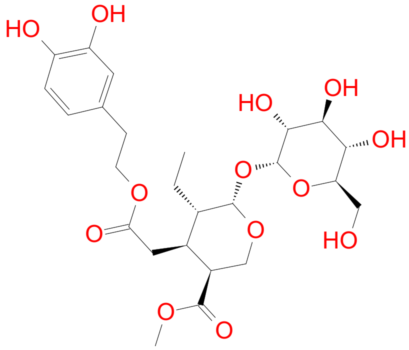

title P127_Dihydrooleurop

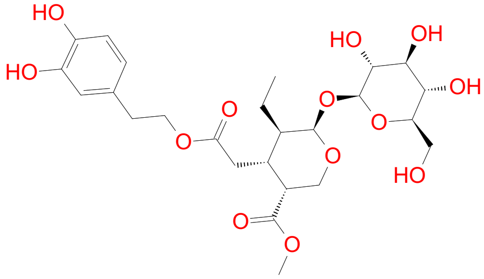

title P127_Dihydrooleurop

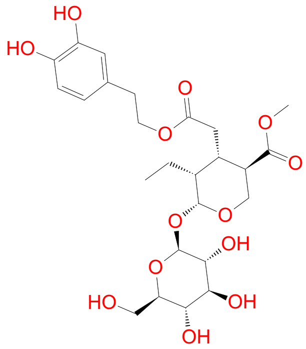

title P127_Dihydrooleurop

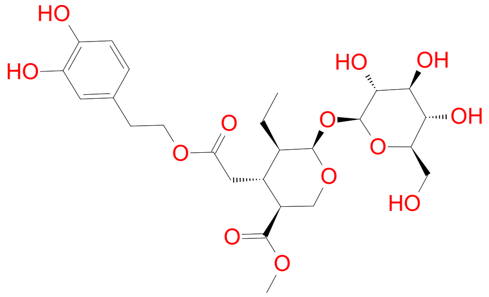

title P127_Dihydrooleurop

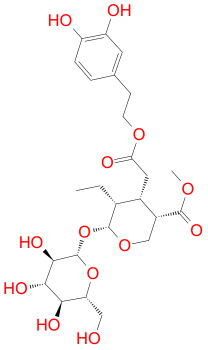

title P127_Dihydrooleurop

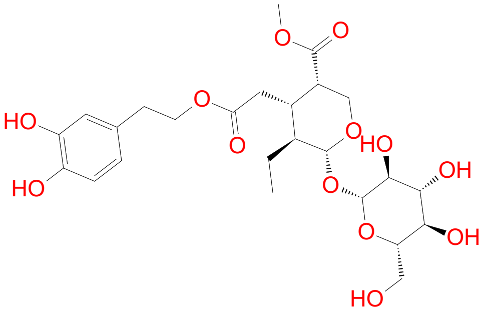

title P127_Dihydrooleurop

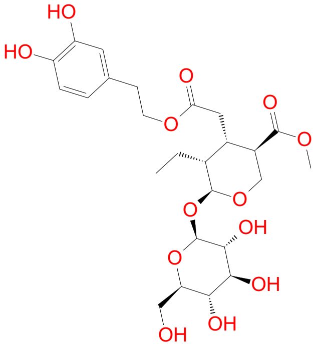

title P127_Dihydrooleurop

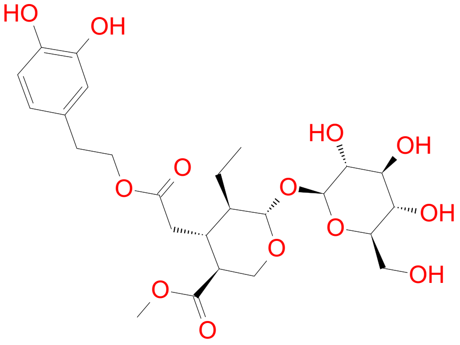

title P127_Dihydrooleurop

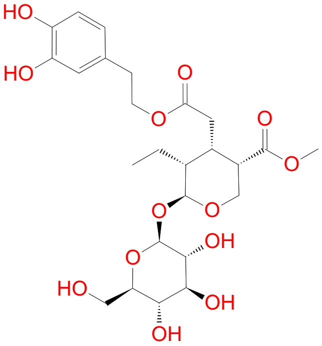

title P127_Dihydrooleurop

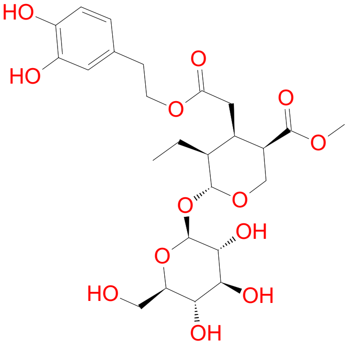

title P127_Dihydrooleurop

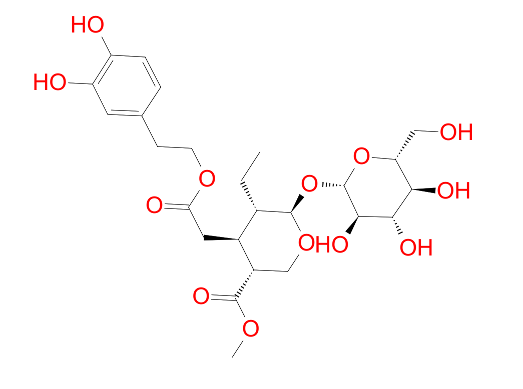

title P127_Dihydrooleurop

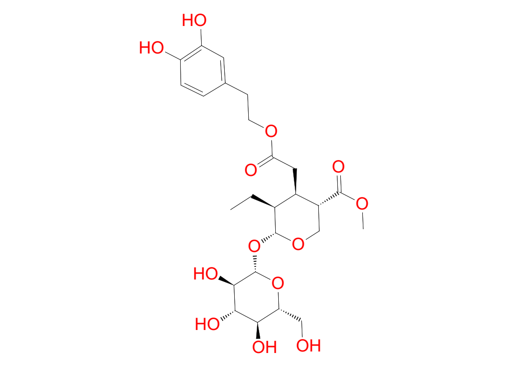

title P127_Dihydrooleurop

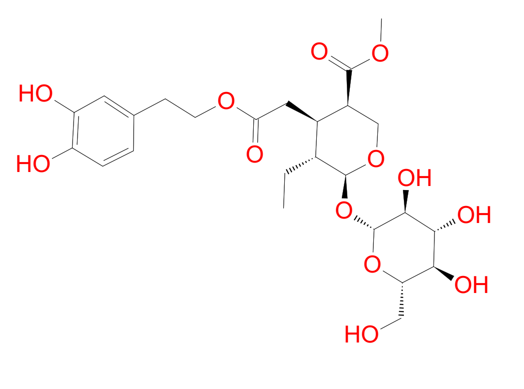

title P127_Dihydrooleurop

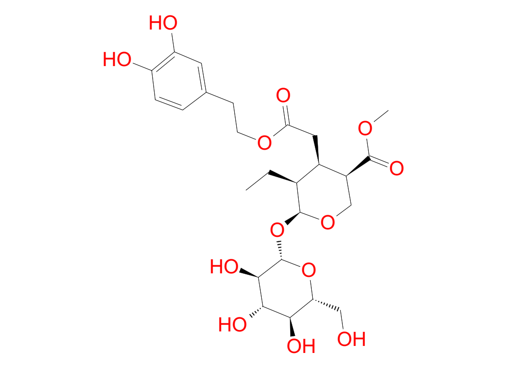

title P127_Dihydrooleurop

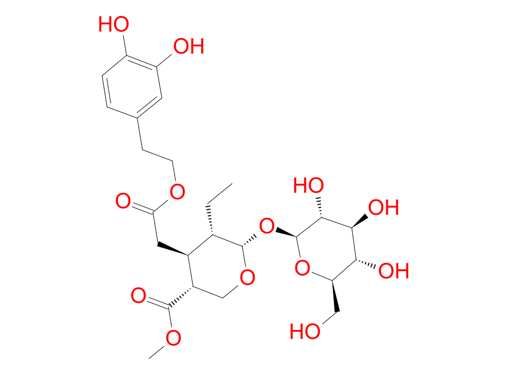

title P127_Dihydrooleurop

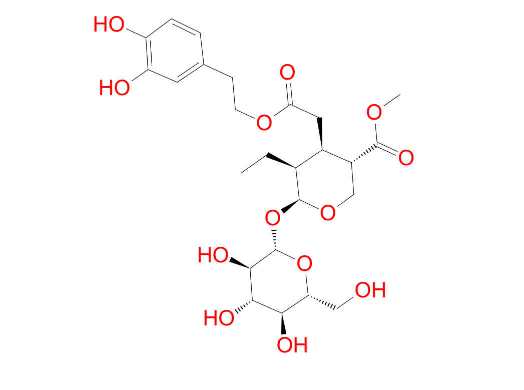

title P127_Dihydrooleurop

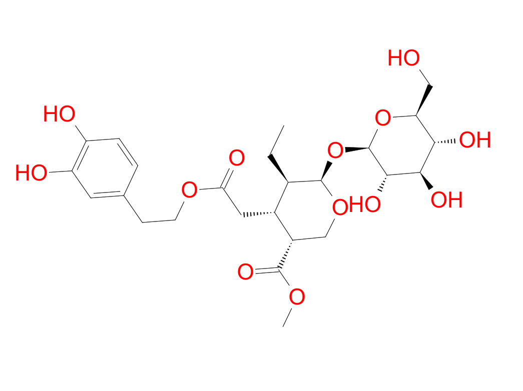

title P127_Dihydrooleurop

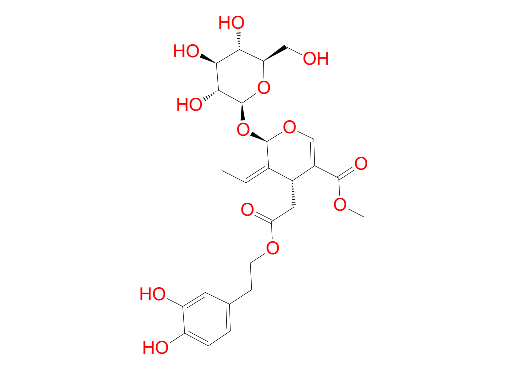

title P128_Oleuropein.cdx

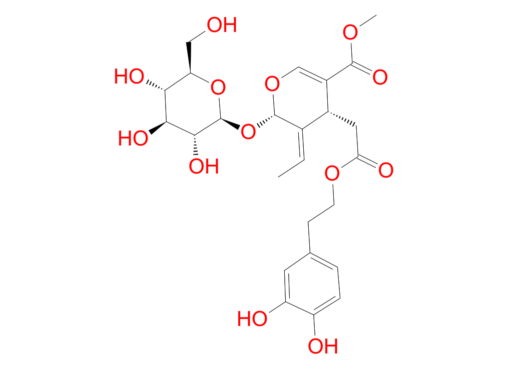

title P128_Oleuropein.cdx

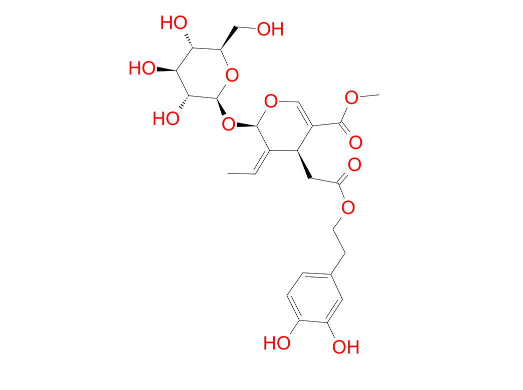

title P128_Oleuropein.cdx

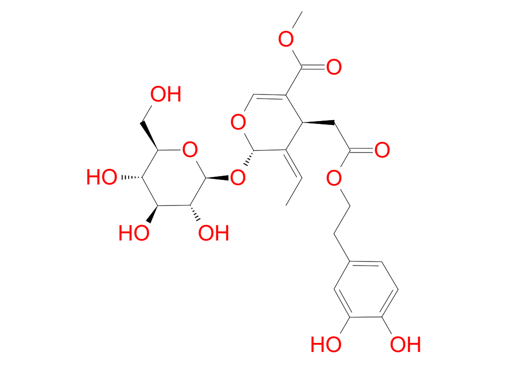

title P128_Oleuropein.cdx

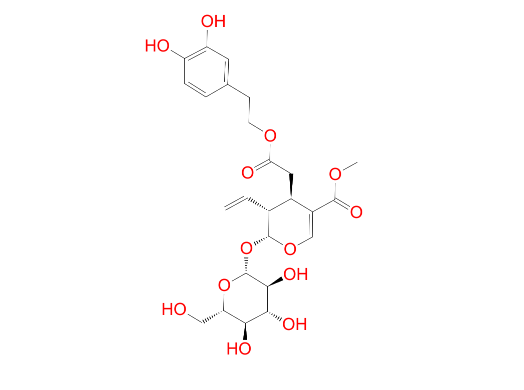

title P129_Oleuroside.cdx

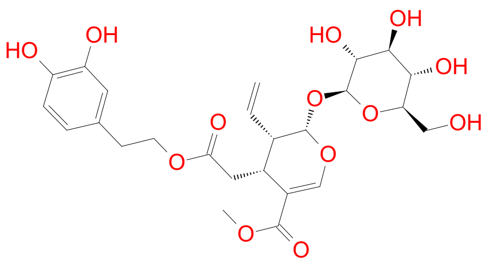

title P129_Oleuroside.cdx

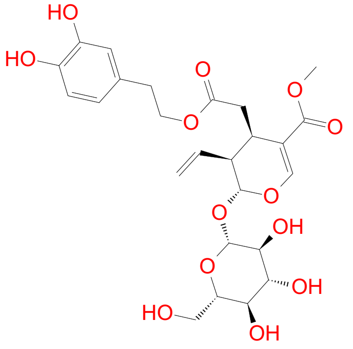

title P129_Oleuroside.cdx

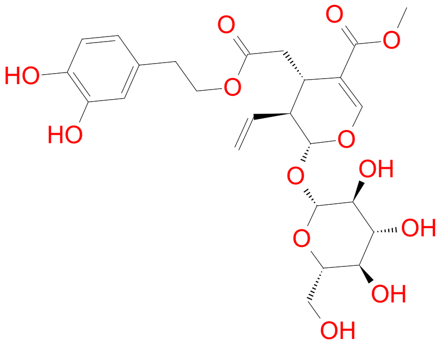

title P129_Oleuroside.cdx

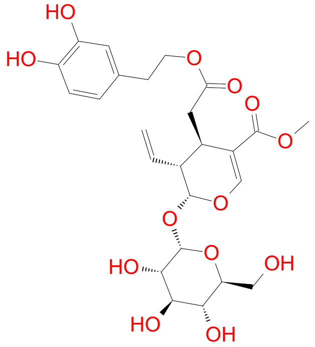

title P129_Oleuroside.cdx

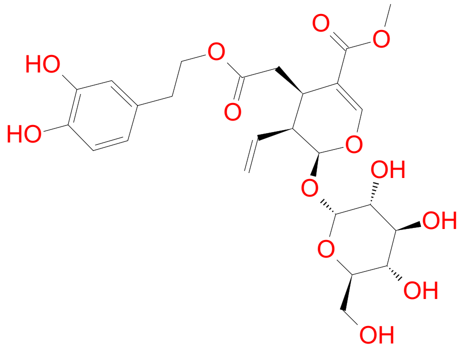

title P129_Oleuroside.cdx

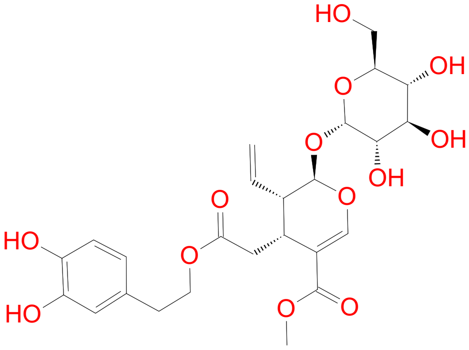

title P129_Oleuroside.cdx

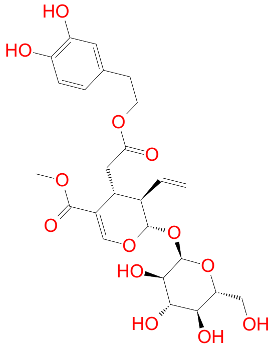

title P129_Oleuroside.cdx

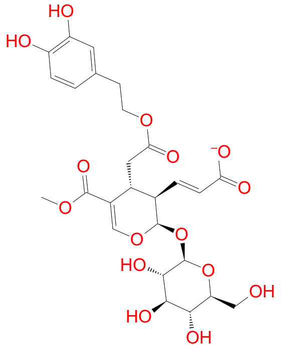

title P130_Oleuroside-10-

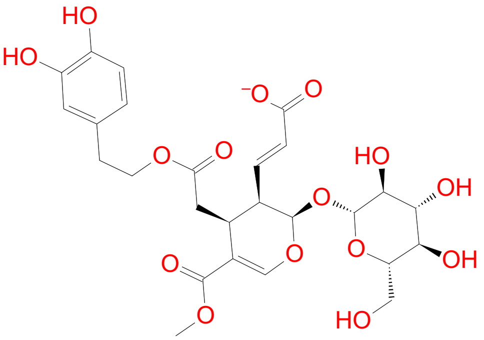

title P130_Oleuroside-10-

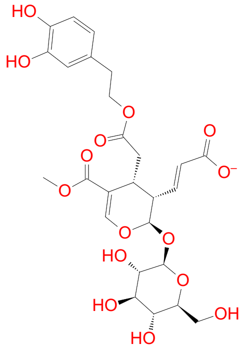

title P130_Oleuroside-10-

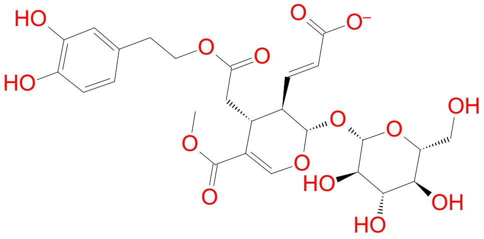

title P130_Oleuroside-10-

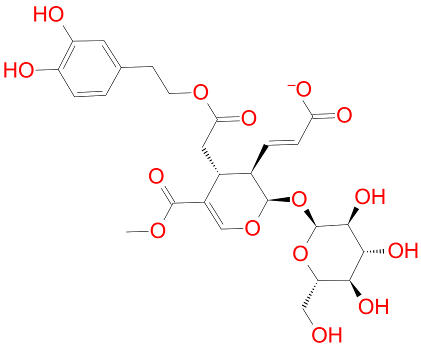

title P130_Oleuroside-10-

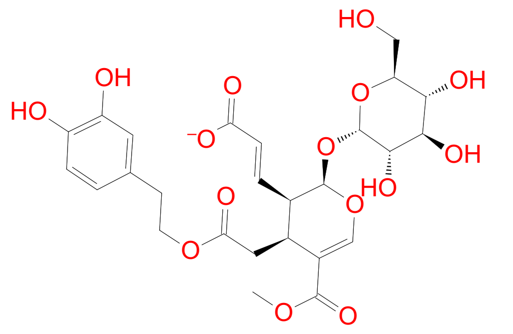

title P130_Oleuroside-10-

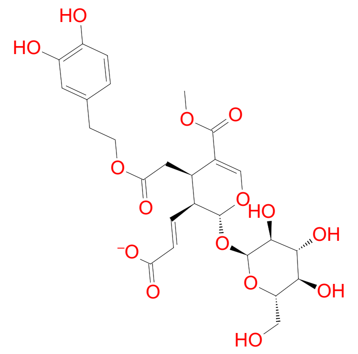

title P130_Oleuroside-10-

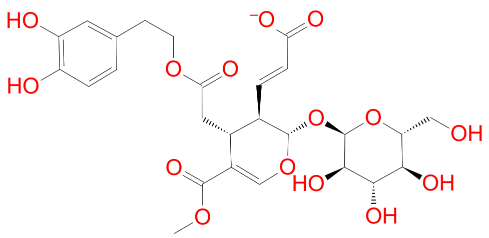

title P130_Oleuroside-10-

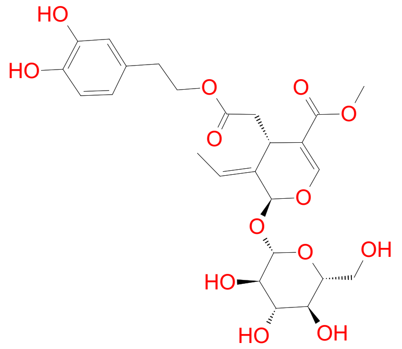

title P131_Oleuropein-3'-(

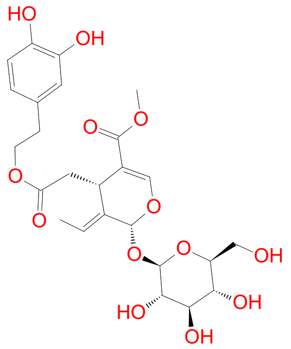

title P131_Oleuropein-3'-(

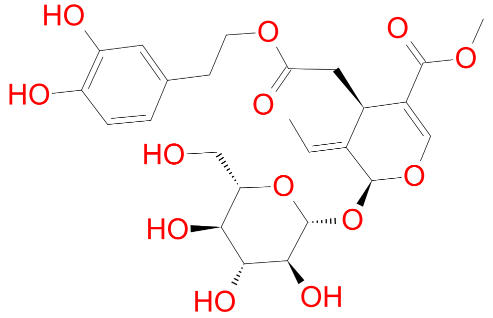

title P131_Oleuropein-3'-(

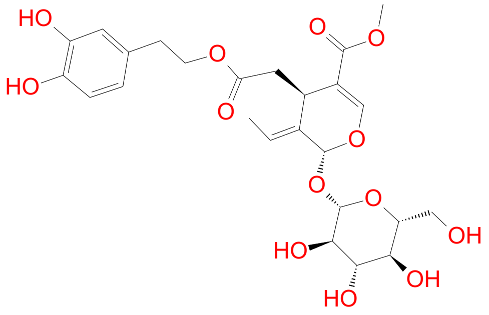

title P131_Oleuropein-3'-(

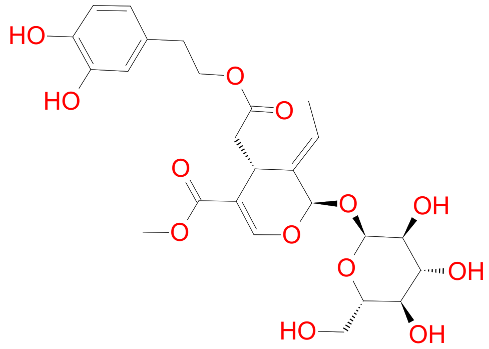

title P131_Oleuropein-3'-(

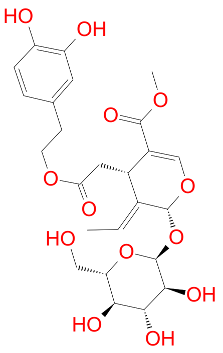

title P131_Oleuropein-3'-(

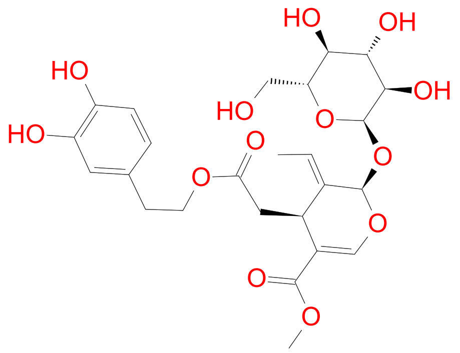

title P131_Oleuropein-3'-(

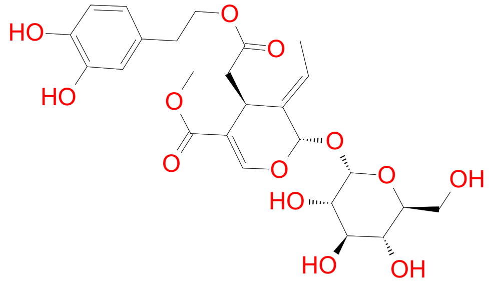

title P131_Oleuropein-3'-(

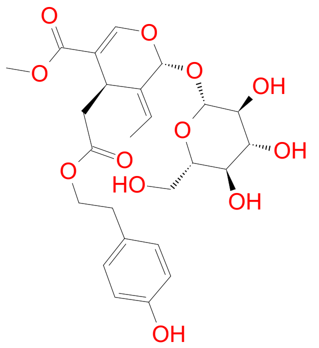

title P132_Ligstroside-3'-(

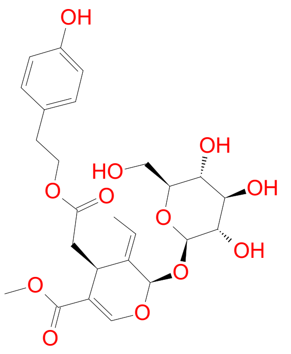
title P132_Ligstroside-3'-(

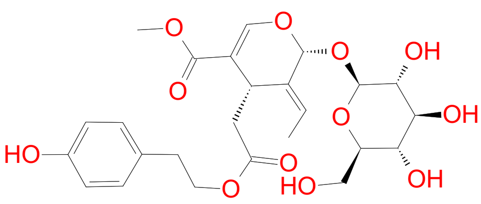
title P132_Ligstroside-3'-(

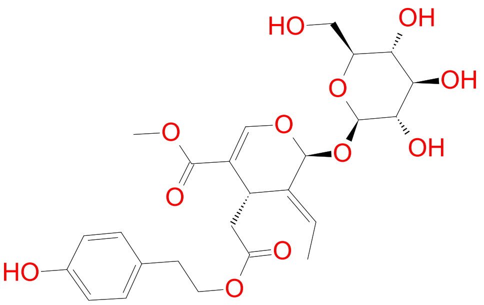
title P132_Ligstroside-3'-(

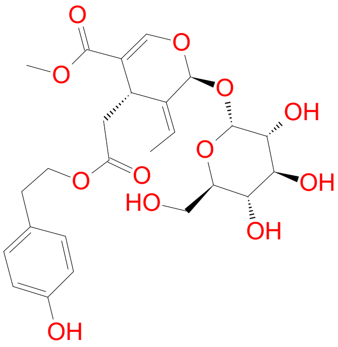
title P132_Ligstroside-3'-(

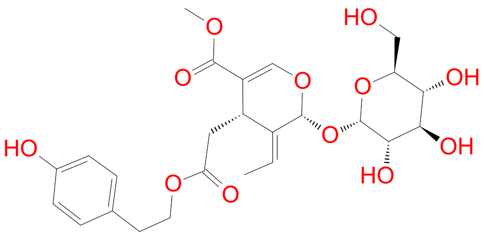
title P132_Ligstroside-3'-(

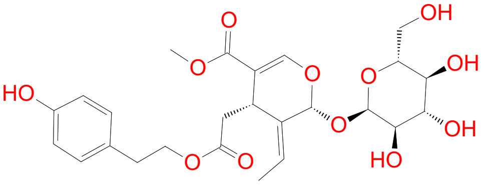
title P132_Ligstroside-3'-(

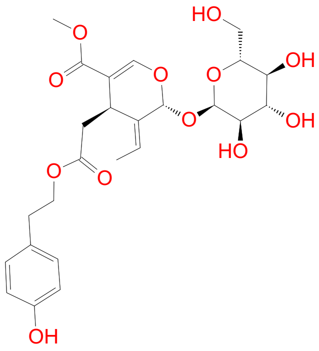
title P132_Ligstroside-3'-(

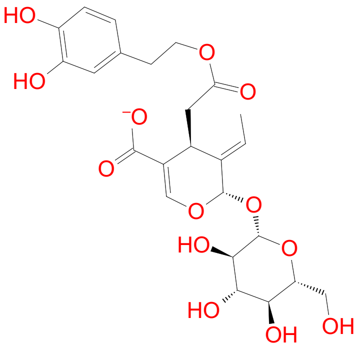
title P133_Demethyloleur

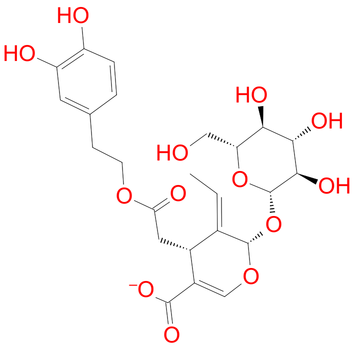
title P133_Demethyloleur

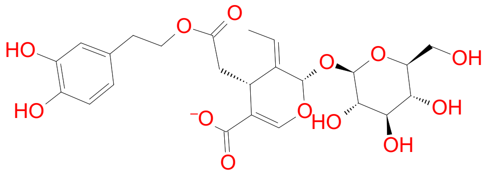
title P133_Demethyloleur

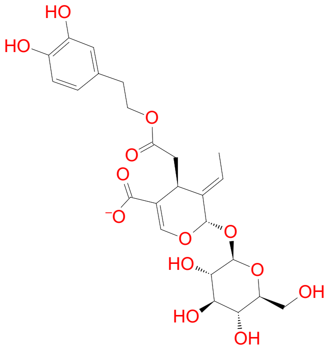
title P133_Demethyloleur

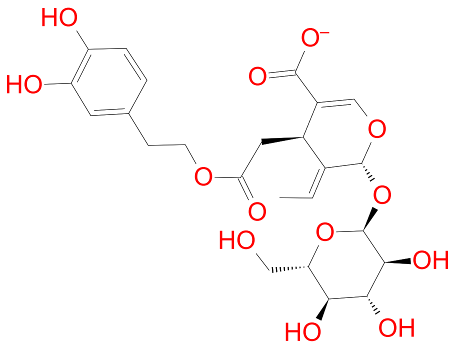
title P133_Demethyloleur

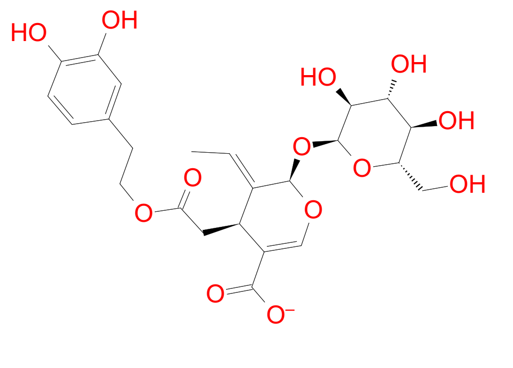

title P133_Demethyloleur

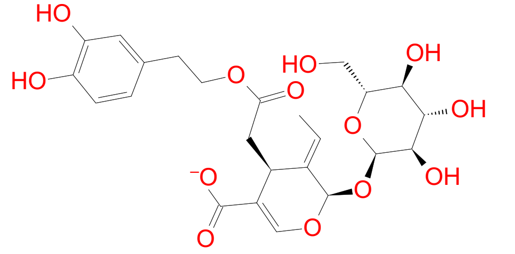

title P133_Demethyloleur

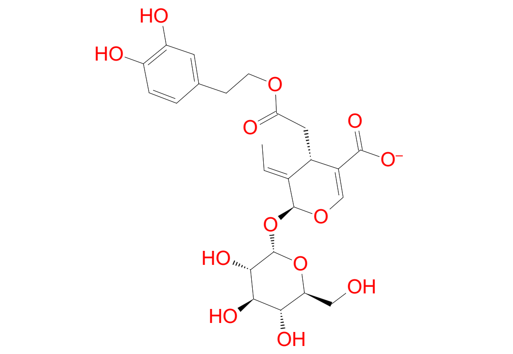

title P133_Demethyloleur

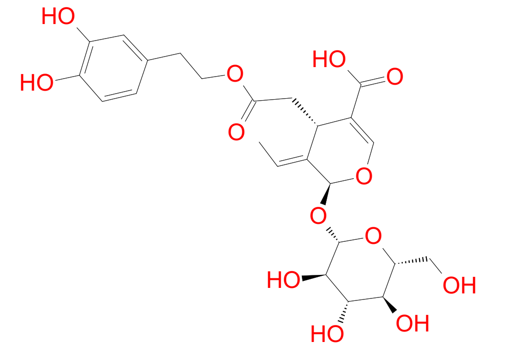

title P133_Demethyloleur

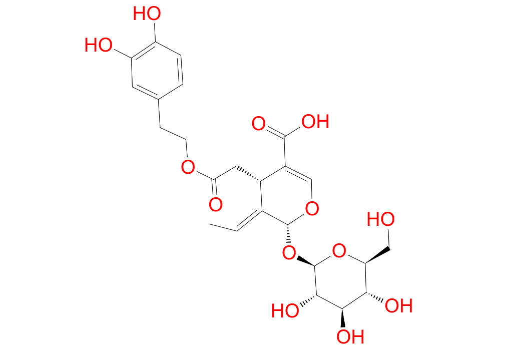

title P133_Demethyloleur

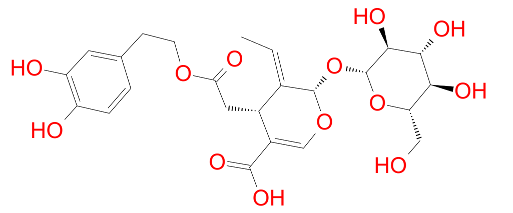

title P133_Demethyloleur

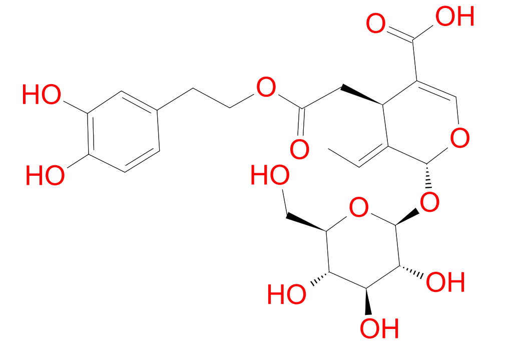

title P133_Demethyloleur

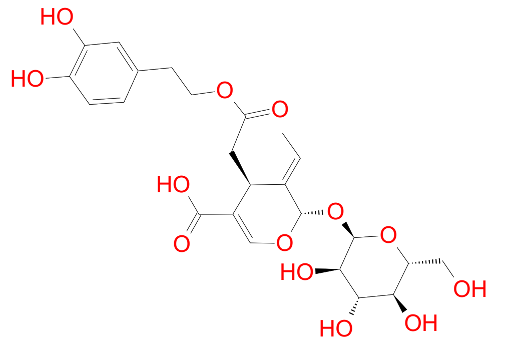

title P133_Demethyloleur

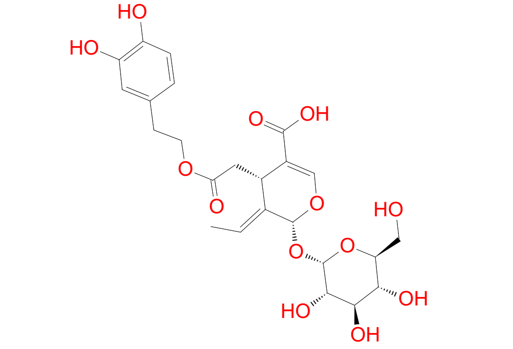

title P133_Demethyloleur

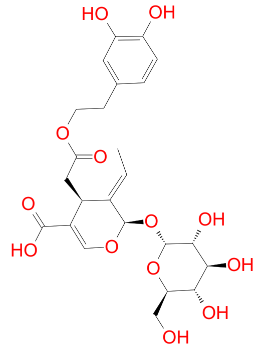

title P133_Demethyloleur

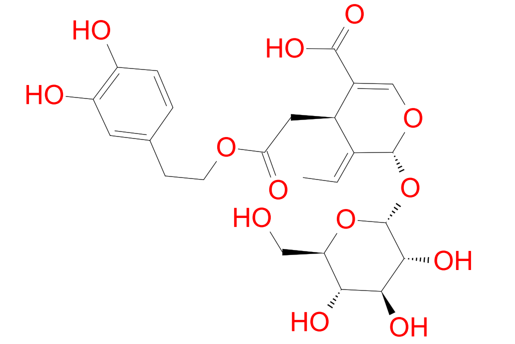

title P133_Demethyloleur

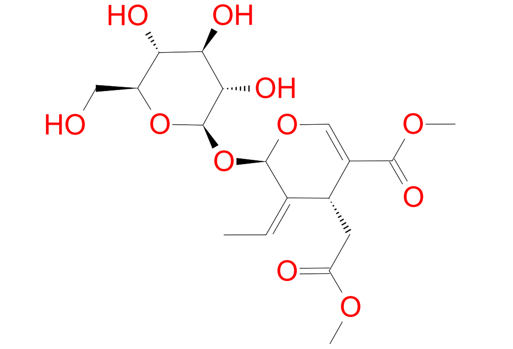

title P134_Oleoside dimet

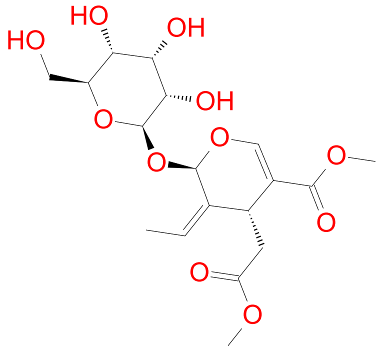

title P134_Oleoside dimet

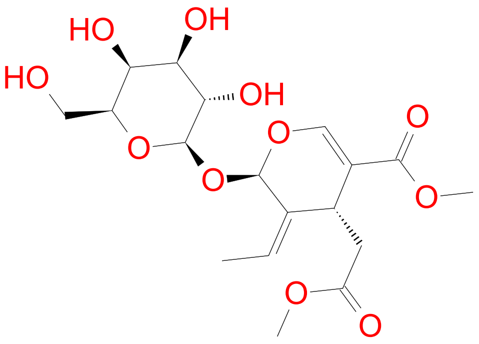

title P134_Oleoside dimet

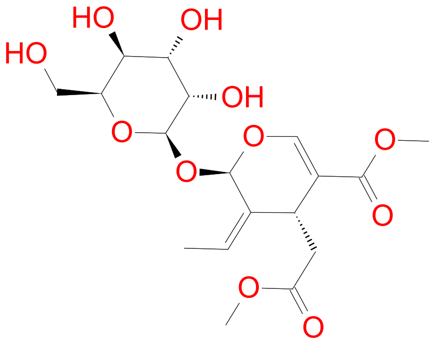

title P134_Oleoside dimet

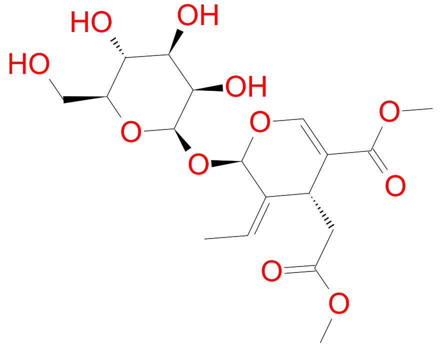

title P134_Oleoside dimet

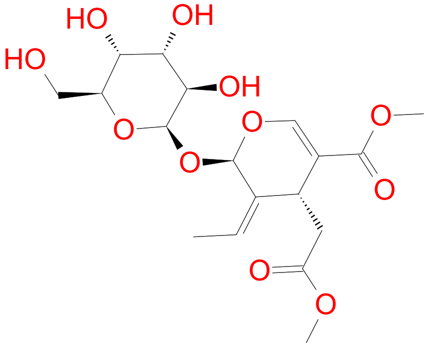

title P134_Oleoside dimet

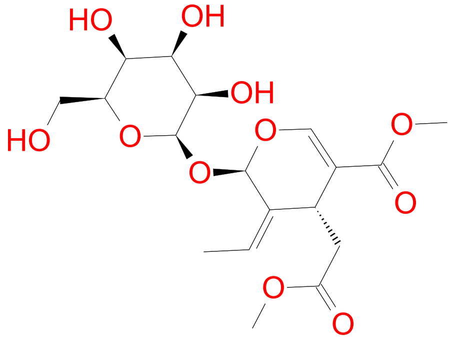

title P134_Oleoside dimet

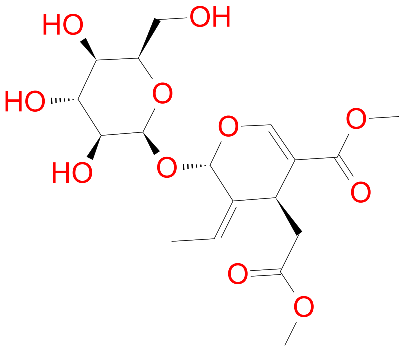

title P134_Oleoside dimet

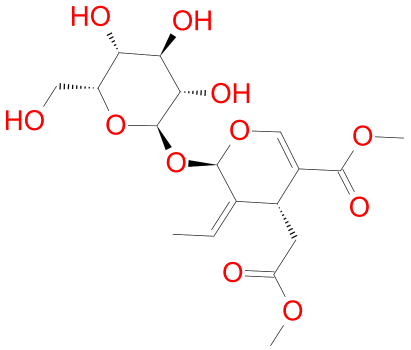

title P134_Oleoside dimet

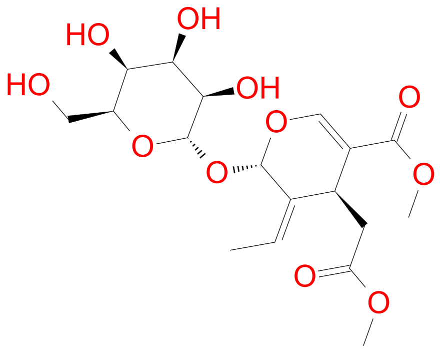

title P134_Oleoside dimet

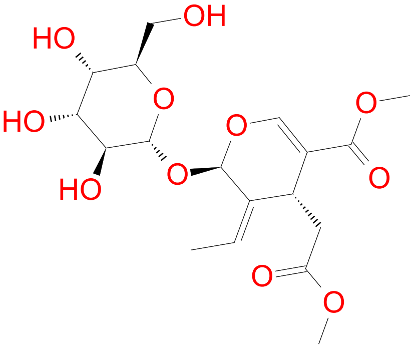

title P134_Oleoside dimet

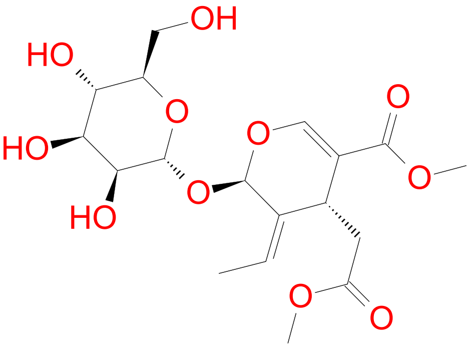

title P134_Oleoside dimet

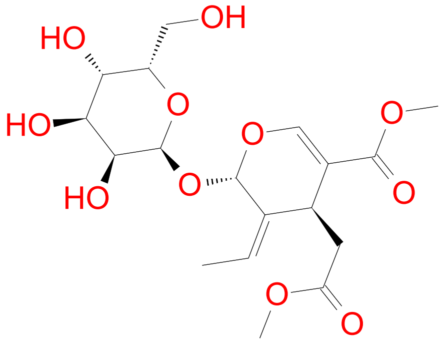

title P134_Oleoside dimet

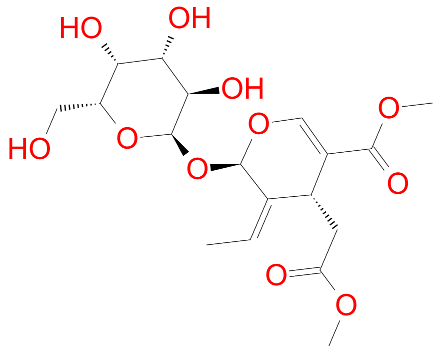

title P134_Oleoside dimet

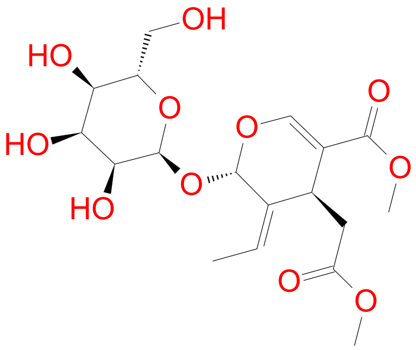

title P134_Oleoside dimet

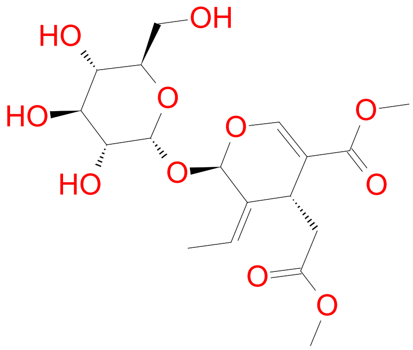

title P134_Oleoside dimet

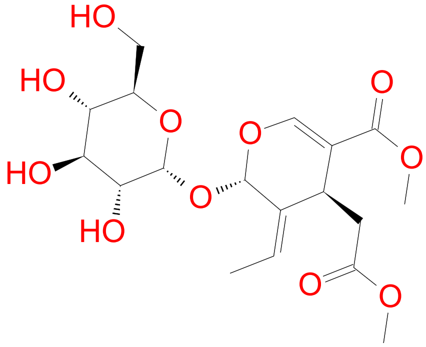

title P134_Oleoside dimet

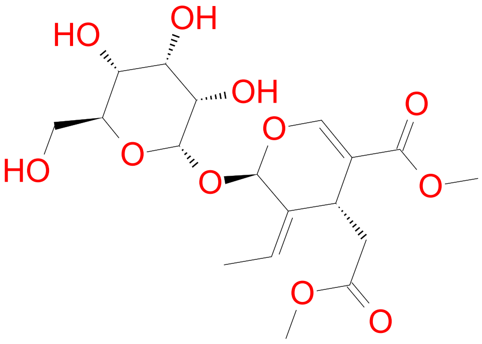

title P134_Oleoside dimet

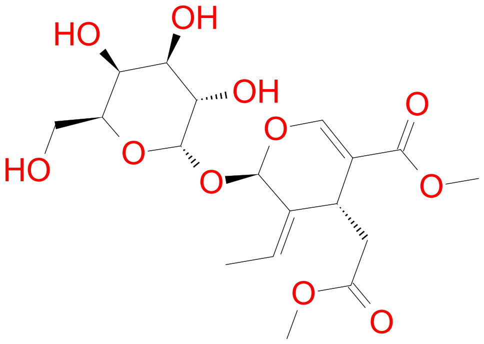

title P134_Oleoside dimet

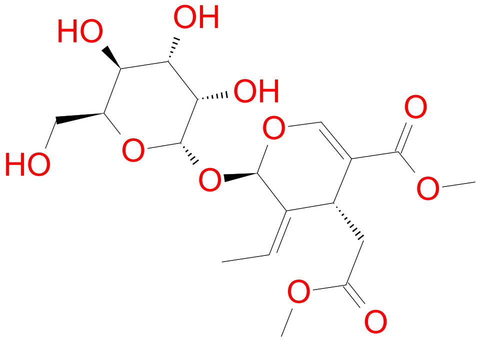

title P134_Oleoside dimet

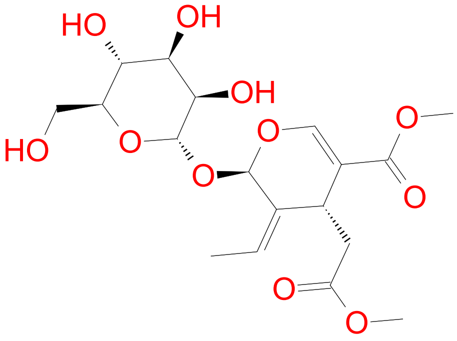

title P134_Oleoside dimet

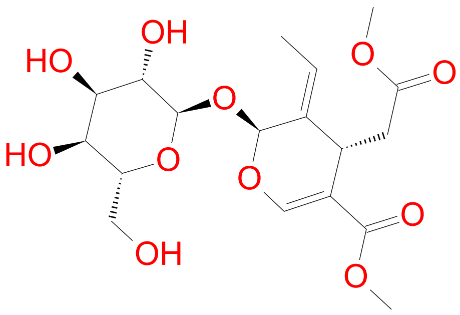

title P134_Oleoside dimet

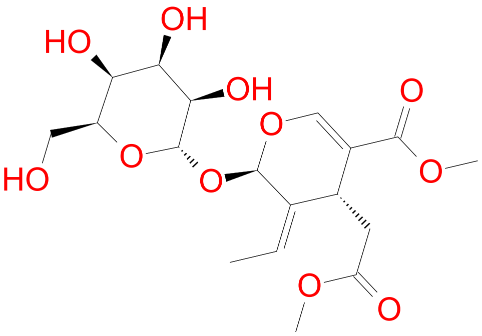

title P134_Oleoside dimet

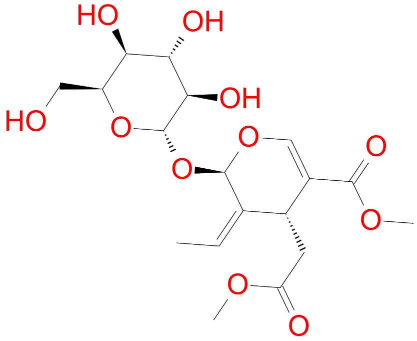

title P134_Oleoside dimet

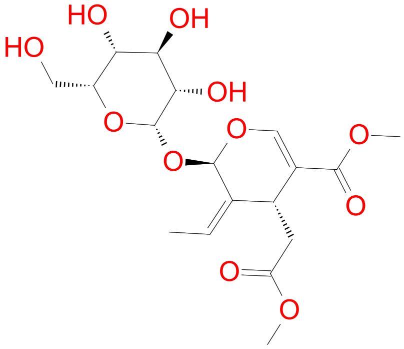

title P134_Oleoside dimet

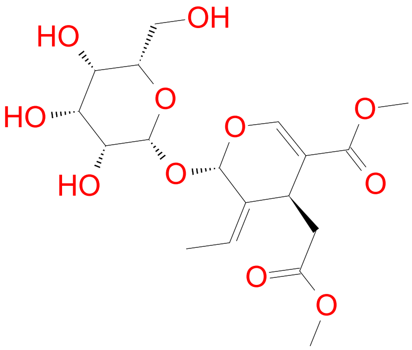

title P134_Oleoside dimet

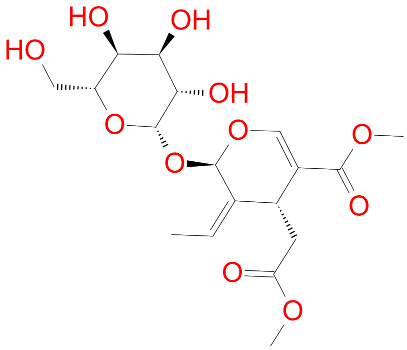

title P134_Oleoside dimet

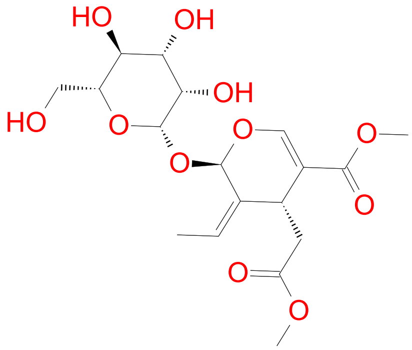

title P134_Oleoside dimet

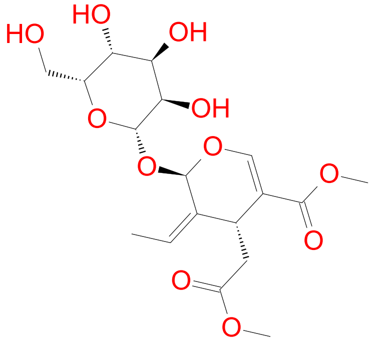

title P134_Oleoside dimet

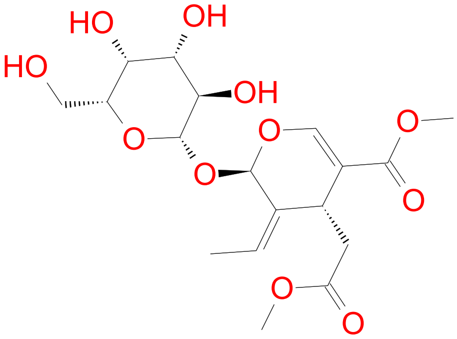

title P134_Oleoside dimet

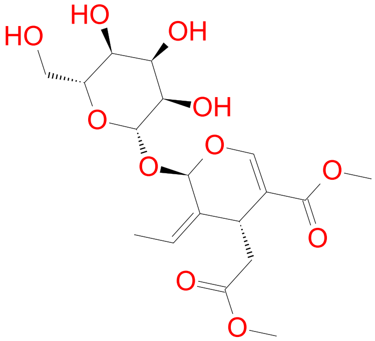

title P134_Oleoside dimet

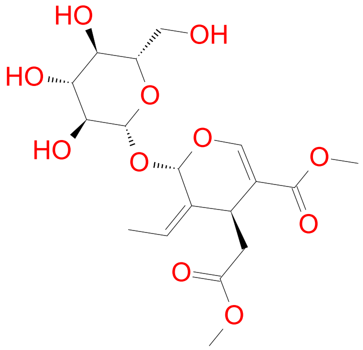

title P134_Oleoside dimet

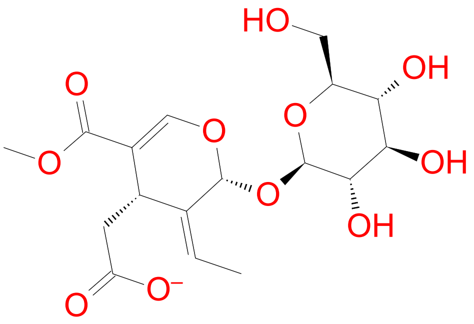

title P135_Elenolic acid gl

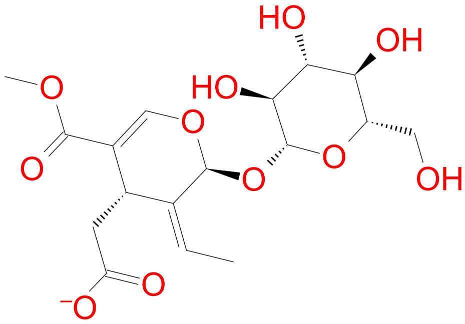

title P135_Elenolic acid gl

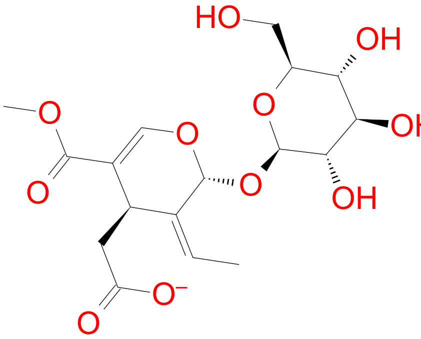

title P135_Elenolic acid gl

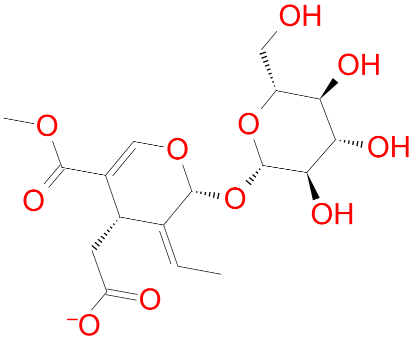

title P135_Elenolic acid gl

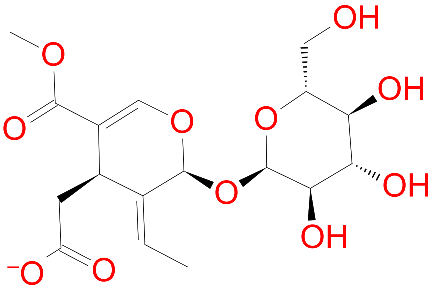

title P135_Elenolic acid gl

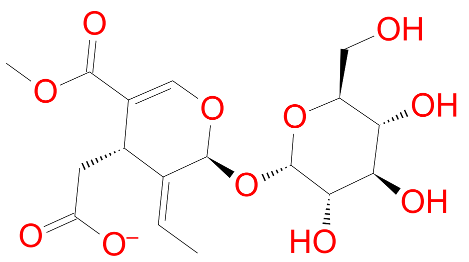
title P135_Elenolic acid gl

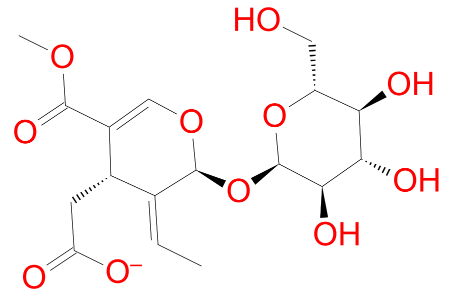
title P135_Elenolic acid gl

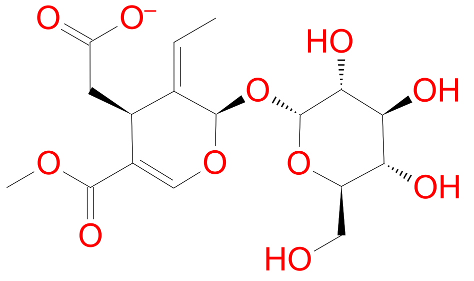
title P135_Elenolic acid gl

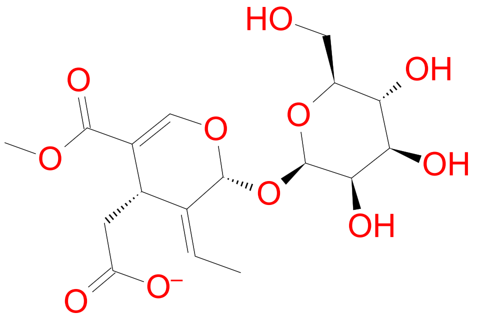
title P135_Elenolic acid gl

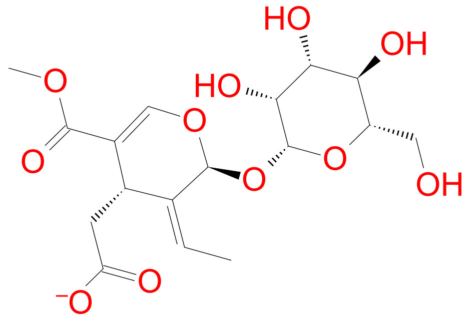
title P135_Elenolic acid gl

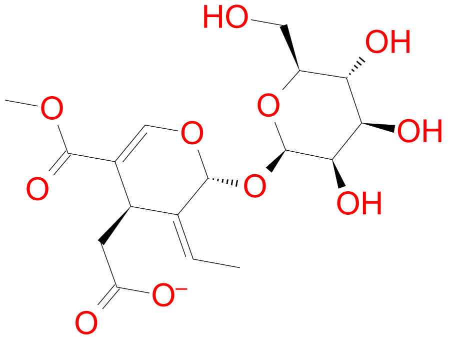
title P135_Elenolic acid gl

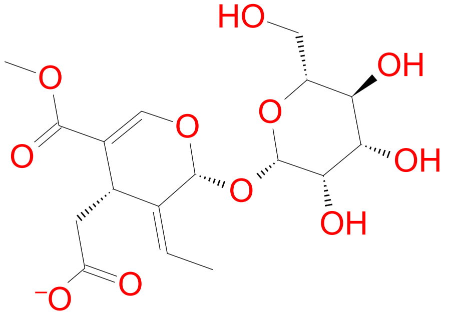
title P135_Elenolic acid gl

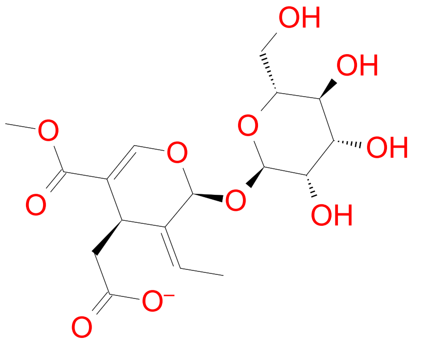
title P135_Elenolic acid gl

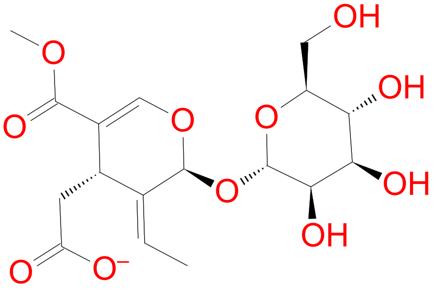
title P135_Elenolic acid gl

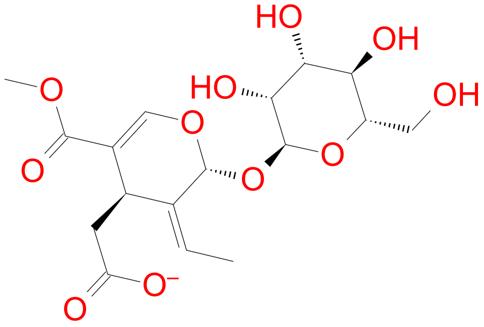
title P135_Elenolic acid gl

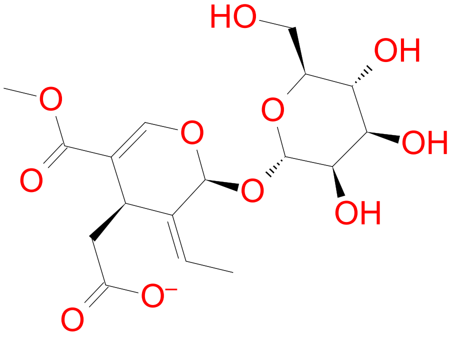
title P135_Elenolic acid gl

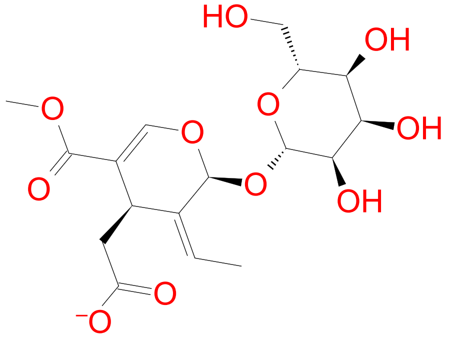
title P135_Elenolic acid gl

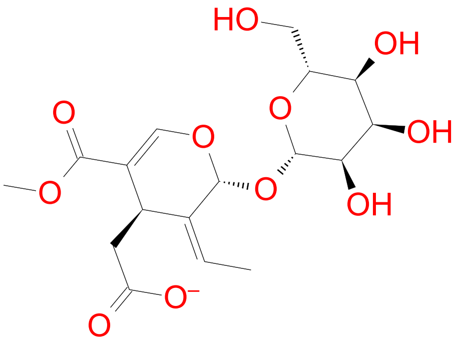

title P135_Elenolic acid gl

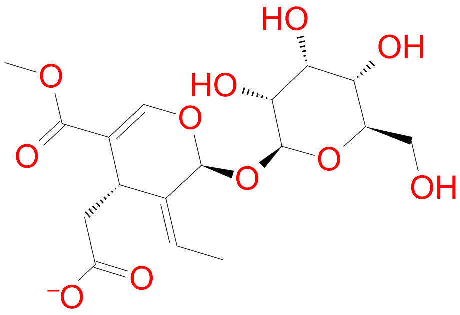

title P135_Elenolic acid gl

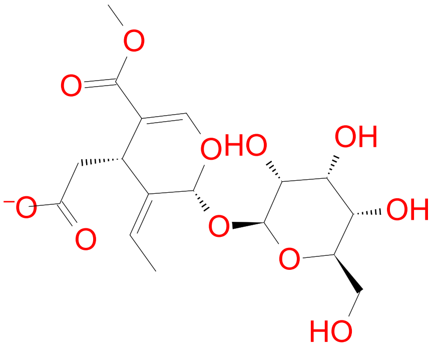

title P135_Elenolic acid gl

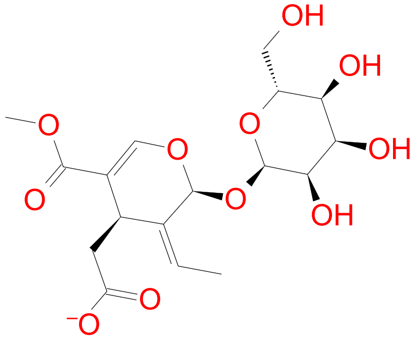

title P135_Elenolic acid gl

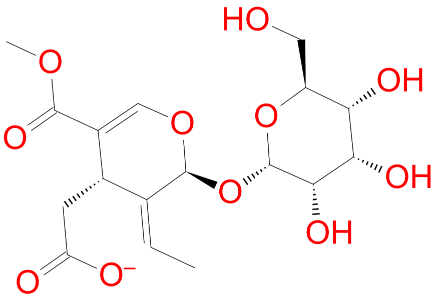

title P135_Elenolic acid gl

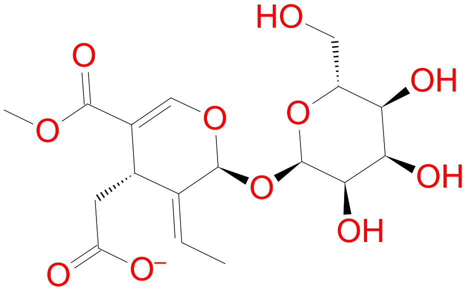

title P135_Elenolic acid gl

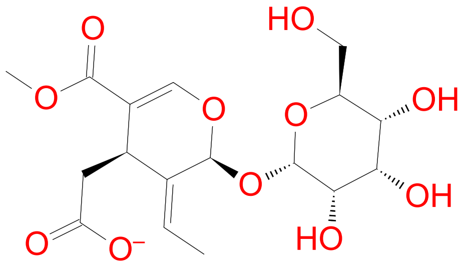

title P135_Elenolic acid gl

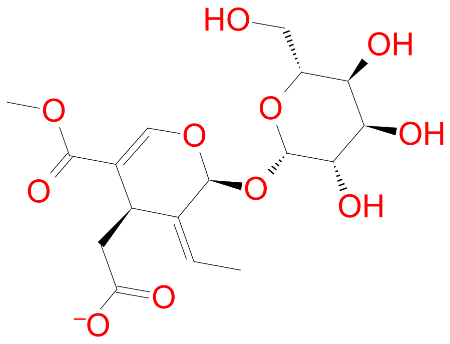

title P135_Elenolic acid gl

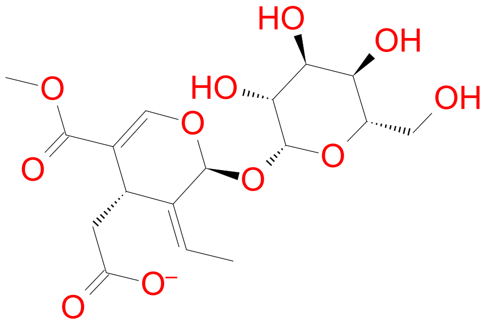

title P135_Elenolic acid gl

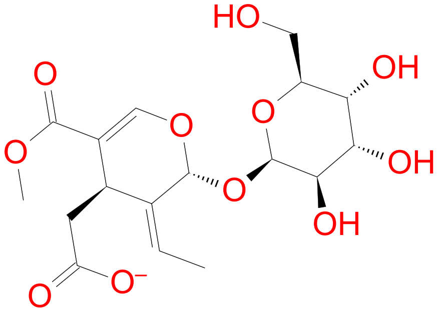

title P135_Elenolic acid gl

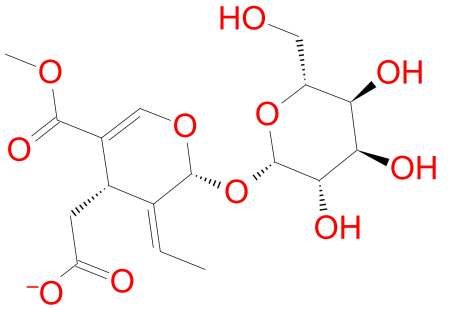

title P135_Elenolic acid gl

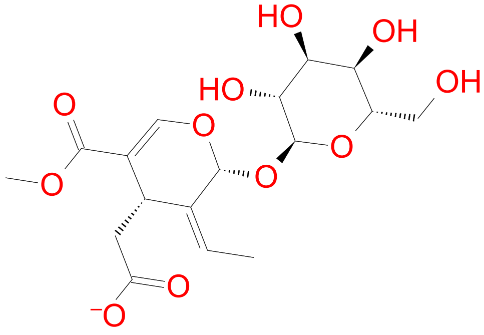

title P135_Elenolic acid gl

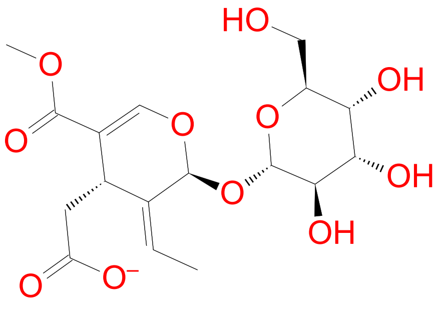
title P135_Elenolic acid gl

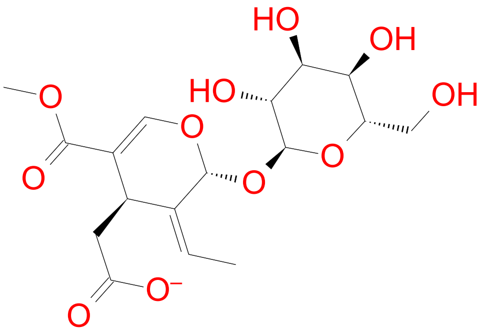
title P135_Elenolic acid gl

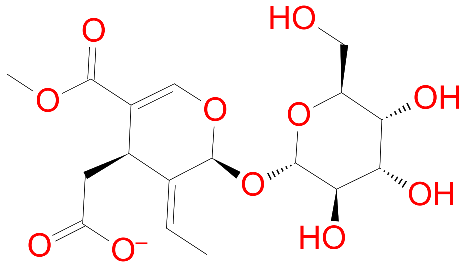
title P135_Elenolic acid gl

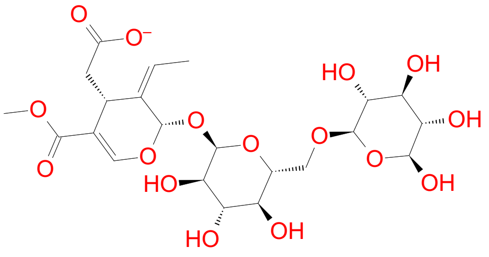
title P136_Elenolic acid di

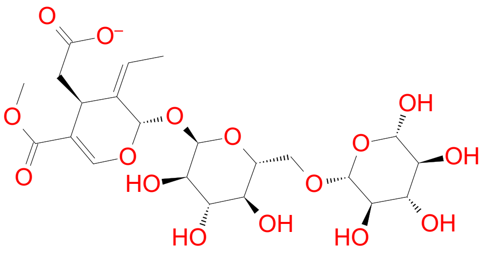
title P136_Elenolic acid di

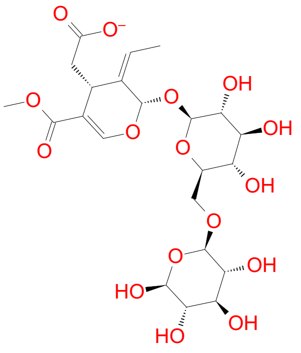
title P136_Elenolic acid di

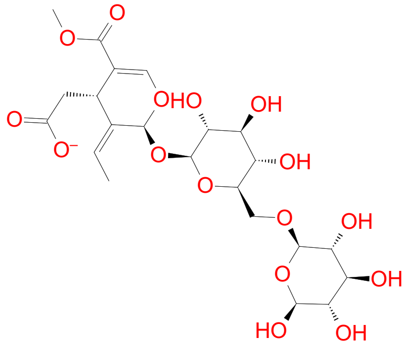
title P136_Elenolic acid di

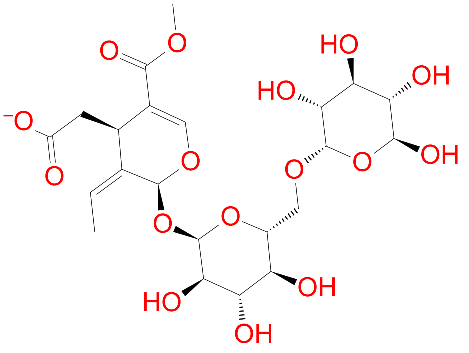
title P136_Elenolic acid di

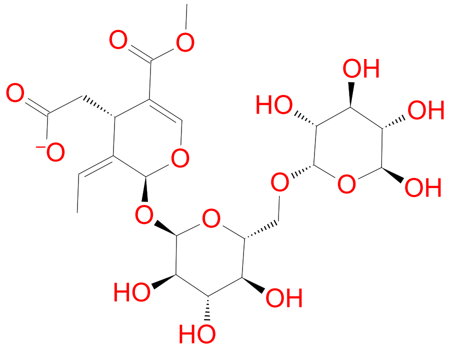
title P136_Elenolic acid di

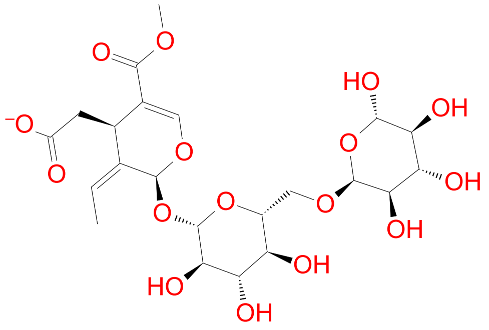
title P136_Elenolic acid di

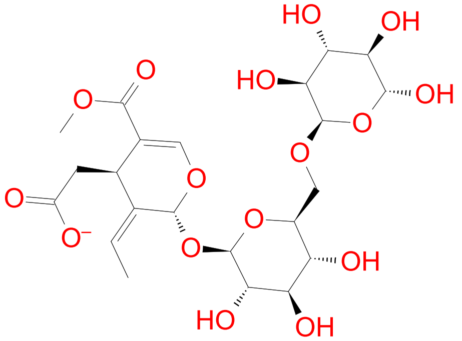
title P136_Elenolic acid di

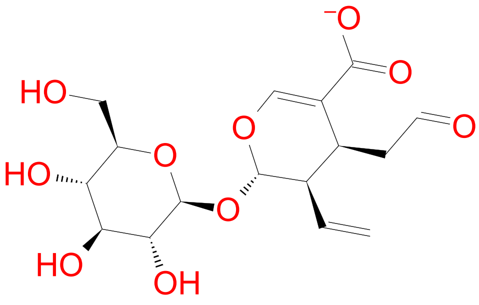
title P137_Secologanic ac

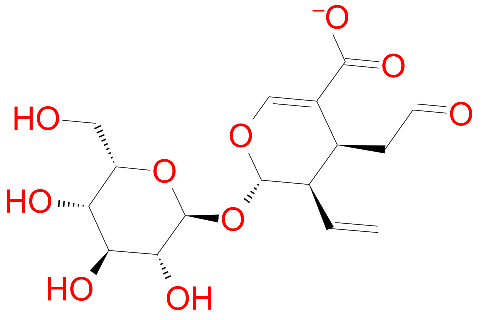

title P137_Secologanic ac

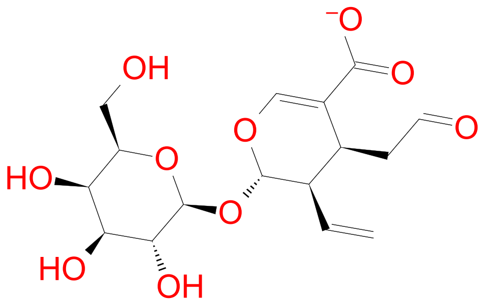

title P137_Secologanic ac

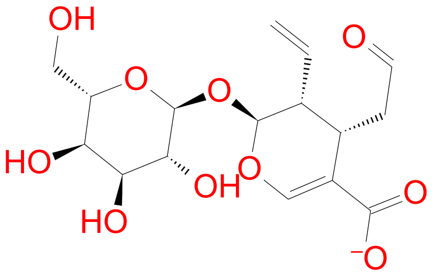

title P137_Secologanic ac

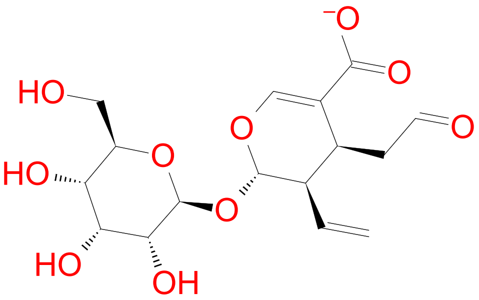

title P137_Secologanic ac

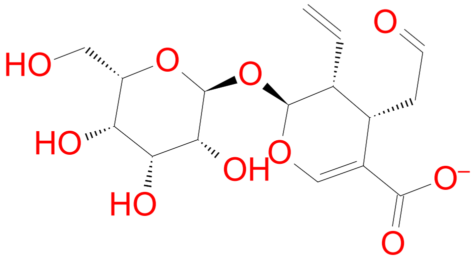

title P137_Secologanic ac

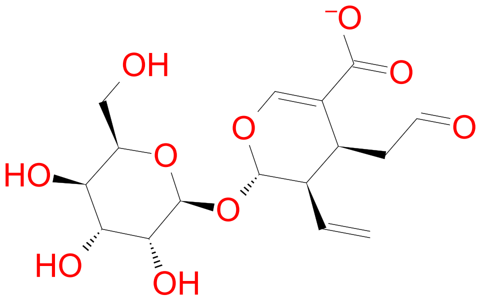

title P137_Secologanic ac

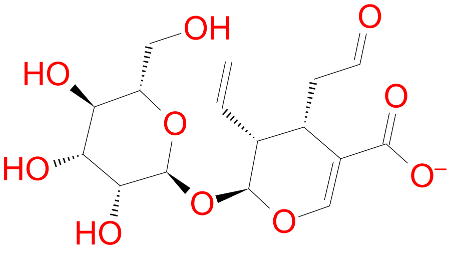

title P137_Secologanic ac

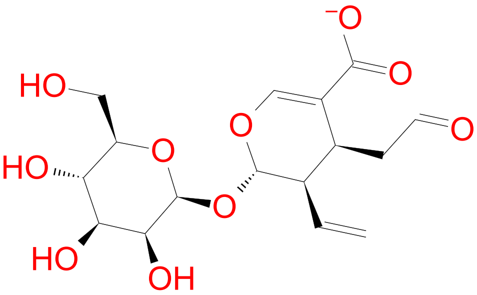

title P137_Secologanic ac

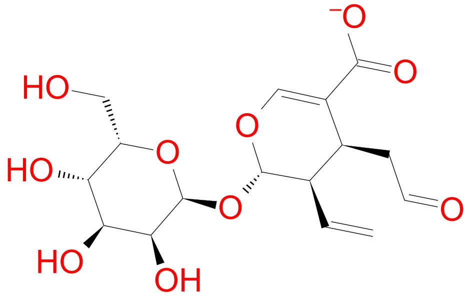

title P137_Secologanic ac

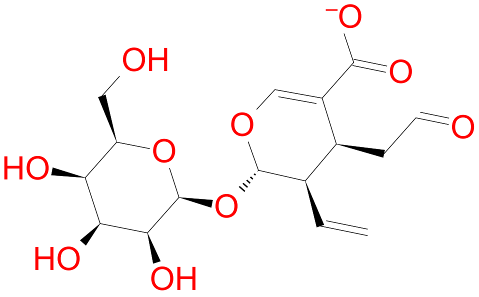

title P137_Secologanic ac

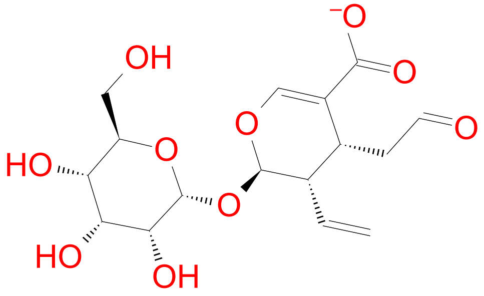

title P137_Secologanic ac

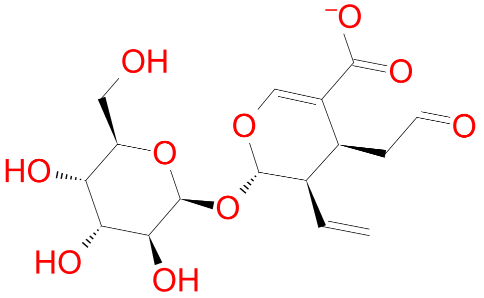

title P137_Secologanic ac

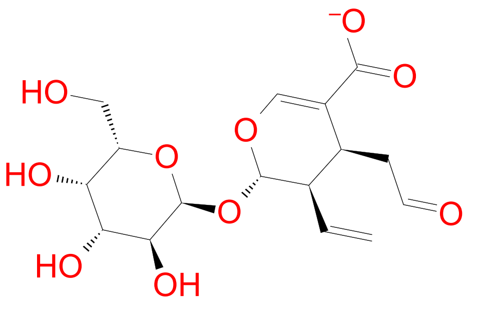

title P137_Secologanic ac

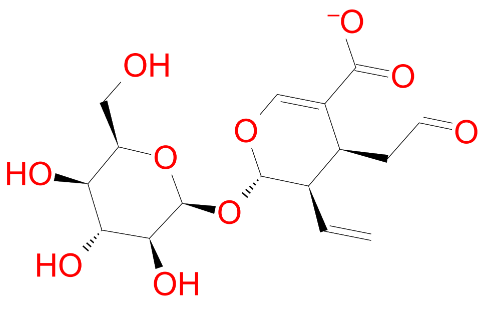

title P137_Secologanic ac

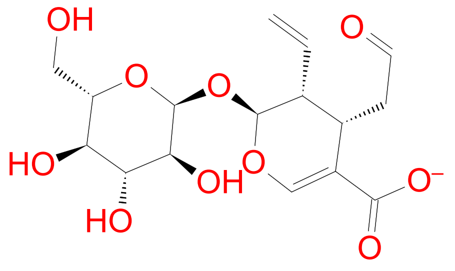

title P137_Secologanic ac

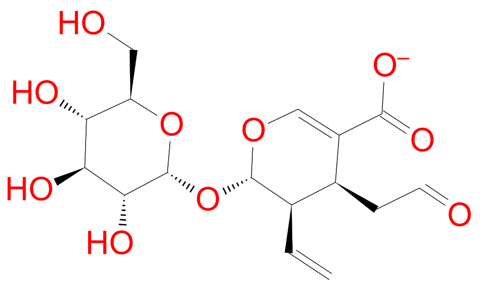

title P137_Secologanic ac

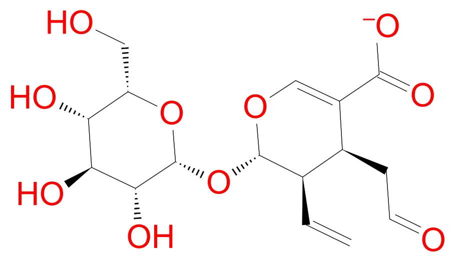

title P137_Secologanic ac

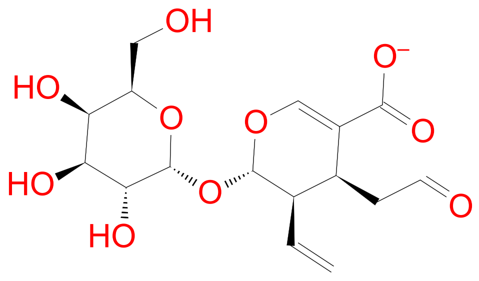

title P137_Secologanic ac

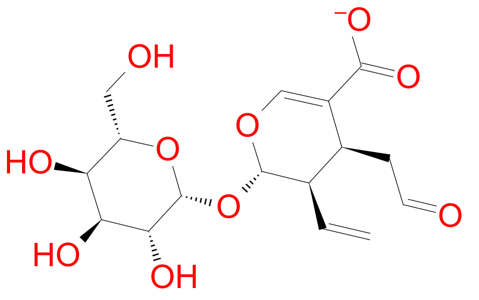

title P137_Secologanic ac

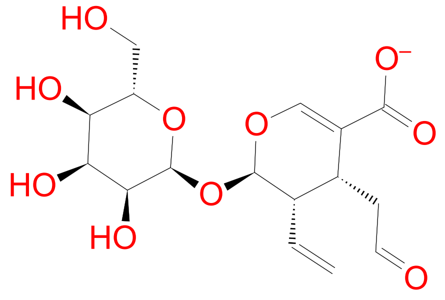

title P137_Secologanic ac

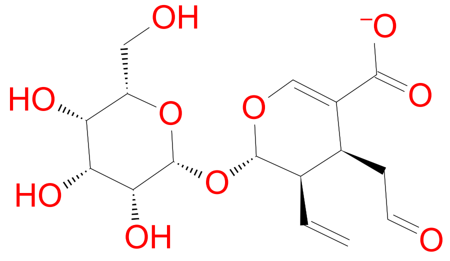

title P137_Secologanic ac

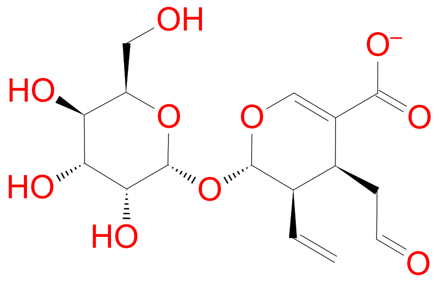

title P137_Secologanic ac

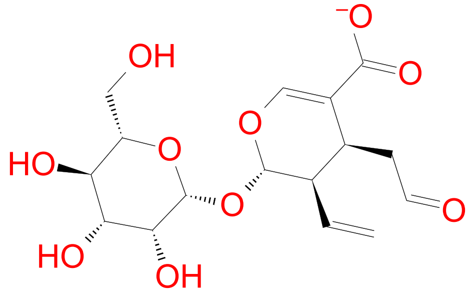

title P137_Secologanic ac

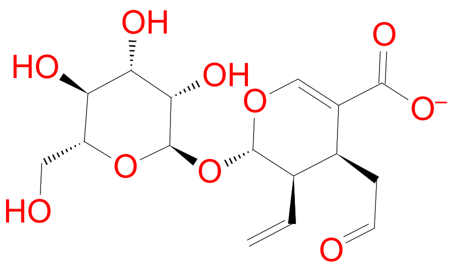

title P137_Secologanic ac

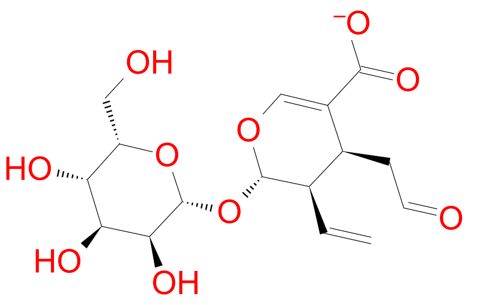

title P137_Secologanic ac

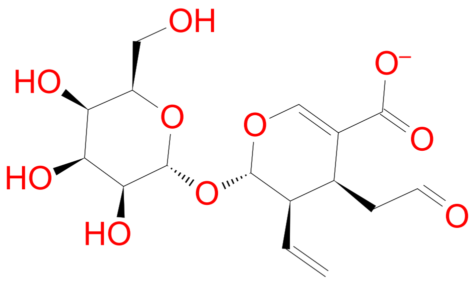

title P137_Secologanic ac

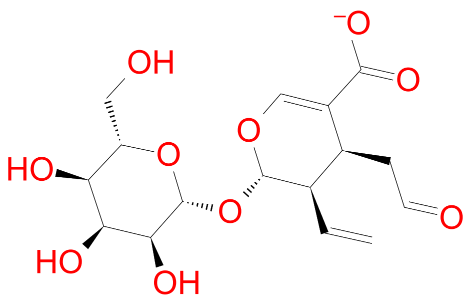

title P137_Secologanic ac

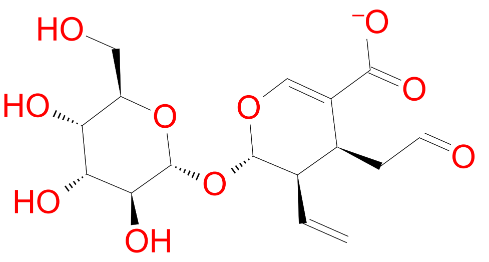

title P137_Secologanic ac

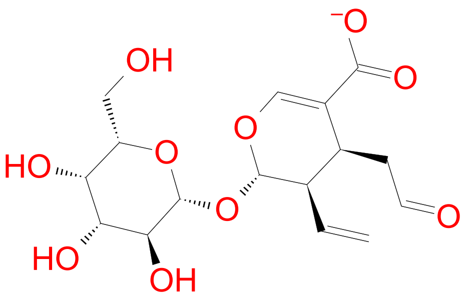

title P137_Secologanic ac

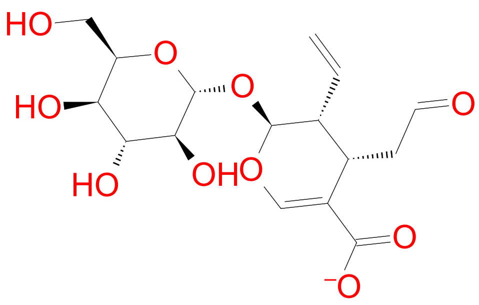

title P137_Secologanic ac

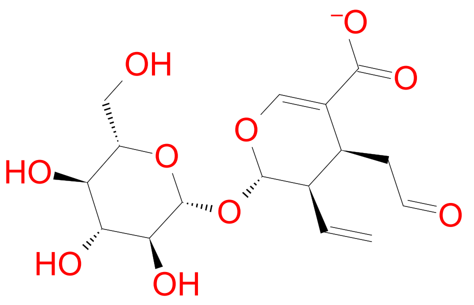

title P137_Secologanic ac

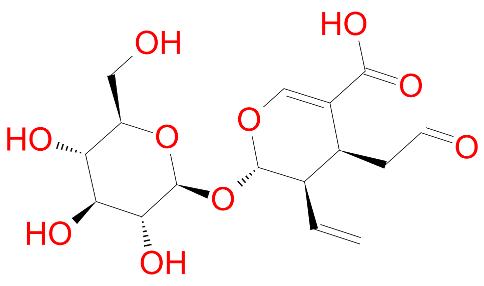

title P137_Secologanic ac

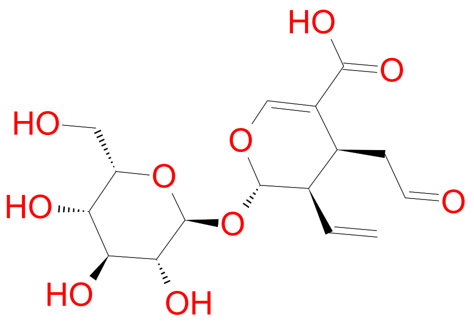

title P137_Secologanic ac

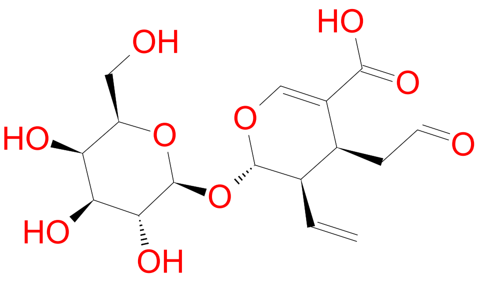

title P137_Secologanic ac

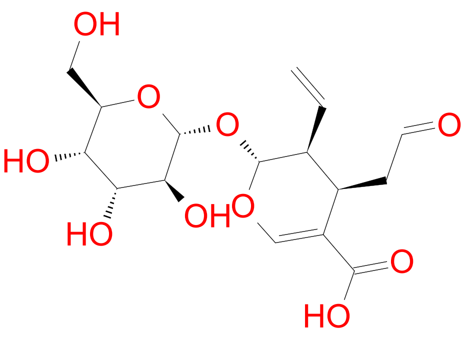

title P137_Secologanic ac

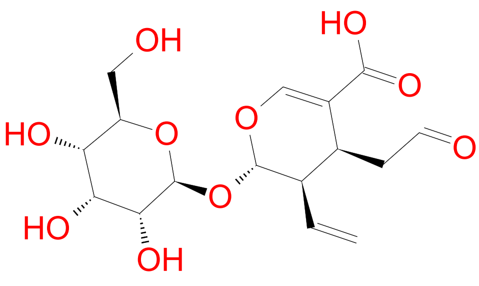

title P137_Secologanic ac

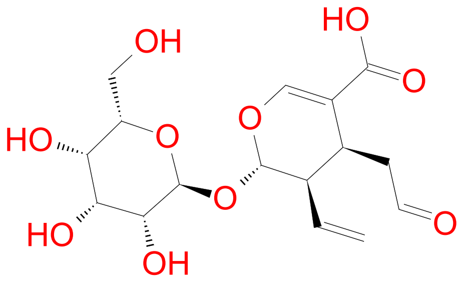

title P137_Secologanic ac

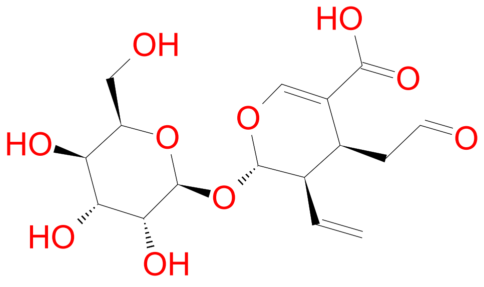

title P137_Secologanic ac

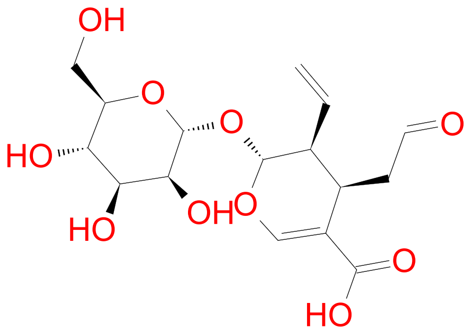

title P137_Secologanic ac

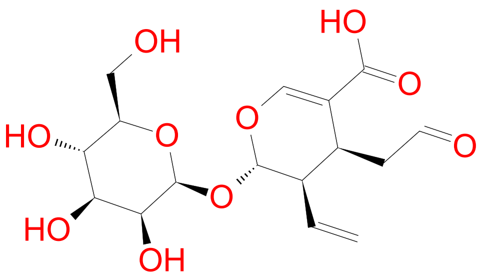

title P137_Secologanic ac

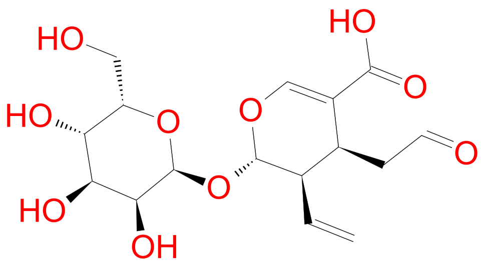

title P137_Secologanic ac

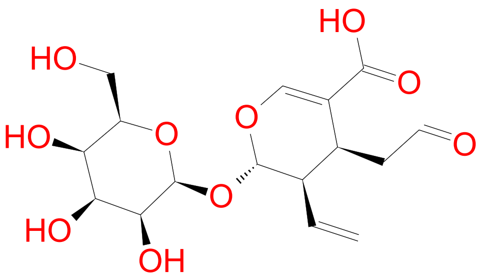

title P137_Secologanic ac

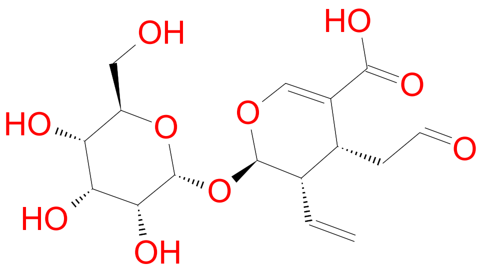

title P137_Secologanic ac

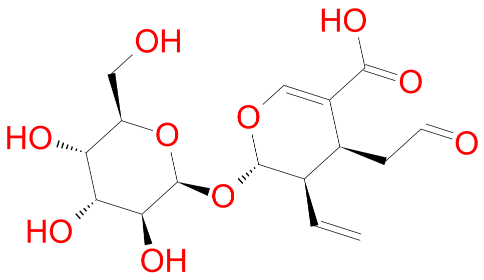

title P137_Secologanic ac

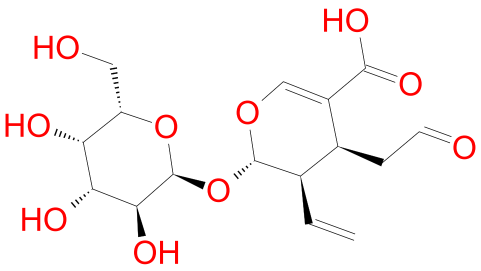

title P137_Secologanic ac

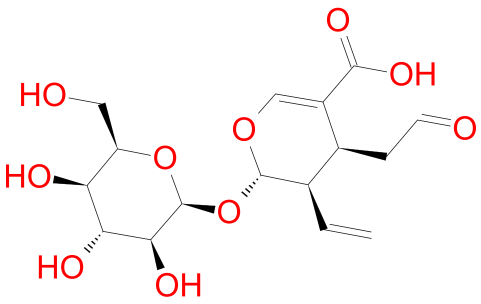

title P137_Secologanic ac

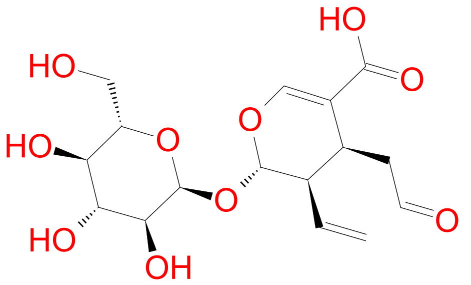

title P137_Secologanic ac

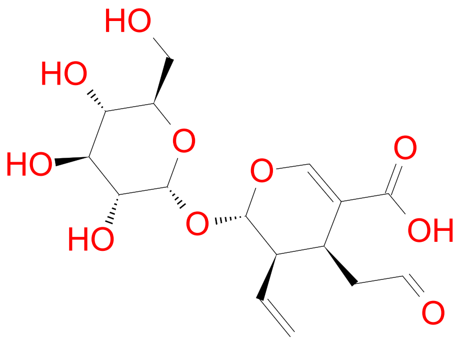

title P137_Secologanic ac

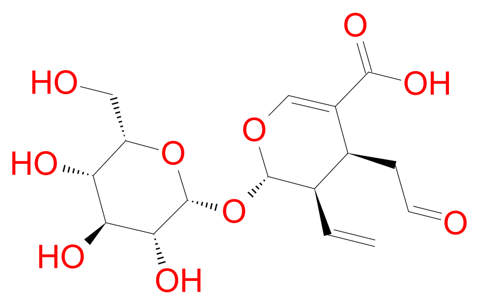

title P137_Secologanic ac

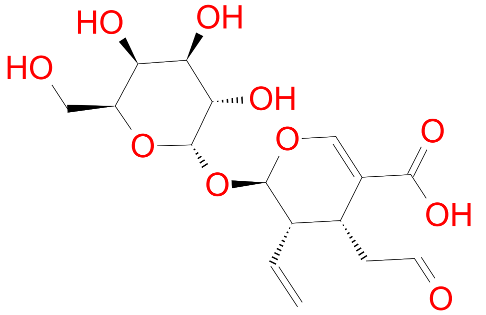

title P137_Secologanic ac

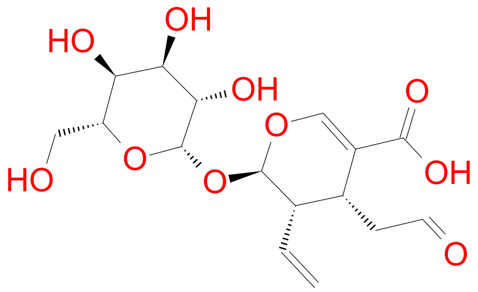

title P137_Secologanic ac

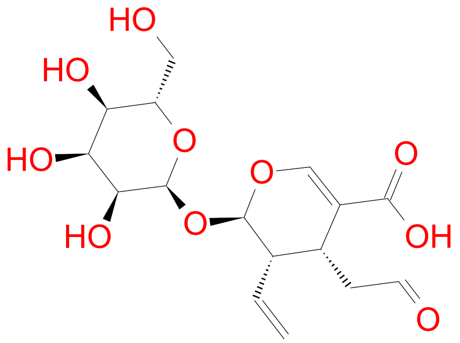

title P137_Secologanic ac

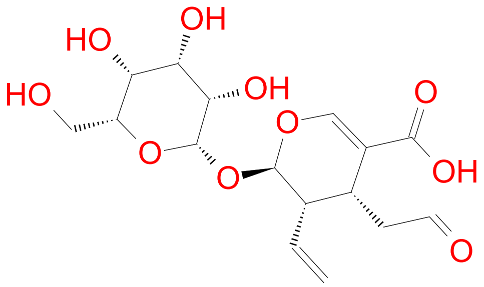

title P137_Secologanic ac

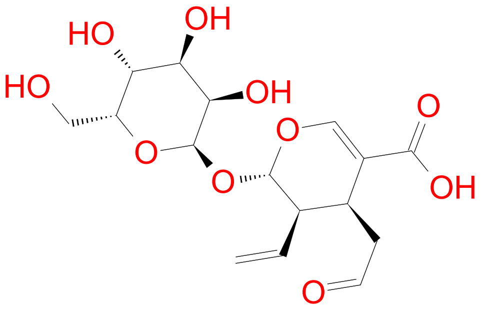

title P137_Secologanic ac

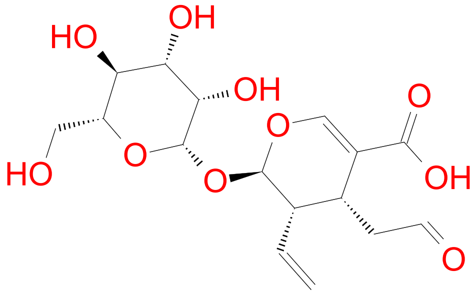

title P137_Secologanic ac

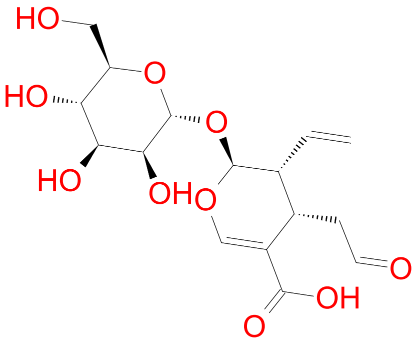

title P137_Secologanic ac

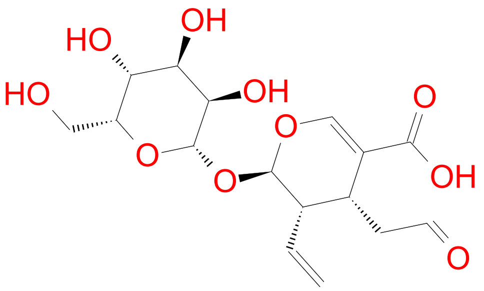

title P137_Secologanic ac

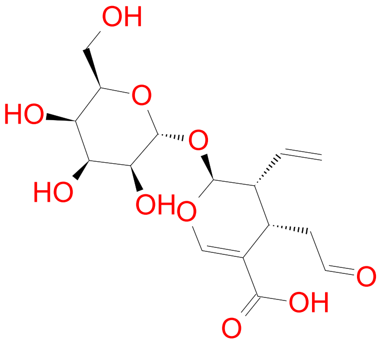

title P137_Secologanic ac

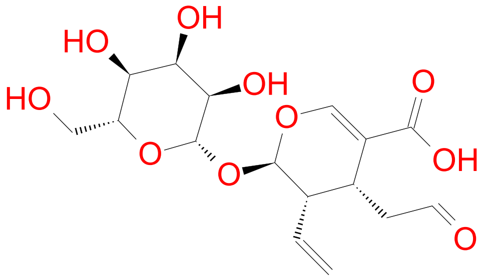

title P137_Secologanic ac

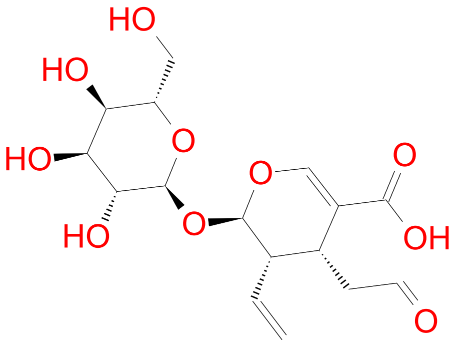

title P137_Secologanic ac

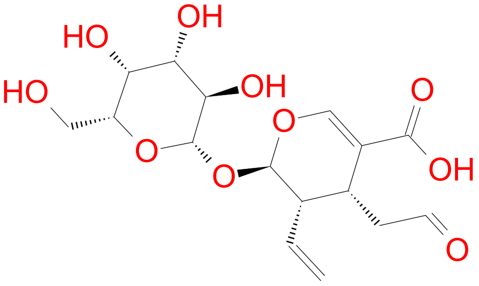

title P137_Secologanic ac

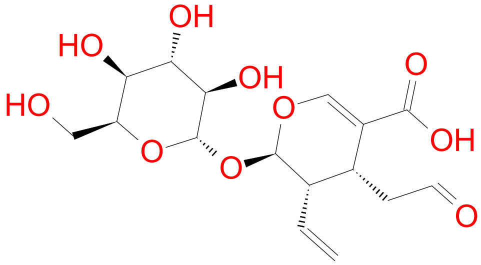

title P137_Secologanic ac

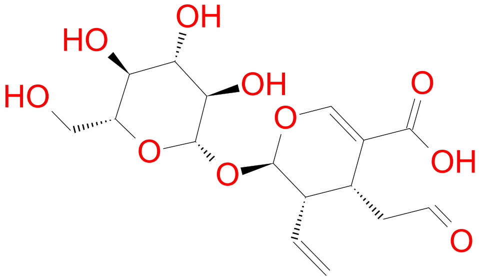

title P137_Secologanic ac

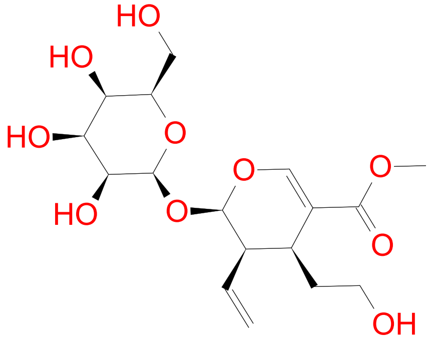

title P138_Secologanol

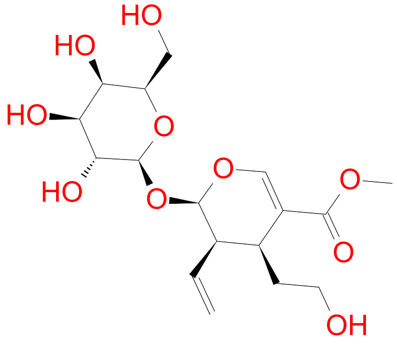

title P138_Secologanol

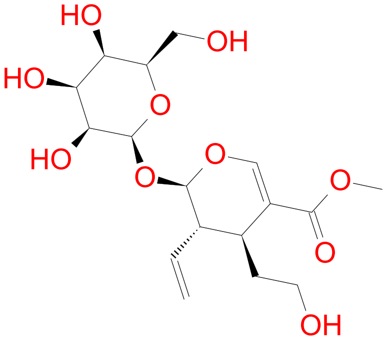

title P138_Secologanol

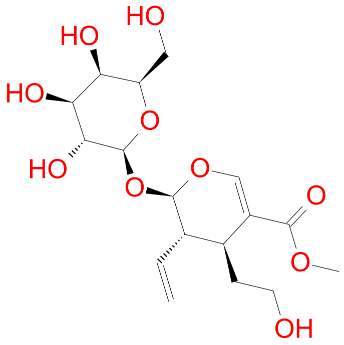

title P138_Secologanol

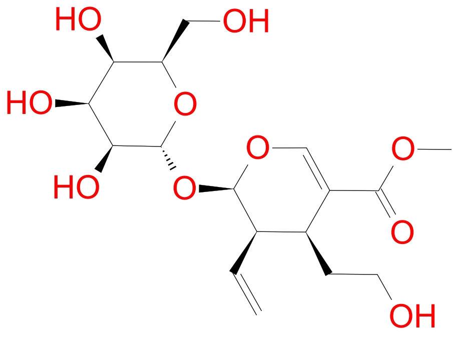

title P138_Secologanol

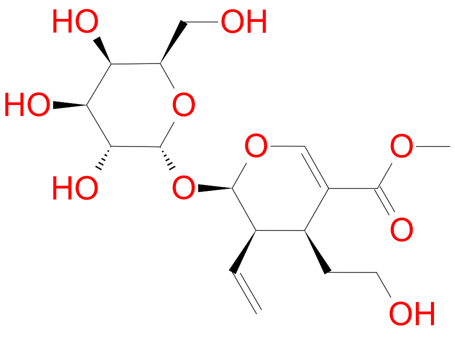

title P138_Secologanol

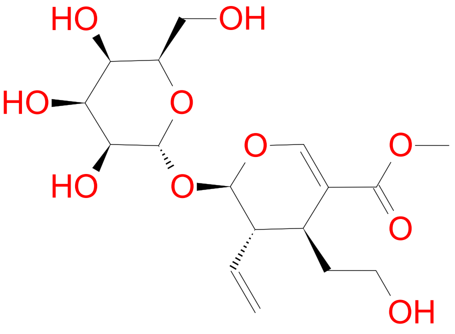

title P138_Secologanol

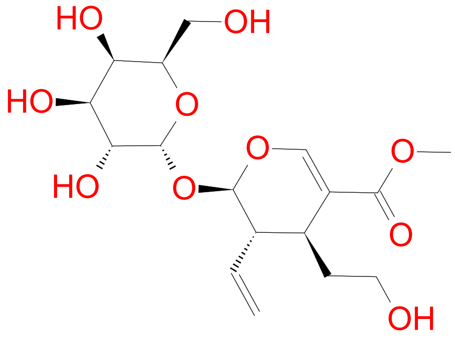

title P138_Secologanol

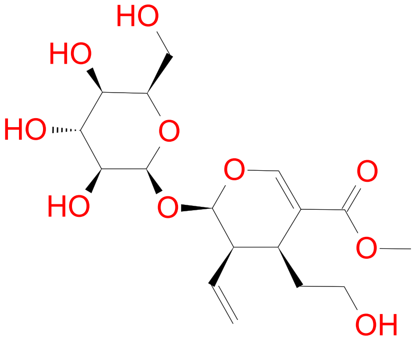

title P138_Secologanol

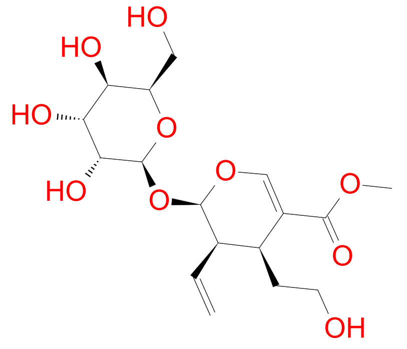
title P138_Secologanol

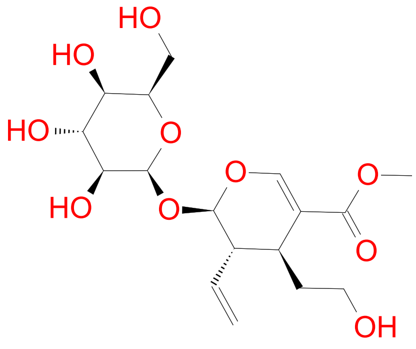
title P138_Secologanol

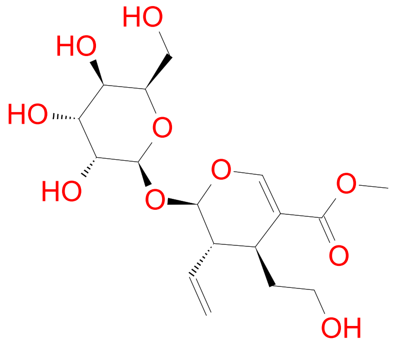
title P138_Secologanol

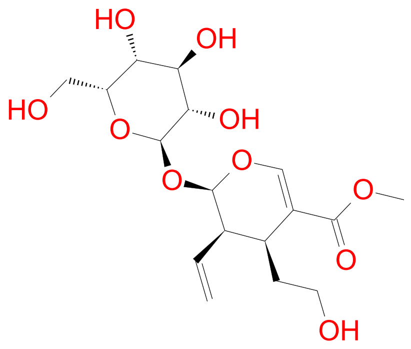
title P138_Secologanol

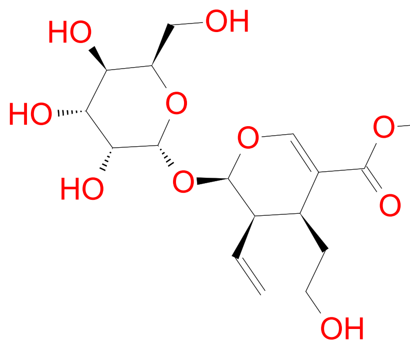
title P138_Secologanol

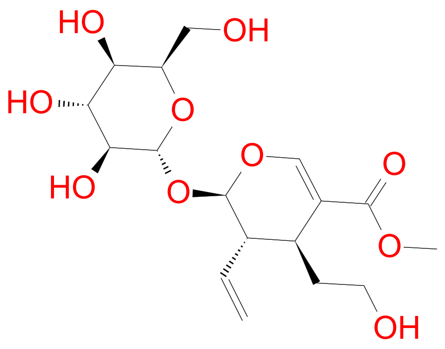
title P138_Secologanol

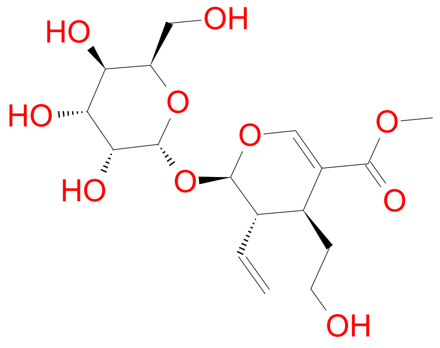
title P138_Secologanol

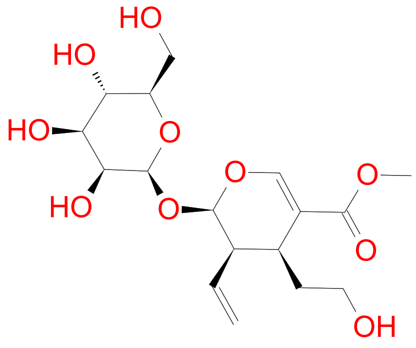
title P138_Secologanol

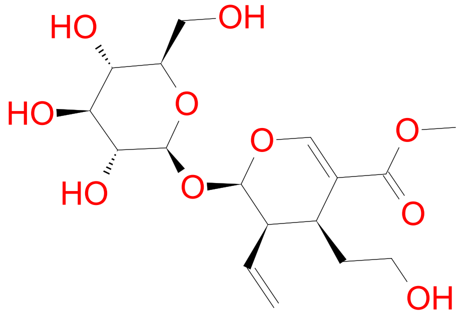
title P138_Secologanol

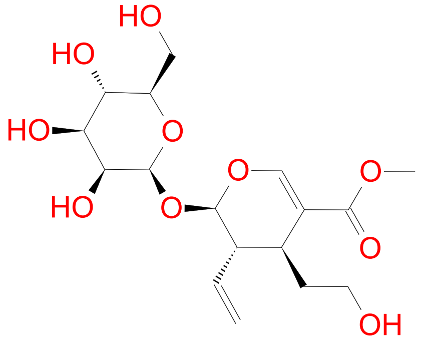
title P138_Secologanol

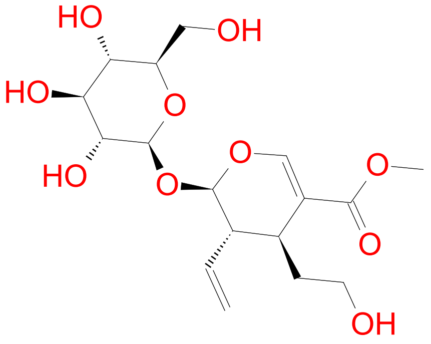
title P138_Secologanol

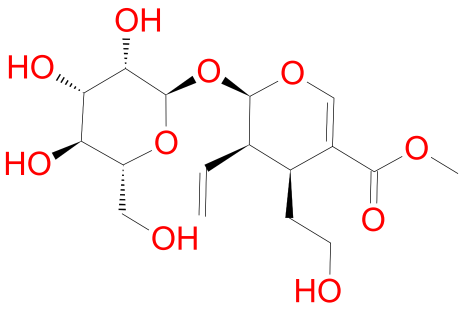
title P138_Secologanol

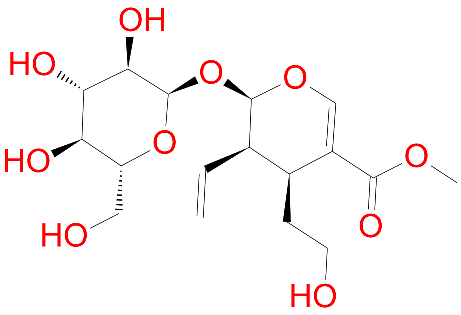

title P138_Secologanol

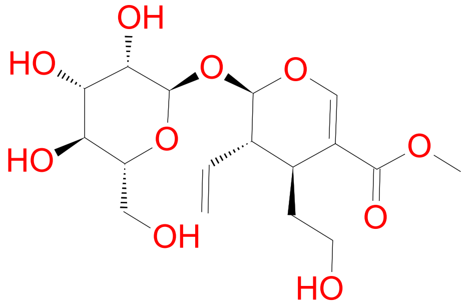

title P138_Secologanol

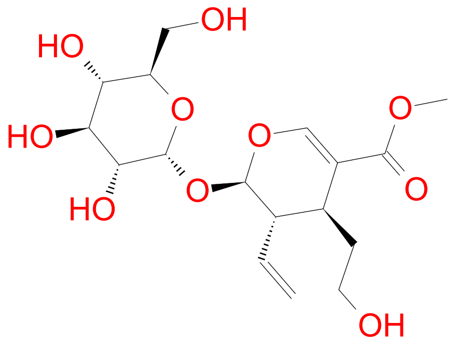

title P138_Secologanol

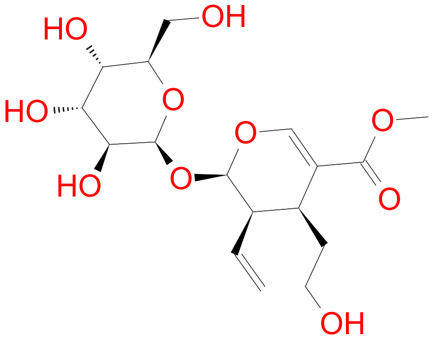

title P138_Secologanol

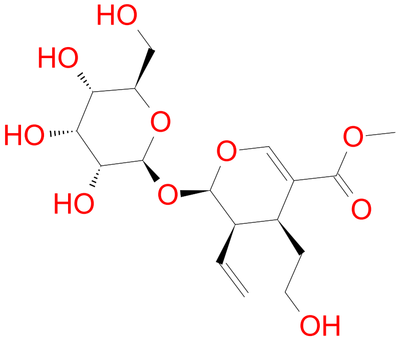

title P138_Secologanol

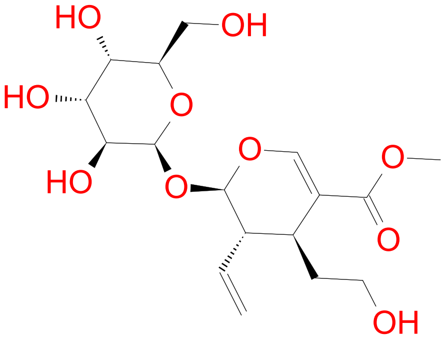

title P138_Secologanol

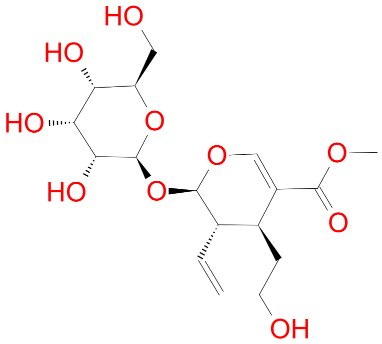

title P138_Secologanol

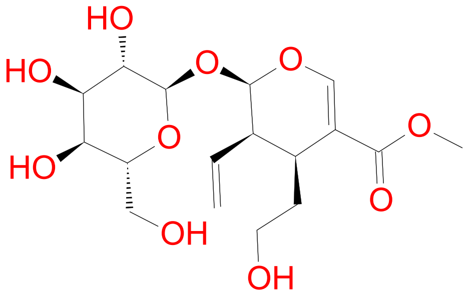

title P138_Secologanol

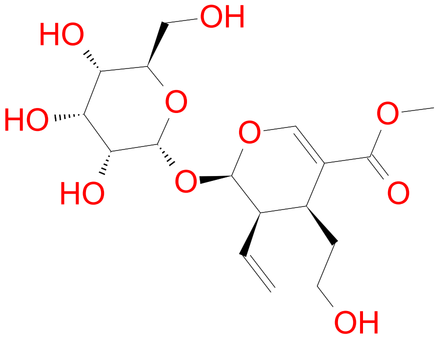

title P138_Secologanol

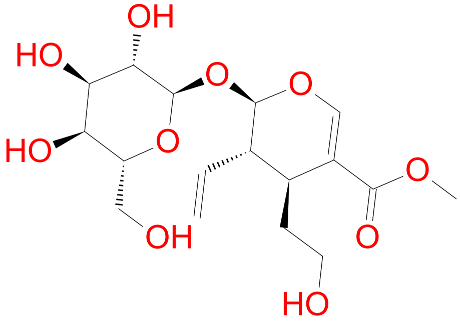

title P138_Secologanol

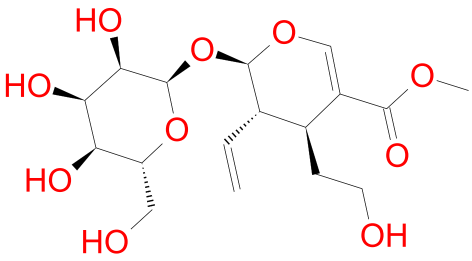

title P138_Secologanol

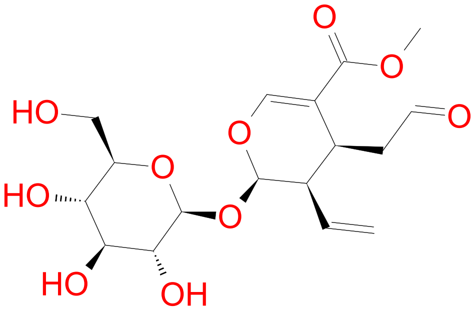

title P140_Secologanin

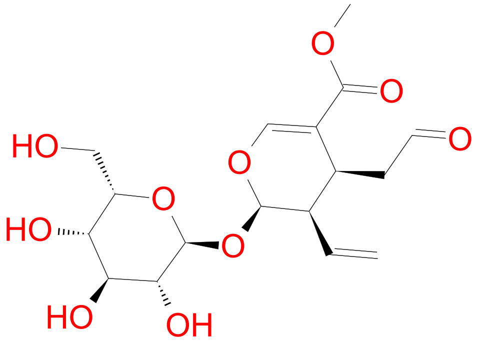

title P140_Secologanin

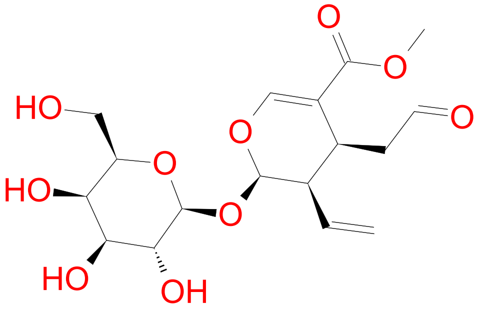

title P140_Secologanin

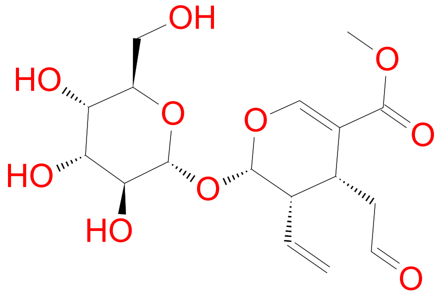

title P140_Secologanin

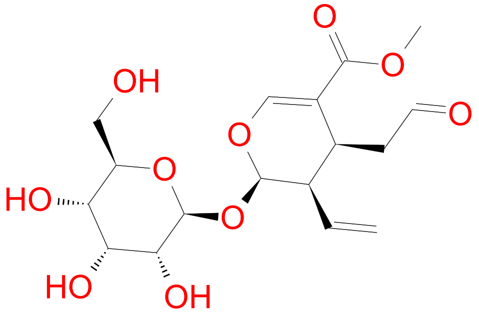

title P140_Secologanin

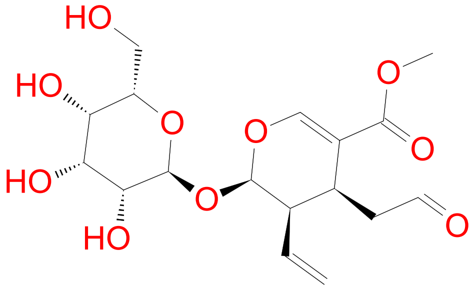

title P140_Secologanin

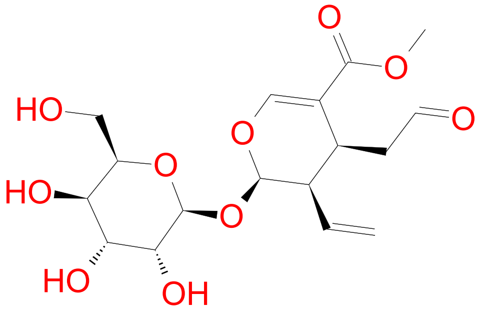

title P140_Secologanin

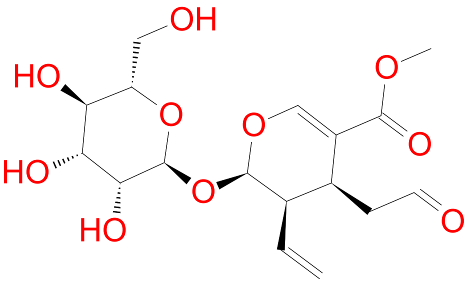

title P140_Secologanin

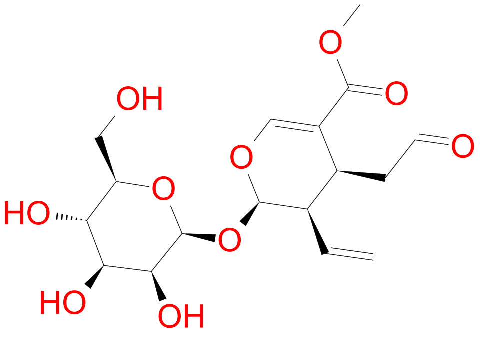

title P140_Secologanin

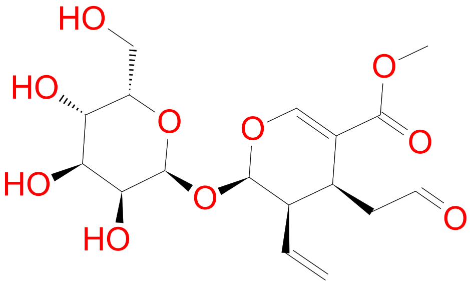

title P140_Secologanin

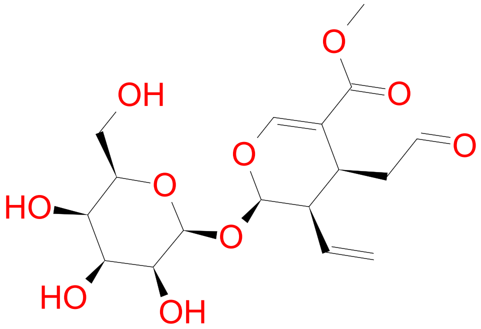

title P140_Secologanin

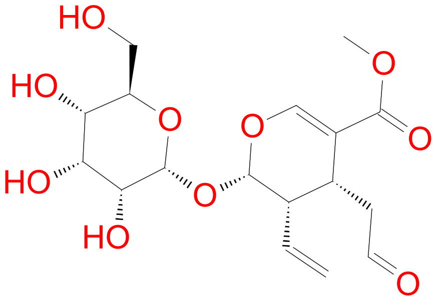

title P140_Secologanin

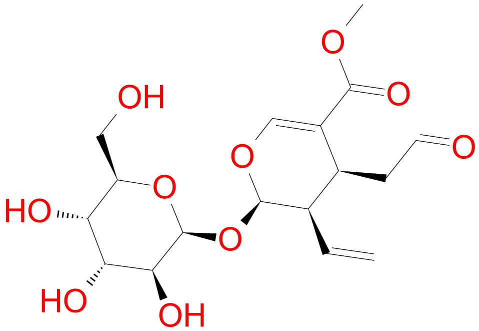

title P140_Secologanin

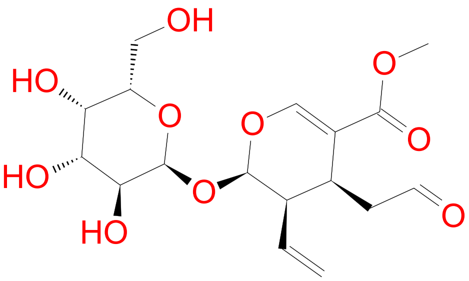

title P140_Secologanin

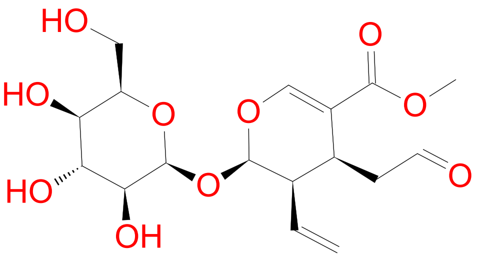

title P140_Secologanin

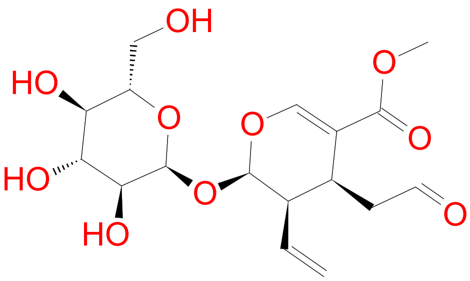

title P140_Secologanin

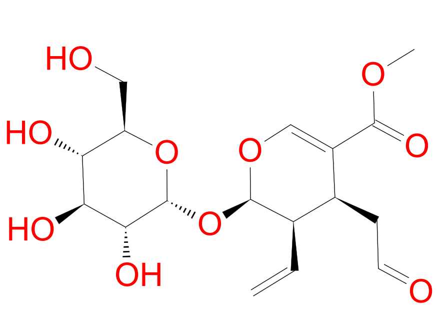

title P140_Secologanin

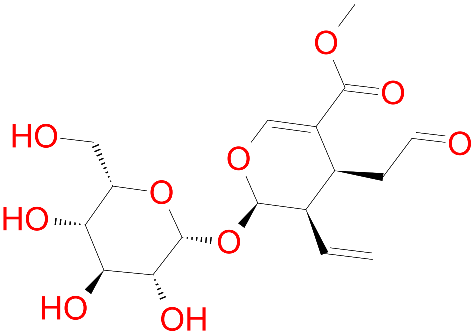

title P140_Secologanin

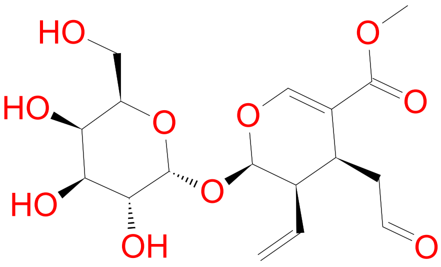

title P140_Secologanin

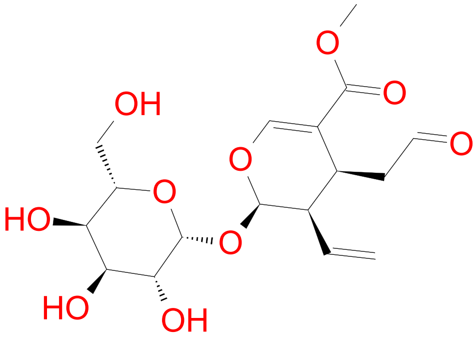

title P140_Secologanin

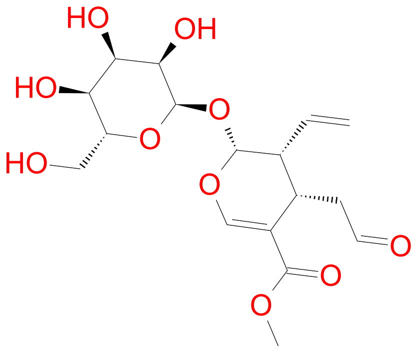

title P140_Secologanin

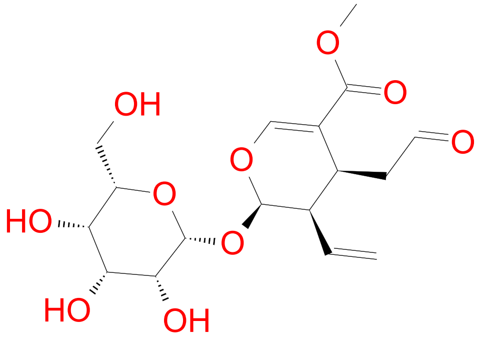

title P140_Secologanin

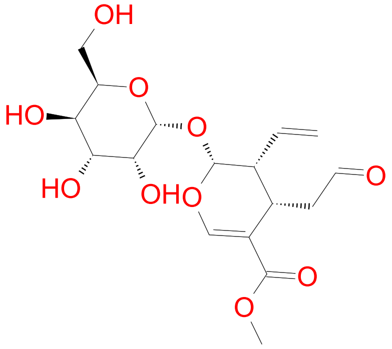

title P140_Secologanin

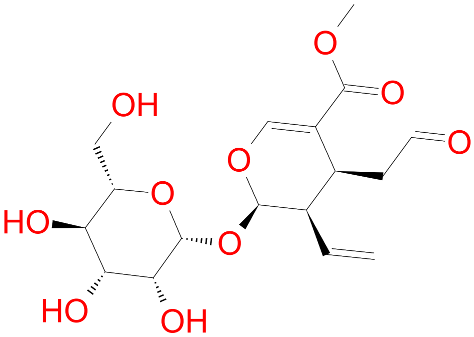

title P140_Secologanin

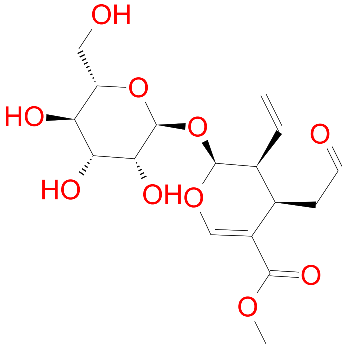

title P140_Secologanin

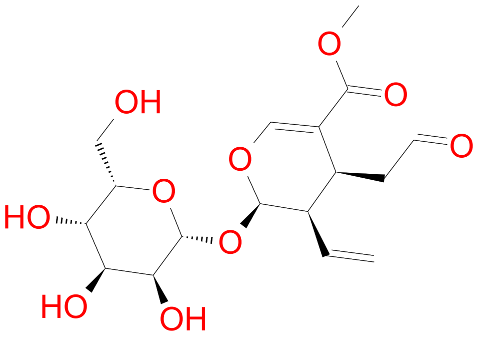

title P140_Secologanin

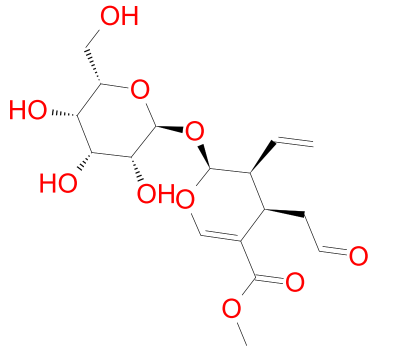

title P140_Secologanin

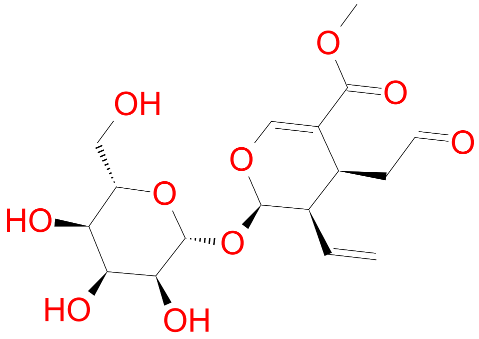

title P140_Secologanin

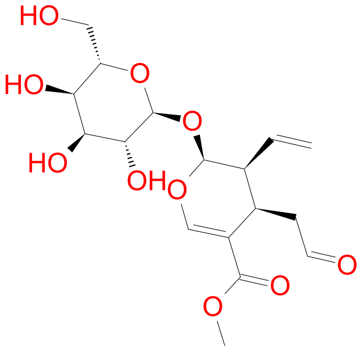

title P140_Secologanin

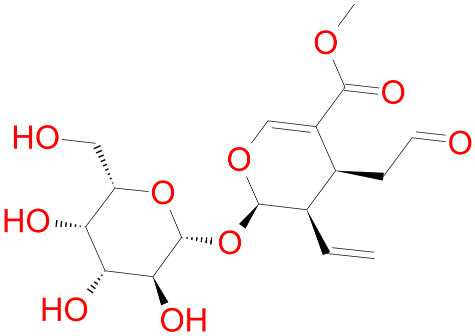

title P140_Secologanin

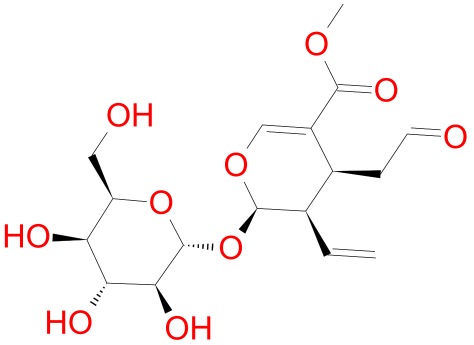

title P140_Secologanin

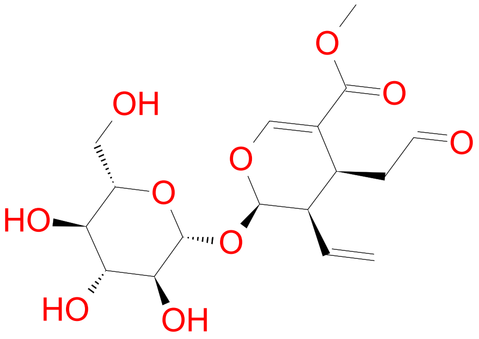

title P140_Secologanin

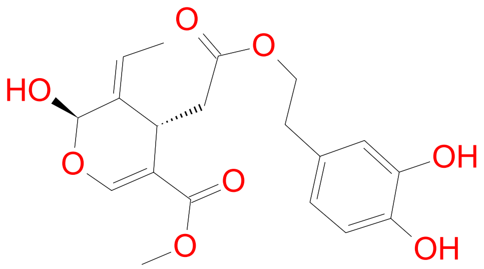

title P141_Oleuropein agl

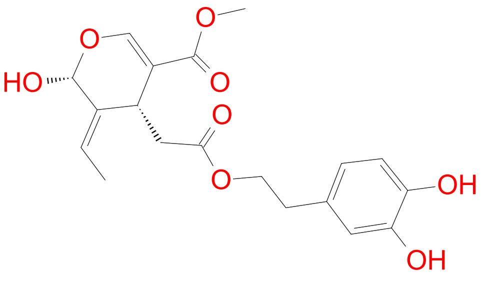

title P141_Oleuropein agl

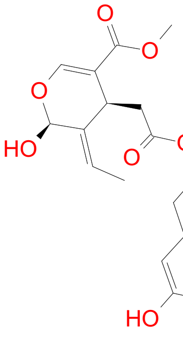

title P141_Oleuropein agl

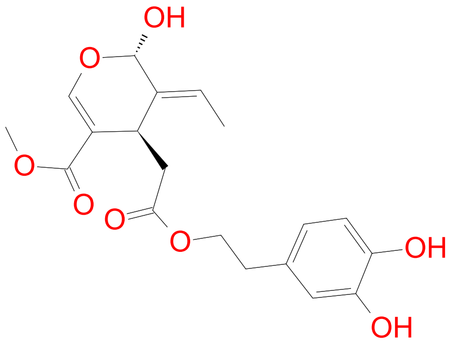

title P141_Oleuropein agl

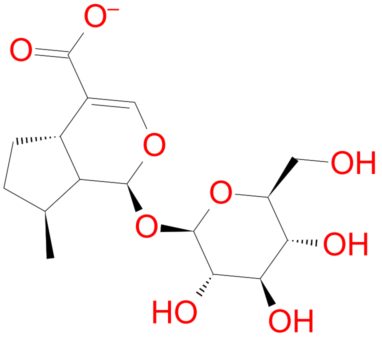

title P142_7-Deoxylogani

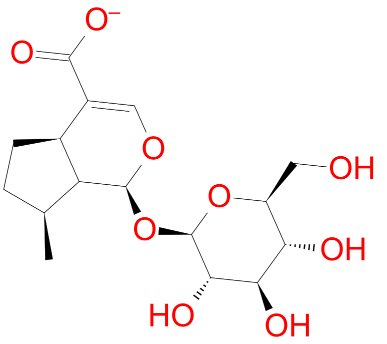

title P142_7-Deoxylogani

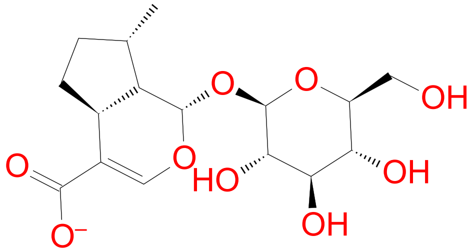

title P142_7-Deoxylogani

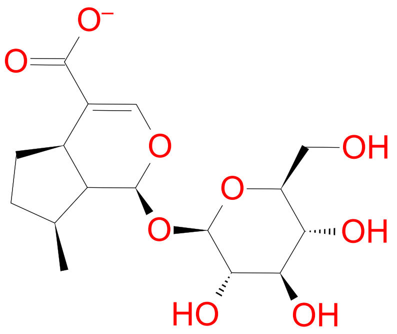

title P142_7-Deoxylogani

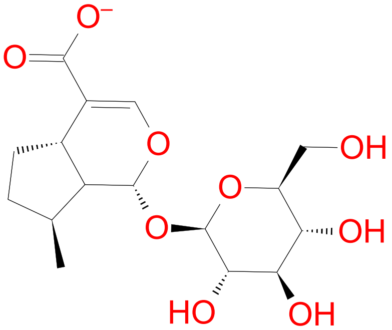

title P142_7-Deoxylogani

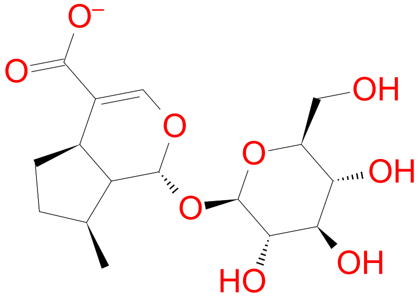

title P142_7-Deoxylogani

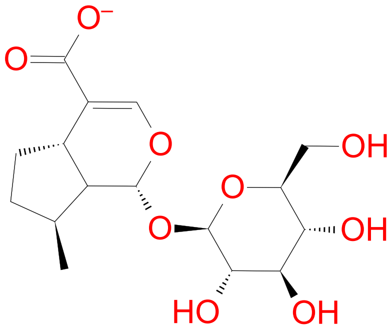

title P142_7-Deoxylogani

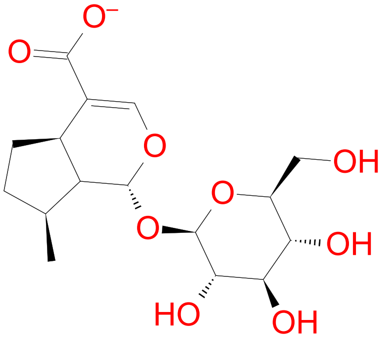

title P142_7-Deoxylogani

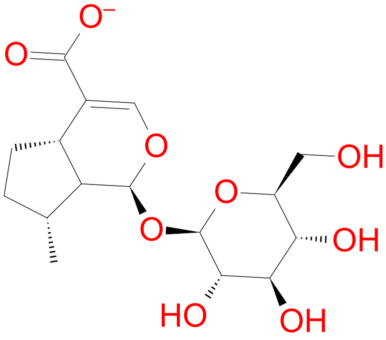

title P142_7-Deoxylogani

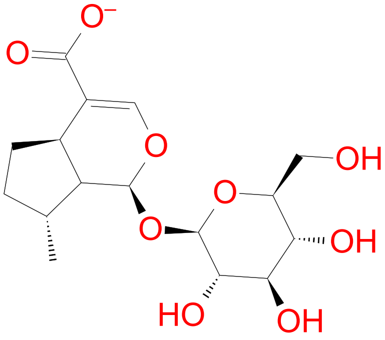

title P142_7-Deoxylogani

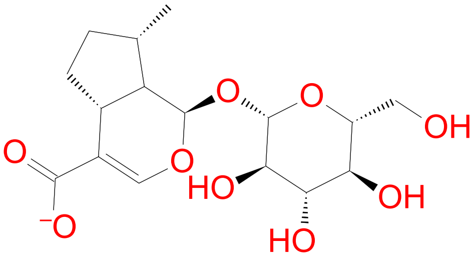

title P142_7-Deoxylogani

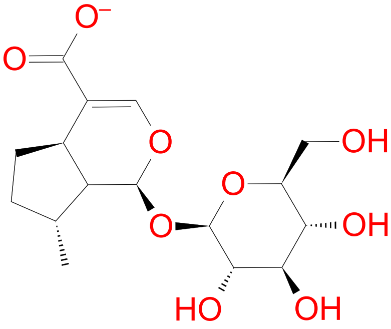

title P142_7-Deoxylogani

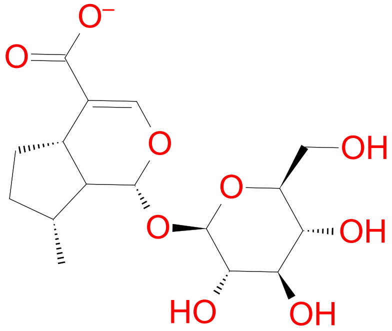

title P142_7-Deoxylogani

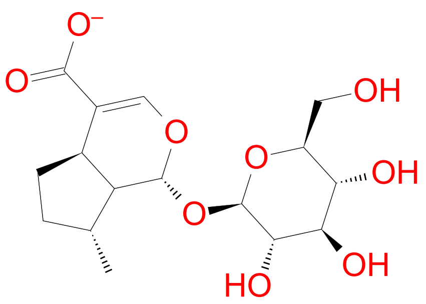

title P142_7-Deoxylogani

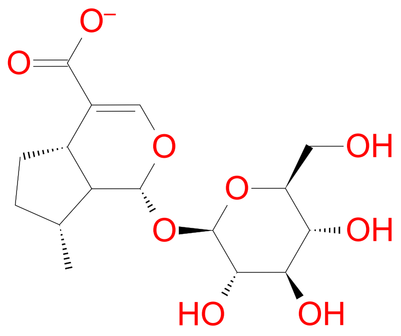

title P142_7-Deoxylogani

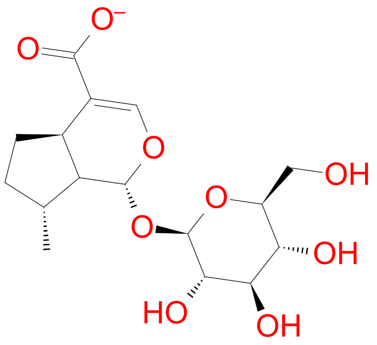

title P142_7-Deoxylogani

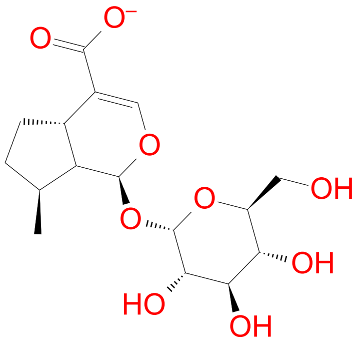

title P142_7-Deoxylogani

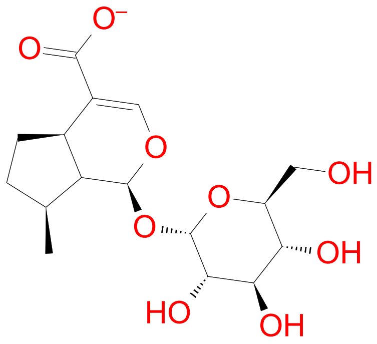

title P142_7-Deoxylogani

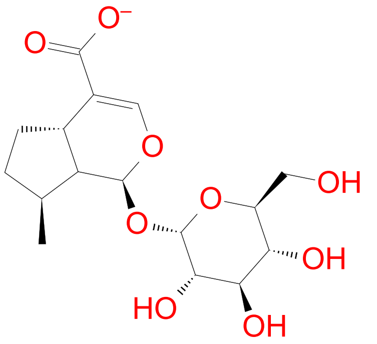

title P142_7-Deoxylogani

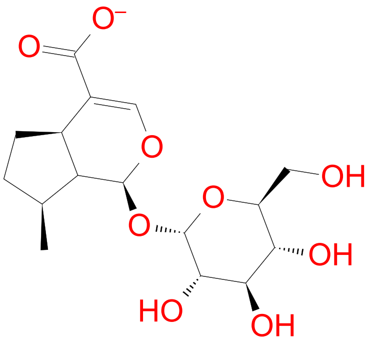

title P142_7-Deoxylogani

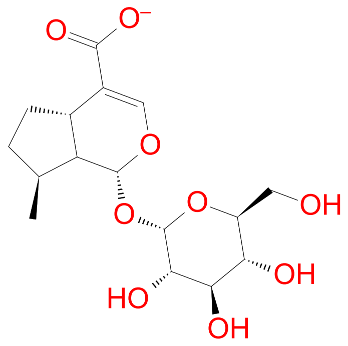

title P142_7-Deoxylogani

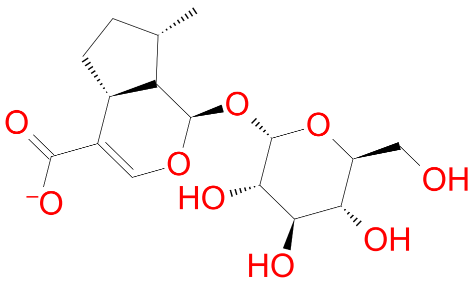

title P142_7-Deoxylogani

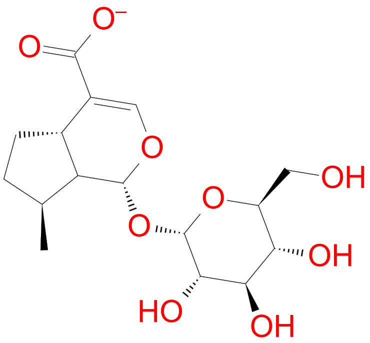

title P142_7-Deoxylogani

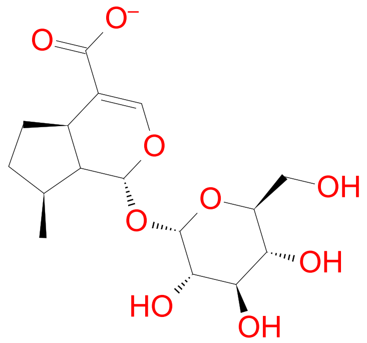

title P142_7-Deoxylogani

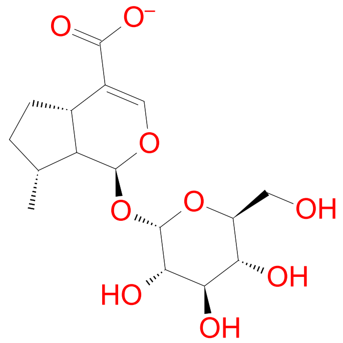

title P142_7-Deoxylogani

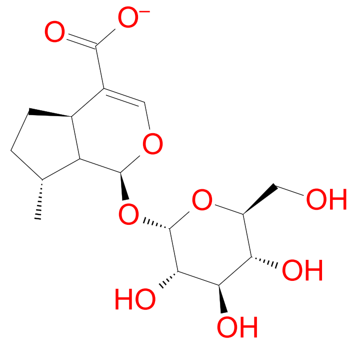

title P142_7-Deoxylogani

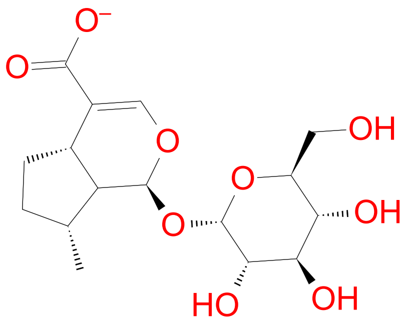

title P142_7-Deoxylogani

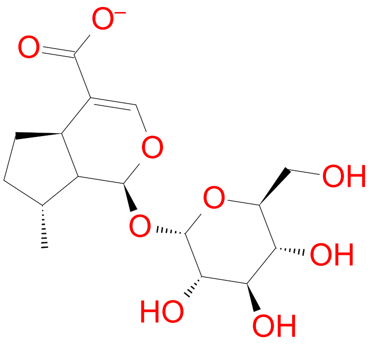

title P142_7-Deoxylogani

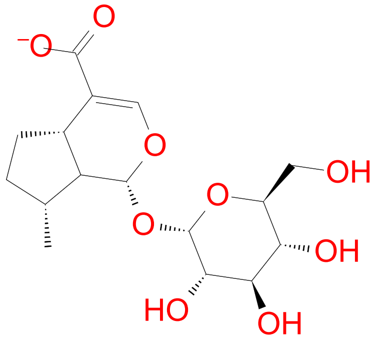

title P142_7-Deoxylogani

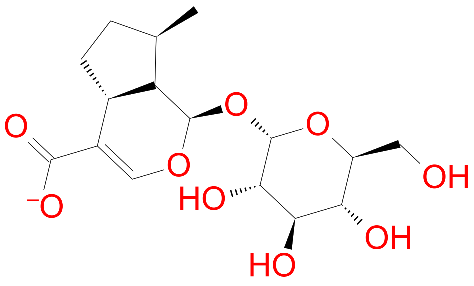

title P142_7-Deoxylogani

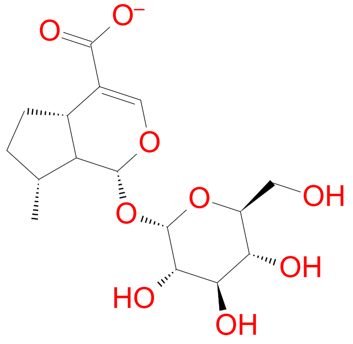

title P142_7-Deoxylogani

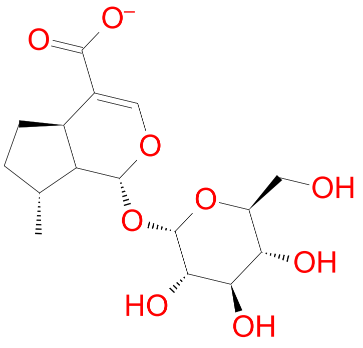

title P142_7-Deoxylogani

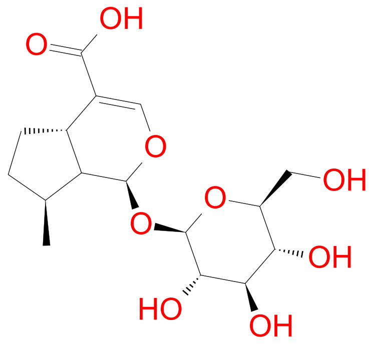

title P142_7-Deoxylogani

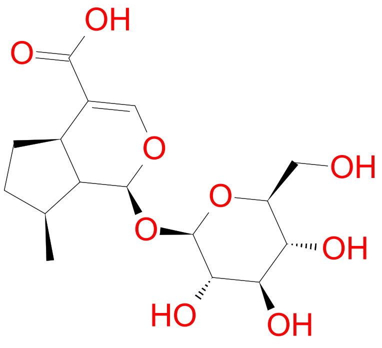

title P142_7-Deoxylogani

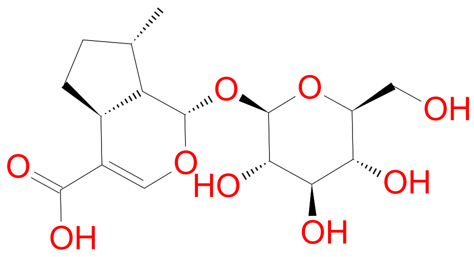

title P142_7-Deoxylogani

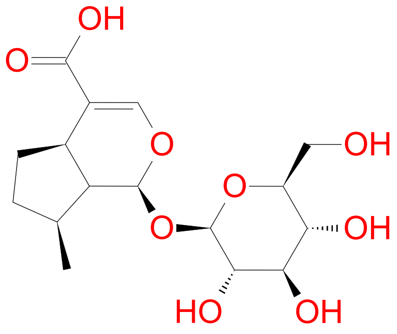

title P142_7-Deoxylogani

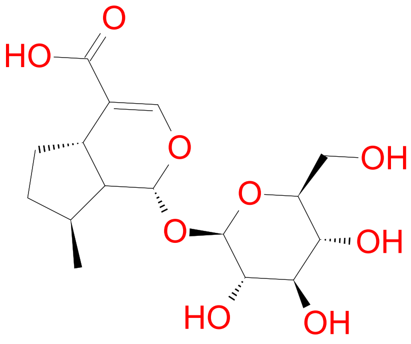

title P142_7-Deoxylogani

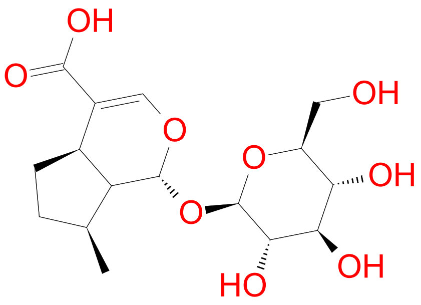

title P142_7-Deoxylogani

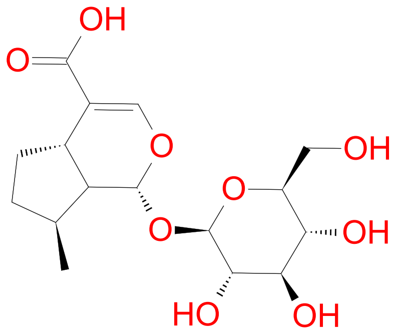

title P142_7-Deoxylogani

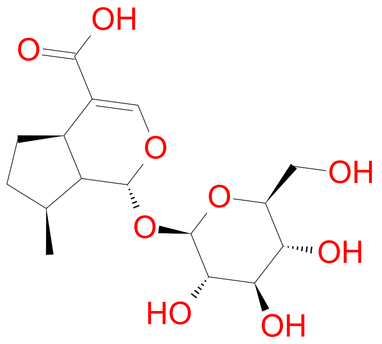

title P142_7-Deoxylogani

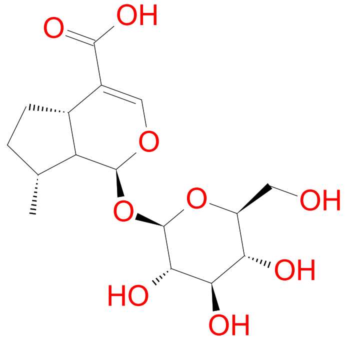

title P142_7-Deoxylogani

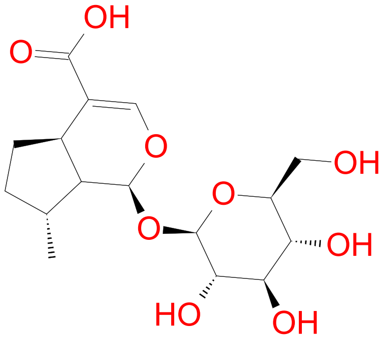

title P142_7-Deoxylogani

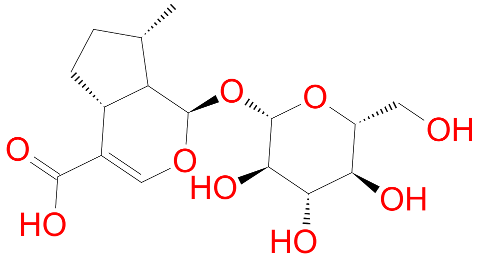

title P142_7-Deoxylogani

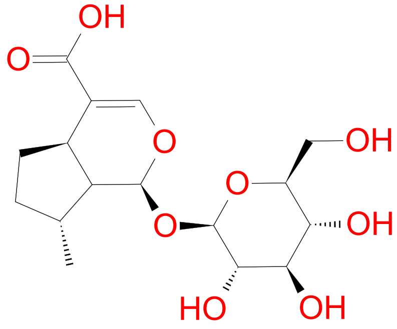

title P142_7-Deoxylogani

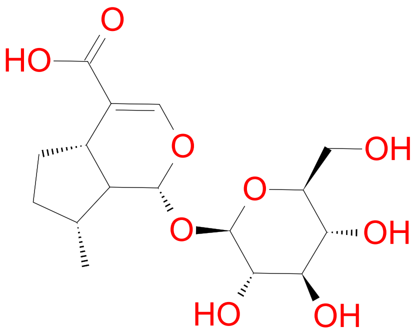

title P142_7-Deoxylogani

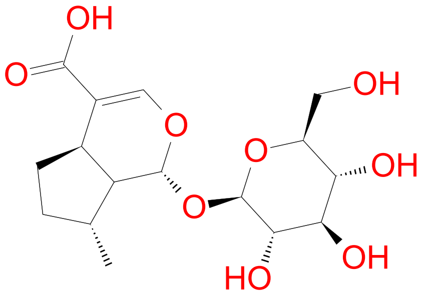

title P142_7-Deoxylogani

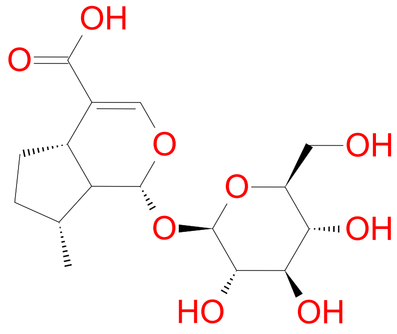

title P142_7-Deoxylogani

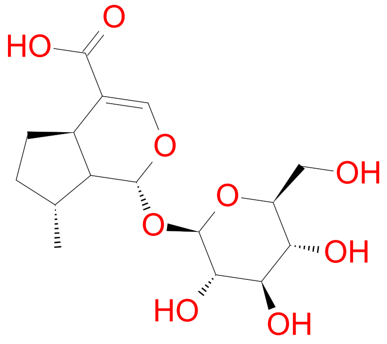

title P142_7-Deoxylogani

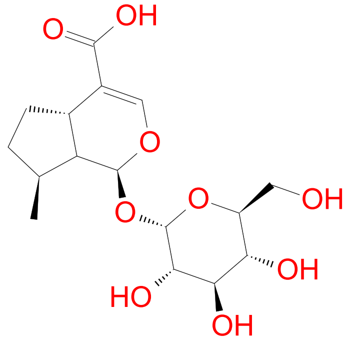

title P142_7-Deoxylogani

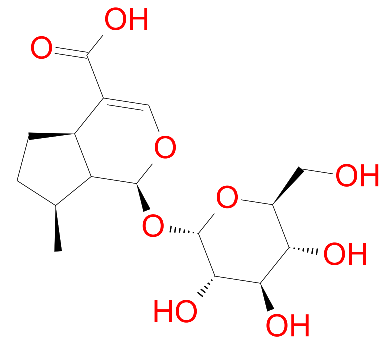

title P142_7-Deoxylogani

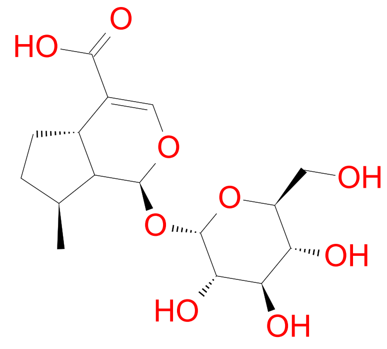

title P142_7-Deoxylogani

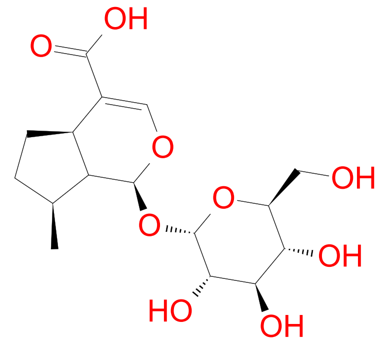

title P142_7-Deoxylogani

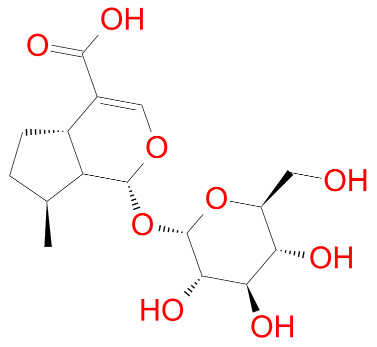

title P142_7-Deoxylogani

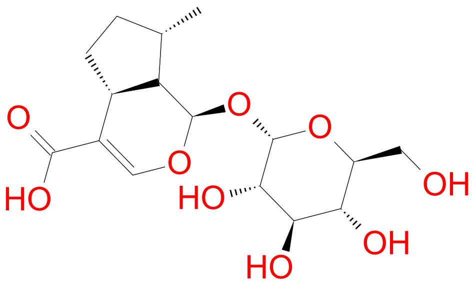

title P142_7-Deoxylogani

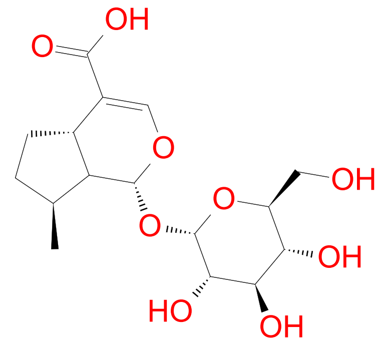

title P142_7-Deoxylogani

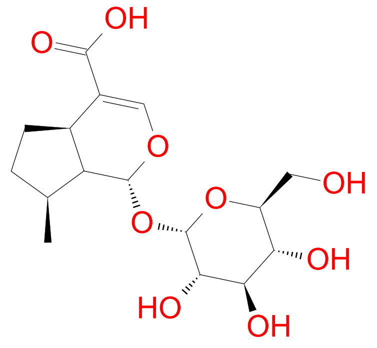

title P142_7-Deoxylogani

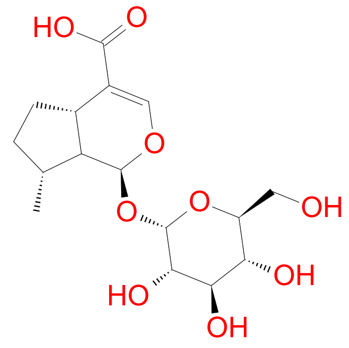

title P142_7-Deoxylogani

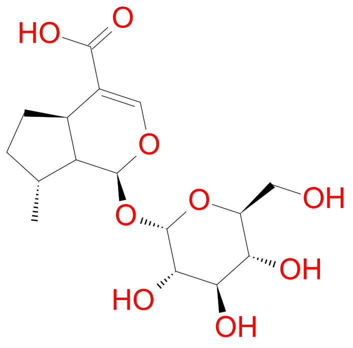

title P142_7-Deoxylogani

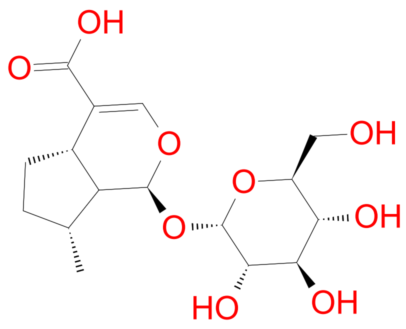

title P142_7-Deoxylogani

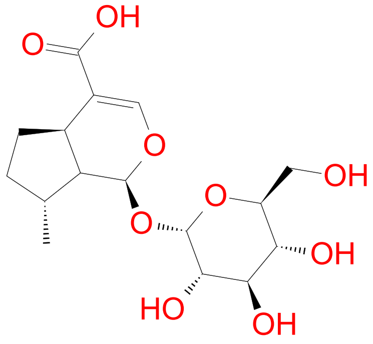

title P142_7-Deoxylogani

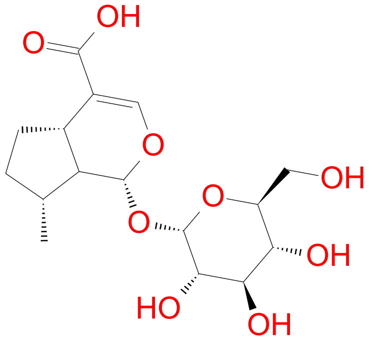

title P142_7-Deoxylogani

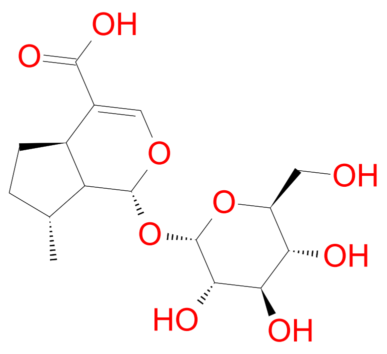

title P142_7-Deoxylogani

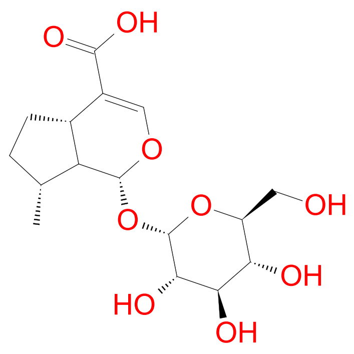

title P142_7-Deoxylogani

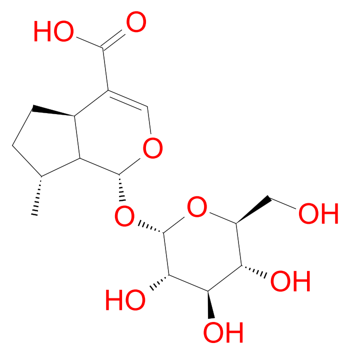

title P142_7-Deoxylogani

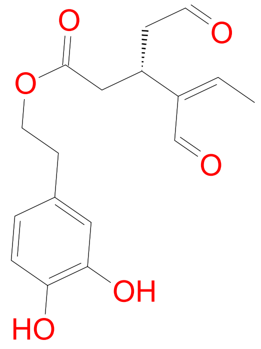

title P143_oleacein

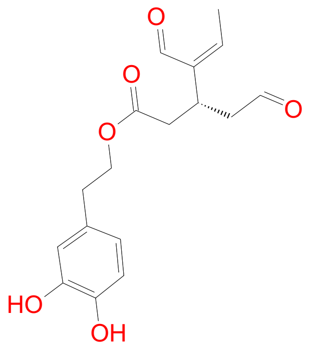

title P143_oleacein

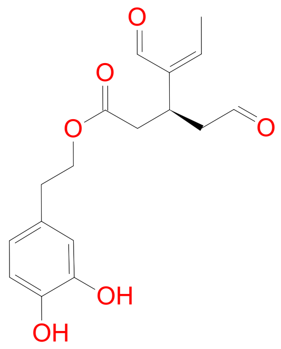

title P144_3,4-DHPEA-ED

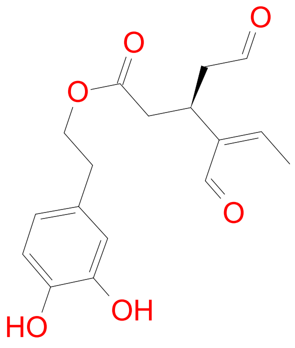

title P144_3,4-DHPEA-ED

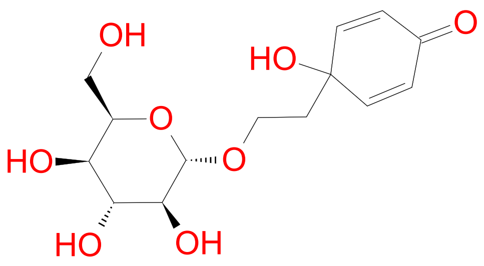

title P145_cornoside

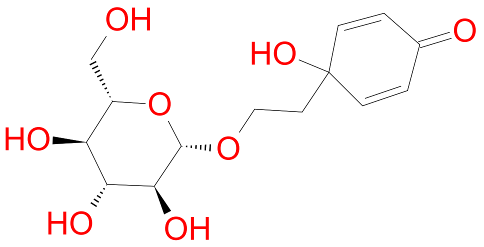

title P145_cornoside

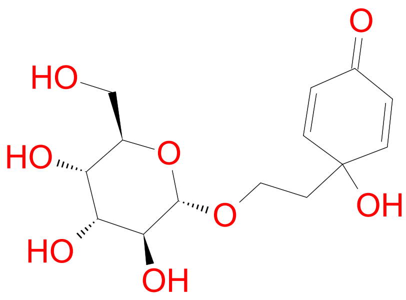

title P145_cornoside

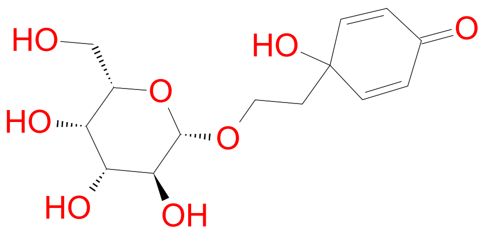

title P145_cornoside

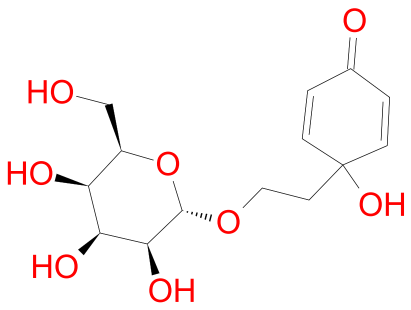

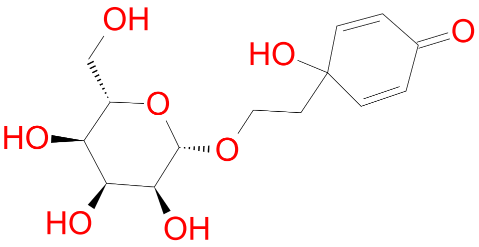

title P145_cornoside

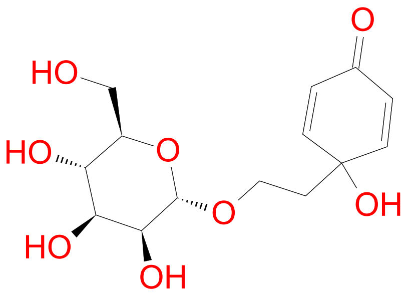

title P145_cornoside

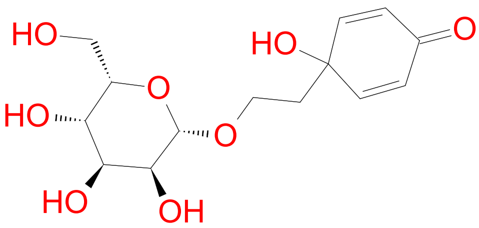

title P145_cornoside

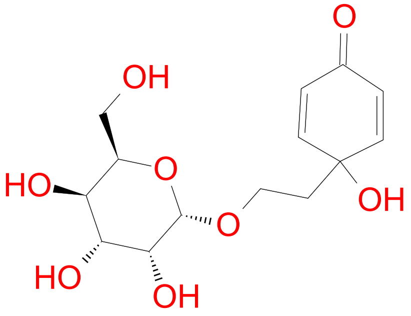

title P145_cornoside

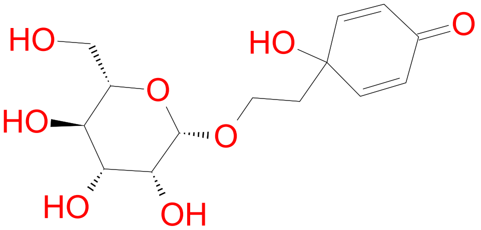

title P145_cornoside

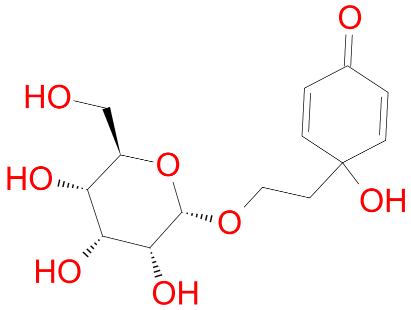

title P145_cornoside

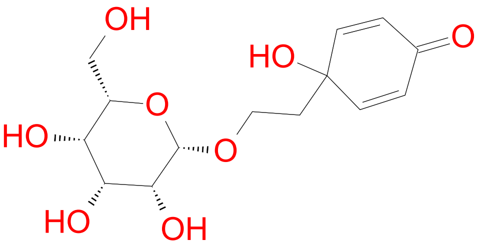

title P145_cornoside

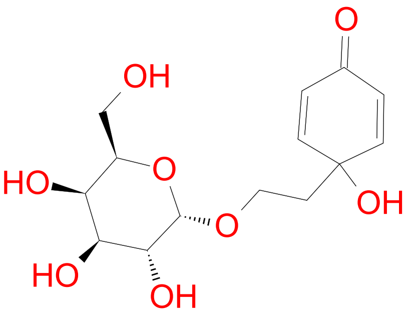

title P145_cornoside

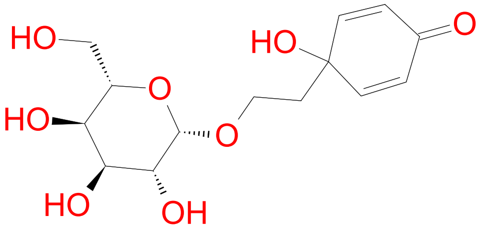

title P145_cornoside

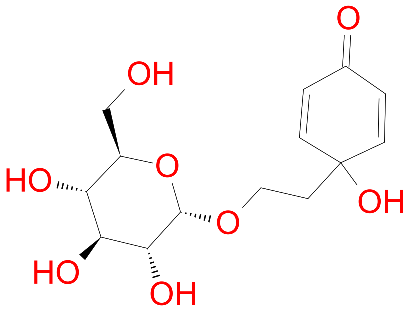

title P145_cornoside

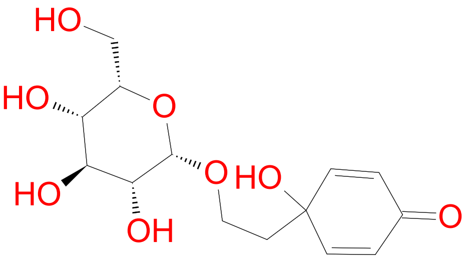

title P145_cornoside

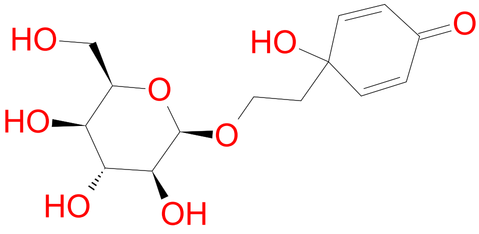

title P145_cornoside

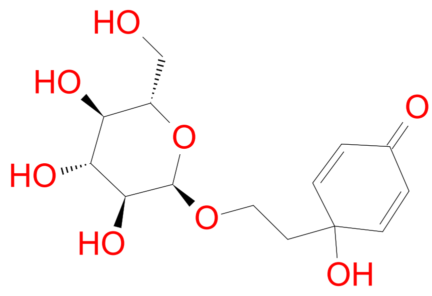

title P145_cornoside

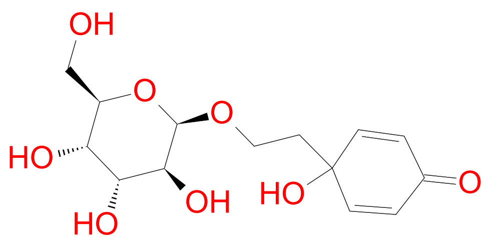

title P145_cornoside

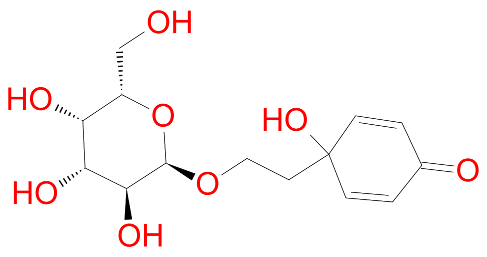

title P145_cornoside

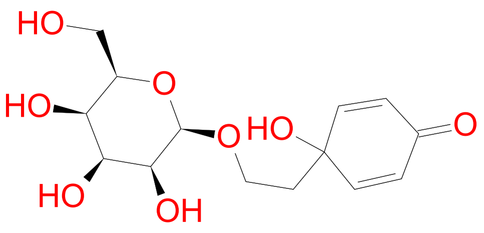

title P145_cornoside

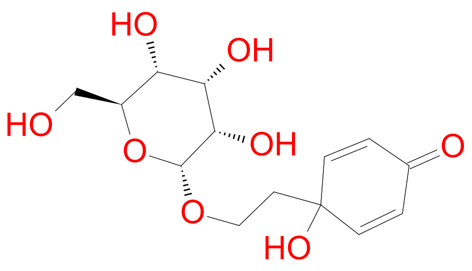

title P145_cornoside

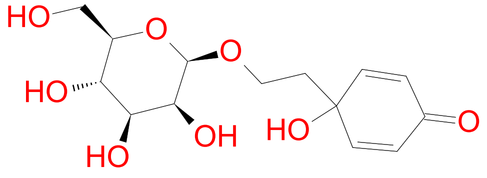

title P145_cornoside

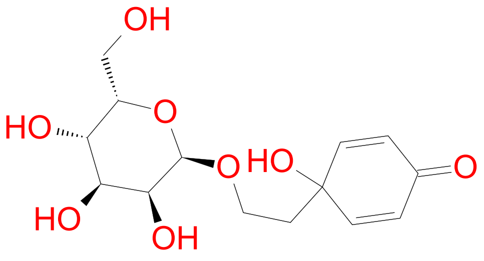

title P145_cornoside

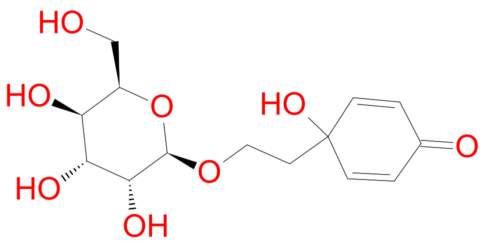

title P145_cornoside

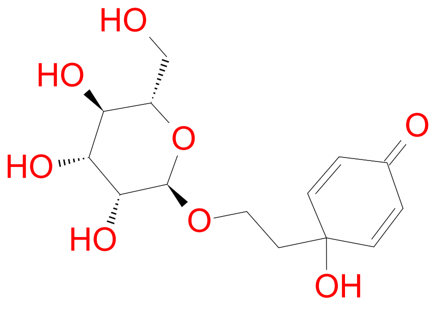

title P145_cornoside

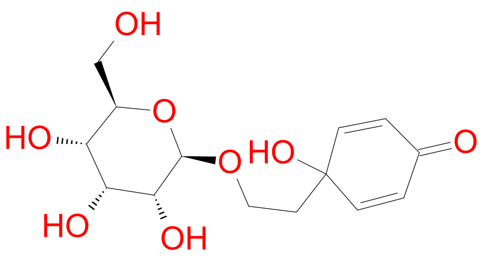

title P145_cornoside

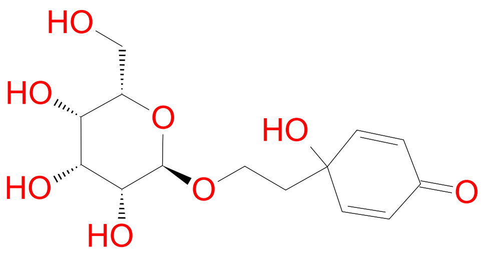

title P145_cornoside

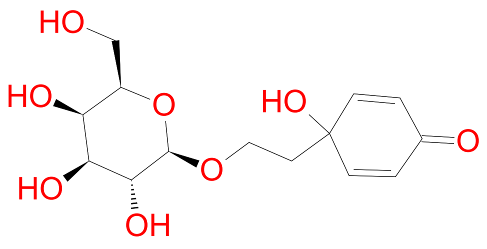

title P145_cornoside

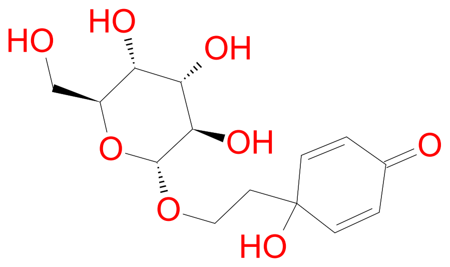

title P145_cornoside

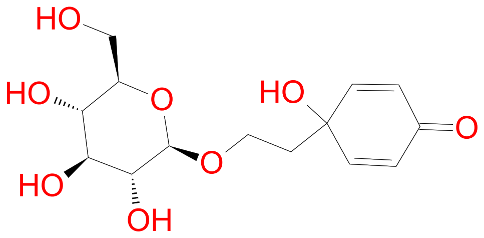

title P145_cornoside

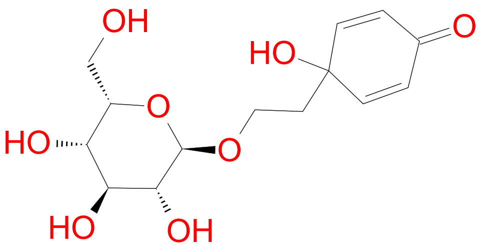

title P145_cornoside

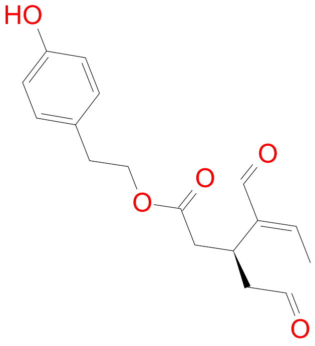

title P146_oleocanthal

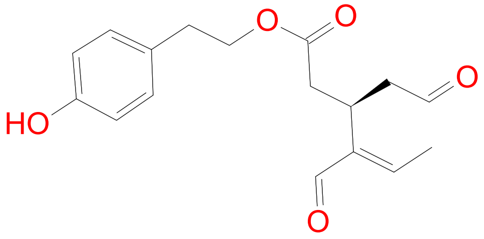

title P146_oleocanthal

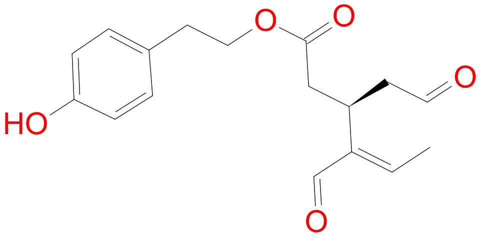

title P147_p-HPEA-EDA

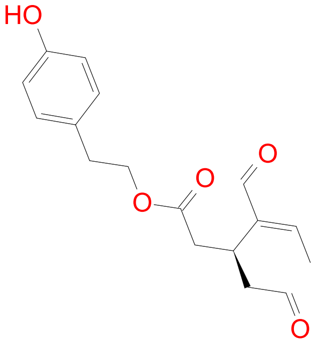

title P147_p-HPEA-EDA

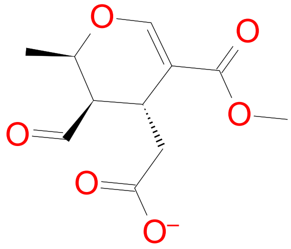

title P148_Elenolic acid

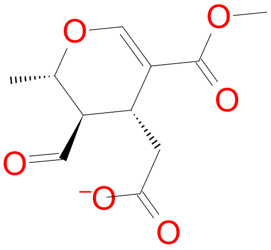

title P148_Elenolic acid

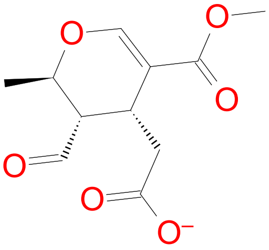

title P148_Elenolic acid

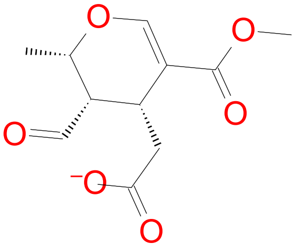

title P148_Elenolic acid

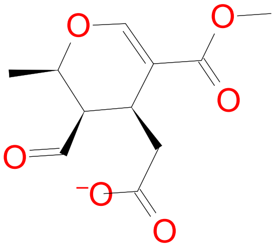

title P148_Elenolic acid

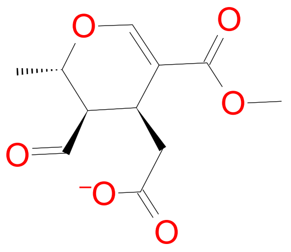

title P148_Elenolic acid

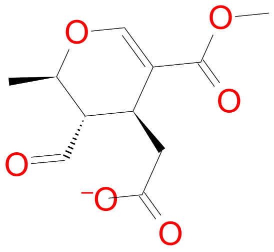

title P148_Elenolic acid

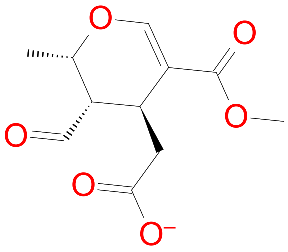

title P148_Elenolic acid

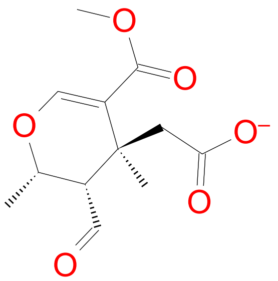

title P149_elenolic acid m

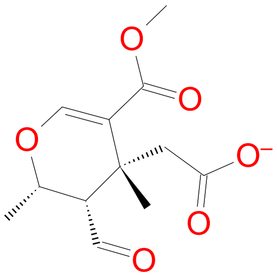

title P149_elenolic acid m

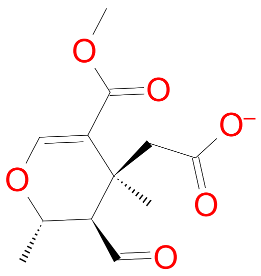

title P149_elenolic acid m

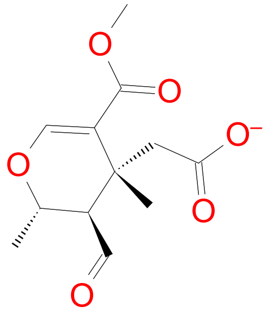

title P149_elenolic acid m

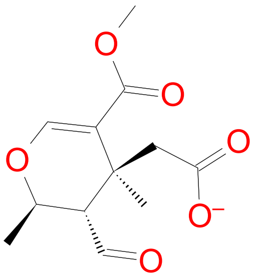

title P149_elenolic acid m

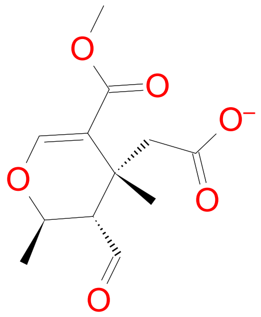

title P149_elenolic acid m

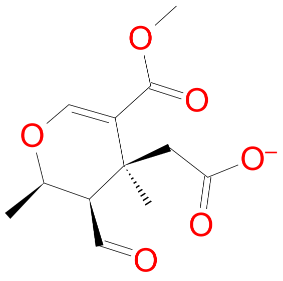

title P149_elenolic acid m

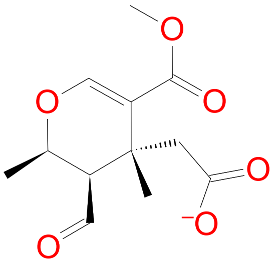

title P149_elenolic acid m

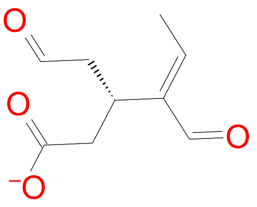

title P150_Dialdehydic ele

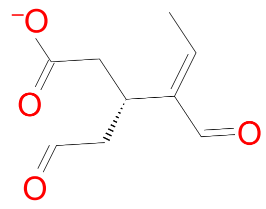

title P150_Dialdehydic ele

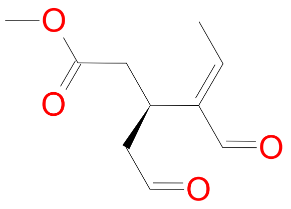

title P151_Dialdehydic ele

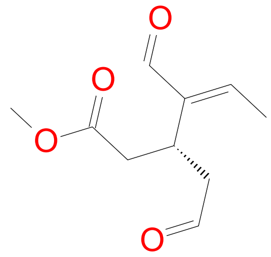

title P151_Dialdehydic ele

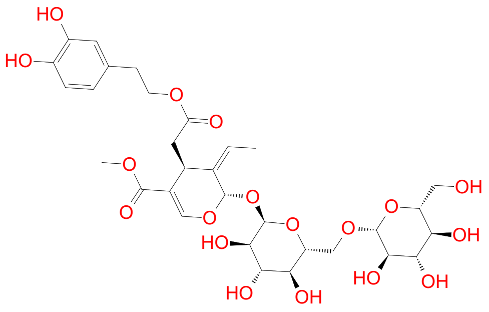

title P152_Oleuricine A.cc

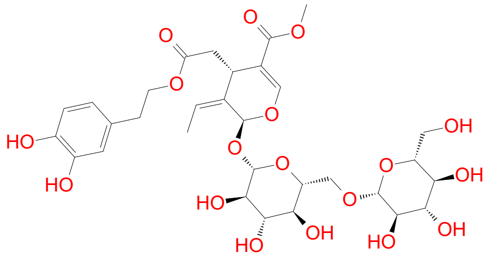

title P152_Oleuricine A.cc

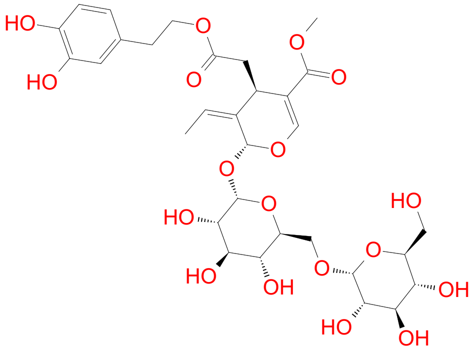

title P152_Oleuricine A.cc

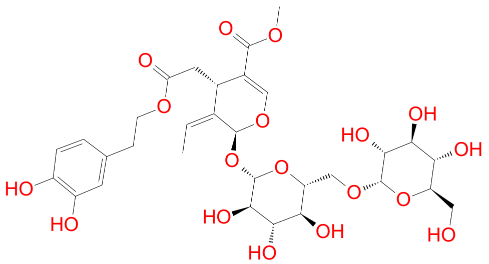

title P152_Oleuricine A.cc

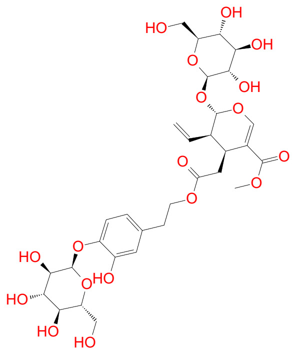

title P153_Oleuricine B.cc

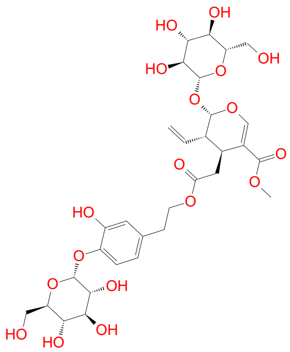

title P153_Oleuricine B.cc

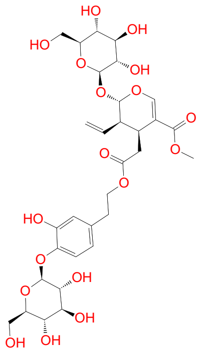

title P153_Oleuricine B.cc

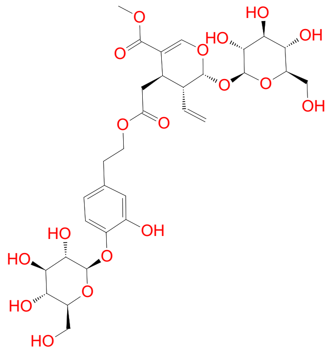

title P153_Oleuricine B.cc

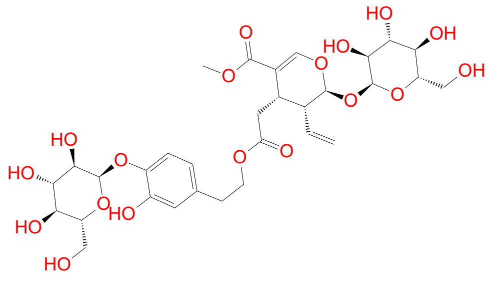

title P153_Oleuricine B.cc

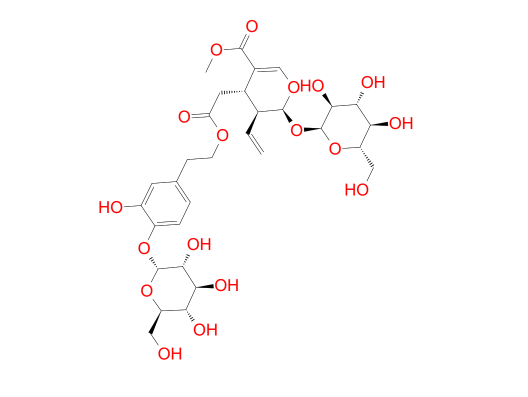

title P153_Oleuricine B.cc

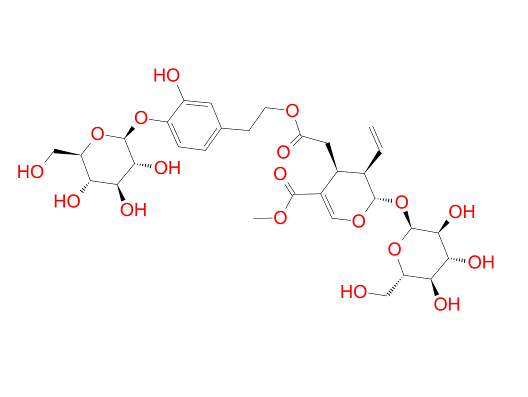

title P153_Oleuricine B.cc

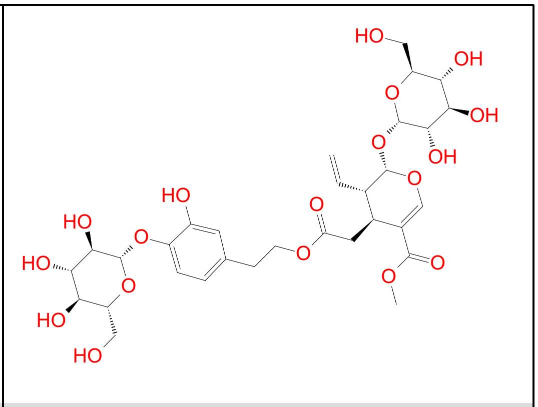

title P153_Oleuricine B.cc

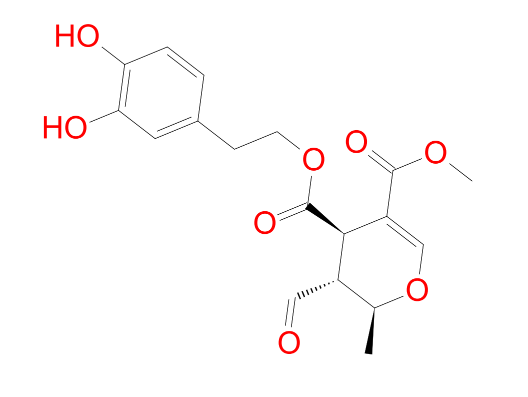

title P154_Hydroxytyrosil

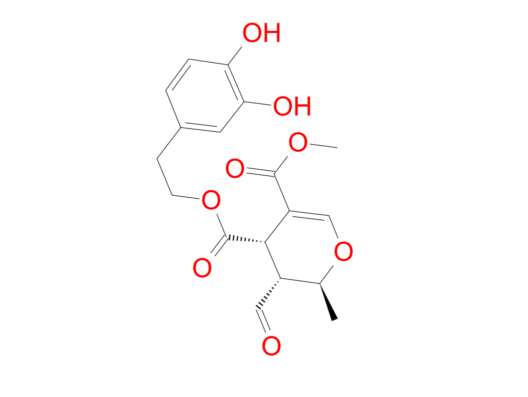

title P154_Hydroxytyrosil

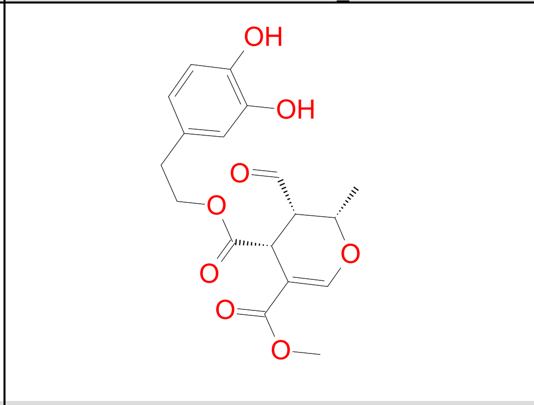

title P154_Hydroxytyrosil

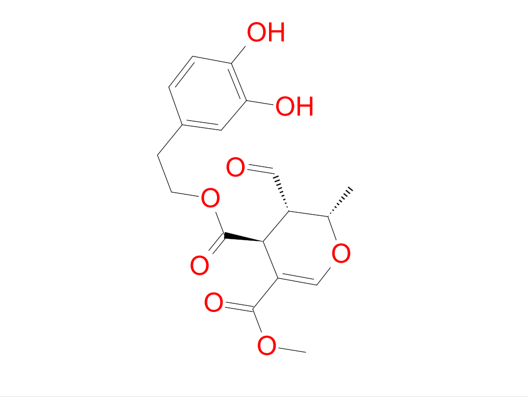

title P154_Hydroxytyrosil

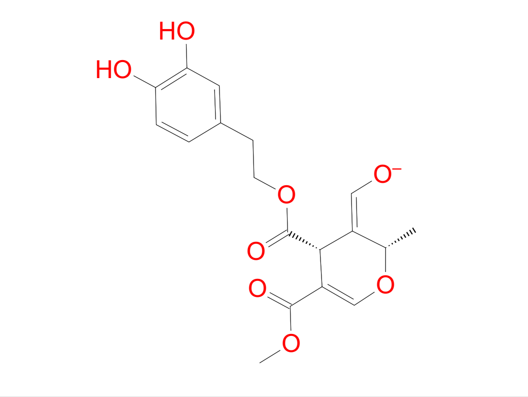

title P154_Hydroxytyrosil

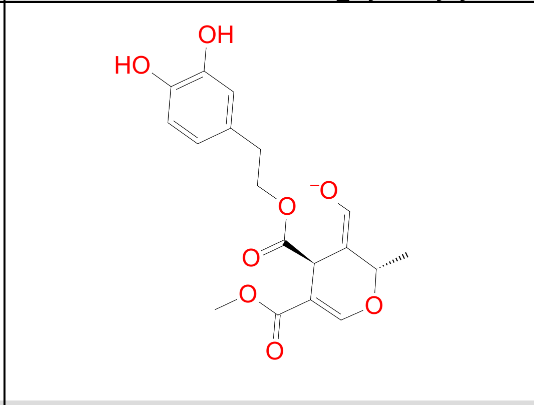

title P154_Hydroxytyrosil

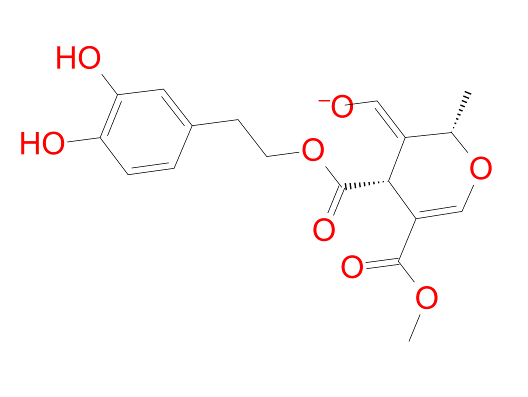

title P154_Hydroxytyrosil

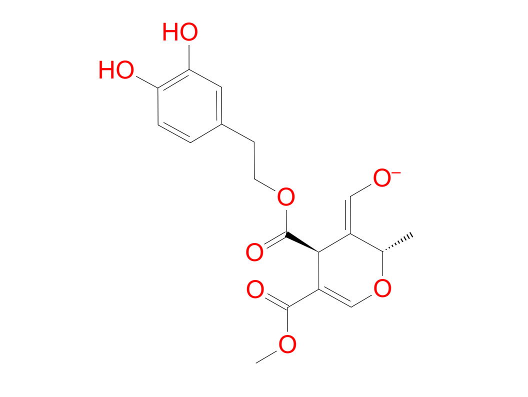

title P154_Hydroxytyrosil

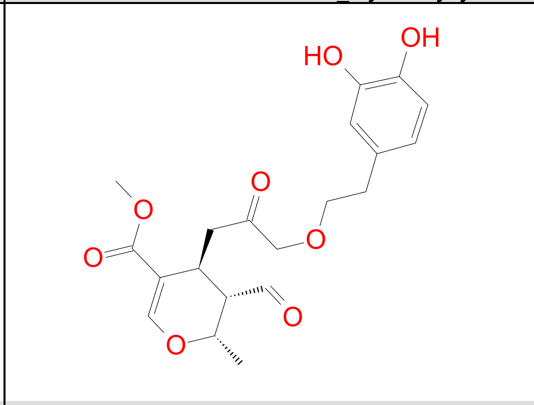

title P154_hydroxytyrosil

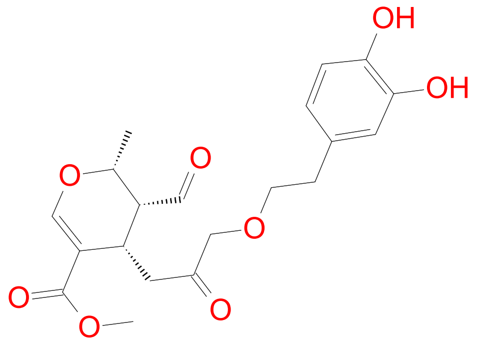

title P154_hydroxytyrosil-

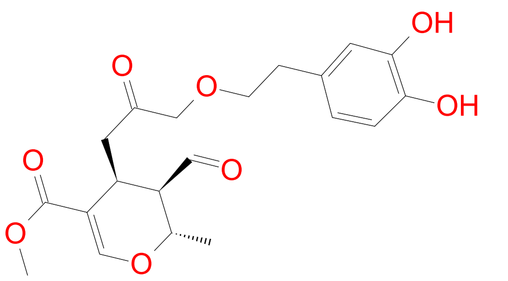

title P154_hydroxytyrosil-

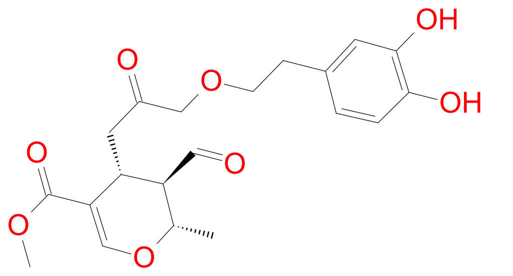

title P154_hydroxytyrosil-

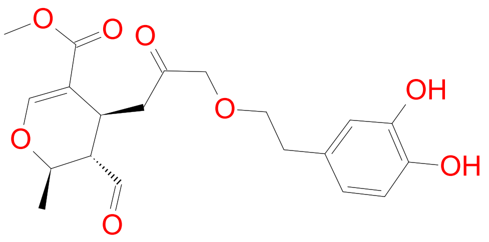

title P154_hydroxytyrosil-

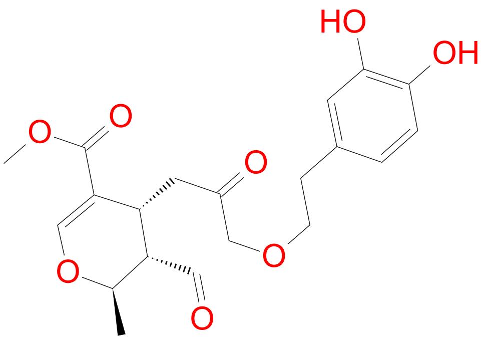

title P154_hydroxytyrosil-

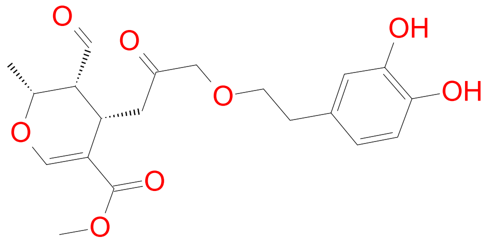

title P154_hydroxytyrosil-

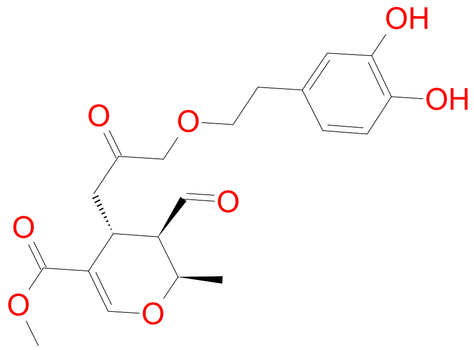

title P154_hydroxytyrosil-

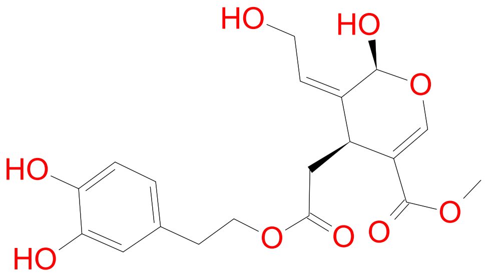

title P155_10-hydroxy ole

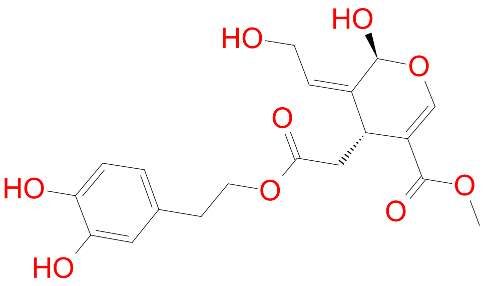

title P155_10-hydroxy ole

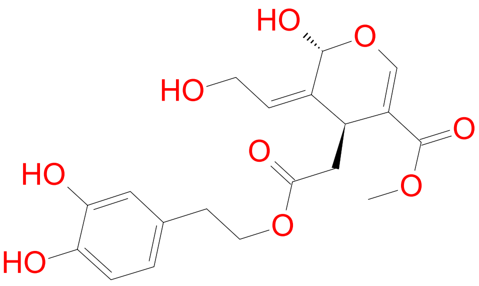

title P155_10-hydroxy ole

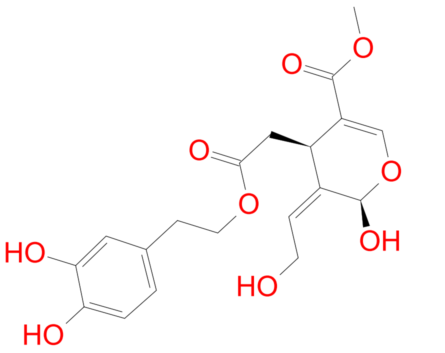

title P155_10-hydroxy ole

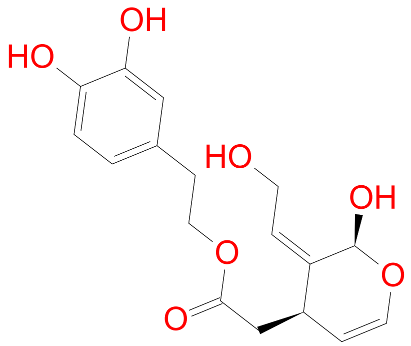

title P156_10-hydroxy ole

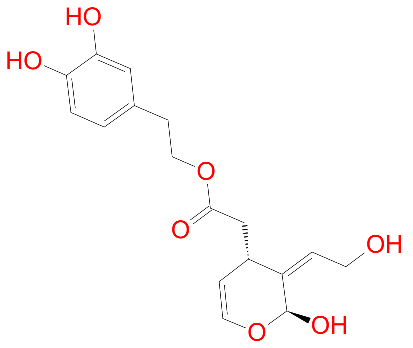

title P156_10-hydroxy ole

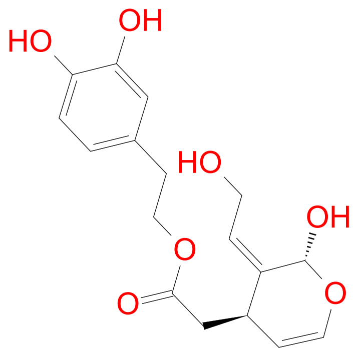

title P156_10-hydroxy ole

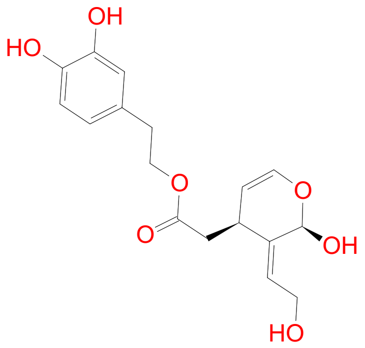

title P156_10-hydroxy ole

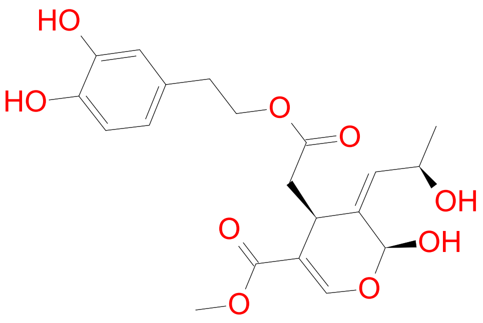

title P157_10-Hydroxy-10

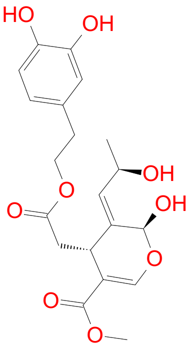

title P157_10-Hydroxy-10

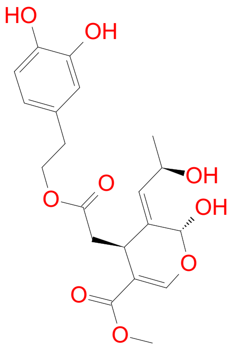

title P157_10-Hydroxy-10

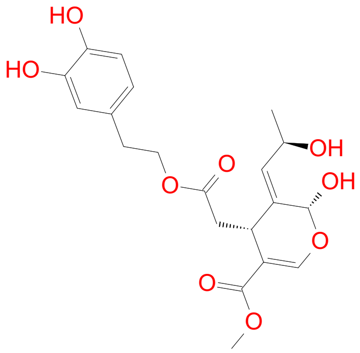

title P157_10-Hydroxy-10

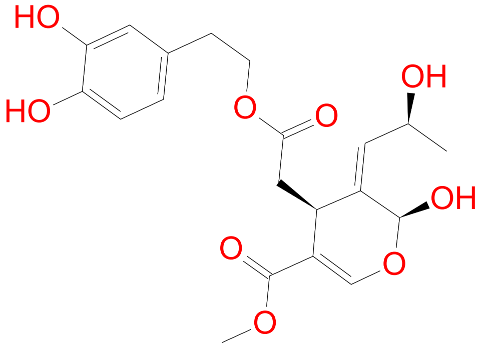

title P157_10-Hydroxy-10

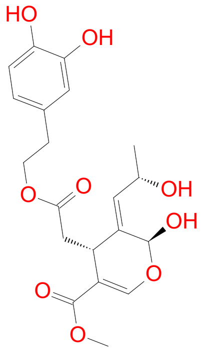

title P157_10-Hydroxy-10

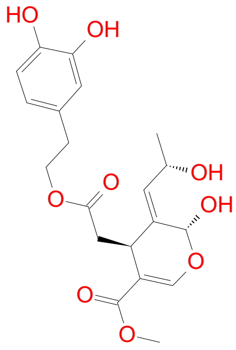

title P157_10-Hydroxy-10

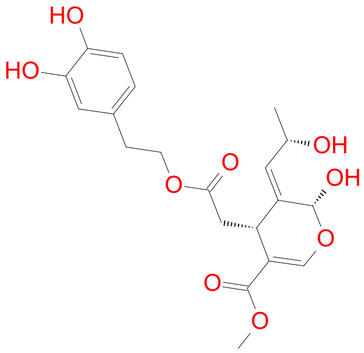

title P157_10-Hydroxy-10

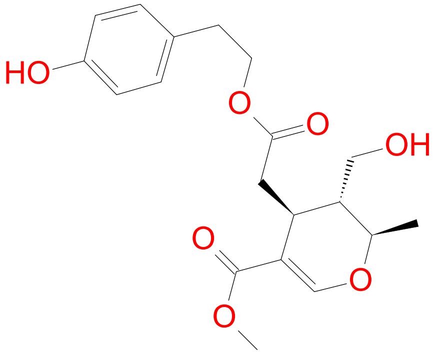

title P158_Monoaldehydic

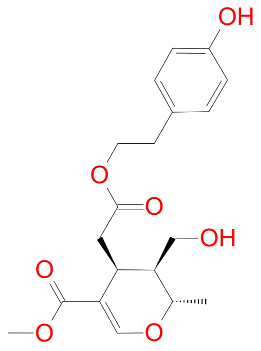

title P158_Monoaldehydic

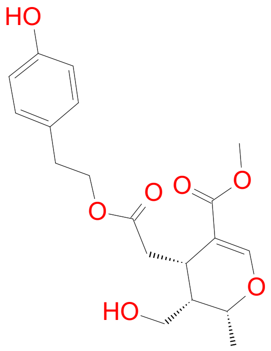

title P158_Monoaldehydic

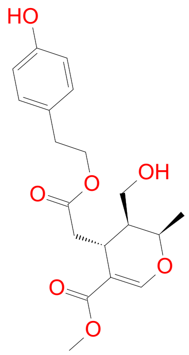

title P158_Monoaldehydic

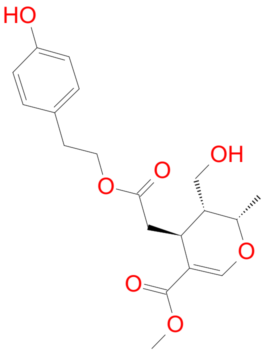

title P158_Monoaldehydic

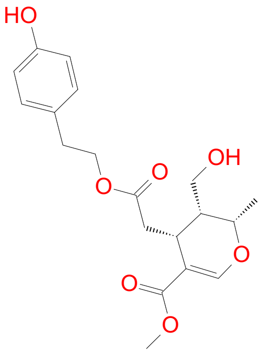

title P158_Monoaldehydic

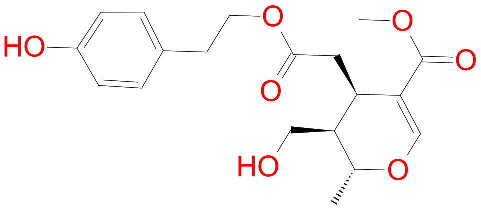

title P158_Monoaldehydic

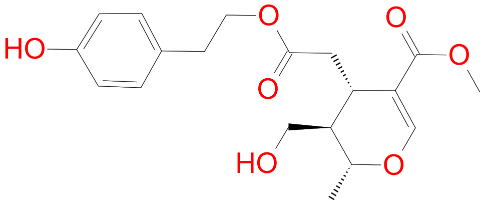

title P158_Monoaldehydic

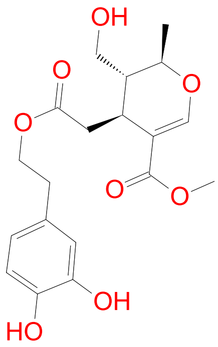

title P159_Monoaldehydic

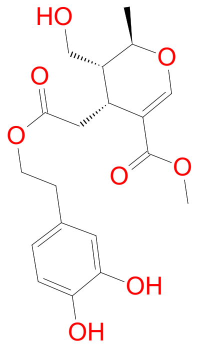

title P159_Monoaldehydic

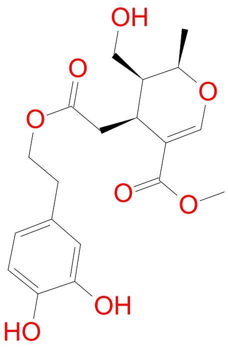

title P159_Monoaldehydic

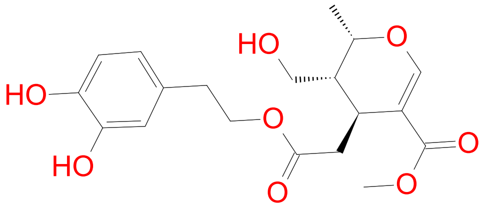

title P159_Monoaldehydic

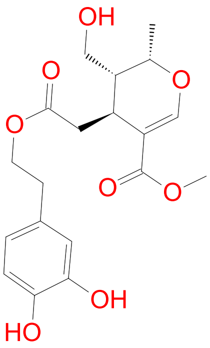

title P159_Monoaldehydic

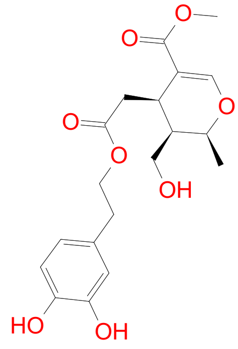

title P159_Monoaldehydic

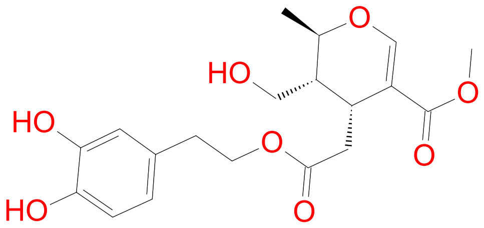

title P159_Monoaldehydic

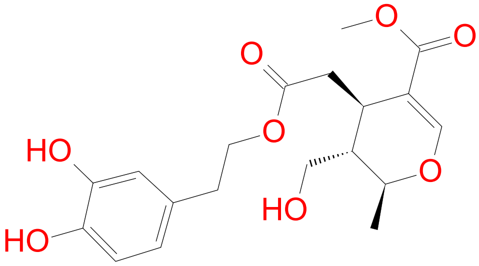

title P159_Monoaldehydic

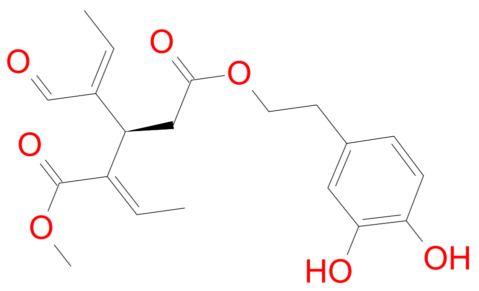

title P160_Oleuropeindial

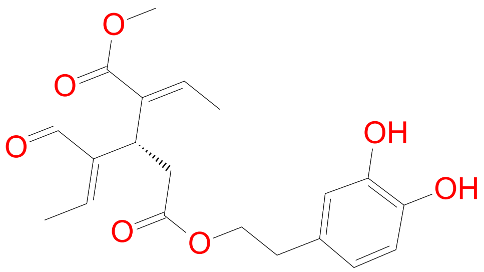

title P160_Oleuropeindial

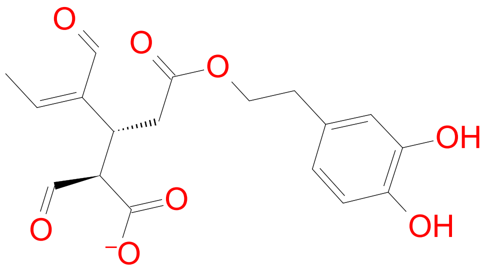

title P161_demethyloleur

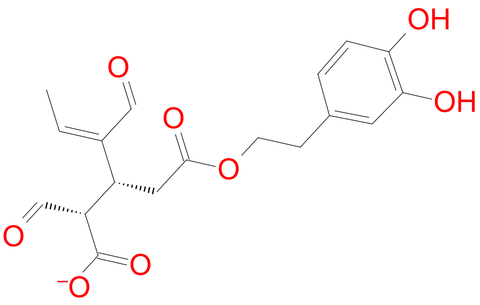

title P161_demethyloleur

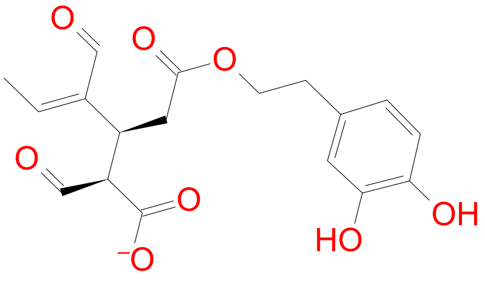

title P161_demethyloleur

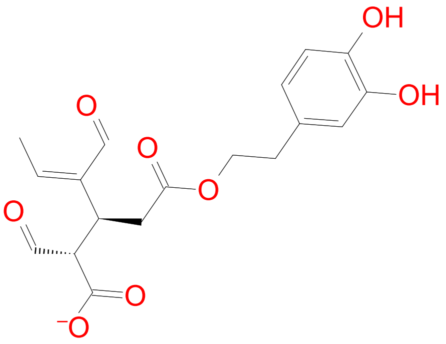

title P161_demethyloleur

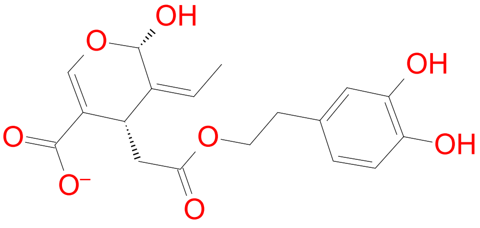

title P162_Demethyloleur

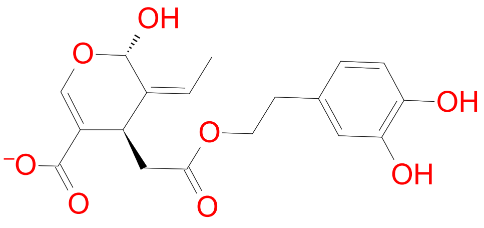

title P162_Demethyloleur

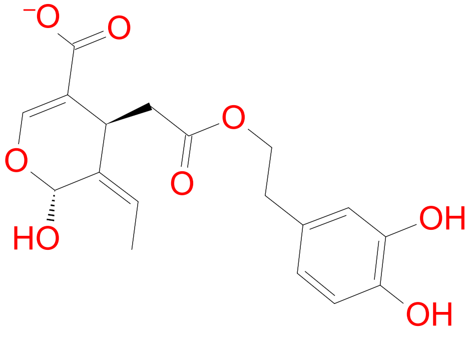

title P162_Demethyloleur

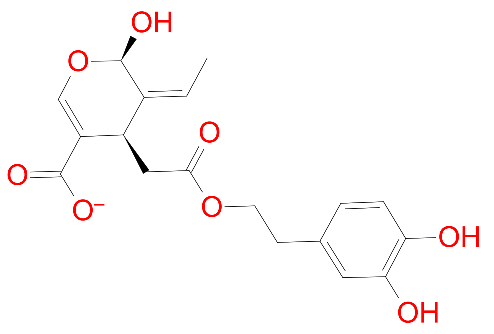

title P162_Demethyloleur

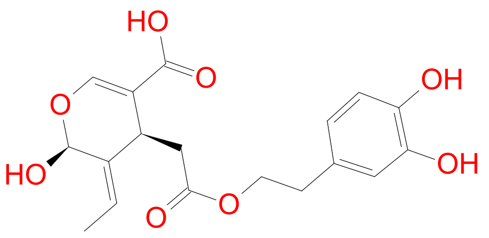

title P162_Demethyloleur

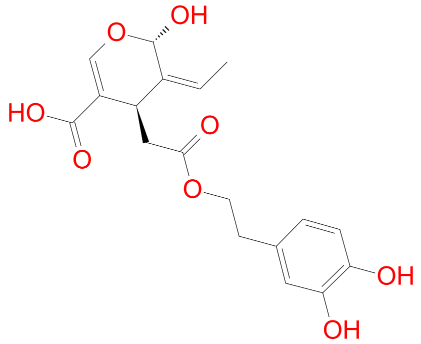

title P162_Demethyloleur

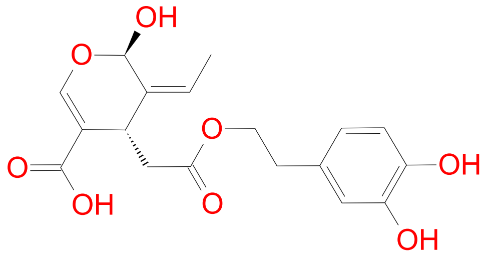

title P162_Demethyloleur

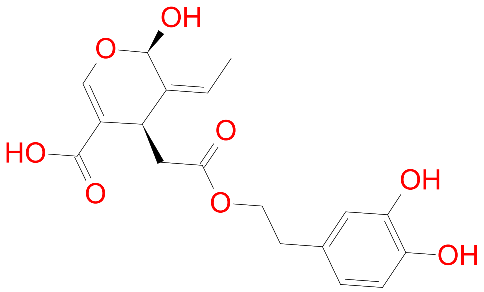

title P162_Demethyloleur

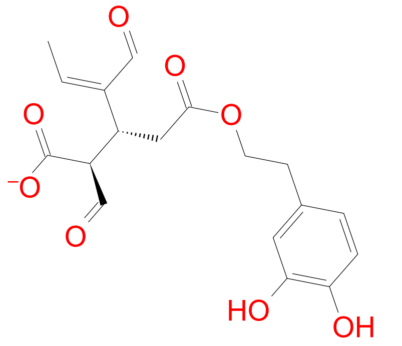

title P164_Demethyloleur

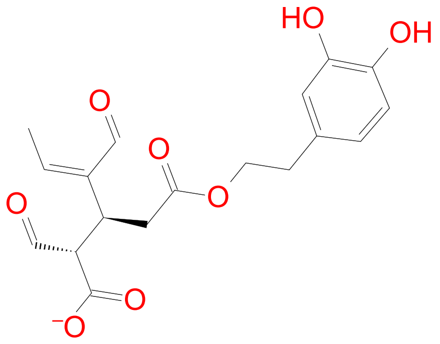

title P164_Demethyloleur

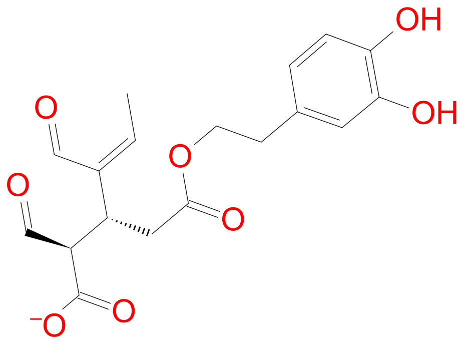

title P164_Demethyloleur

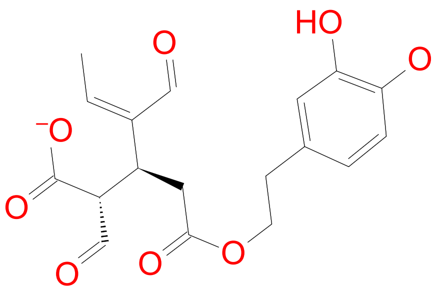

title P164_Demethyloleur

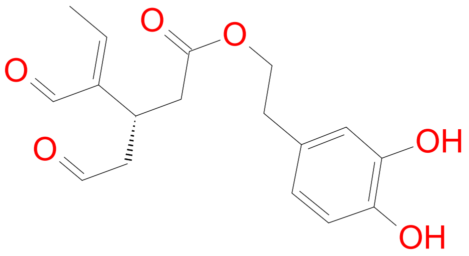

title P165_DHPEA-DEDA

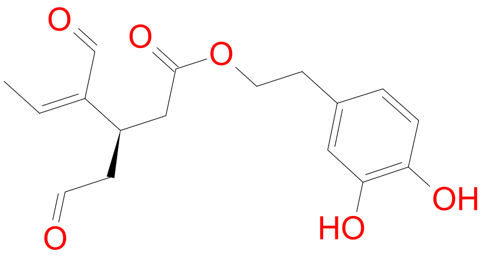

title P165_DHPEA-DEDA

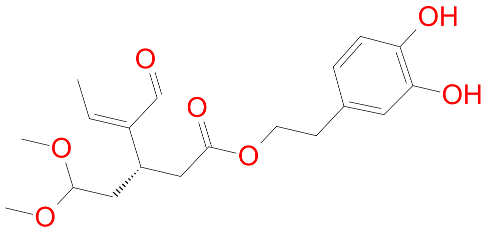

title P166_DHPEA-DEDA ε

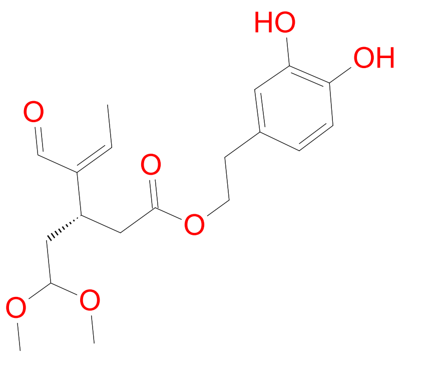

title P166_DHPEA-DEDA a

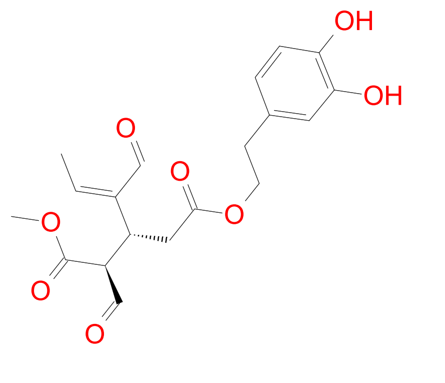

title P167_Oleuropeindial

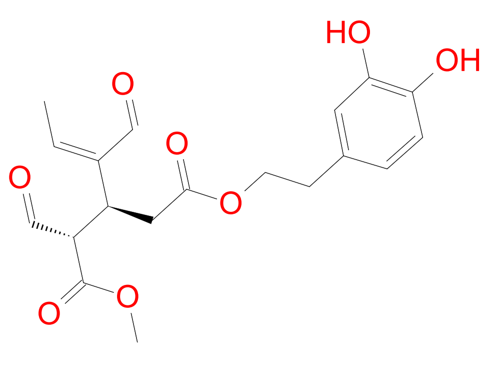

title P167_Oleuropeindial

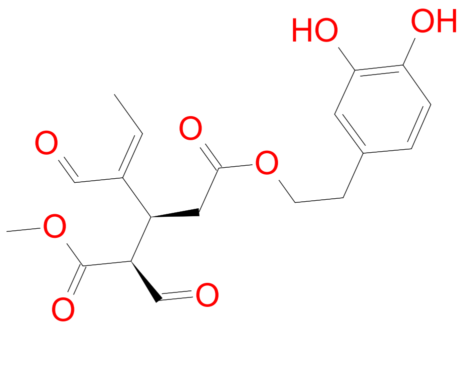

title P167_Oleuropeindial

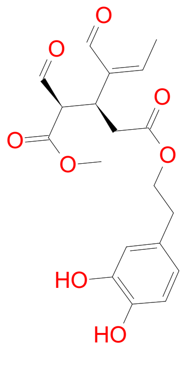

title P167_Oleuropeindial

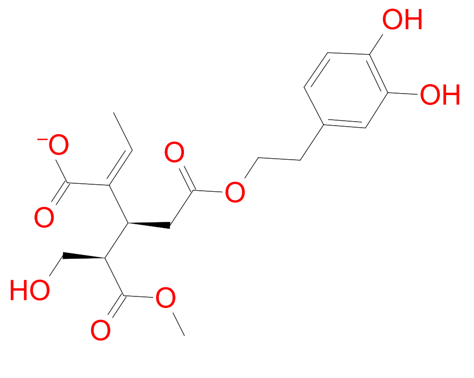

title P168_Oleuropeindial

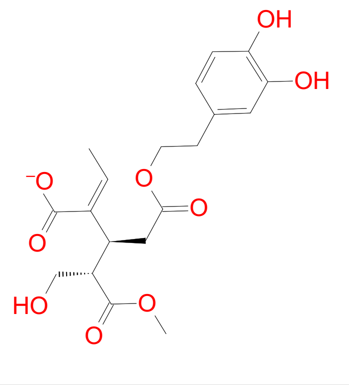

title P168_Oleuropeindial

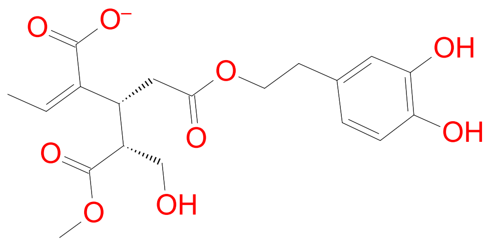

title P168_Oleuropeindial

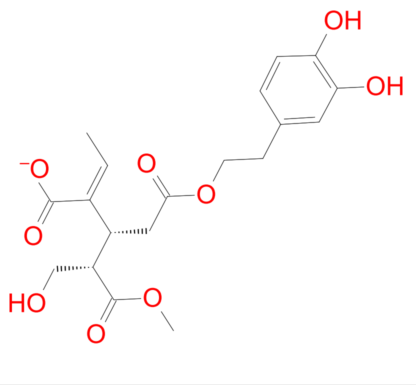

title P168_Oleuropeindial

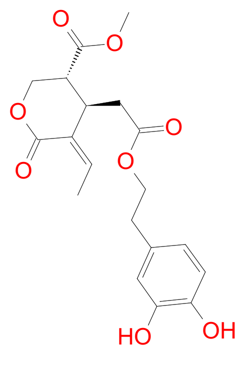

title P169_Oleuropeindial

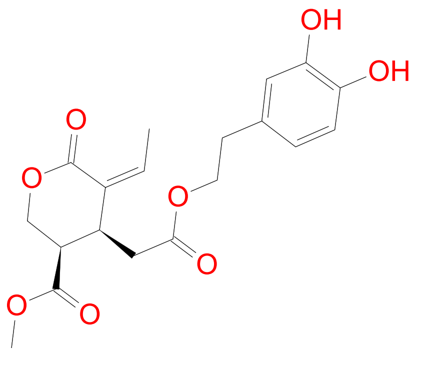

title P169_Oleuropeindial

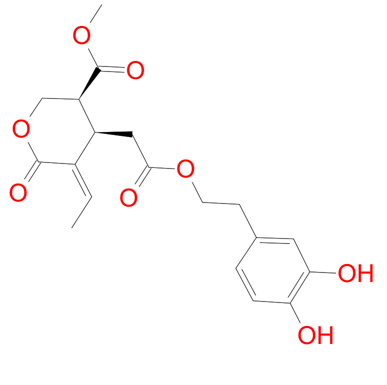

title P169_Oleuropeindial

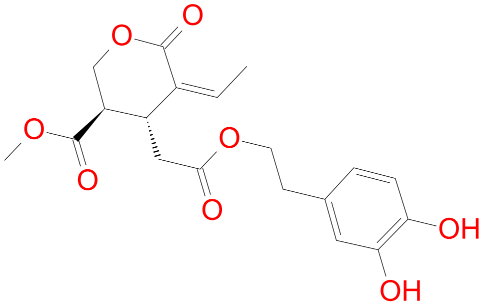

title P169_Oleuropeindial

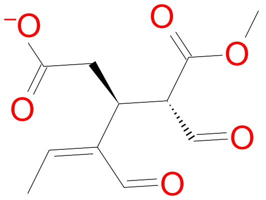

title P170_Elenolic acid di

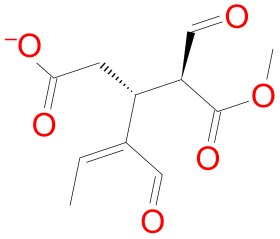

title P170_Elenolic acid di

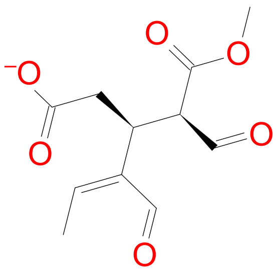

title P170_Elenolic acid di

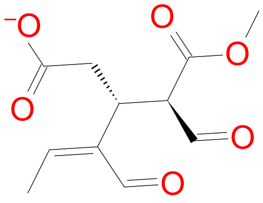

title P170_Elenolic acid di

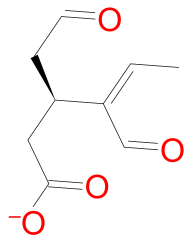

title P171_DEDA

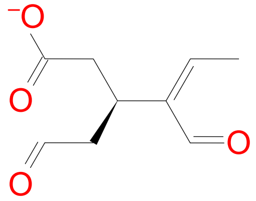

title P171_DEDA

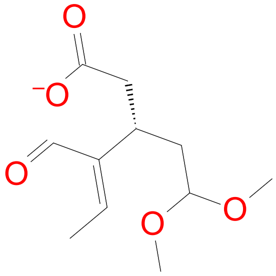

title P172_DEDA acetal

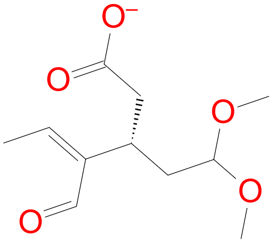

title P172_DEDA acetal

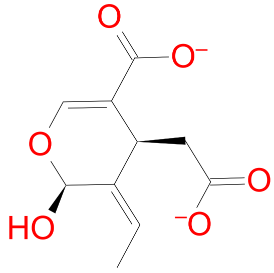

title P173_Demethyl elen

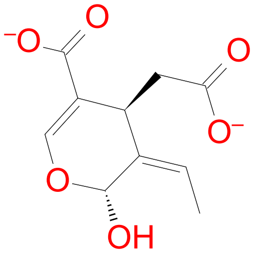

title P173_Demethyl elen

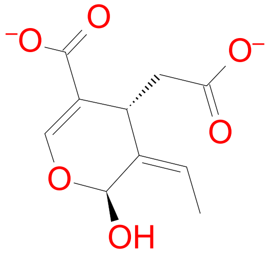

title P173_Demethyl elen

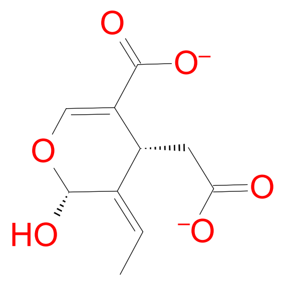

title P173_Demethyl elen

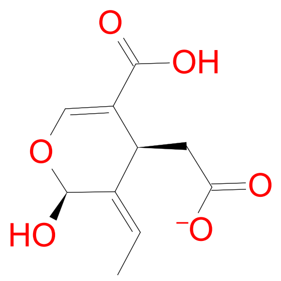

title P173_Demethyl elen

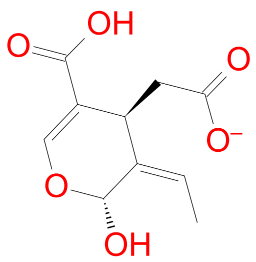

title P173_Demethyl elen

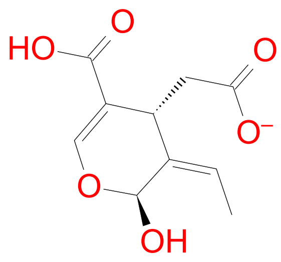

title P173_Demethyl elen

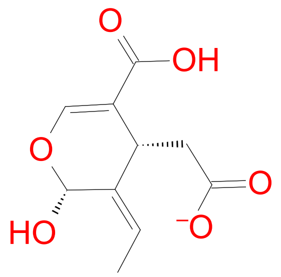

title P173_Demethyl elen

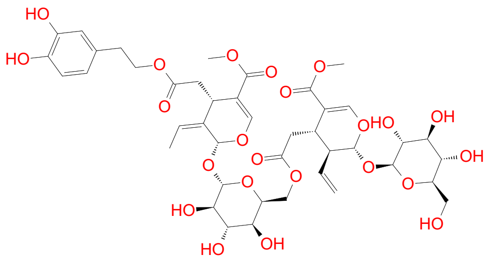

title P174_Jaspolyoside.cc

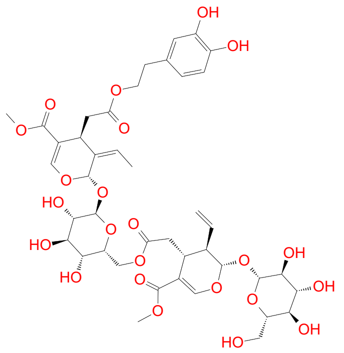

title P174_Jaspolyoside.cc

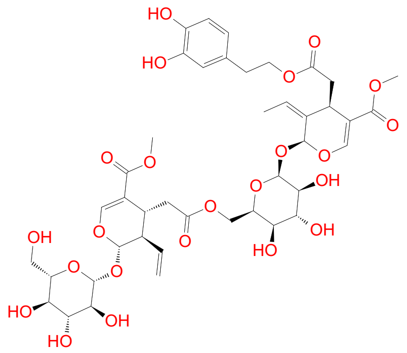

title P174_Jaspolyoside.cc

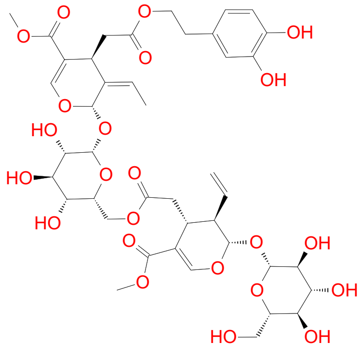

title P174_Jaspolyoside.cc

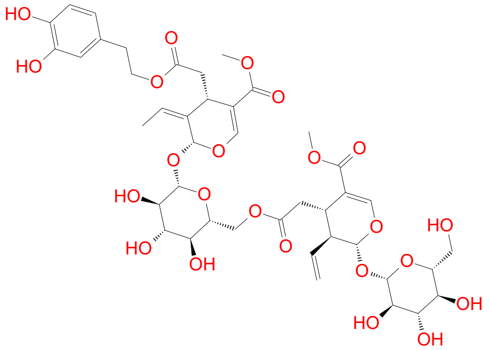

title P174_Jaspolyoside.cc

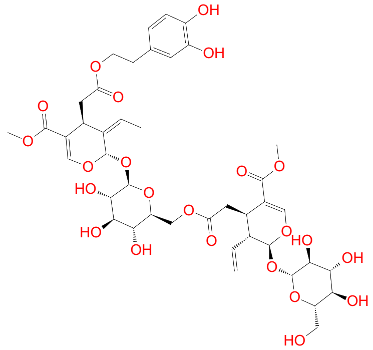

title P174_Jaspolyoside.cc

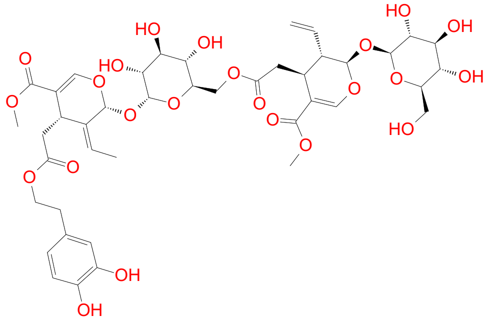

title P174_Jaspolyoside.cc

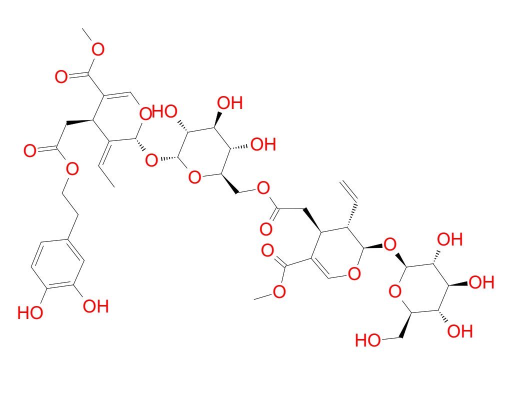

title P174_Jaspolyoside.cc

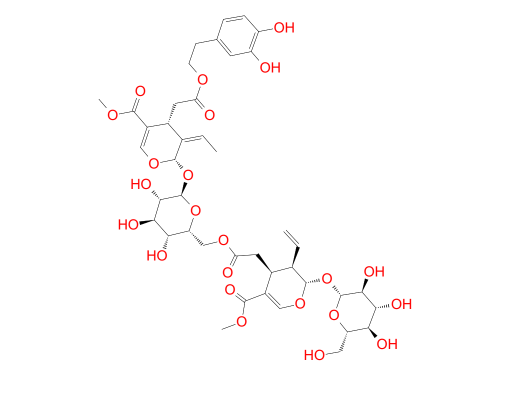

title P174_Jaspolyoside.cc

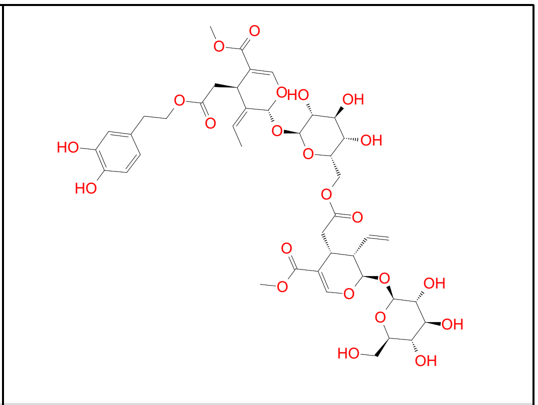

title P174_Jaspolyoside.cc

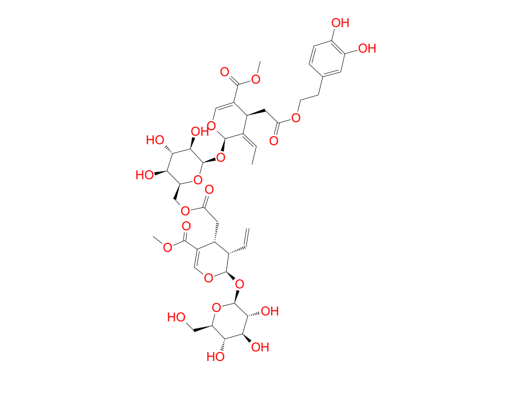

title P174_Jaspolyoside.cc

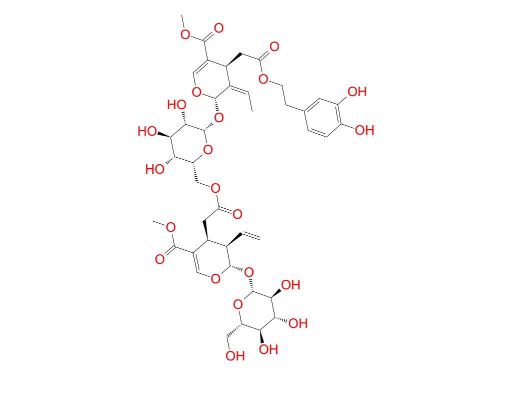

title P174_Jaspolyoside.cc

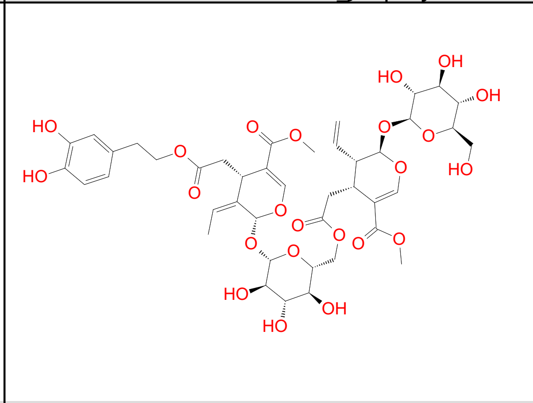

title P174_Jaspolyoside.cc

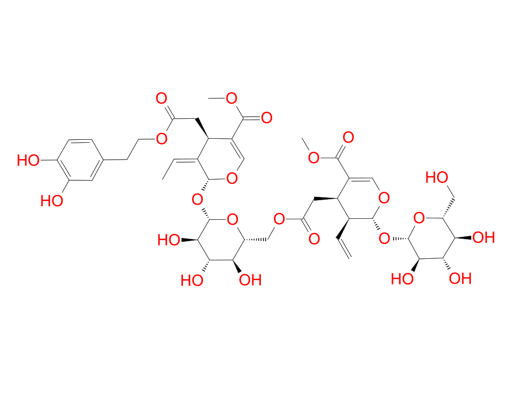

title P174_Jaspolyoside.cc

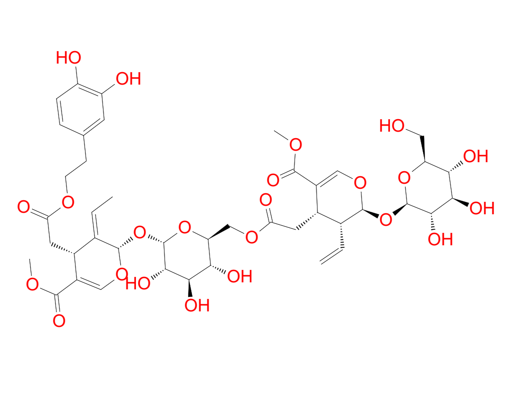

title P174_Jaspolyoside.cc

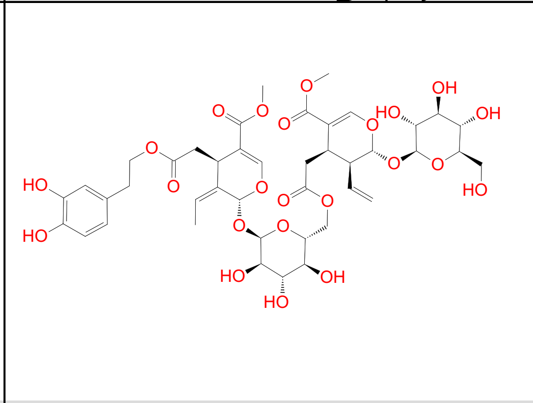

title P174_Jaspolyoside.cc

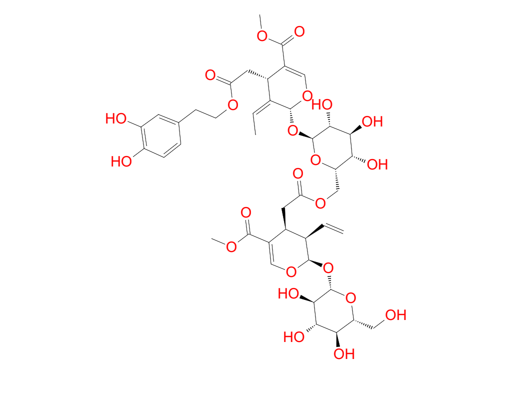

title P174_Jaspolyoside.cc

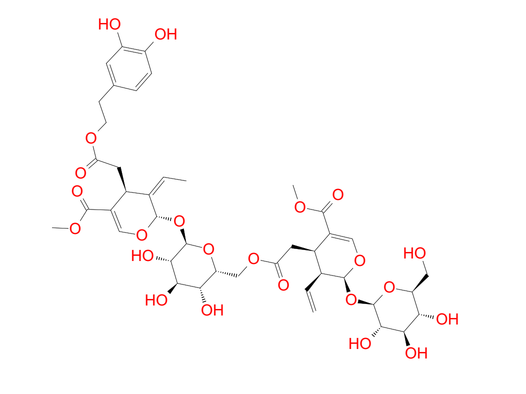

title P174_Jaspolyoside.cc

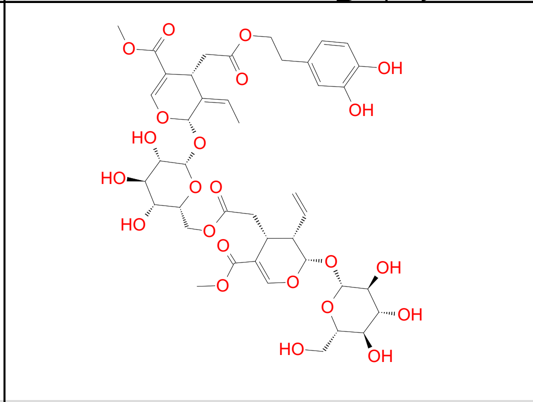

title P174_Jaspolyoside.cc

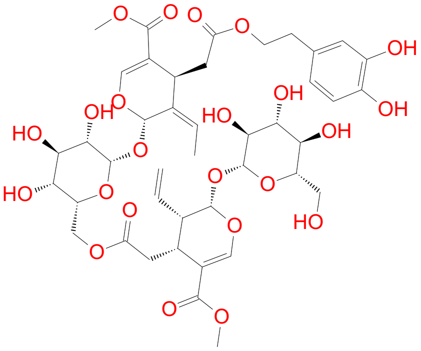

title P174_Jaspolyoside.cc

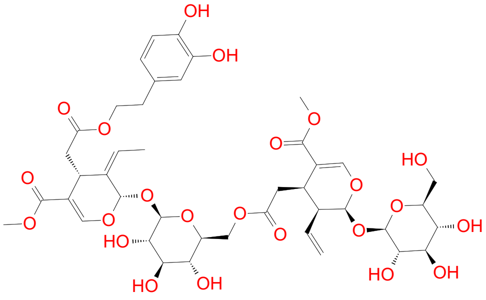

title P174_Jaspolyoside.cc

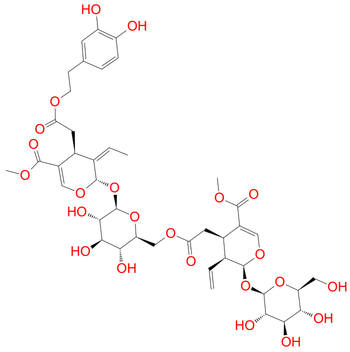

title P174_Jaspolyoside.cc

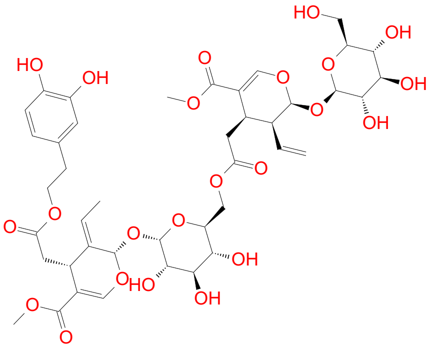

title P174_Jaspolyoside.cc

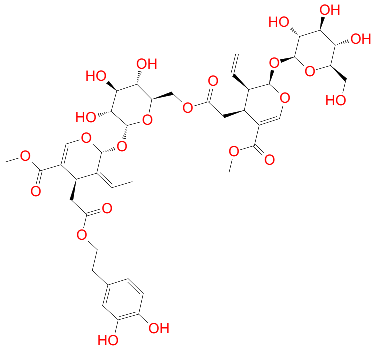

title P174_Jaspolyoside.cc

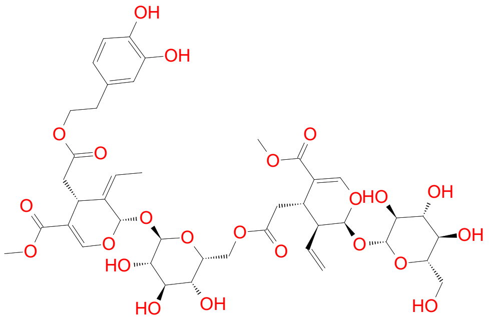

title P174_Jaspolyoside.cc

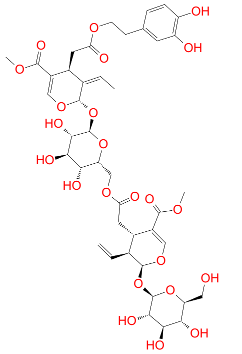

title P174_Jaspolyoside.cc

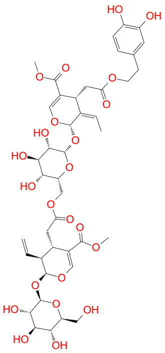

title P174_Jaspolyoside.cc

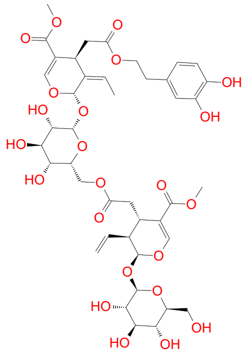

title P174_Jaspolyoside.cc

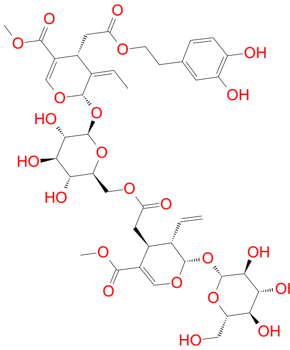

title P174_Jaspolyoside.cc

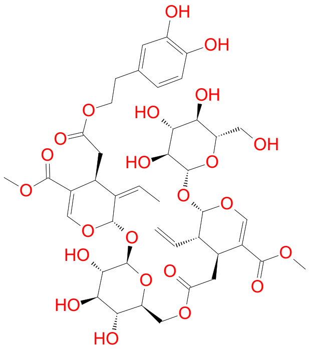

title P174_Jaspolyoside.cc

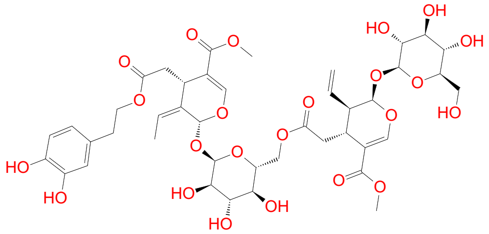

title P174_Jaspolyoside.cc

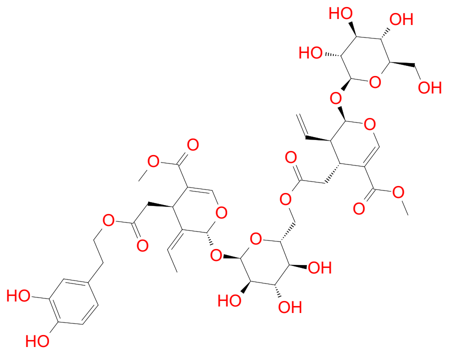

title P174_Jaspolyoside.c

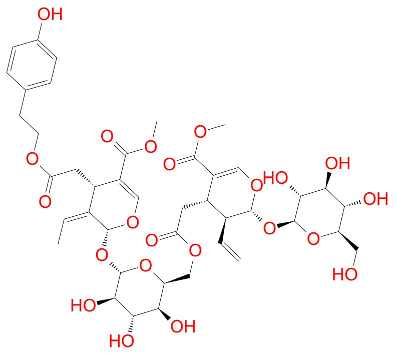

title P175_Jaspolyanoside

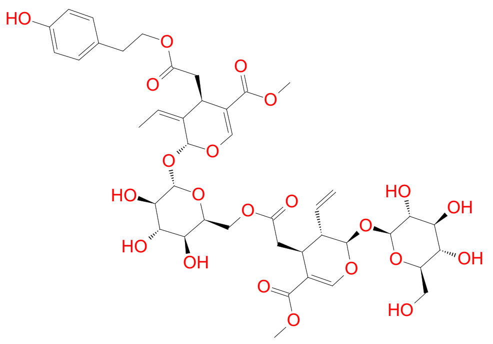

title P175_Jaspolyanoside

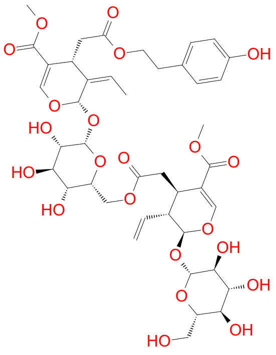

title P175_Jaspolyanoside

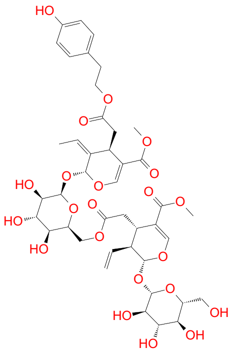

title P175_Jaspolyanoside

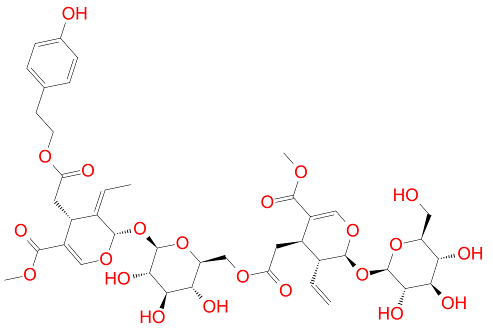

title P175_Jaspolyanoside

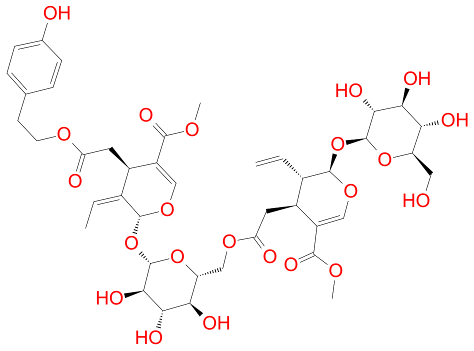

title P175_Jaspolyanoside

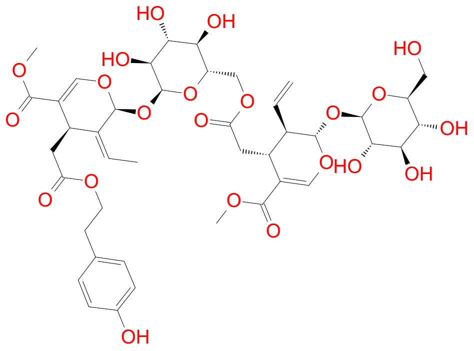

title P175_Jaspolyanoside

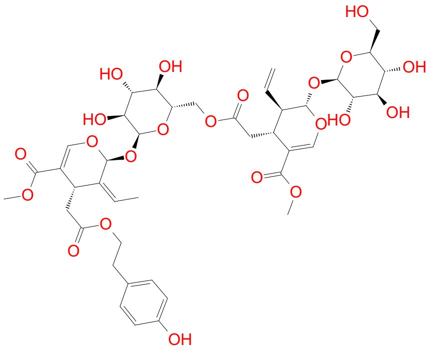

title P175_Jaspolyanoside

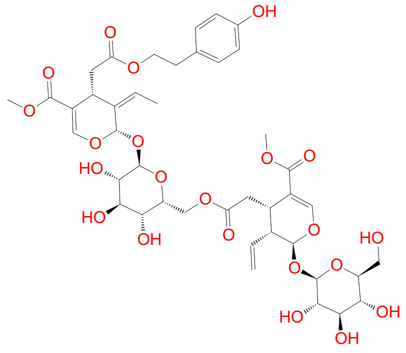

title P175_Jaspolyanoside

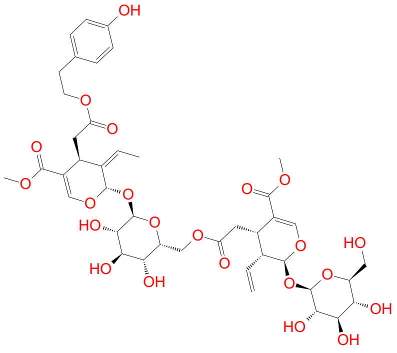

title P175_Jaspolyanoside

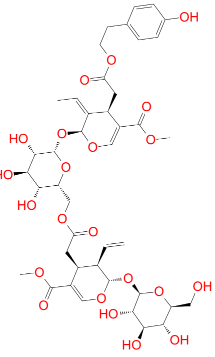

title P175_Jaspolyanoside

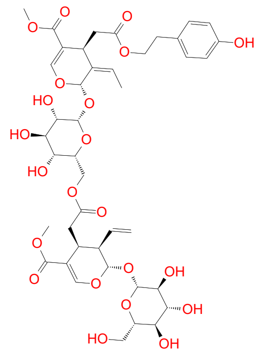

title P175_Jaspolyanoside

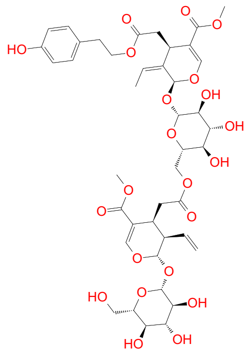

title P175_Jaspolyanoside

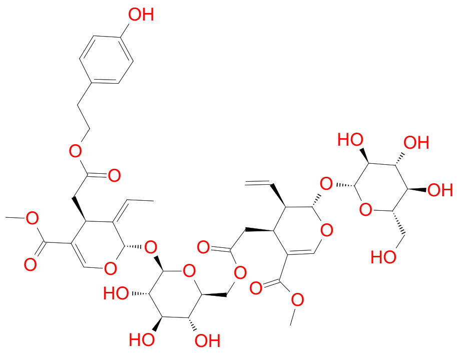

title P175_Jaspolyanoside

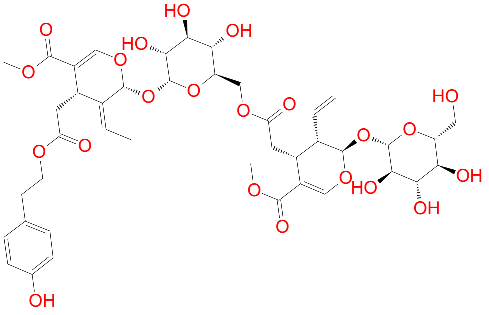

title P175_Jaspolyanoside

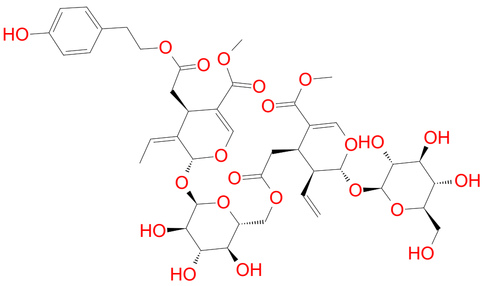

title P175_Jaspolyanoside

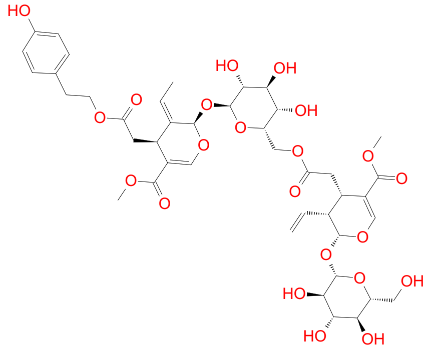

title P175_Jaspolyanoside

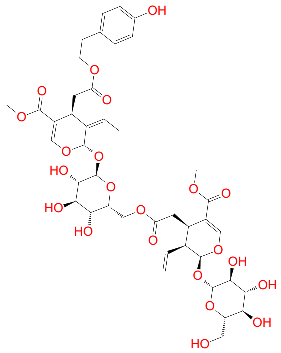

title P175_Jaspolyanoside

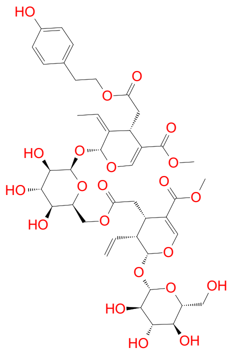

title P175_Jaspolyanoside

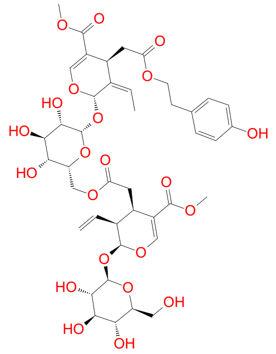

title P175_Jaspolyanoside

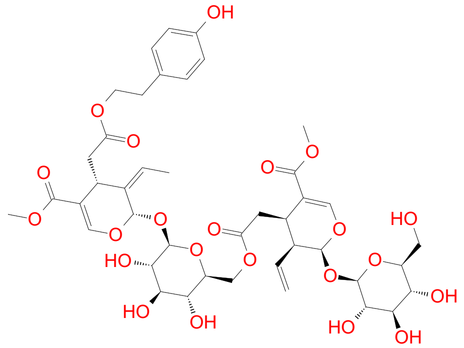

title P175_Jaspolyanoside

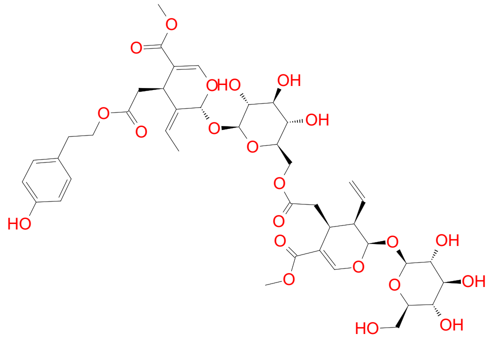

title P175_Jaspolyanoside

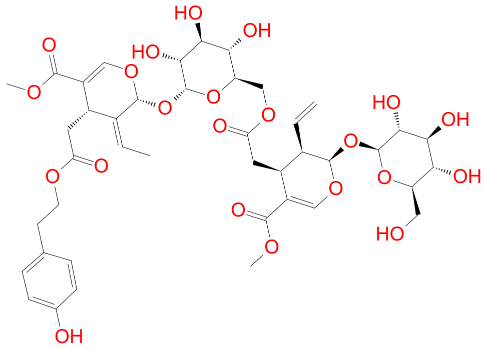

title P175_Jaspolyanoside

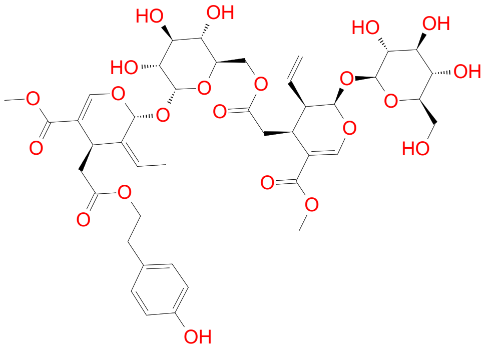

title P175_Jaspolyanoside

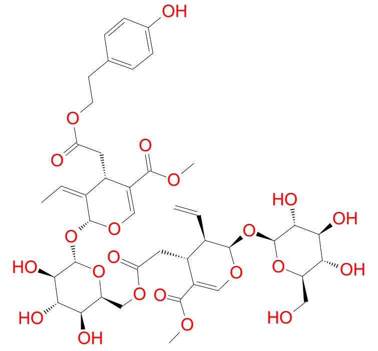

title P175_Jaspolyanoside

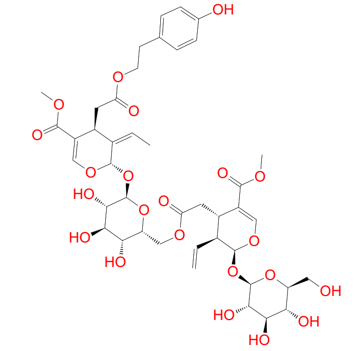

title P175_Jaspolyanoside

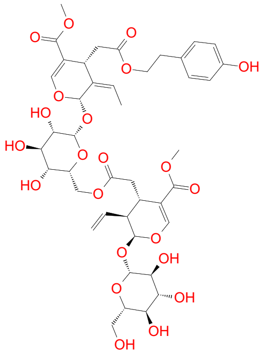

title P175_Jaspolyanoside

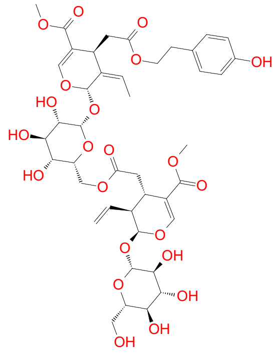

title P175_Jaspolyanoside

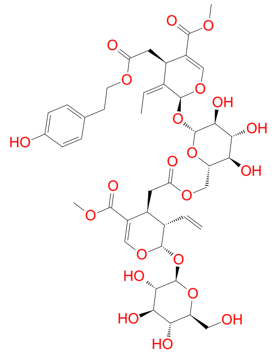

title P175_Jaspolyanoside

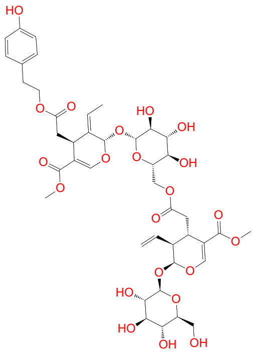

title P175_Jaspolyanoside

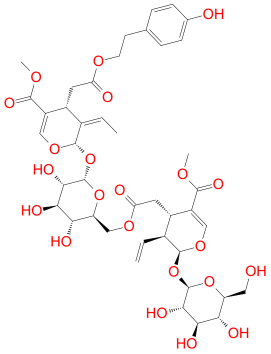

title P175_Jaspolyanoside

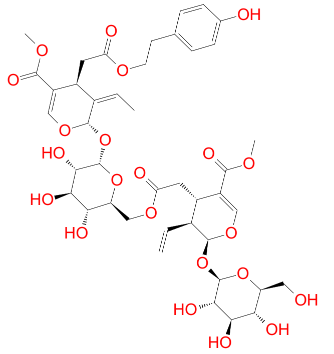

title P175_Jaspolyanoside

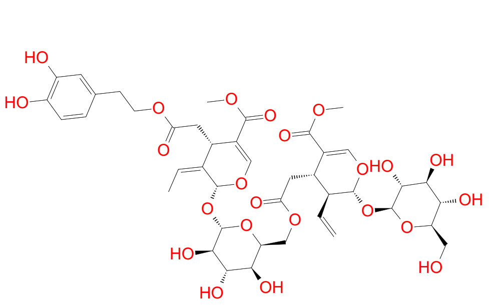

title P176_Isojaspolyoside

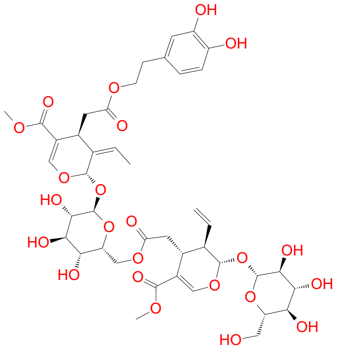

title P176_Isojaspolyoside

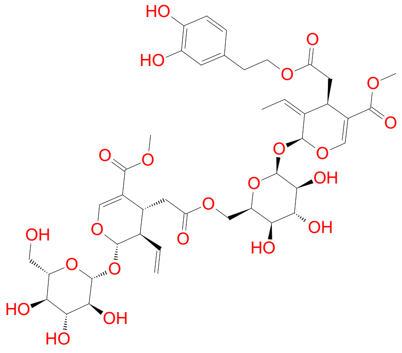

title P176_Isojaspolyoside

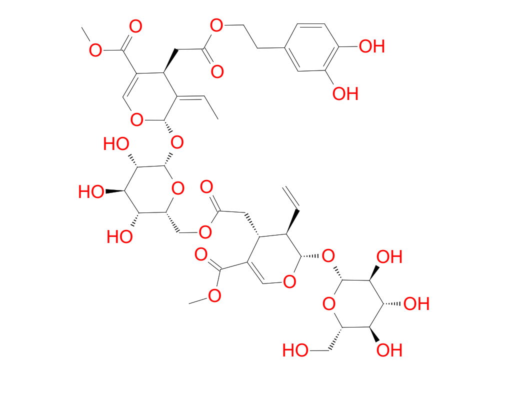

title P176_Isojaspolyoside

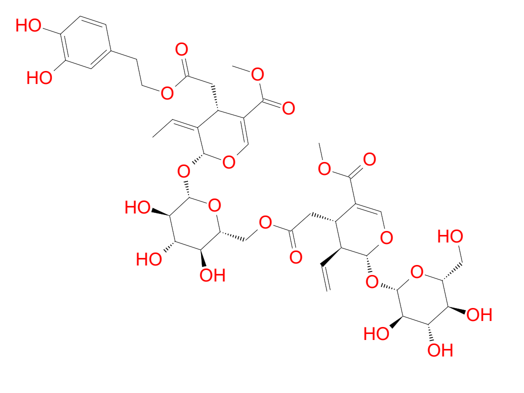

title P176_Isojaspolyoside

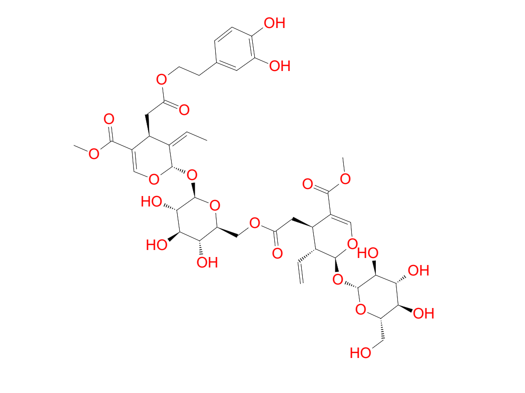

title P176_Isojaspolyoside

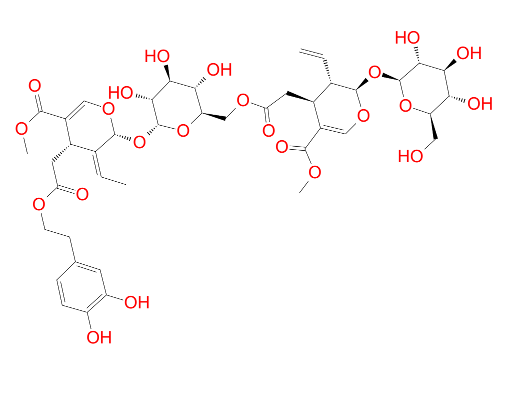

title P176_Isojaspolyoside

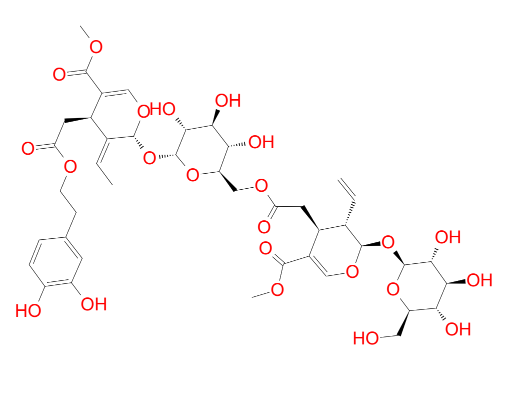

title P176_Isojaspolyoside

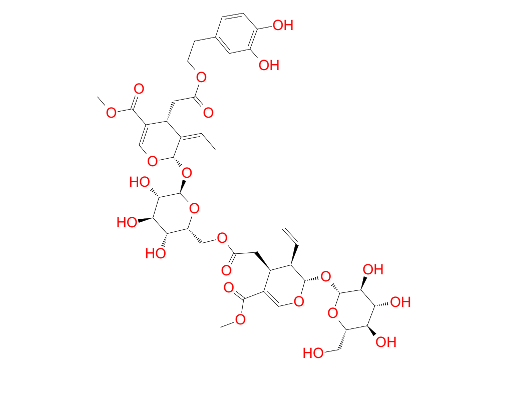

title P176_Isojaspolyoside

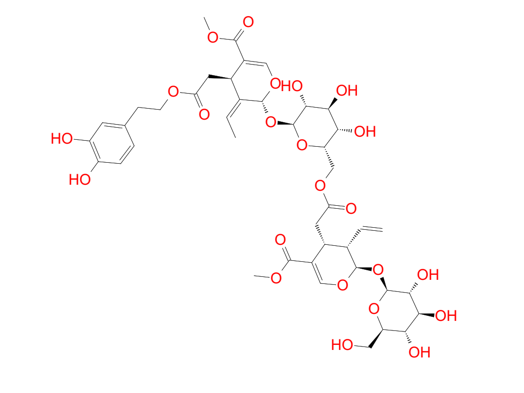

title P176_Isojaspolyoside

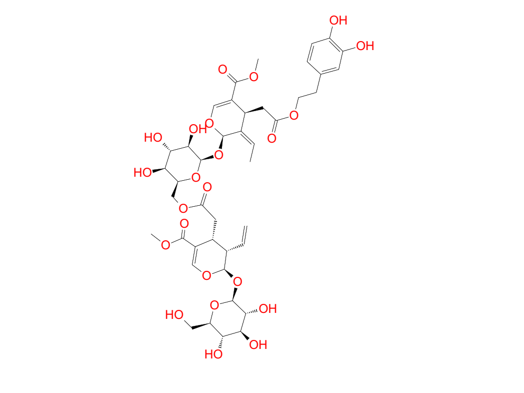

title P176_Isojaspolyoside

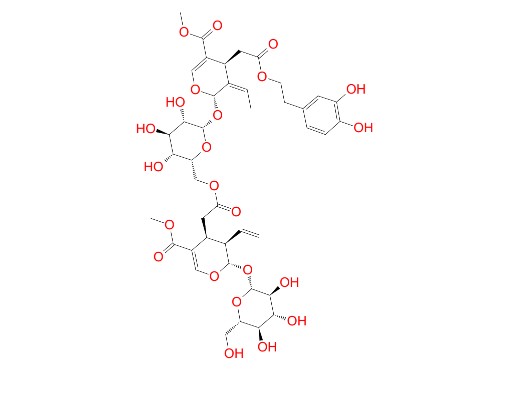

title P176_Isojaspolyoside

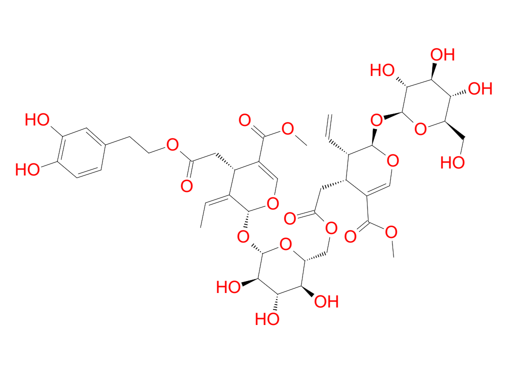

title P176_Isojaspolyoside

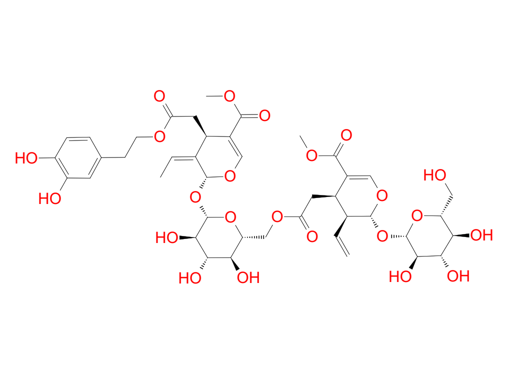

title P176_Isojaspolyoside

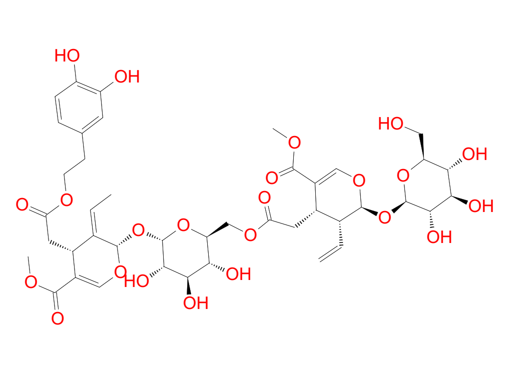

title P176_Isojaspolyoside

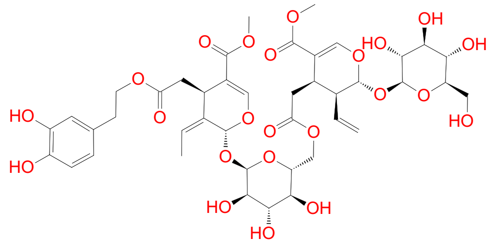<br>title P176_Isojaspolyoside	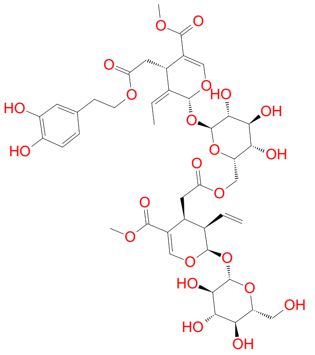<br>title P176_Isojaspolyoside	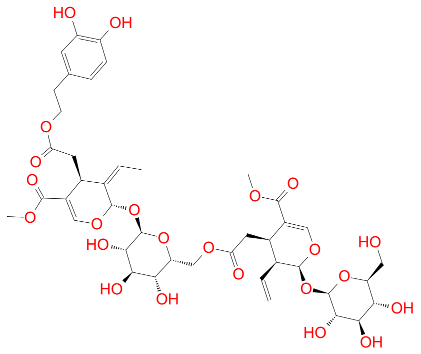<br>title P176_Isojaspolyoside
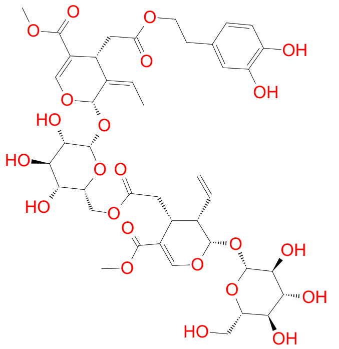<br>title P176_Isojaspolyoside	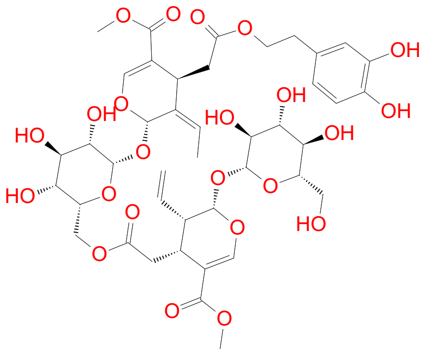<br>title P176_Isojaspolyoside	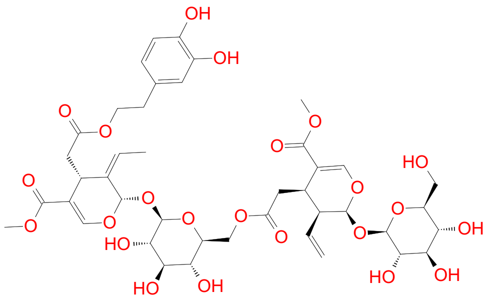<br>title P176_Isojaspolyoside
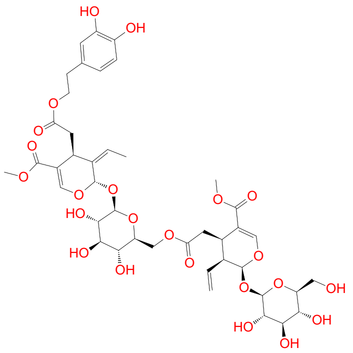<br>title P176_Isojaspolyoside	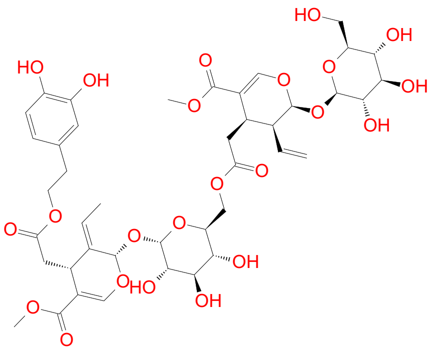<br>title P176_Isojaspolyoside	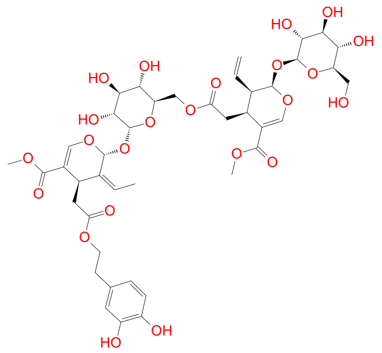<br>title P176_Isojaspolyoside
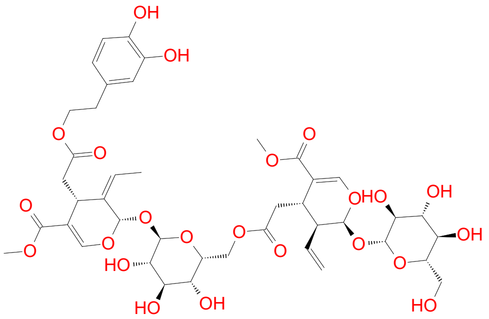<br>title P176_Isojaspolyoside	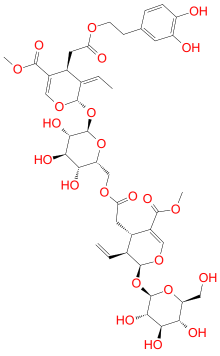<br>title P176_Isojaspolyoside	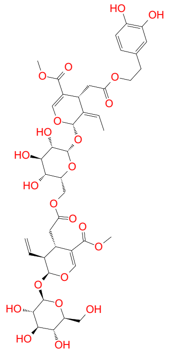<br>title P176_Isojaspolyoside

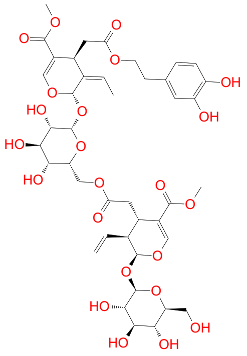

title P176_Isojaspolyoside

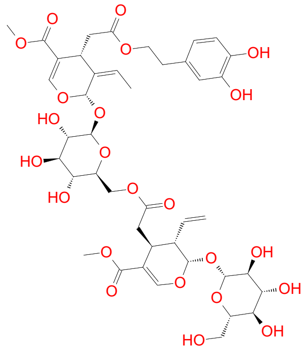

title P176_Isojaspolyoside

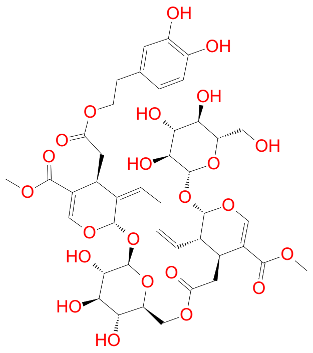

title P176_Isojaspolyoside

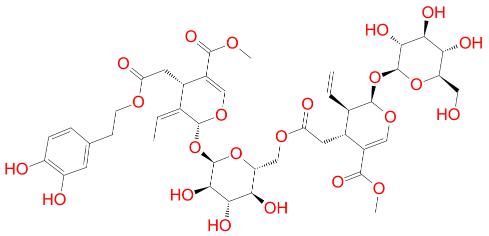

title P176_Isojaspolyoside

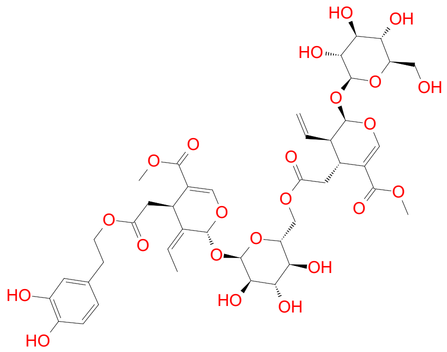

title P176_Isojaspolyoside

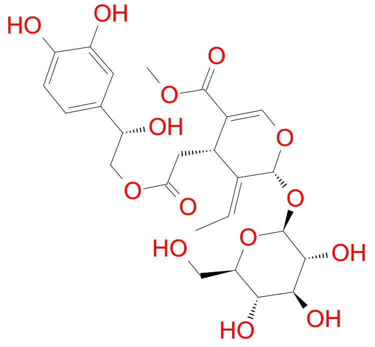

title P185_7-S-Hydroxyole

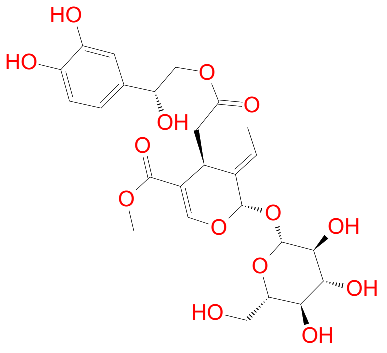

title P185_7-S-Hydroxyole

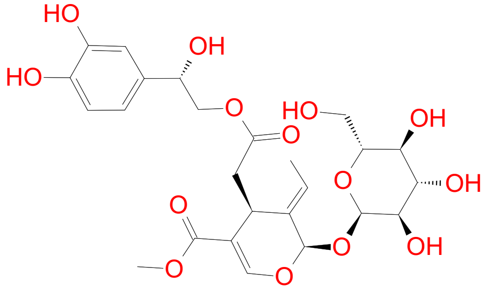

title P185_7-S-Hydroxyole

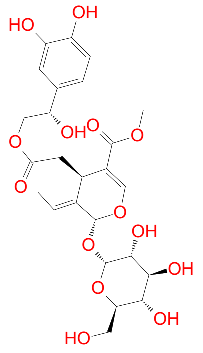

title P185_7-S-Hydroxyole

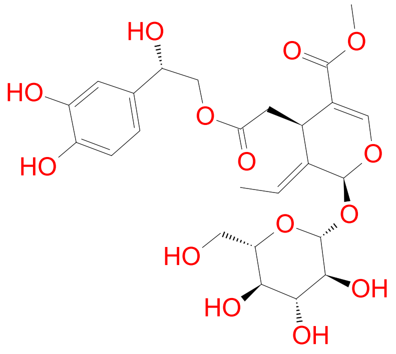

title P185_7-S-Hydroxyole

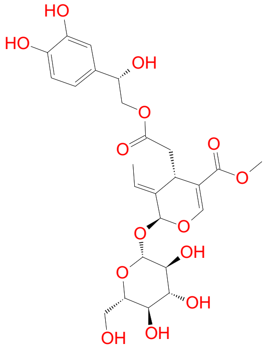

title P185_7-S-Hydroxyole

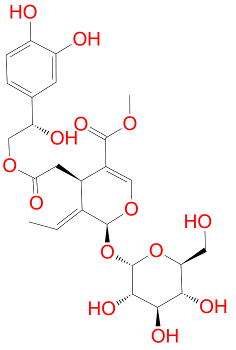

title P185_7-S-Hydroxyole

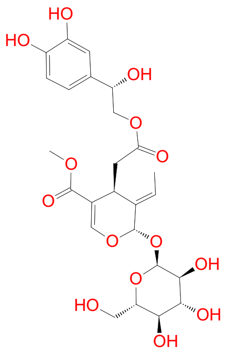

title P185_7-S-Hydroxyole

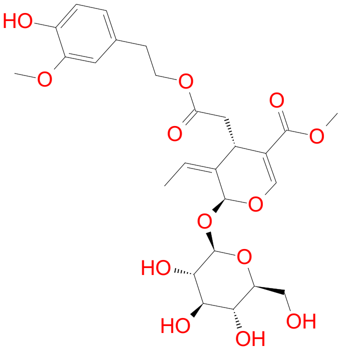

title P190_Oleuropein-3-M

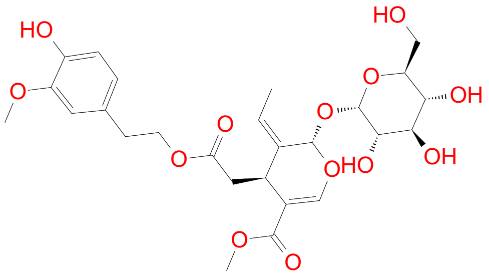

title P190_Oleuropein-3-M

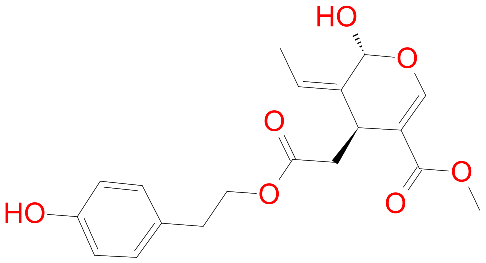

title P192_Ligstroside agl

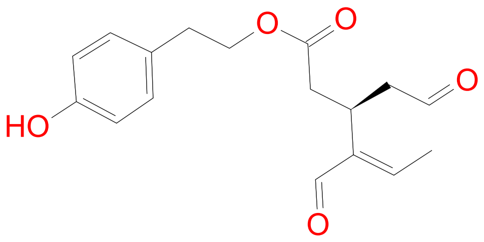

title P197_Decarboxymet

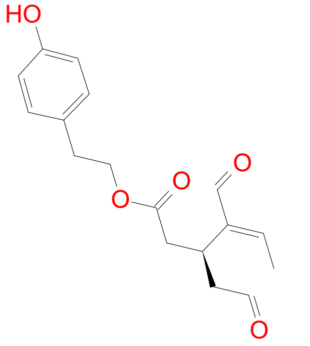

title P197_Decarboxymet

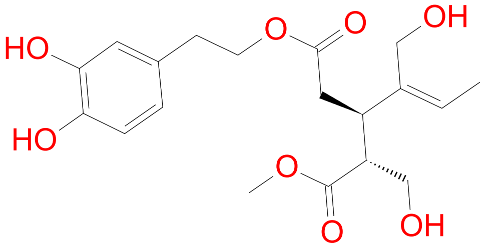

title P198_Hydroxytyroso

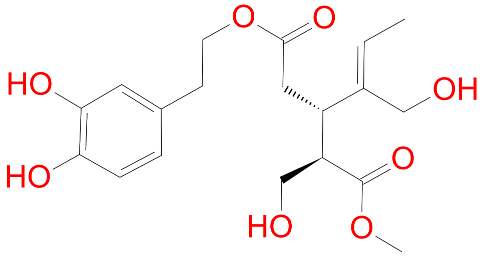

title P198_Hydroxytyroso

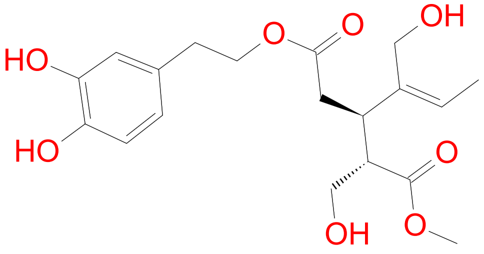

title P198_Hydroxytyroso

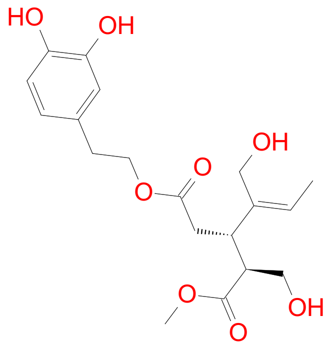

title P198_Hydroxytyroso

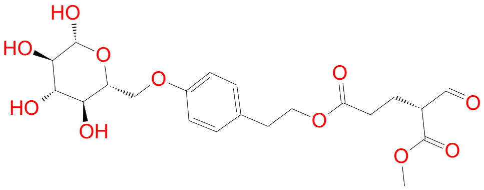

title P213_Ligstroside der

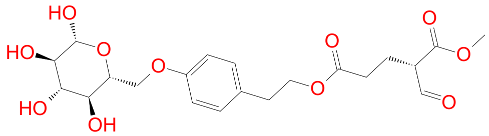

title P213_Ligstroside der

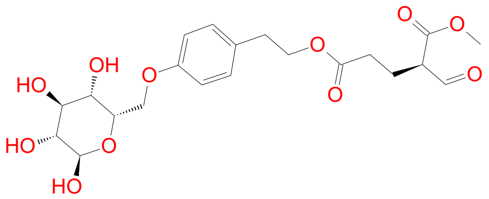

title P213_Ligstroside der

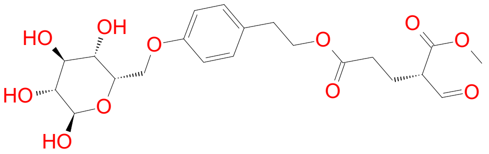

title P213_Ligstroside der

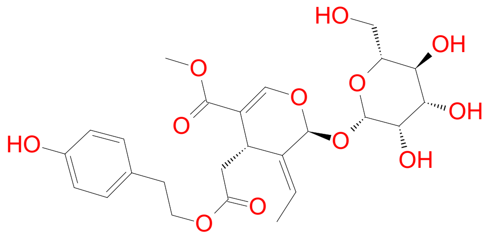

title P217_Ligstroside.cdx

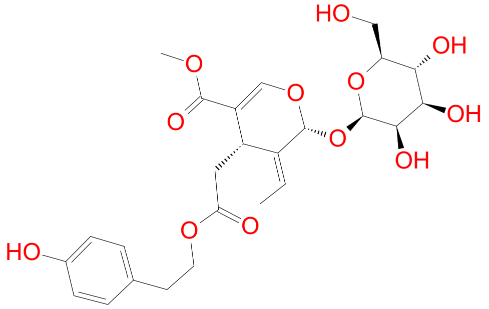

title P217_Ligstroside.cdx

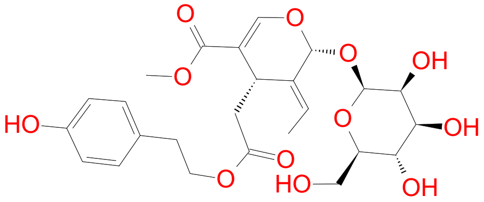

title P217_Ligstroside.cdx

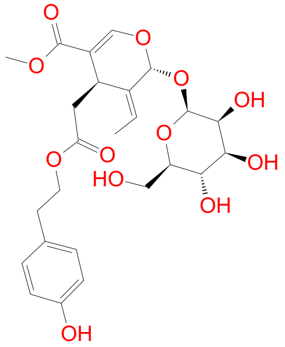

title P217_Ligstroside.cdx

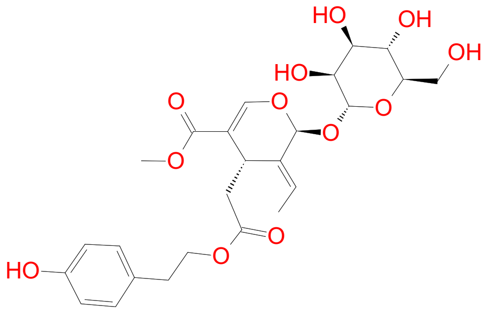

title P217_Ligstroside.cdx

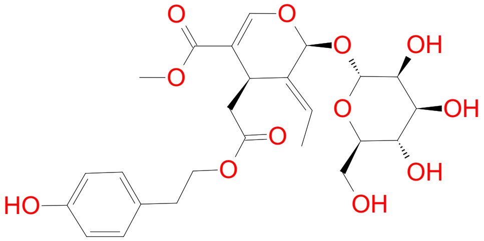

title P217_Ligstroside.cdx

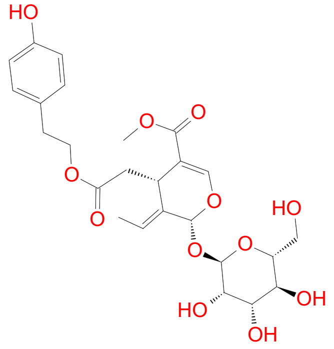

title P217_Ligstroside.cdx

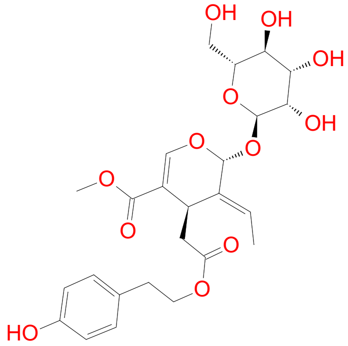

title P217_Ligstroside.cdx

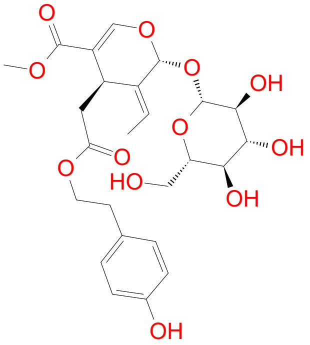

title P217_Ligstroside.cdx

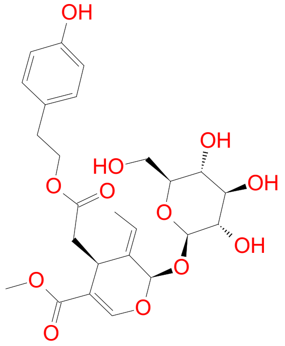

title P217_Ligstroside.cdx

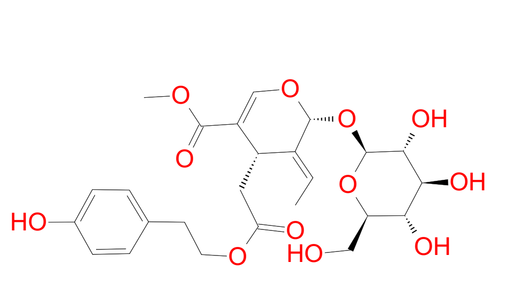

title P217_Ligstroside.cdx

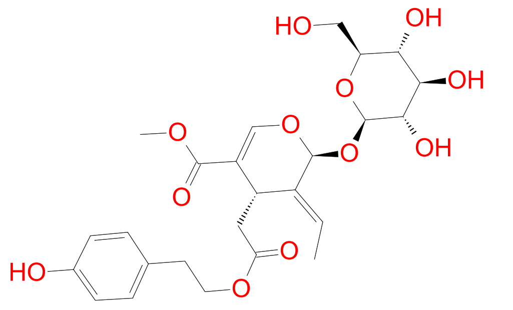

title P217_Ligstroside.cdx

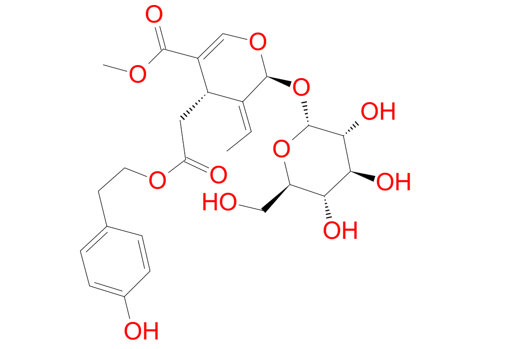

title P217_Ligstroside.cdx

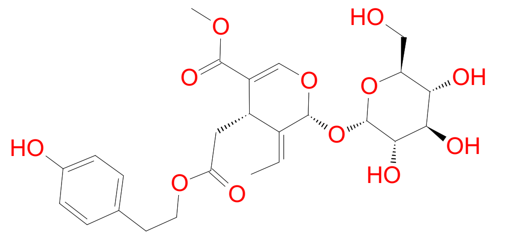

title P217_Ligstroside.cdx

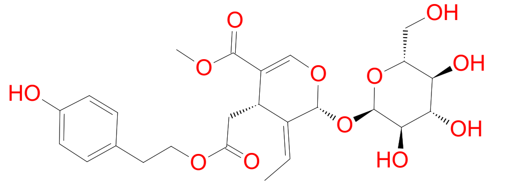

title P217_Ligstroside.cdx

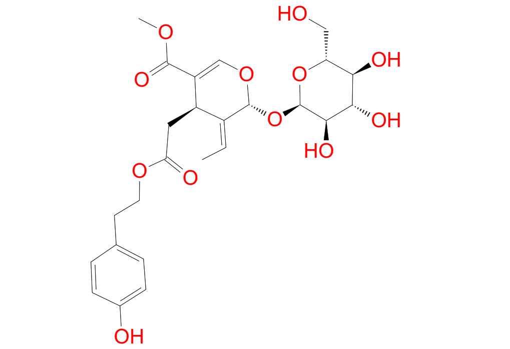

title P217_Ligstroside.cdx

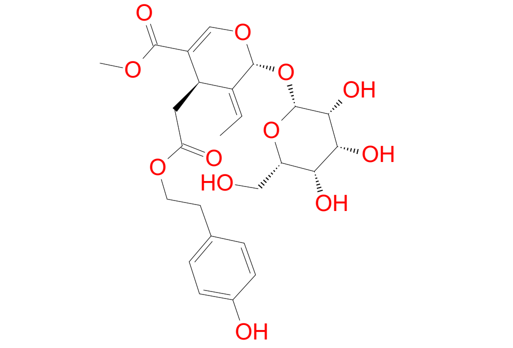

title P217_Ligstroside.cdx

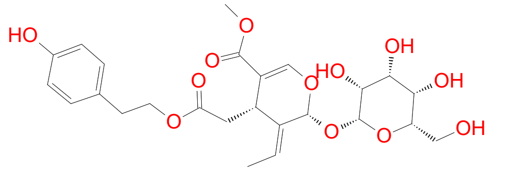

title P217_Ligstroside.cdx

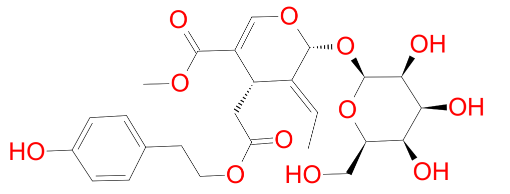

title P217_Ligstroside.cdx

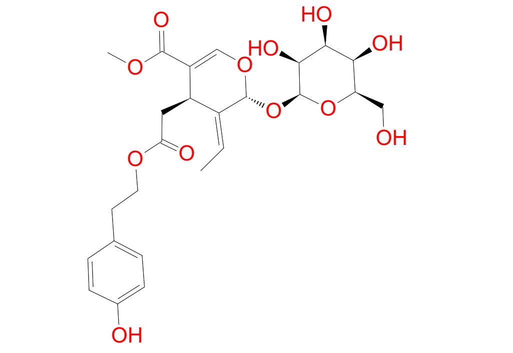

title P217_Ligstroside.cdx

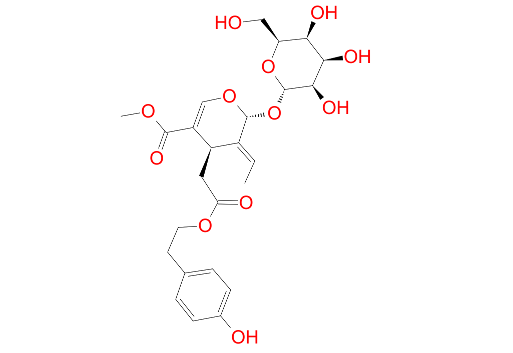

title P217_Ligstroside.cdx

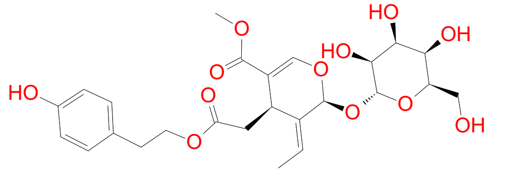

title P217_Ligstroside.cdx

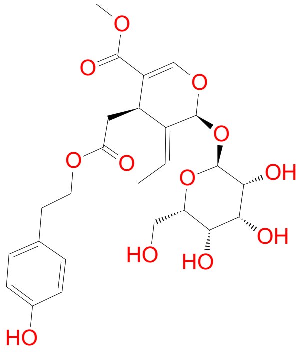

title P217_Ligstroside.cdx

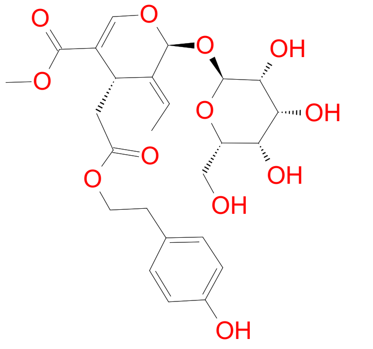

title P217_Ligstroside.cdx

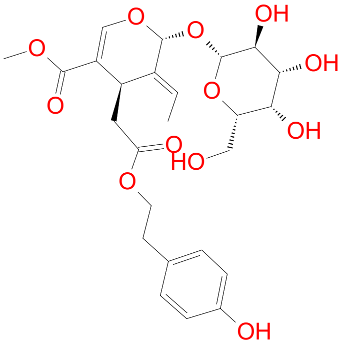

title P217_Ligstroside.cdx

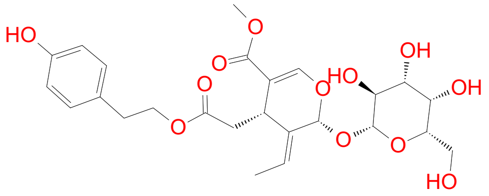

title P217_Ligstroside.cdx

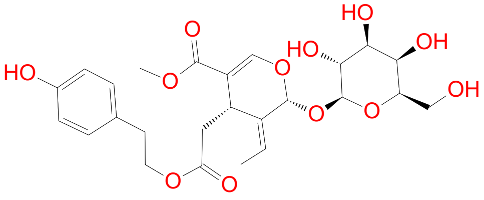

title P217_Ligstroside.cdx

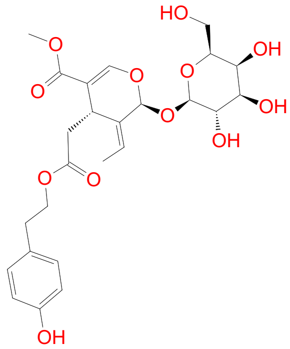

title P217_Ligstroside.cdx

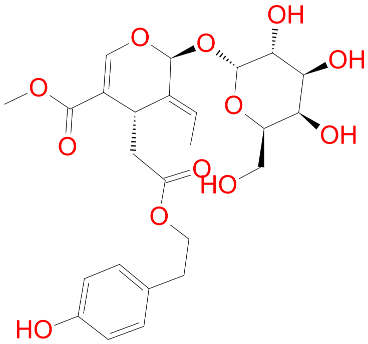

title P217_Ligstroside.cdx

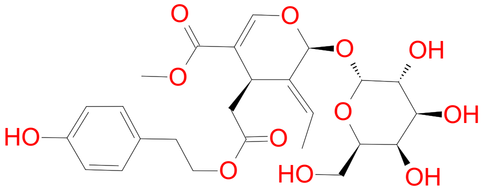

title P217_Ligstroside.cdx

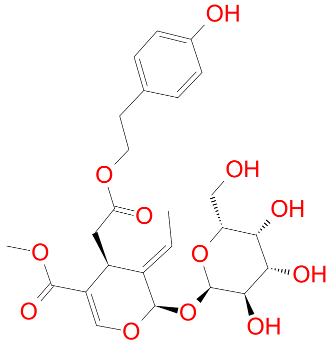

title P217_Ligstroside.cdx

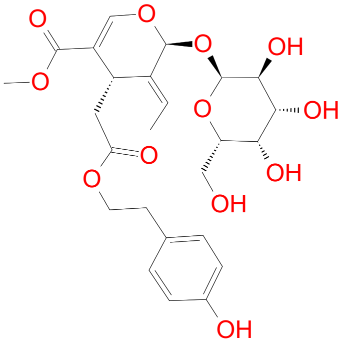

title P217_Ligstroside.cdx

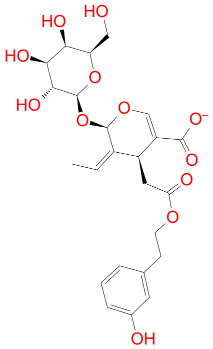

title P219_Demethylligstr

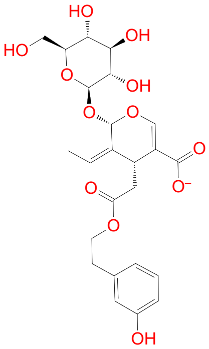

title P219_Demethylligstr

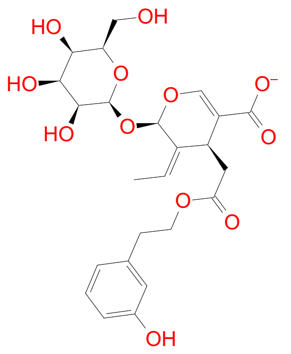

title P219_Demethylligstr

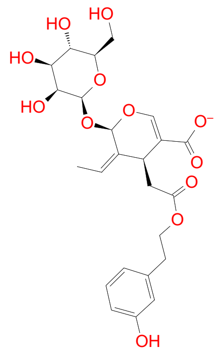

title P219_Demethylligstr

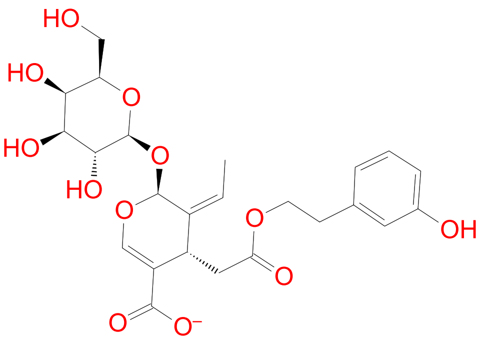

title P219_Demethylligstr

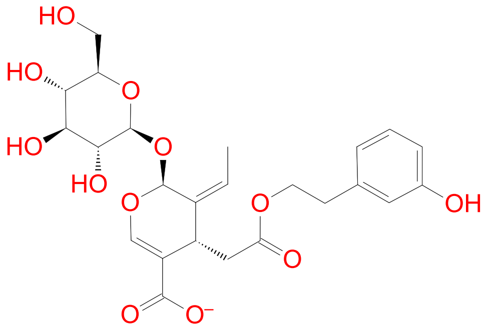

title P219_Demethylligstr

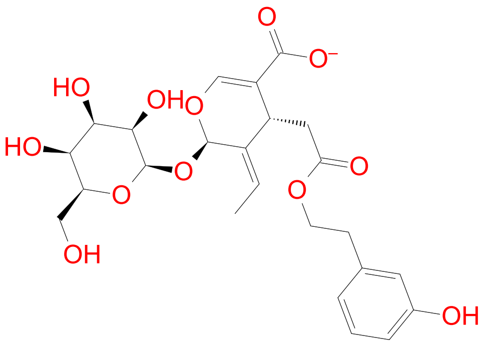

title P219_Demethylligstr

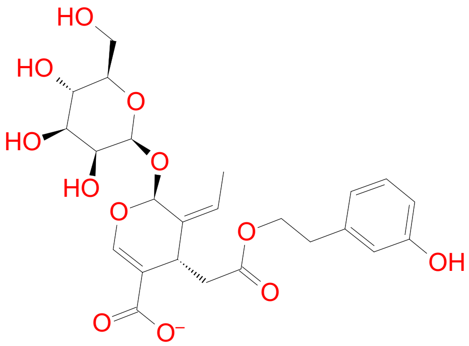

title P219_Demethylligstr

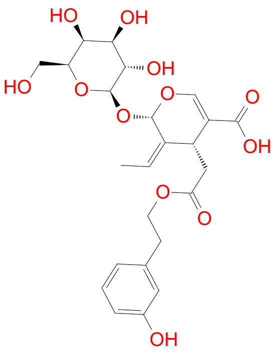

title P219_Demethylligstr

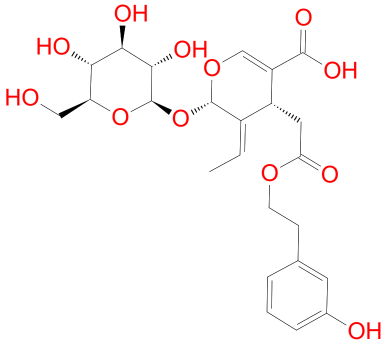

title P219_Demethylligstr

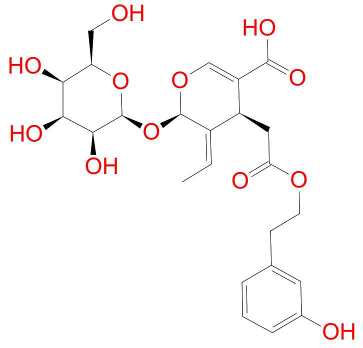

title P219_Demethylligstr

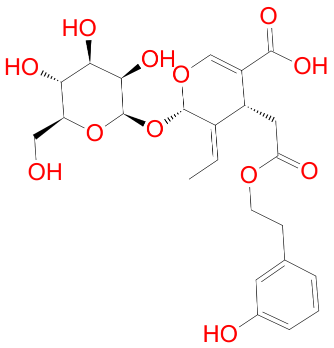

title P219_Demethylligstr

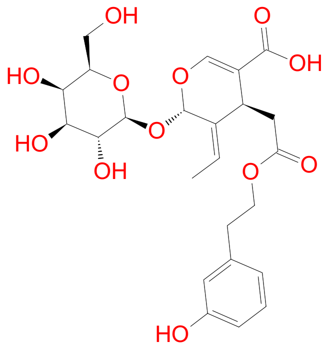

title P219_Demethylligstr

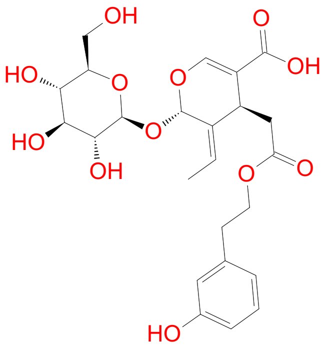

title P219_Demethylligstr

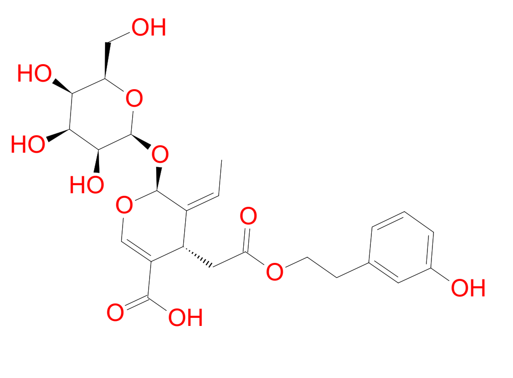

title P219_Demethylligstr

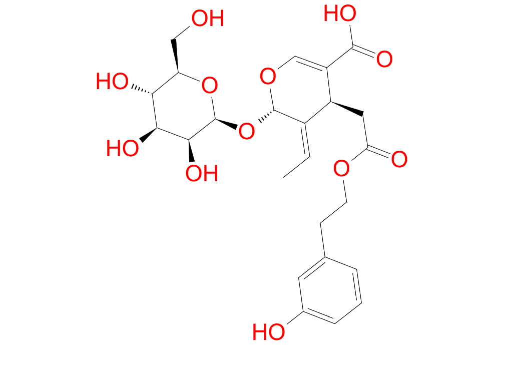

title P219_Demethylligstr

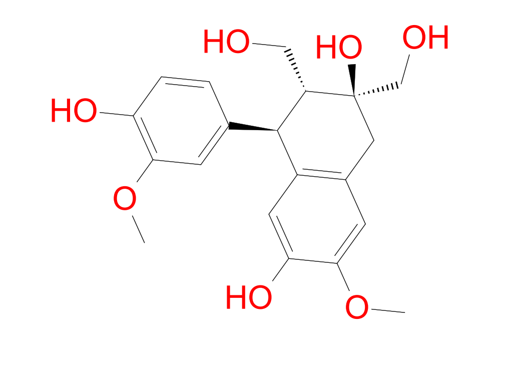

title P222_(+)-Cycloolivil.

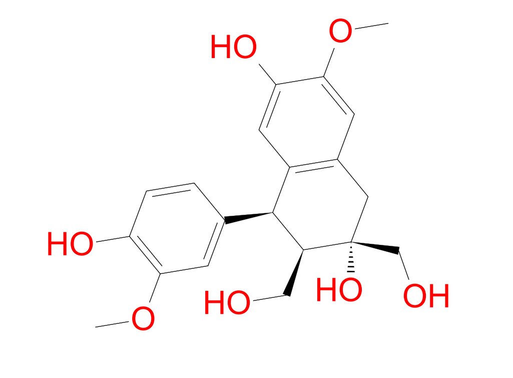

title P222_(+)-Cycloolivil.

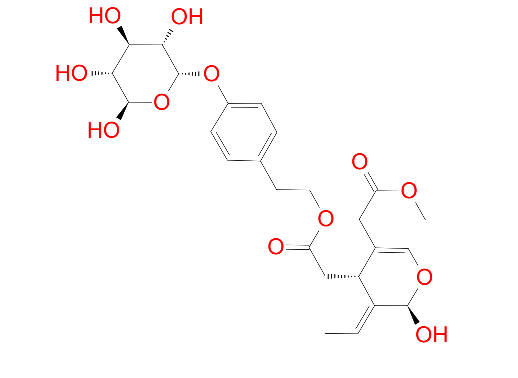

title P227_Ligstroside der

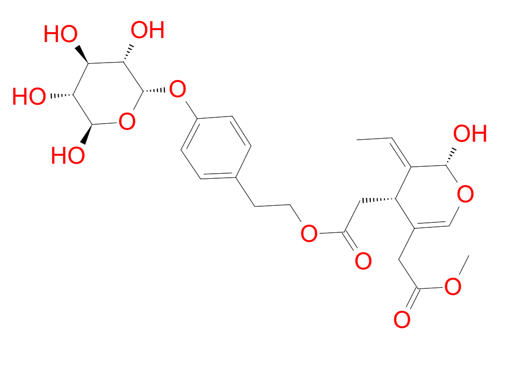

title P227_Ligstroside der

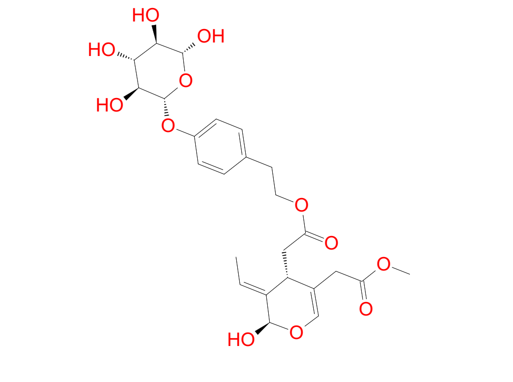

title P227_Ligstroside der

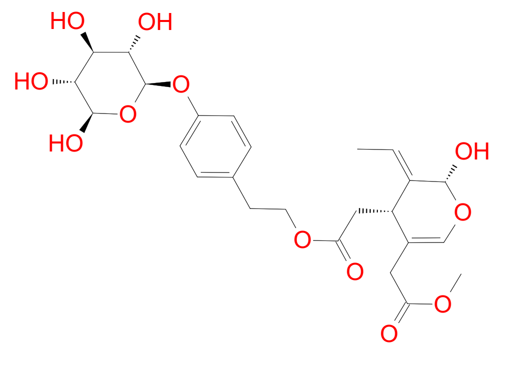

title P227_Ligstroside der

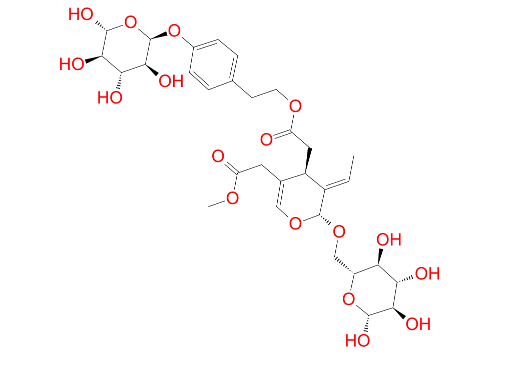

title P229_Ligstroside der

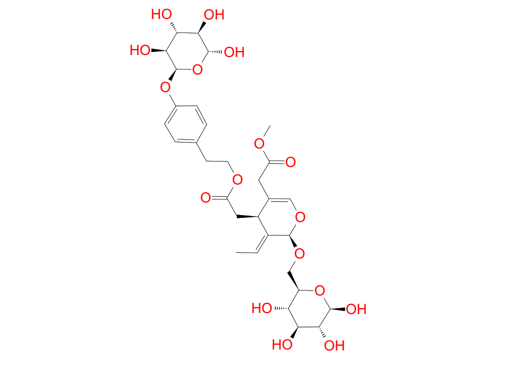

title P229_Ligstroside der

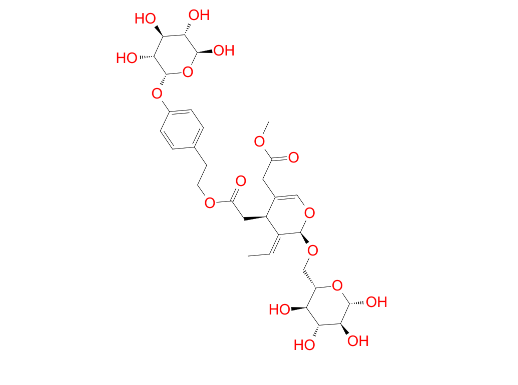

title P229_Ligstroside der

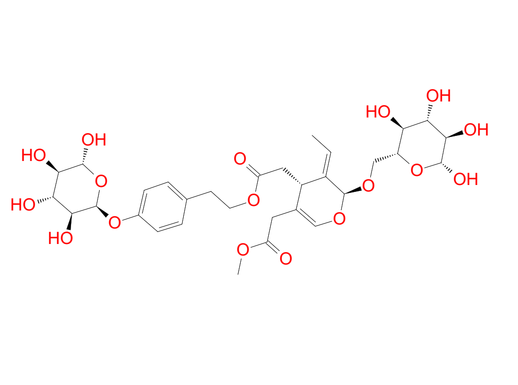

title P229_Ligstroside der

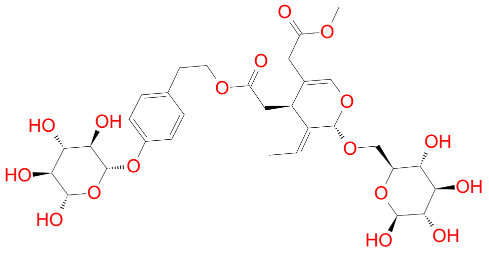

title P229_Ligstroside der

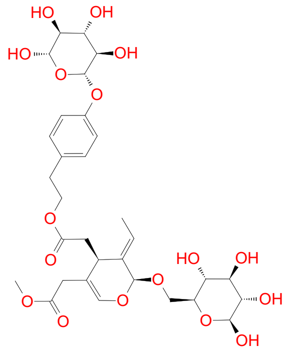

title P229_Ligstroside der

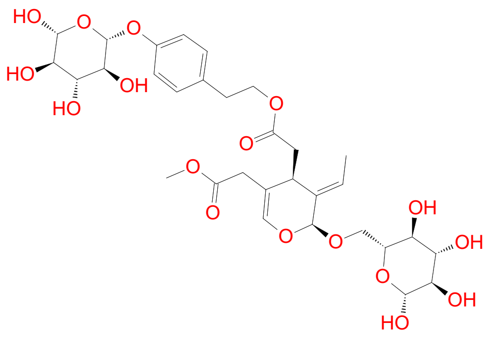

title P229_Ligstroside der

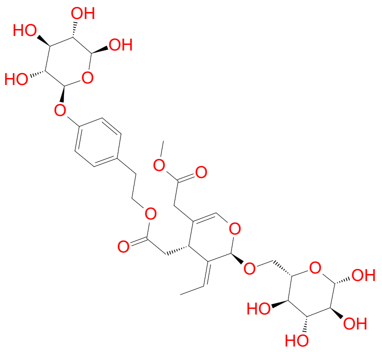

title P229_Ligstroside der

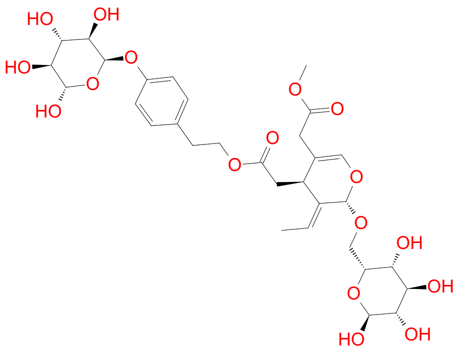

title P229_Ligstroside der

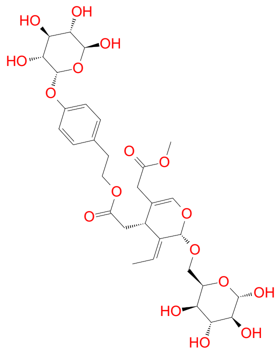

title P229_Ligstroside der

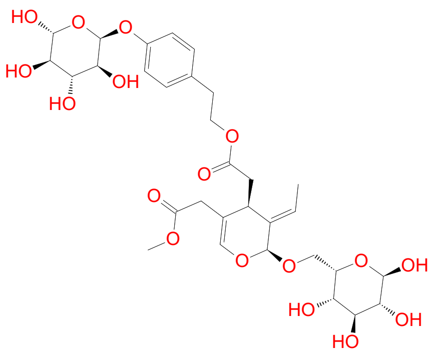

title P229_Ligstroside der

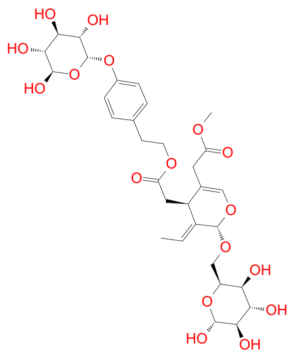

title P229_Ligstroside der

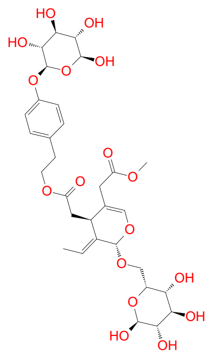

title P229_Ligstroside der

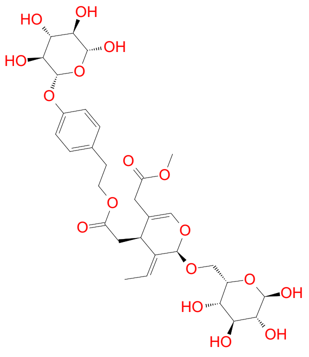

title P229_Ligstroside der

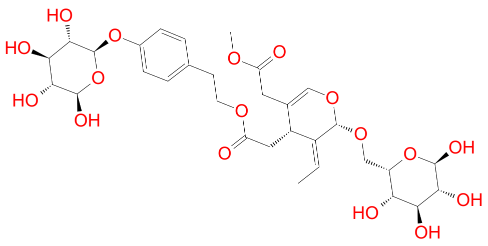

title P229_Ligstroside der

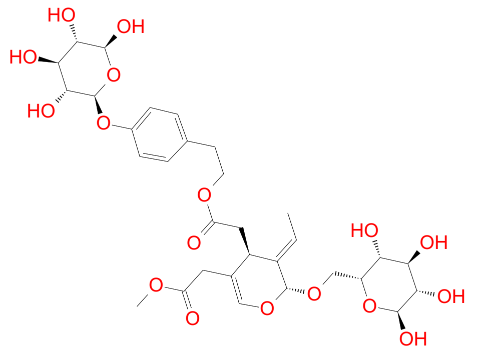

title P229_Ligstroside der

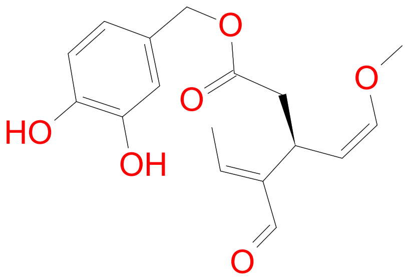

title P230_Hemiacetal of

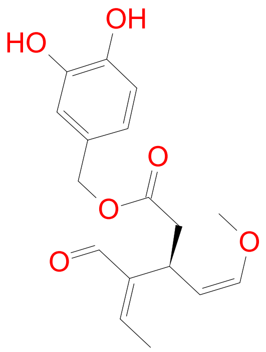

title P230_Hemiacetal of

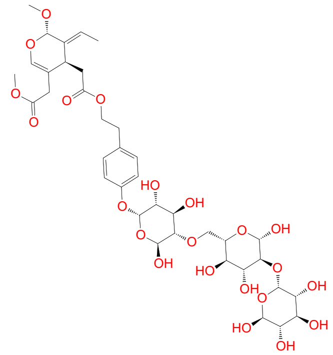

title P230_Ligstroside der

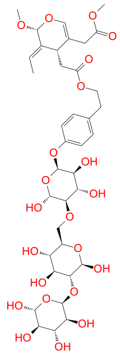

title P230_Ligstroside der

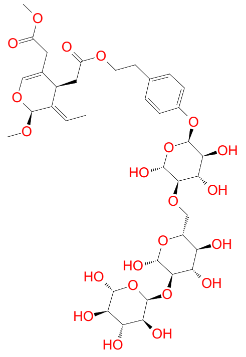

title P230_Ligstroside der

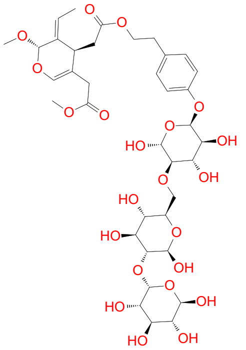

title P230_Ligstroside der

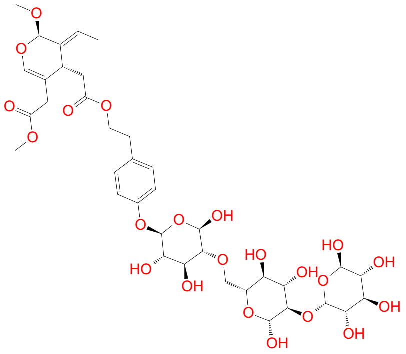

title P230_Ligstroside der

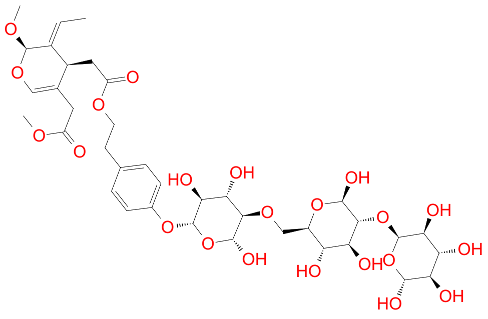

title P230_Ligstroside der

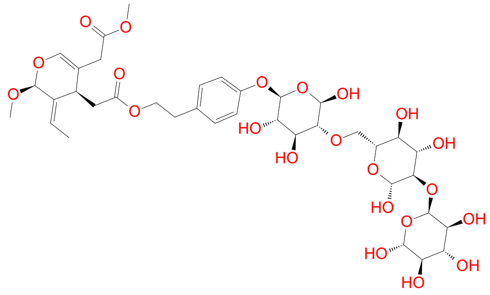

title P230_Ligstroside der

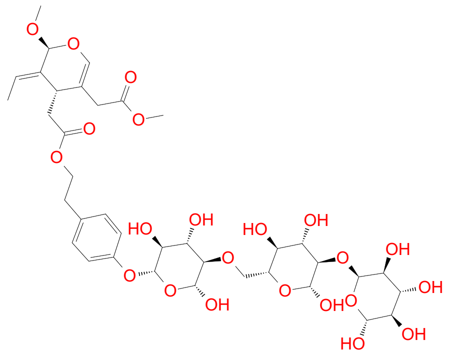

title P230_Ligstroside der

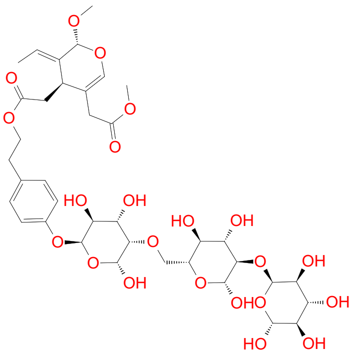

title P230_Ligstroside der

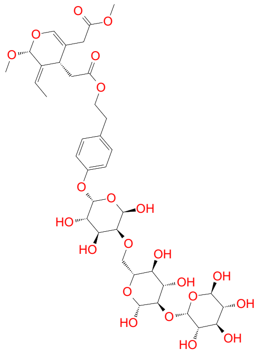

title P230_Ligstroside der

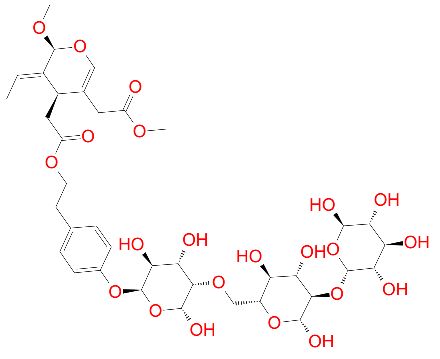

title P230_Ligstroside der

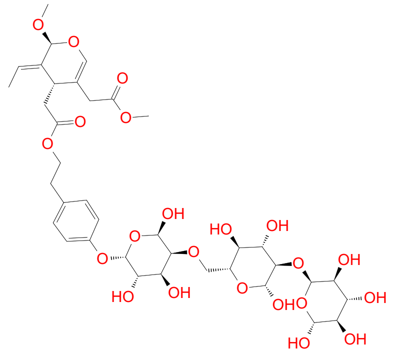

title P230_Ligstroside der

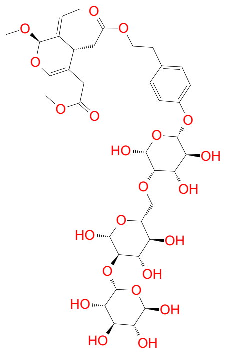

title P230_Ligstroside der

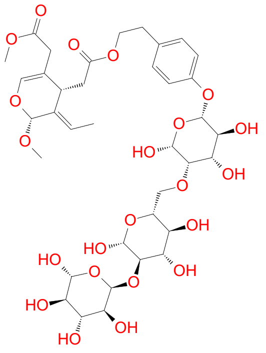

title P230_Ligstroside der

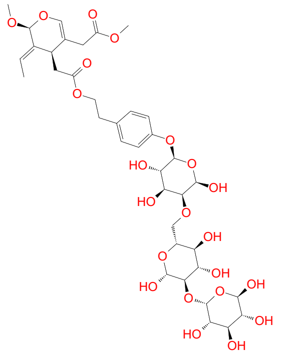

title P230_Ligstroside der

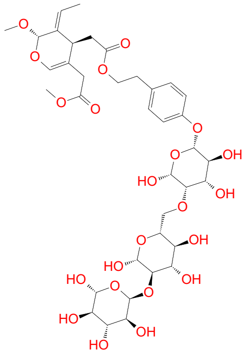

title P230_Ligstroside der

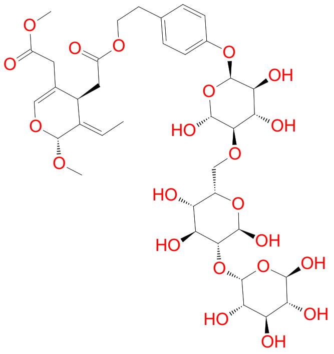

title P230_Ligstroside der

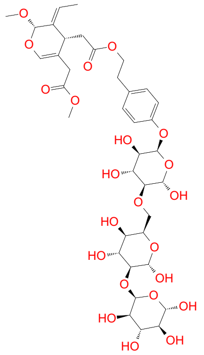

title P230_Ligstroside der

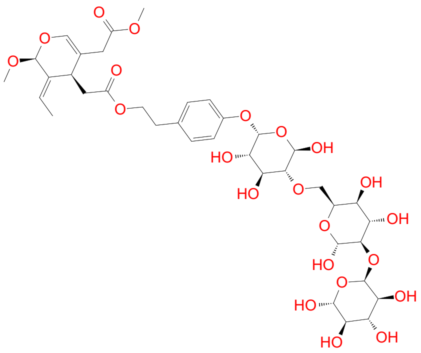

title P230_Ligstroside der

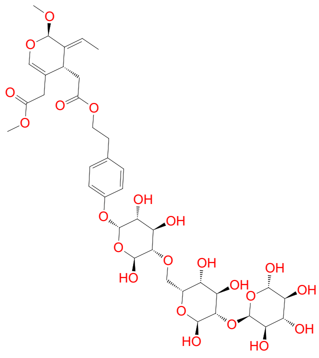

title P230_Ligstroside der

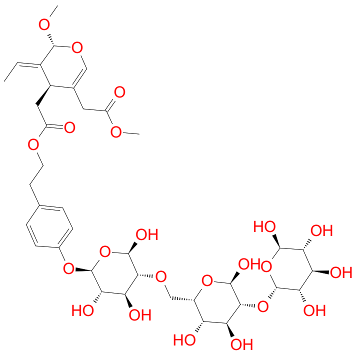

title P230_Ligstroside der

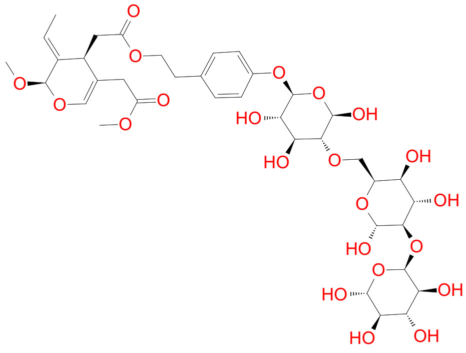

title P230_Ligstroside der

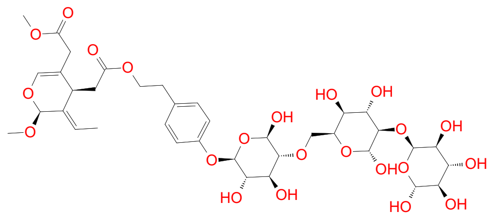

title P230_Ligstroside der

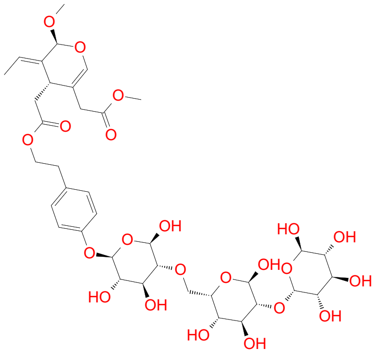

title P230_Ligstroside der

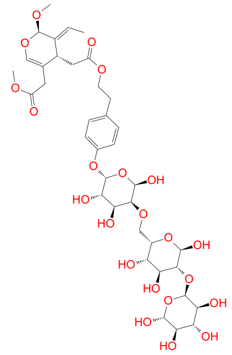

title P230_Ligstroside der

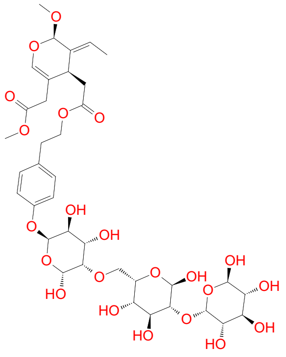

title P230_Ligstroside der

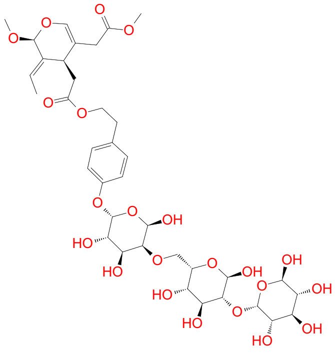

title P230_Ligstroside der

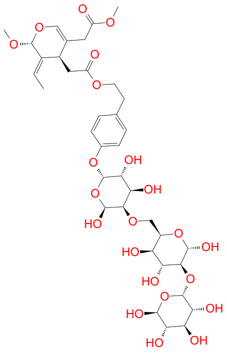

title P230_Ligstroside der

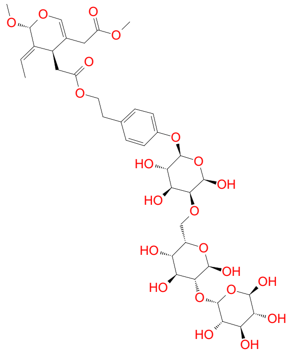

title P230_Ligstroside der

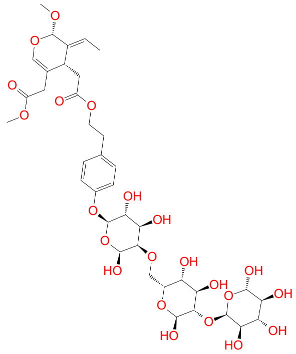

title P230_Ligstroside der

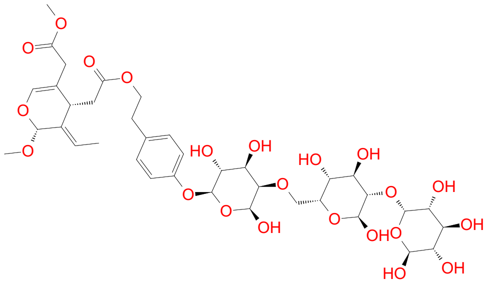

title P230_Ligstroside der

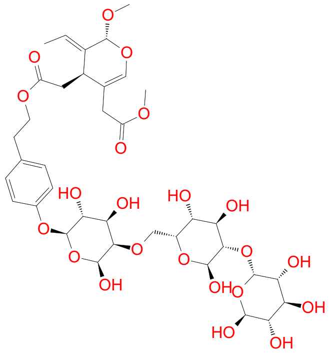

title P230_Ligstroside der

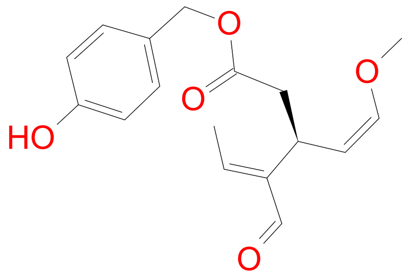

title P231_Hemiacetal of

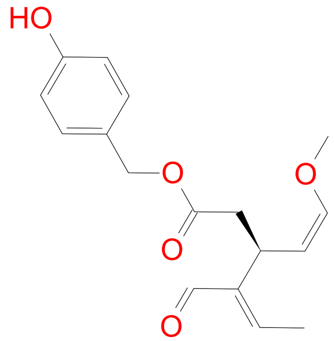

title P231_Hemiacetal of

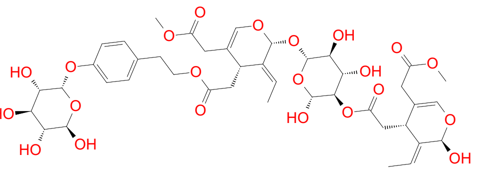

title P231_Ligstroside der

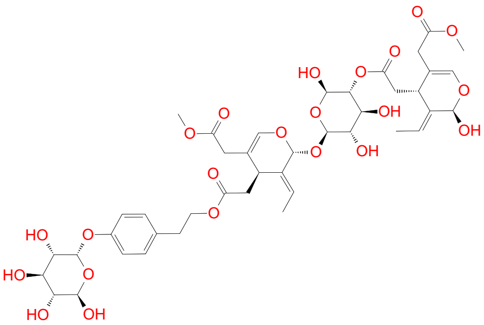

title P231_Ligstroside der

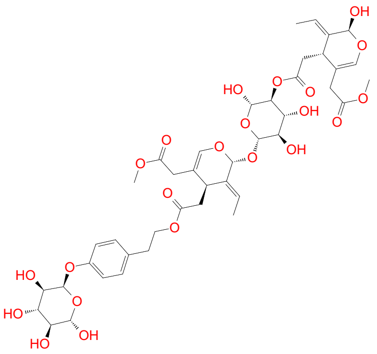

title P231_Ligstroside der

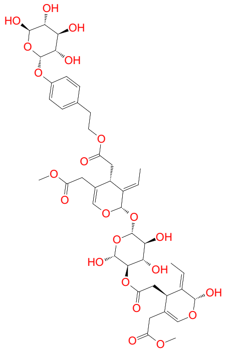

title P231_Ligstroside der

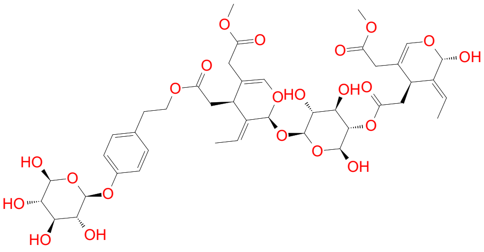

title P231_Ligstroside der

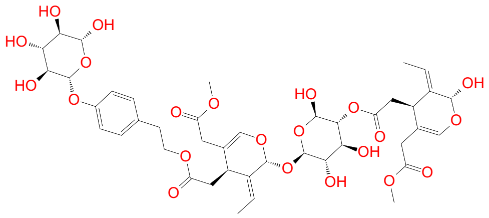

title P231_Ligstroside der

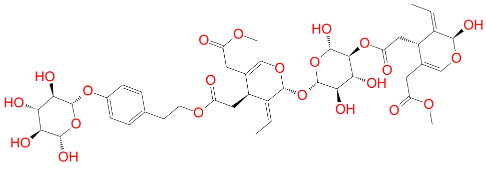

title P231_Ligstroside der

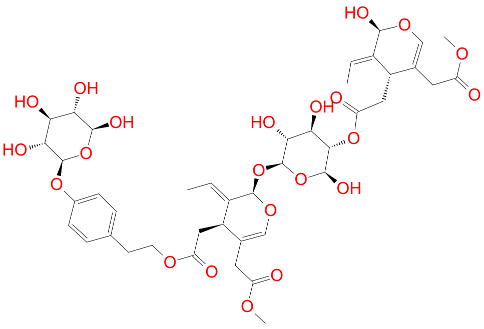

title P231_Ligstroside der

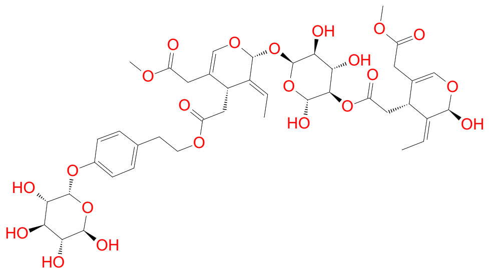

title P231_Ligstroside der

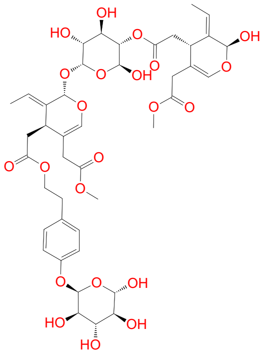

title P231_Ligstroside der

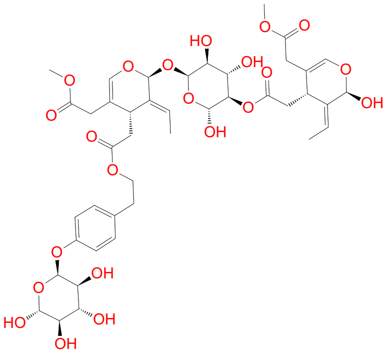

title P231_Ligstroside der

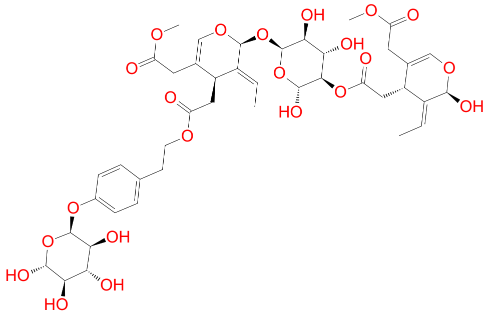

title P231_Ligstroside der

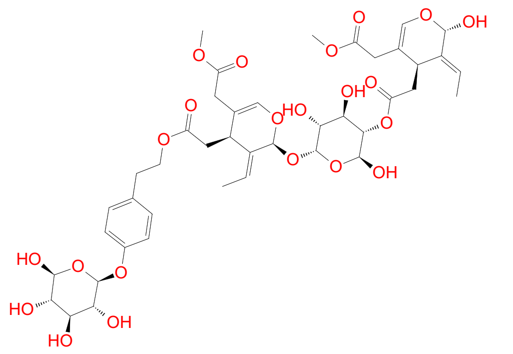

title P231_Ligstroside der

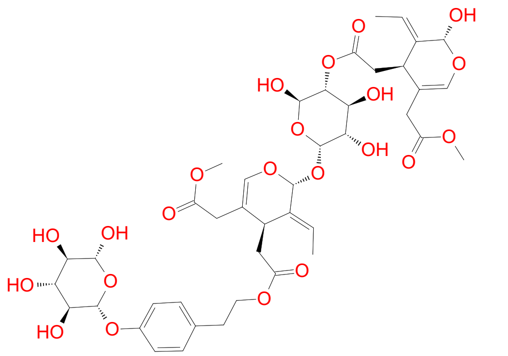

title P231_Ligstroside der

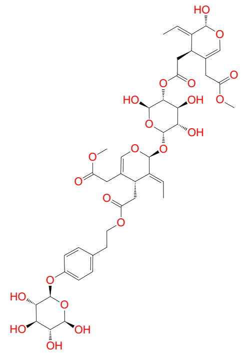

title P231_Ligstroside der

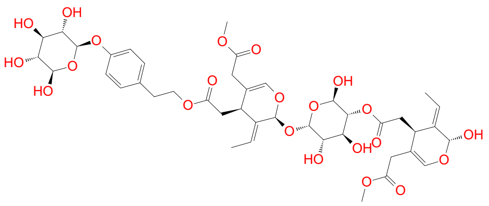

title P231_Ligstroside der

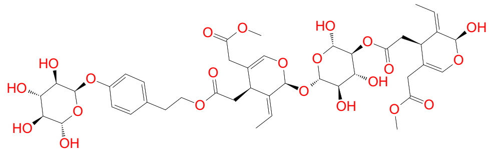

title P231_Ligstroside der

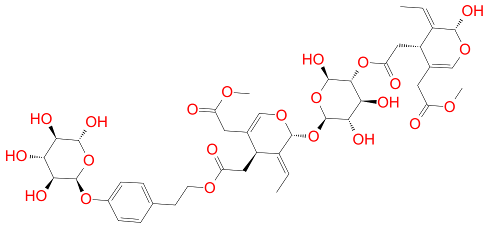

title P231_Ligstroside der

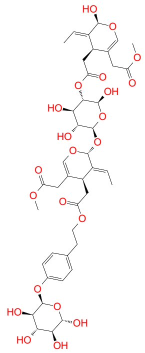

title P231_Ligstroside der

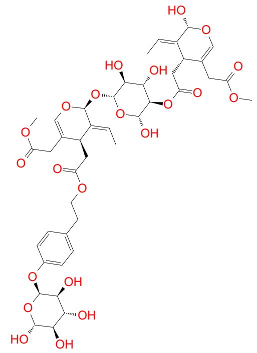

title P231_Ligstroside der

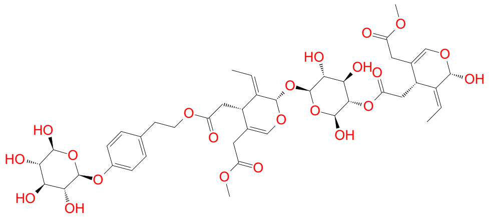

title P231_Ligstroside der

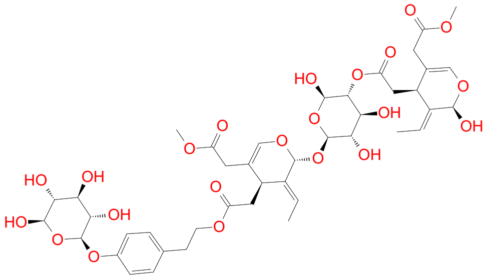

title P231_Ligstroside der

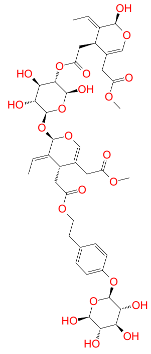

title P231_Ligstroside der

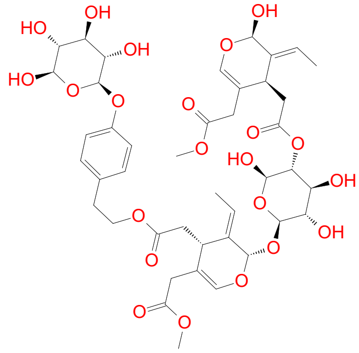

title P231_Ligstroside der

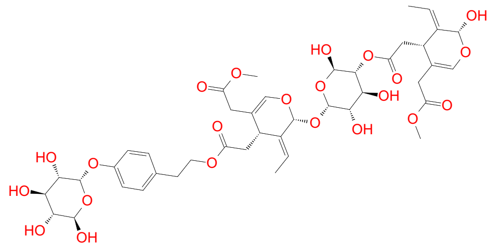

title P231_Ligstroside der

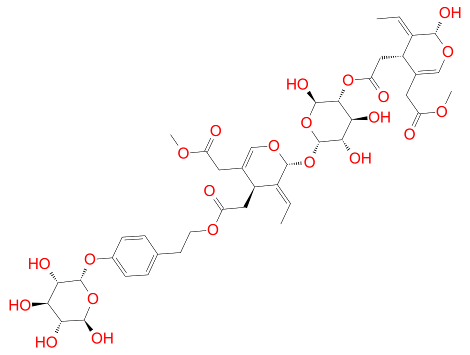

title P231_Ligstroside der

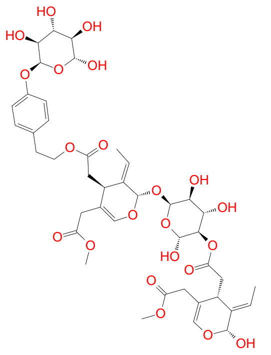

title P231_Ligstroside der

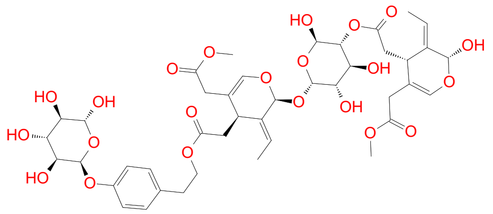

title P231_Ligstroside der

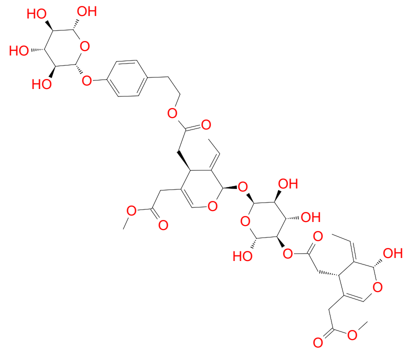

title P231_Ligstroside der

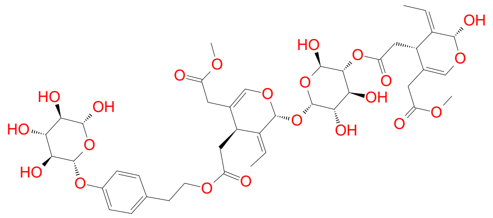

title P231_Ligstroside der

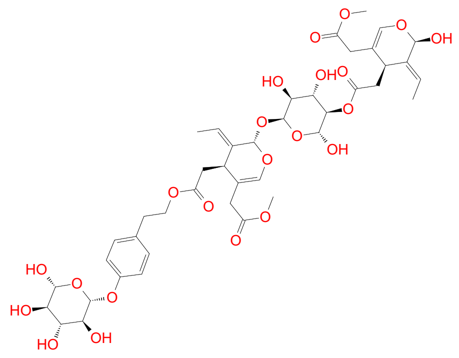

title P231_Ligstroside der

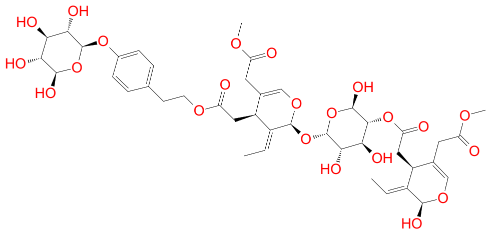

title P231_Ligstroside der

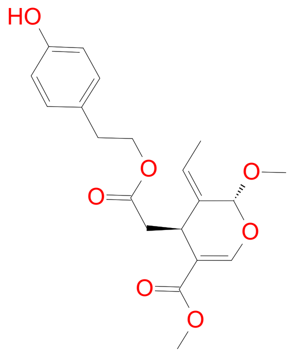

title P234_Ligstroside agl

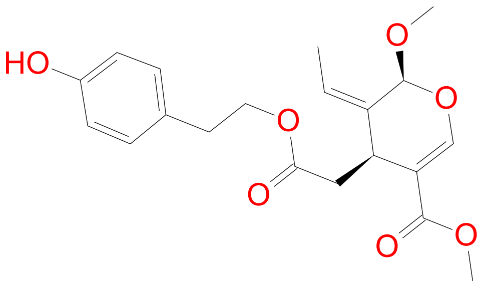

title P234_Ligstroside agl

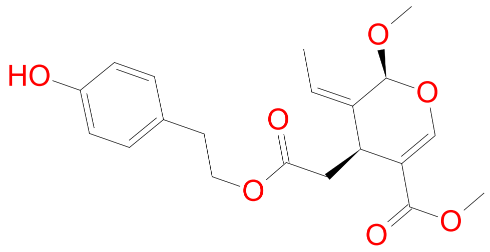

title P234_Ligstroside agl

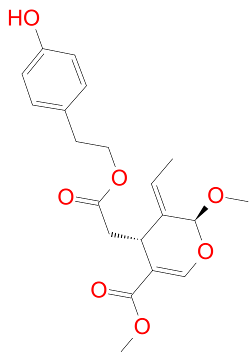

title P234_Ligstroside agl
