# Supplementary figures and images for: Molecular Docking and Molecular Dynamics Aided Virtual Search of OliveNet™ Directory for Secoiridoids to Combat SARS-CoV-2 Infection and Associated Hyperinflammatory Responses
Source: Front Mol Biosci. 2021 Jan 7;7:627767. doi: 10.3389/fmolb.2020.627767 (PMC7817976; doi:10.3389/fmolb.2020.627767)

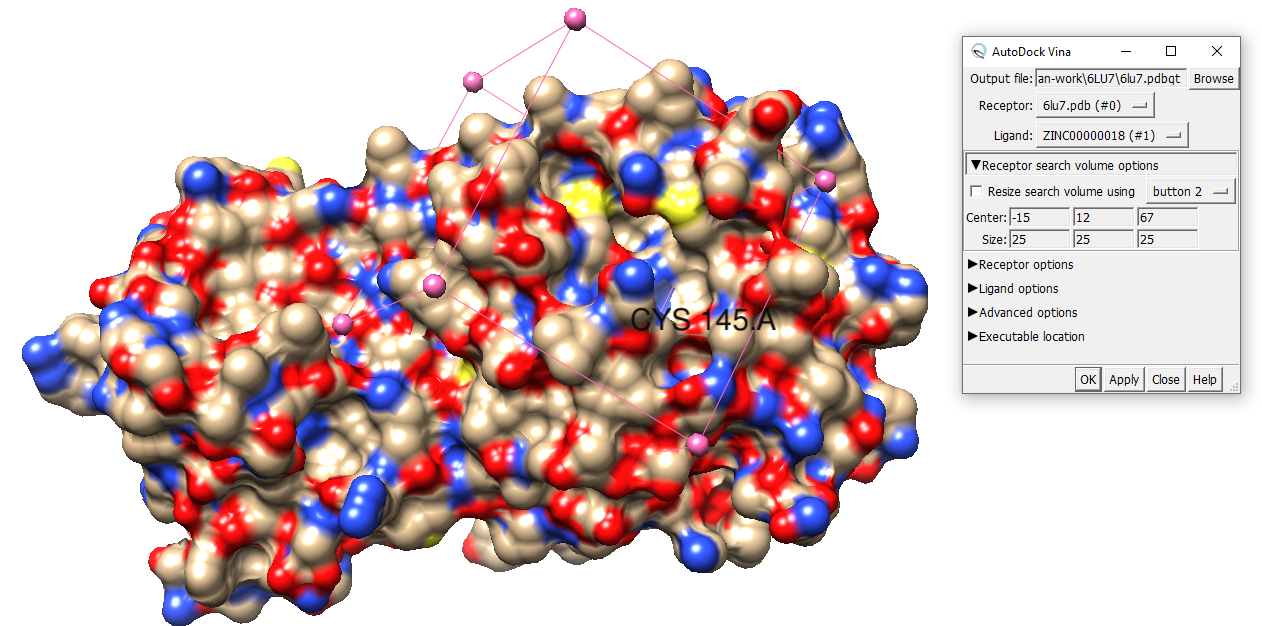

Supplement: Supplementary file 11 [file Image_1.PNG]

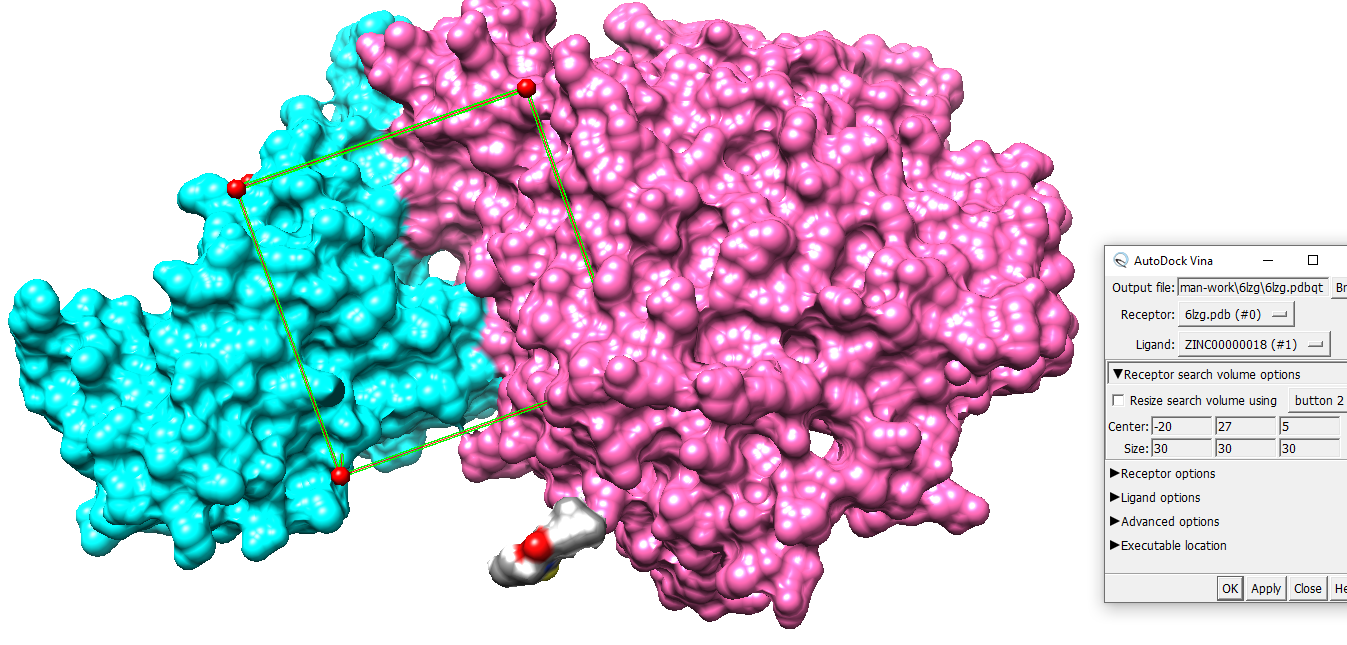

Supplement: Supplementary file 12 [file Image_2.PNG]

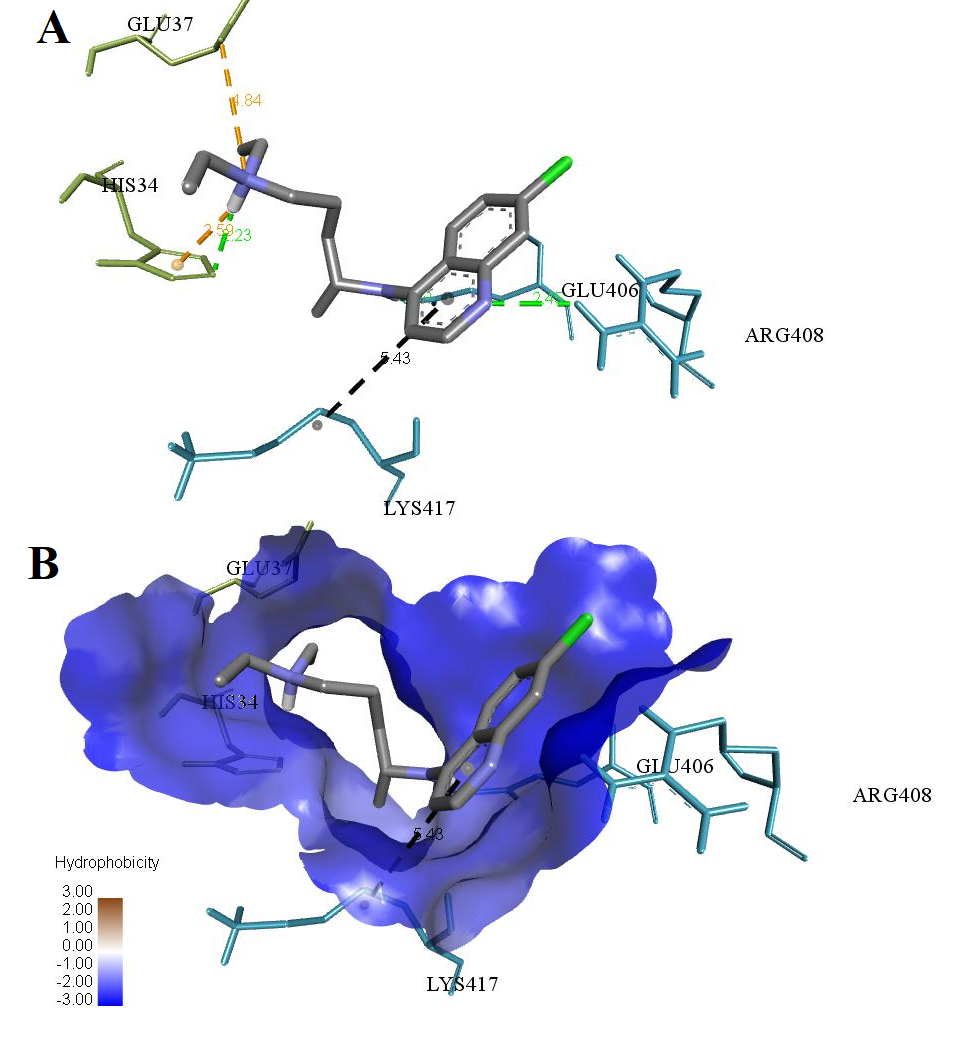

Supplement: Supplementary file 13 [file Image_3.TIF]

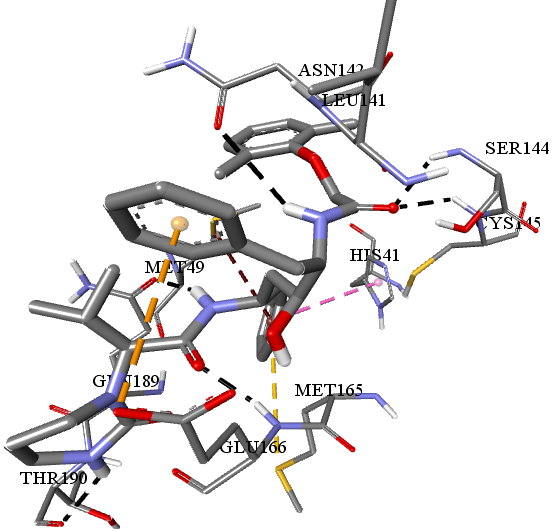

Supplement: Supplementary file 14 [file Image_4.TIF]

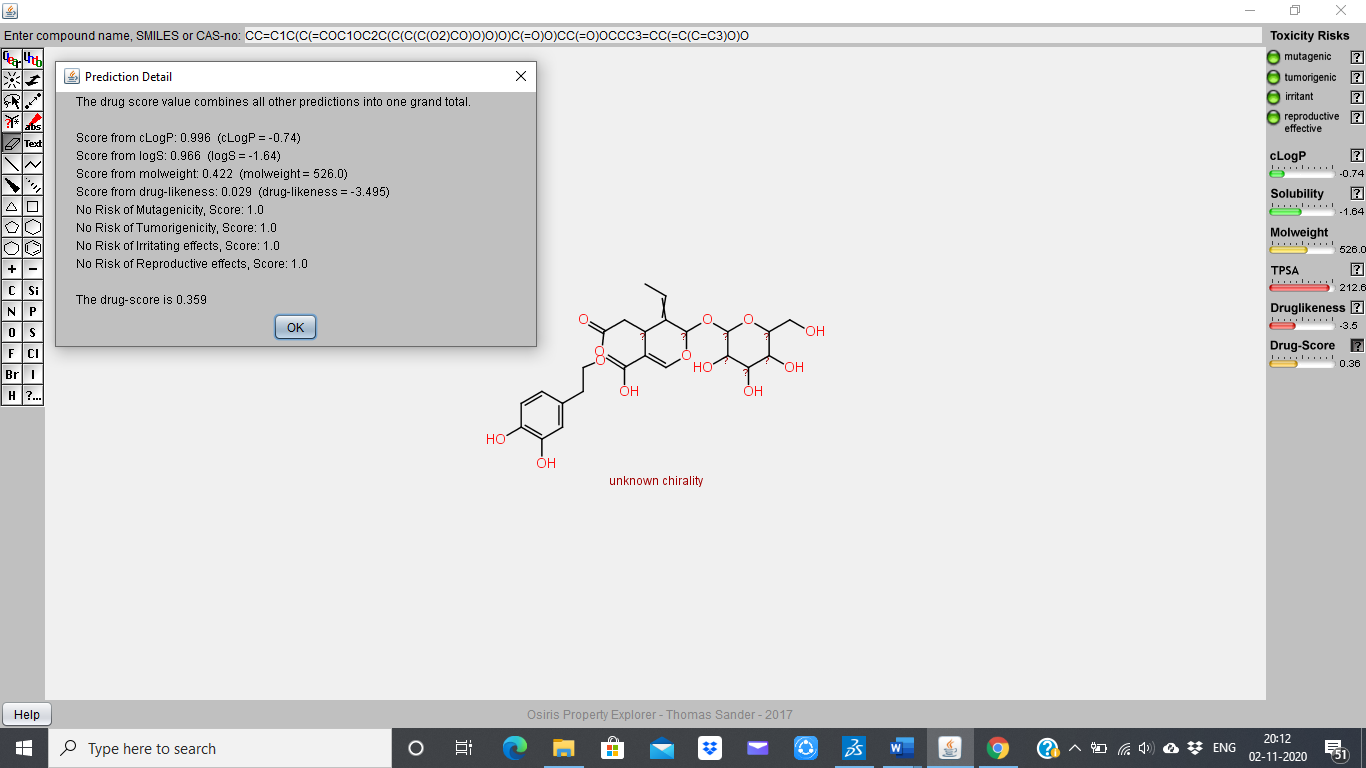

Supplement: Supplementary file 15 [file Image_5.PNG]

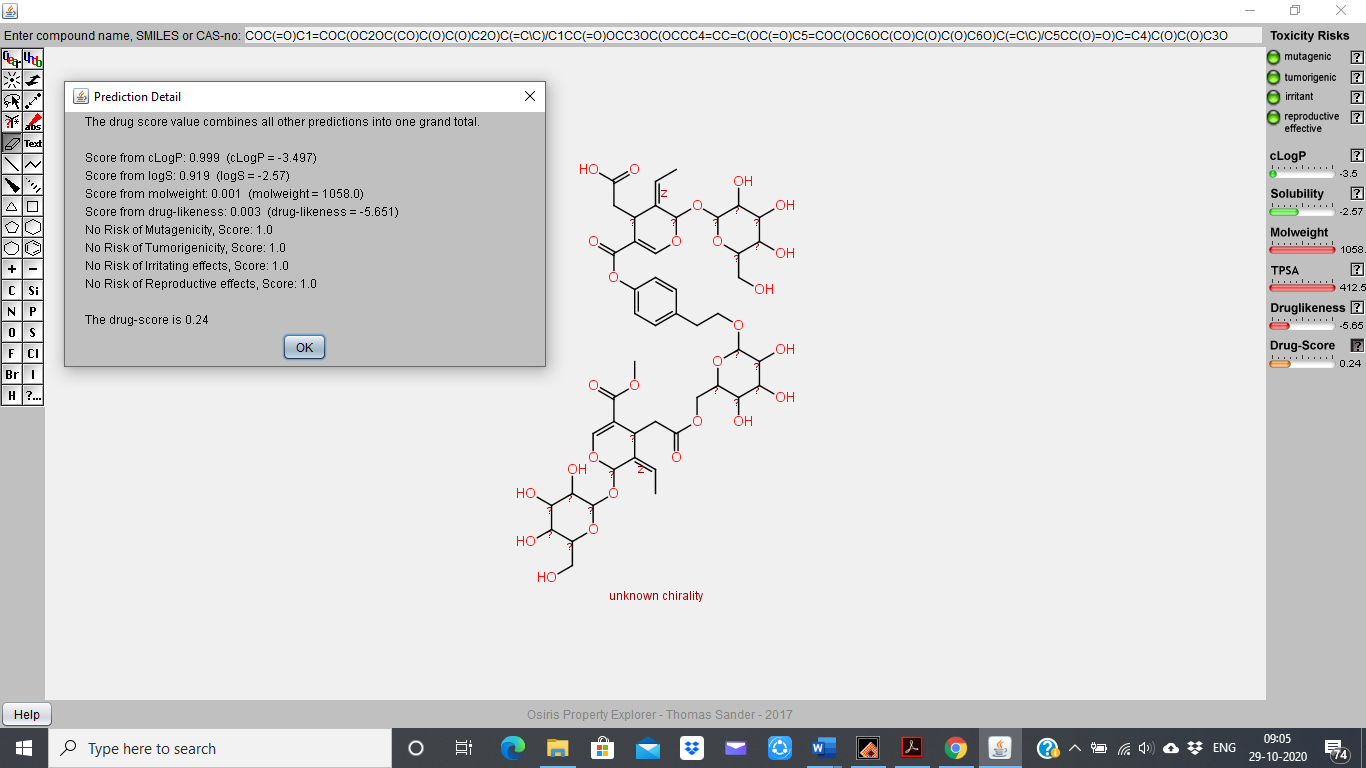

Supplement: Supplementary file 16 [file Image_6.PNG]

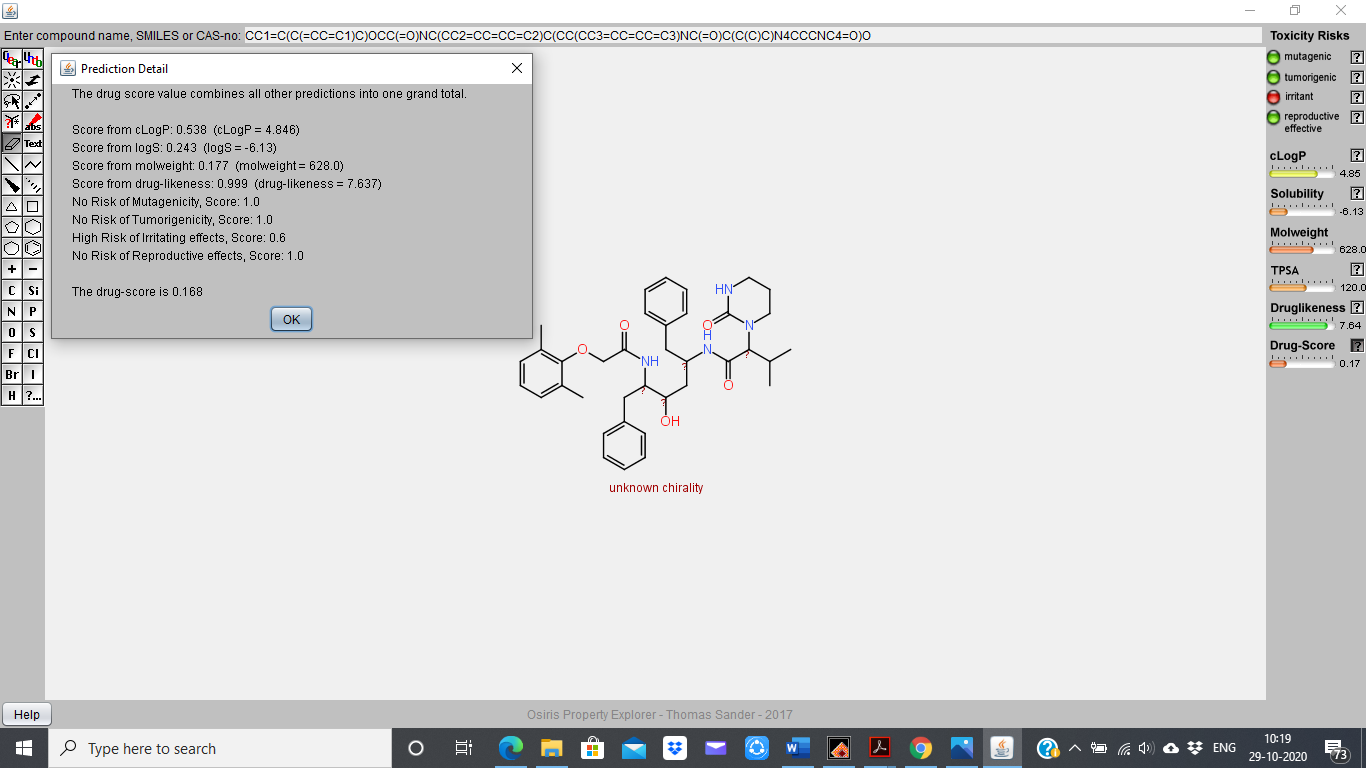

Supplement: Supplementary file 17 [file Image_7.PNG]

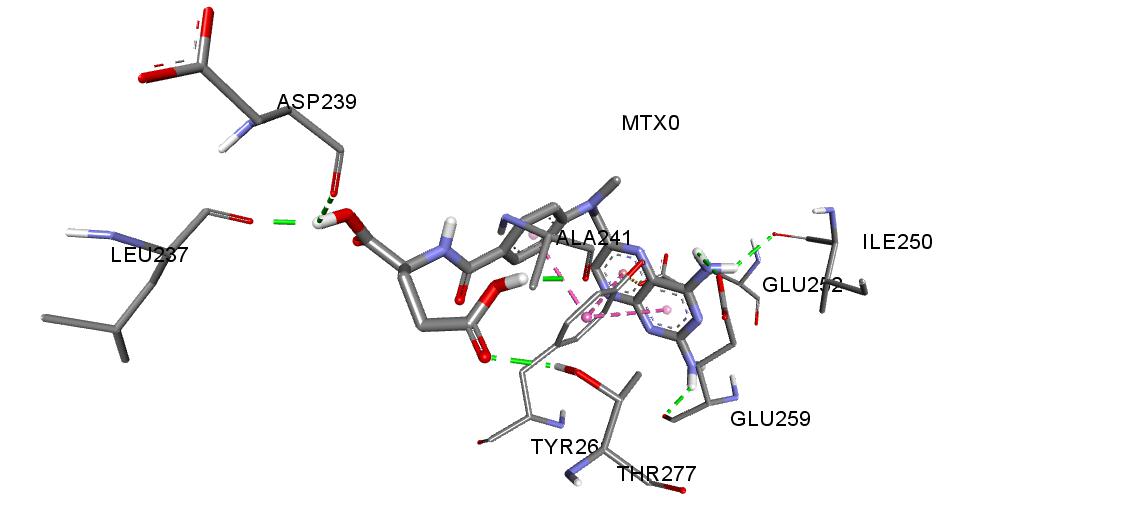

Supplement: Supplementary file 18 [file Image_8.JPEG]

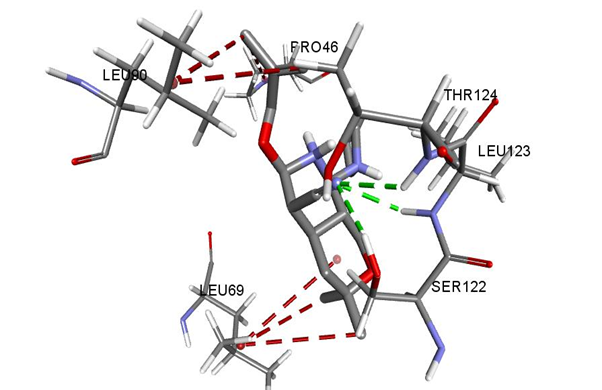

Supplement: Supplementary file 19 [file Image_9.tif]

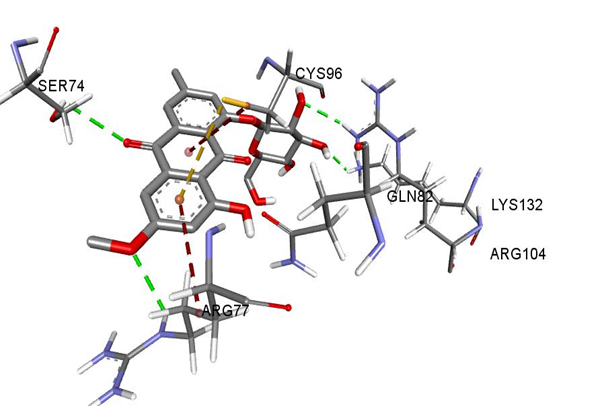

Supplement: Supplementary file 20 [file Image_10.tif]

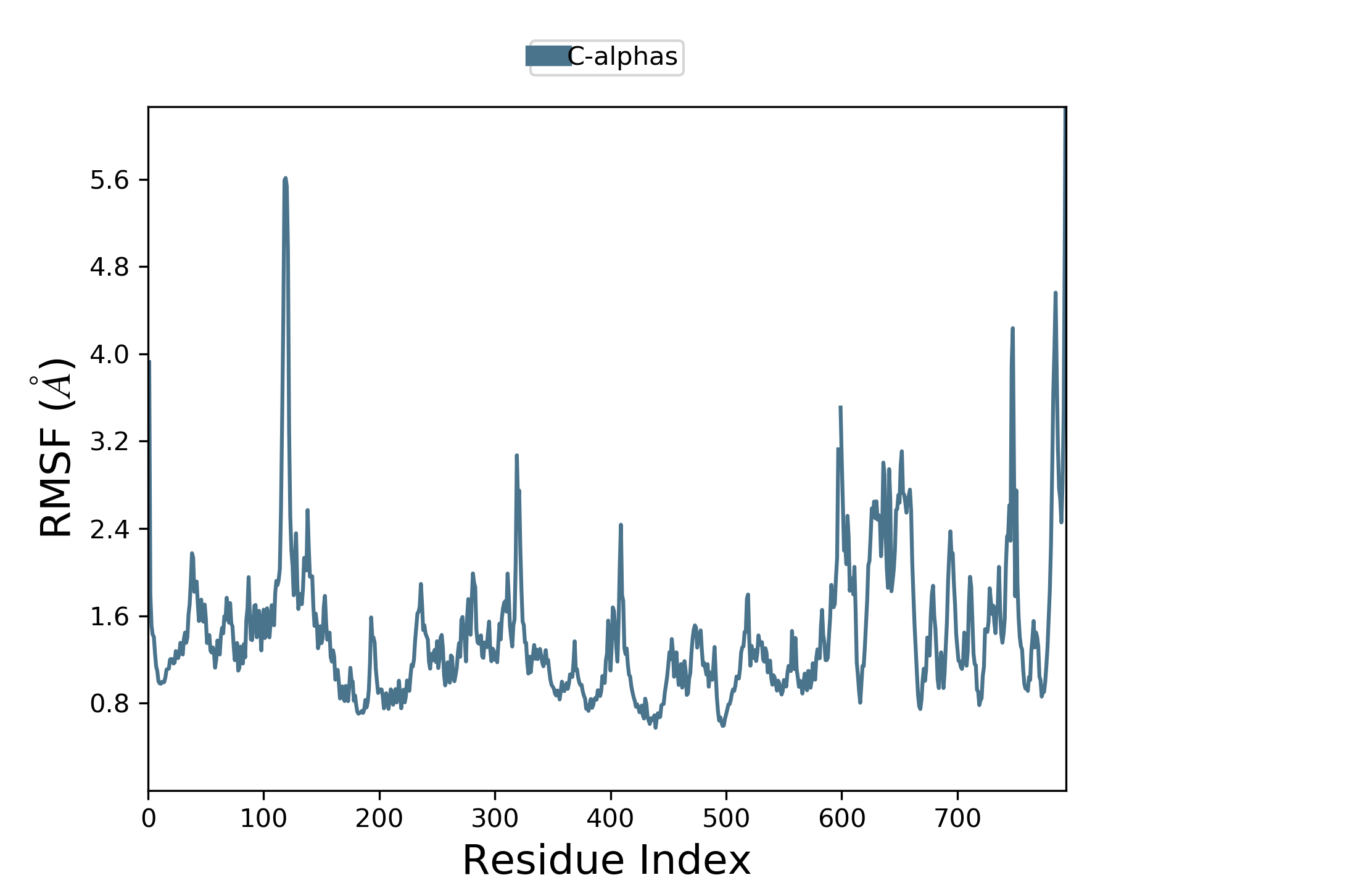

Supplement: Supplementary file 21 [file Image_11.TIF]

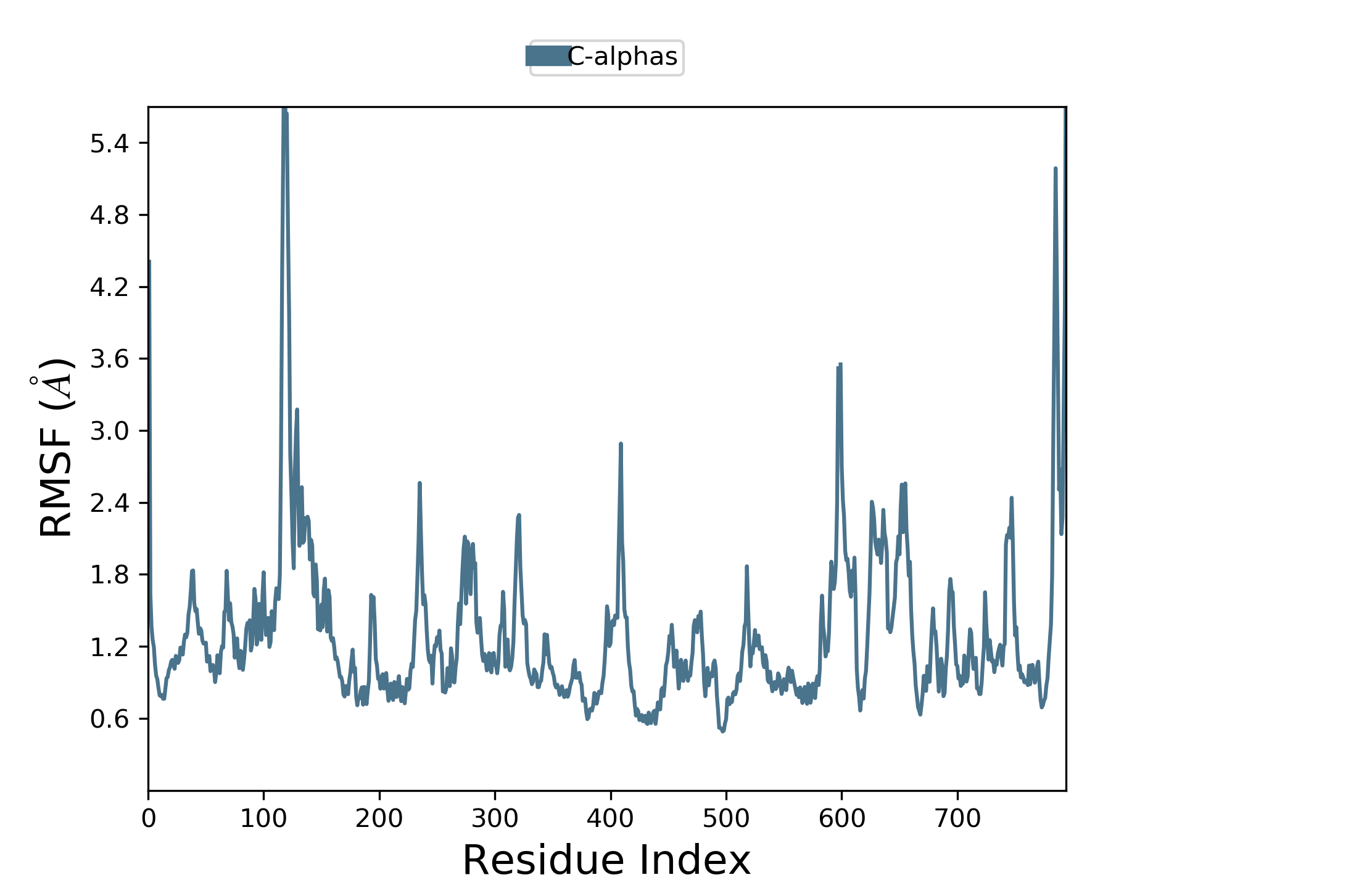

Supplement: Supplementary file 22 [file Image_12.TIF]

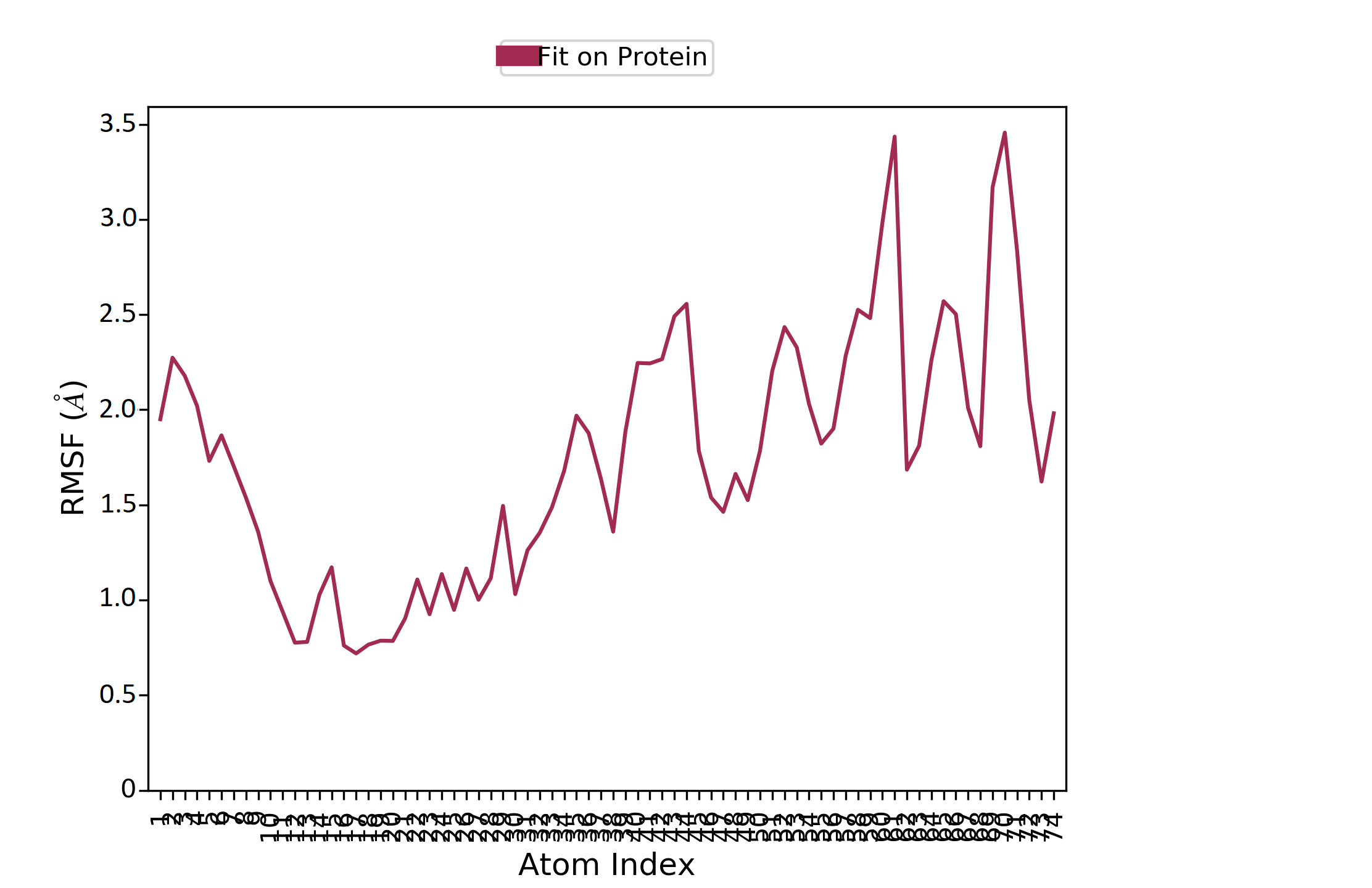

Supplement: Supplementary file 23 [file Image_13.TIF]

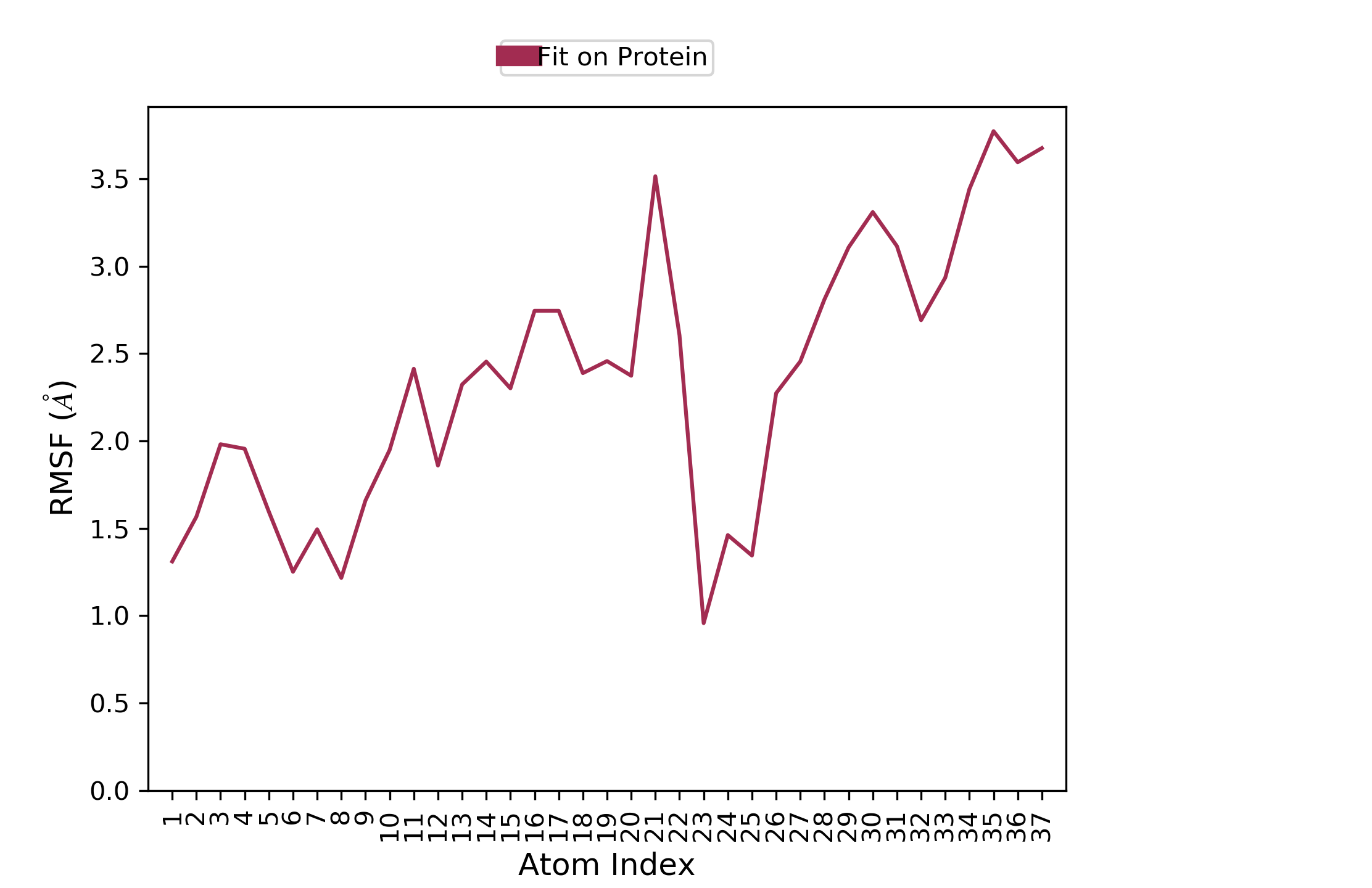

Supplement: Supplementary file 24 [file Image_14.TIF]

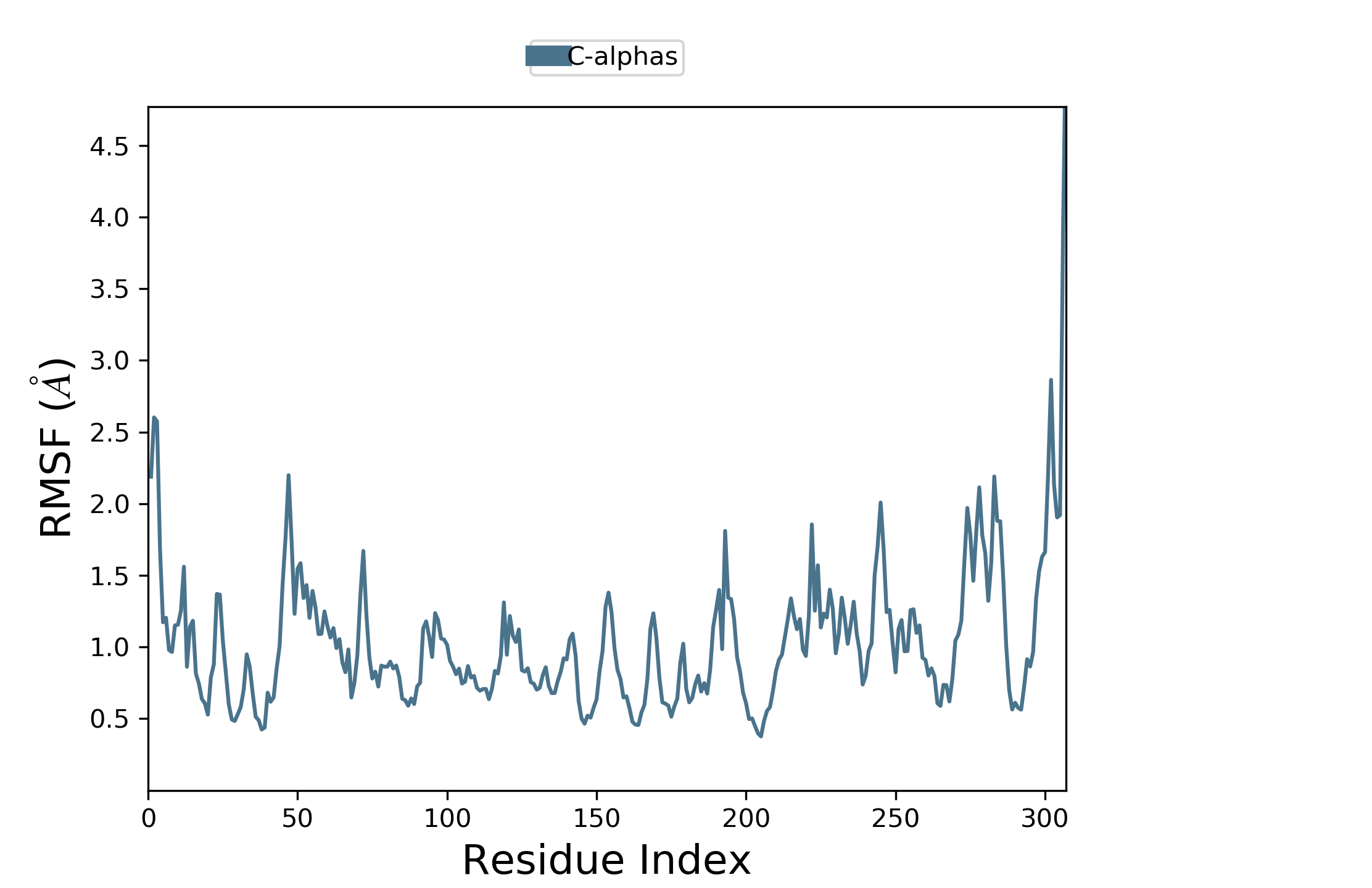

Supplement: Supplementary file 25 [file Image_15.TIF]
